# Retrieval of long DNA reads from herbarium specimens

**DOI:** 10.1093/aobpla/plad074

**Published:** 2023-11-08

**Authors:** Anne-Sophie Quatela, Patrik Cangren, Farzaneh Jafari, Thibauld Michel, Hugo J de Boer, Bengt Oxelman

**Affiliations:** Department of Biological and Environmental Sciences, University of Gothenburg, Box 463, 405 30, Gothenburg, Sweden; Gothenburg Global Biodiversity Center, Gothenburg, Box 463, 405 30, Sweden; Department of Biological and Environmental Sciences, University of Gothenburg, Box 463, 405 30, Gothenburg, Sweden; Department of Biology, Faculty of Basic Sciences, Lorestan University, P.O. BOX 6815144316, Khorramabad, Iran; Department of Plant Science, Center of Excellence in Phylogeny of Living Organisms, School of Biology, College of Science, University of Tehran, P.O. Box 14155-6455, Tehran, Iran; Tropical Diversity Research Department, Royal Botanic Garden of Edinburgh, 20A Inverleith Row, Edinburgh, EH3 5LRUK; Natural History Museum, University of Oslo, P.O. Box 1172 Blindern, 0318 Oslo, Norway; Department of Biological and Environmental Sciences, University of Gothenburg, Box 463, 405 30, Gothenburg, Sweden; Gothenburg Global Biodiversity Center, Gothenburg, Box 463, 405 30, Sweden

**Keywords:** Cross-contaminants, degraded DNA, herbarium specimens, long-read sequencing, *Silene*, target capture

## Abstract

High-throughput sequencing of herbarium specimens’ DNA with short-read platforms has helped explore many biological questions. Here, for the first time, we investigate the potential of using herbarium specimens as a resource for long-read DNA sequencing technologies. We use target capture of 48 low-copy nuclear loci in 12 herbarium specimens of *Silene* as a basis for long-read sequencing using SMRT PacBio Sequel. The samples were collected between 1932 and 2019. A simple optimization of size selection protocol enabled the retrieval of both long DNA fragments (>1 kb) and long on-target reads for nine of them. The limited sampling size does not enable statistical evaluation of the influence of specimen age to the DNA fragmentation, but our results confirm that younger samples, that is, collected after 1990, are less fragmented and have better sequencing success than specimens collected before this date. Specimens collected between 1990 and 2019 yield between 167 and 3403 on-target reads > 1 kb. They enabled recovering between 34 loci and 48 (i.e. all loci recovered). Three samples from specimens collected before 1990 did not yield on-target reads > 1 kb. The four other samples collected before this date yielded up to 144 reads and recovered up to 25 loci. Young herbarium specimens seem promising for long-read sequencing. However, older ones have partly failed. Further exploration would be necessary to statistically test and understand the potential of older material in the quest for long reads. We would encourage greatly expanding the sampling size and comparing different taxonomic groups.

## Teaser

Herbarium collections host more than 390 million specimens across 3100 institutions. They cover all continents, all taxonomic groups of plants. In the current biodiversity crisis, accessing the genetic information enclosed in collections is of crucial importance. However, only short DNA fragments could be sequenced because of the natural fragmentation of herbarium specimens’ genomes. For the first time, our study shows that even long DNA fragments can be sequenced from young herbarium specimens, collected after 1990. This raises hope for accessing genomic complexity from museum collections.

## Introduction

Herbaria host more than 390 million specimens across 3100 institutions (Thiers 2021), and represent a crucial biobank for research. Collections cover all continents, all biomes and all taxonomic groups of plants, algae and fungi. These biorepositories provide a solid source of metadata enabling the study of a wide range of questions. The collections are at the forefront of long-standing biological investigations in for example, taxonomy, systematics, floristics, biogeography and phenology (e.g. Heberling *et al.* 2019; López and Sassone 2019), but are also crucial to address anthropogenic challenges threatening biodiversity (Nualart *et al.* 2017; Meineke *et al.* 2018; Nic Lughadha *et al.* 2018; Lang *et al.* 2019). Modern threats to biodiversity, for example, climate change, habitat loss, have given rise to urgent needs such as quantifying biodiversity erosion, and, therefore, new ways to use herbaria (e.g. Nualart *et al.* 2017; Bieker and Martin 2018; Carine *et al.* 2018; Blom 2021) alongside with traditional ones. One of those modern ambitions is to maximize the potential of herbarium specimens as a source of genomic data (e.g. Jordon-Thaden *et al.* 2020; Marinček *et al.* 2022), in order to understand for example how agriculture, urbanization, deforestation, climate change and so on have impacted plant populations over time. The development of high-throughput sequencing during the last two decades (Lemmon and Lemmon 2013) and its application to herbaria has opened what Staats *et al.* (2013) coined a ‘genomic treasure trove’.

However, obtaining high-quality genomic data from dried specimens is challenging. After plant death, the DNA undergoes spontaneous damage such as strand break and an increase of C-to-T mutations (Staats *et al.* 2011; Weiß *et al.* 2016). This leads to relatively degraded genetic material and low DNA yields (Rogers and Bendich 1985; Staats *et al.* 2011; Weiß *et al.* 2016). Weiß *et al.* (2016) have estimated a per nucleotide fragmentation rate of 1.66 × 10^−4^ per year in the DNA of herbarium specimens. Besides the spontaneous effect of time on DNA integrity, postmortem DNA damages are also enhanced by preparation and preservation techniques (e.g. Forrest *et al.* 2019). Some preparation methods and preservation agents, used for centuries, are now known as catalyzing DNA degradation and fragmentation (Doyle and Dickson 1987; Pyle and Adams 1989; Harris 1993). Heat-drying, frequently used in the tropics, rapidly breaks down DNA into small fragments (Staats *et al.* 2011; Forrest *et al.* 2019). In addition, samples from the tropics have commonly been soaked in formalin or ethanol prior drying to conserve morphology over time. However, ethanol soaking is known to significantly affect DNA integrity (Doyle and Dickson 1987; Chase and Hills 1991; Srinivasan *et al*. 2002; Forrest *et al* 2019). All herbarium specimens are affected to a certain extent by postmortem DNA degradation, which tends to increase over time (Staats *et al.* 2011; Weiß *et al.* 2016).

Despite these obstacles, the fast-moving development in high-throughput sequencing has enabled significant achievements in herbariomics. Over the last decade, several studies have demonstrated that DNA damage in herbarium specimens is not insurmountable in phylogenomics (Staats *et al.* 2011, 2013; Hart *et al.* 2016; Brewer *et al.* 2019; Van Andel *et al.* 2019; McGaughran 2020; Manzanilla *et al.* 2022). Genome skimming (e.g. Cronn *et al.* 2008) and target capture/HybSeq (e.g. Hart *et al.* 2016; Van Andel *et al.* 2019; Manzanilla *et al.* 2022) have proven to work successfully with herbarium collections. The entire nuclear genome of *Arabidopsis thaliana* has been retrieved from several herbarium specimens collected between 1863 and 1993 (Staats *et al.* 2013; Exposito-Alonso *et al.* 2018). Full plastome assembly has been successfully achieved across 12 angiosperm families including 146-year-old specimens from tropical regions (Bakker *et al.* 2016), and large-scale genome skimming to retrieve cpDNA and rDNA from dozens of angiosperm families for DNA barcoding and phylogenomics are reported (Zeng *et al.* 2018; Alsos *et al.* 2020). Recently, studies associating herbarium specimens and HybSeq/target capture (Weitemier *et al.* 2014) have multiplied to unravel complex evolutionary histories and resolve both deep and shallow nodes. Hundreds of low-copy-nuclear genes were successfully retrieved from low-quality DNA in (sub)tropical plants such as *Ipomoea* with some specimens 180 years old (Hart *et al.* 2016), *Costus* (Valderrama *et al.* 2020), the woody angiosperm genus *Schefflera* (Shee *et al.* 2020), the highly diversified families Apocynaceae (Straub *et al.* 2020) and Asteraceae (Beck and Semple 2015; Jones *et al.* 2019), and even resolving population-level relationships in Euphorbiaceae (Villaverde *et al.* 2018), Poaceae (Van Andel *et al.* 2019) and Asteraceae (Manzanilla *et al.* 2022). Biologists also keep pushing the limits of herbarium specimens by extending their use beyond systematics. For example, historical collections helped assessing C3 to C4 transition from full chloroplast and ribosomal nuclear sequences from two *Sartidia* species (Besnard *et al.* 2014), studying repetitive elements and genome size evolution with organelle genomes and low-copy nuclear genes from *Nicotiana* (Dodsworth *et al.* 2018), assessing ploidy level with HybSeq/target capture in *Dioscorea* (Viruel *et al.* 2019). All these studies share a common and fundamental methodological similarity in that they are all based on short-read sequencing technology.

The short-read sequencing platform Illumina is probably the widest-used mass sequencing technology. It provides highly accurate reads, that is, much less than 1 % error rate on average per read (Stoler and Nekrutenko 2021), abundant data, is cost-efficient and has proven to work with poor quality libraries (Forrest *et al.* 2019). Even though this popular platform helps investigations in a wide range of biological questions, assembling more or less complex genomes with short reads (i.e. 2 × 150 base pairs (bp) and 2 × 300 bp paired-end reads are commonly used) is often difficult (e.g. Berger *et al.* 2013; Pollard *et al.* 2018). As opposed to short reads, long reads usually refer to 5–30 kb reads, but their strict definition is longer than or equal to 1000 bp (Pollard *et al.* 2018). Short read-related difficulties can be mitigated by long-read sequencing technologies (English *et al.* 2012; Lu *et al.* 2016; Li *et al.* 2018; Pollard *et al.* 2018; Jung *et al.* 2019; Michael and VanBuren 2020), for example, Pacbio SMRT and Nanopore Oxford Technology. For a long time, the accuracy of long reads has been a significant caveat compared to short-read technologies, with error rates as high as 10–15 % for Oxford Nanopore (Lu *et al.* 2016). Nowadays, HiFi reads (i.e. ‘high fidelity reads’, also known as ‘CCS reads’) from SMRT PacBio technology average at least 99.8 % accuracy per read (e.g. Wenger *et al.* 2019). This is due to CCS technology implemented by SMRT PacBio, standing for ‘circular consensus sequencing’. This technology uses a polymerase to read a single template molecule across multiple passes, increasing the accuracy after each pass and creating a highly accurate consensus from noisy individual subreads. Highly accurate long reads improve assembly contiguity compared to short reads assembly (e.g. Lu *et al.* 2016). Consequently, long reads are more advantageous than short ones to inform complex genome architecture (Lu *et al.* 2016) such as large structural variants, repeated motifs, transposable elements (Jung *et al.* 2019; Shahid and Slotkin 2020), high GC content (Shin *et al.* 2013), high levels of heterozygosity and polyploidy (Jung *et al.* 2012; Michael and VanBuren 2020). Over the last three decades, abundant literature has discussed the advantages and limits of herbarium specimens in genomics (McKain *et al.* 2018; Dodsworth *et al.* 2019; Bakker *et al.* 2020; Jordon-Thaden *et al.* 2020; Folk *et al.* 2021), but long read sequencing has usually been ignored from these reviews.

Because of degradation and fragmentation processes, herbarium specimens are assumed to be suitable for short-read techniques only (e.g. Kates *et al.* 2021). However, since time and preservation techniques influence DNA degradation stochastically, we predict that some long DNA fragments should be present also in herbarium material and therefore available for long-read sequencing. In addition, we hypothesize that samples with higher concentrations of long fragments will produce better sequencing results, that is, longer and more abundant on-target reads, more recovered loci, higher read depth, better coverage. Furthermore, depending on the level of taxonomic specificity of the baits, target capture (e.g. HybSeq) can overcome contamination from sources genetically distant from taxa of interest, contrary to methods such as shotgun sequencing (Gutaker and Burbano 2017) or to some extent PCR-based sequencing (i.e. Sanger sequencing with primers non-species-specific). DNA fragments from contaminants genetically distant from the baits are unlikely to be captured. However, target capture does not prevent contamination from closely related organisms. As a matter of fact, one of the strongest advantages of the technique might also be one of its pitfalls. Cross-contamination is particularly prone to happen when samples of different DNA quantities and qualities are treated simultaneously (Marinček *et al.* 2022), for example, when herbarium specimens of different ages are treated together. Here, we present a study aiming at retrieving long reads, defined as 1 kb or longer, through probe-based target capture (Gnirke *et al.* 2009) and sequenced with the long-read PacBio Sequel SMRT technology. We use custom *Silene*-specific baits to capture 48 low-copy nuclear markers in diploid and tetraploid *Silene* from 12 herbarium specimens collected between 1932 and 2019. Firstly, (i) we assess the proportion of long DNA fragments in libraries at different stages of the sample preparation. Secondly, we (ii) assess the level of cross-contamination. To finish, (iii) we quantify the proportion of on-target reads longer than 1000 bp.

## Material and Methods

### Sampling

Twelve specimens were sampled from herbarium sheets, belonging to four different sections of the genus *Silene*, collected from 1932 to 2019 (see Table 1). The fresh samples collected from 2018 to 2019 were dried while pressed between paper sheets, without mechanical heat. We do not have information about the drying methods used for the samples collected before 2018. However, because none of them are succulent plants, we make the assumption they were dried without mechanical heat.

**Table 1.** Twelve *Silene* specimens were sampled from 1932 to 2019, belonging to four sections: *Silene* sect. *Physolychnis*, *S*. sect. *Siphonomorpha*, *S.* sect. *Silene* and *S*. sect *Elisanthe* (see Jafari *et al*. 2020).

Species	Collection year	Taxonomic group (Sections in genus *Silene)*	Name used in text	Ploidy level
*S. acaulis* (L.) Jacq.	2019	*Siphonomorpha* Otth	2019 *S. acaulis*	diploid
*S. burchellii* Otth	2019	*Silene*	2019 *S. burchellii*	diploid
*S. noctiflora* L.	2018	*Elisanthe* (Fenzl) Fenzl	2018 *S. noctiflora*	diploid
*S. sachalinensis* F. Schmidt	2017	*Physolychnis* (Bentham) Bocquet	2017 *S. sachalinensis*	tetraploid
*S. acaulis* (L.) Jacq.	1994	*Siphonomorpha* Otth	1994 *S. acaulis*	diploid
*S. burchellii* Otth	1987	*Silene*	1987 *S. burchellii*	diploid
*S. noctiflora* L.	1981	*Elisanthe* (Fenzl) Fenzl	1981 *S. noctiflora*	diploid
*S. involucrata* ssp. *furcata* (Raf.) Petrovsky and Elven	1979	*Physolychnis* (Bentham) Bocquet	1979 *S. involucrata*	tetraploid
*S. acaulis* (L.) Jacq.	1969	*Siphonomorpha* Otth	1969 *S. acaulis*	diploid
*S. uralensis* (Rupr.) *Bocquet* ssp. *uralensis*	1959	*Physolychnis* (Bentham) Bocquet	1959 *S. uralensis*	diploid
*S. burchellii* Otth	1948	*Silene*	1948 *S. burchellii*	diploid
*S. rigens* Goldblatt and J. C. Manning	1932	*Elisanthe* (Fenzl) Fenzl	1932 *S. rigens*	diploid

### Sample preparation (extraction, size selection, library preparation, hybridization)

The samples were not treated in an ancient DNA lab (e.g. no bleaching of the surfaces before use, no restricted access to PPE sterile suits, etc), but measures were taken to minimize contamination. The lab facilities have separated spaces for pre-PCR and post-PCR sample handling. No fresh samples were treated concomitantly, significantly reducing the chance of contamination from fresh specimens. Pipettes and tips were sterilized under UV machines (i.e. dedicated to sterilization of lab items) for 30 min minimum before use. Benches and necessary materials were cleaned with manufactured cleaning solutions dedicated to this use. DNA extractions were executed in a lab free from PCR products. Size selection, library preparation and target enrichment were performed in a separate lab. Target enrichment with genus-specific RNA probes largely prevented alien contamination (i.e. contamination from sources distantly related to *Silene*) of the samples post-hybridization.

**Figure 1.**
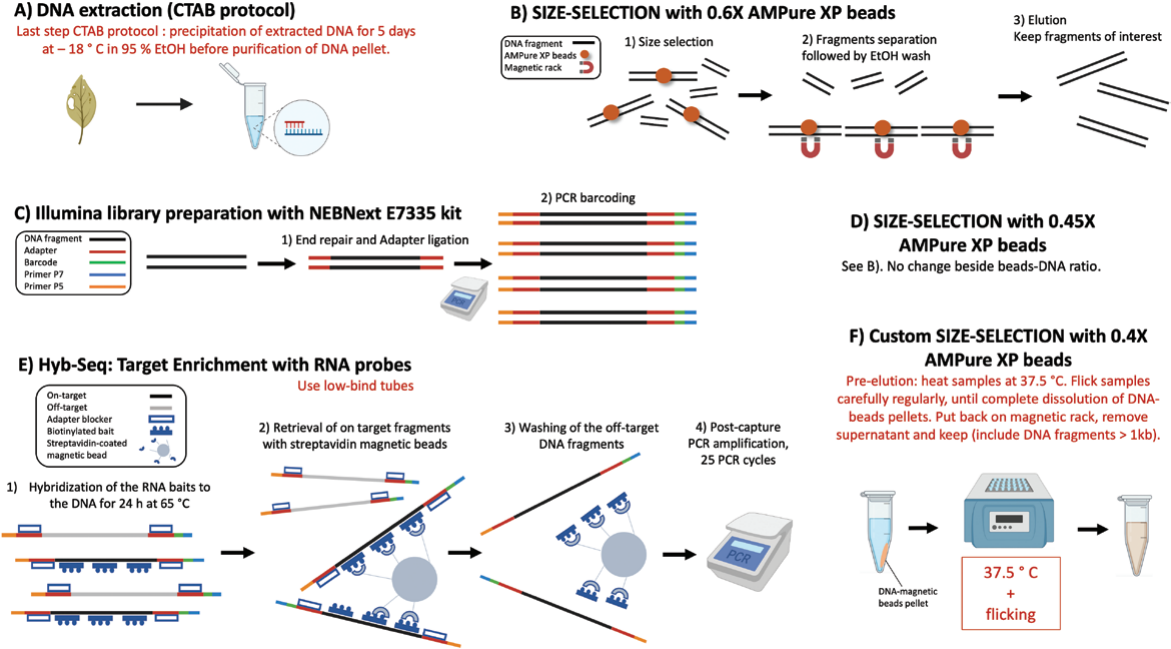
The different steps of the laboratory protocol are described in six steps (from steps A–F). Steps B, C, D and E follow protocols from the respective manufacturer. Steps A and F are customized for the purpose of the study. The custom steps are written in red.

The DNA was extracted using a CTAB protocol (Doyle and Doyle 1987). The extracted DNA was precipitated for 5 days in 95 % ethanol at −18 °C (see Fig. 1). DNA was purified and size selected with 0.6× AMPure XP beads (Beckman Coulter Inc., Brea, CA, USA), that is, selecting DNA fragments 500 bp and longer, according to the manufacturer’s protocol. The samples were barcoded with the NEBNext E7335 kit (New England Biosciences, Ipswich, MA, USA). The number of PCR cycles necessary for PCR barcoding was applied according to the manufacturer’s recommendations (see Supporting Information Appendix S7). Size selection with 0.45×AMPure XP beads, that is, selecting DNA fragments about 900 bp and longer, was performed after PCR barcoding. The probe-based target enrichment was performed on individual libraries (i.e. not pooled) with a set of customized *Silene*-specific probes (Patrik Cangren *et al.* pers. comm.) to enrich 48 low-copy nuclear loci, according to the ArborBioscience V4 manual (Ann Arbor, MI, USA). The hybridization was performed for 24 h at 65 °C (see Fig. 1). Following Witek *et al.* (2016)’s protocol, the captured libraries were amplified with 25 PCR cycles using the Kapa HiFi HotStart Amp kit (Roche Molecular Systems Inc., Pleasanton, CA, USA) according to the instructions from the manufacturer. 0.4×AMPure XP beads size selection, that is, selecting DNA fragments 1000 bp and longer, was performed on the amplified captured library. We extended the elution time to 30 min while leaving the solution at 37.5 °C in a thermoblock and flicking every 5 min, in order to get all the beads resuspended (see Fig. 1). Size selection was followed by quality assessment (see below). Finally, the amplified libraries were multiplexed in approximately equimolar proportions. SMRT bell library preparation and sequencing with the SMRT PacBio Sequel was performed by the Uppsala Genome Center (Science for Life Laboratory, Uppsala University). After SMRT bell ligation another size selection was performed.

### Quality control

Quality control was performed after CTAB extraction, after the first size selection, after PCR barcoding, after post-capture amplification, after last size selection and after library preparation. DNA quantity was measured with a Qubit fluorometer (Thermo-Fisher Scientific, Waltham, MA, USA). The BR (Broad-Range) Assay Kit (Thermo-Fisher Scientific) was used for post-extraction DNA quantity assessment, and the HS (High Sensitivity) Assay Kit (Thermo-Fisher Scientific) was used for the further steps, that is, after first size selection until the end of the protocol. Library purity was measured with NanoDrop 2000/2000c spectrophotometers (Thermo-Fisher Scientific). Distribution of DNA fragment length and molarity were measured on a TapeStation 4200 (Agilent Technologies, Santa Clara, CA, USA) with genomic DNA screentape (Agilent Technologies). The molarity of fragments > 1 kb was used as an approximation of long fragments distribution in libraries. Molarity of fragments>1 kb was summed up from Tapestation reports (see Supporting Information—Appendix S1) after each quality assessment, divided by the total molarity of each sample and multiplied by 100. However, the molarity of a single long DNA fragment being larger than the molarity of a shorter fragment, approximating fragments distribution by molarity ratios might be skewed toward longer fragments, overestimating the proportion of longer molecules.

### Demultiplexing, adapter trimming, duplicates and chimeras removal

Demultiplexing and adapter trimming were performed with Porechop (v 0.2.4, https://github.com/rrwick/Porechop), with default parameters and a custom barcode adapter file. Three flanking nucleotides (GAT-XXXXXX-GTG on forward reads; CAC-XXXXXX-ATC on reverse reads) were added to the six nucleotides barcodes to ensure correct identification. FastQC v0.11.9 (https://www.bioinformatics.babraham.ac.uk/projects/fastqc/) was used for the quality assessment of the sequencing. Mean read quality (Phred score) was estimated for each sample from the section ‘Per sequence quality scores’ in the fastqc reports. Geneious Prime version 2020.2.4 was used to remove duplicated reads with the plugin Dedupe from BBTools (version 1.0, Bushnell 2022) with default parameters (i.e. Kmer seed length 31, maximum edits 0, maximum substitution 0). Chimeras were removed with the plugin UCHIME (version 4.2, Edgar *et al.* 2011) from Geneious Prime version 2020.2.4 with default parameters.

### Detection of cross contaminants and removal of ambiguous reads

**Figure 2.**
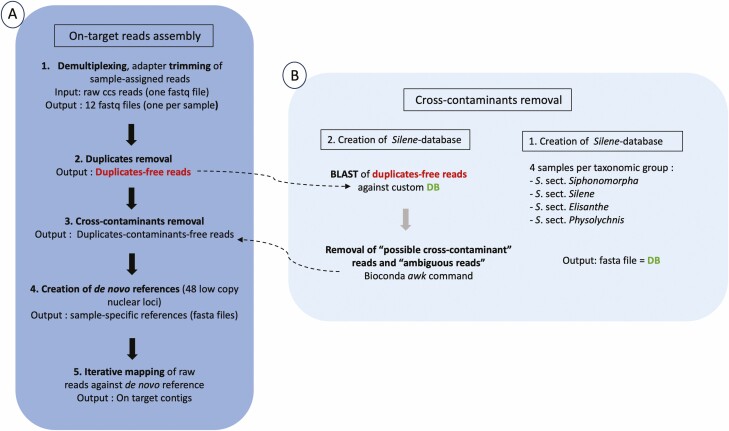
(A) The assembly of on-target reads into contigs go through five different steps, from raw ccs reads (A.1) to final contigs (A.5). The output of (A.2) is used as input to identify cross-contaminants in (A.3). The output of (A.3) is used as input in (A.4) to create sample-specific *de novo* references. The *de novo* references are used in (A.5) to iteratively map the reads. (B) In order to removed likely cross-contaminants, a custom *Silene*-database is created with the sequences from 16 samples divided in 4 taxonomic sections (i.e. 4 samples per section). These sequences are 48 low-copy nuclear loci enriched by custom *Silene*-baits. The duplicate-free reads in step (A.2) are blasted against this custom database and produce the output in (A.3).

A custom database was made using the makeblastdb function of BLAST + (Camacho *et al.* 2009) with sequences from the 48 target loci retrieved for four samples from each of the four sections in a different study (Patrik Cangren *et al.* pers. comm; see Supporting Information—Appendix S2-1 for sequences in fasta file; Fig. 2). Multiple alignments were performed with mafft (version 7, Katoh and Standley 2013) from Geneious Prime (default parameters, i.e. algorithm ‘auto’ selecting appropriate strategy according to sample size) and substitution distances were calculated with the R package ape (version 5.1, Paradis and Schliep 2019) with R studio (version ‘2023.3.0.386’, Posit Software PBC, Boston, MA, USA), function ‘dist.dna’ with default K80/Kimura substitution model. The genetic distances between sequences within the four sections ranged between 0.38 % and –0.9 %, and between 3.8 % and 7.9 % between the sections groups (see Supporting Information—Appendix S2-2). The species chosen to create the database were selected because no hybridization has ever been reported between members of these taxonomic groups and is considered very unlikely. The four *S. acaulis* samples (see Supporting Information—Appendix S2-1), representing the section *Siphonomorpha*, had an average of 0.38 % genetic distance (see Supporting Information—Appendix S2-2), the four section *Silene* samples (i.e. three samples *S. burchellii* and one *S. aethiopica*; see Supporting Information—Appendix S2-1) had on average 0.76 % genetic distance (see Supporting Information—Appendix S2-2), the four section *Physolychnis* samples (i.e. three *S. uralensis* and one *S. violascens*; see Supporting Information——Appendix S2-1) had 0.9 % genetic distance (see Supporting Information—appendix S2-2), and the four section *Elisanthe* samples (i.e. three *S. undulata* and one *S. saldanhensis*; see Supporting Information—Appendix S2-1) had 0.64 % genetic distance (see Supporting Information—Appendix S2-2). The trimmed duplicate- and chimera-free reads were blasted against the custom database with blastn (Camacho *et al.* 2009), for each query sequence only reporting high-scoring segment pairs (i.e. local alignment with no gaps that achieves one of the highest alignment scores in a given search https://www.ncbi.nlm.nih.gov/books/NBK62051/) for the first subject sequence (argument -max_target_seqs 1). We define as ‘possible cross-contaminants’ reads with a top-scoring match to the wrong taxonomic group, at least 95 % pairwise identity and alignment length longer than 600 bp. We define ‘ambiguous reads’ as reads less than 600 bp matching best to non-expected taxa. To visualize the distribution of ‘possible cross-contaminants’, we used R version 3.5.1 (R Core Team 2021) to plot such reads. To clean the fasta files from exogenous DNA, we adopted a conservative approach and removed both ambiguous reads and ‘possible cross-contaminants’. Both ‘possible cross-contaminants’ and ‘ambiguous reads’ IDs were retrieved from the blast output text files and removed from the fasta files with the Unix program awk. Read lengths of the duplicates-chimeras-contaminants free reads were summarized with the bioconda package bioawk (https://github.com/lh3/bioawk; see Supporting Information—Appendix S4).

### Assembly

Reads assembly was performed in two steps. First, the processed and cleaned reads (i.e. trimmed-, contaminant-, duplicate- and chimera-free reads) were assembled *de novo* with canu version 2.1 (Koren *et al.* 2017) to create internal *de novo* references for each sample. The consensus sequences from the *de novo* assemblies were matched with blastn (Camacho *et al.* 2009) against the *Silene* probe regions and the loci were identified with custom scripts. Second, the trimmed-, contaminant-, duplicate- and chimera-free reads (i.e. same reads used to create the *de novo* references) were then mapped and assembled into contigs through iterative mapping against the internal references using Geneious Prime (v. 2020.2.4) assembler. Twenty-five iterations with Medium sensitivity (fast) were performed. The mapped reads are defined as on-target reads in this study. The contigs were extracted from Geneious Prime (v. 2020.2.4) in .sam format (Li *et al.* 2009). They were converted into .bam files (Li *et al.* 2009) with Samtools (v. 1.4, Li *et al.* 2009). The average read depth per position, number of assembled bases and average coverage (i.e. percentage of reference recovered by the assembled reads) were calculated from the .bam files with function ‘coverage’ in Samtools (v. 1.4, Li *et al.* 2009). On-target ratios were calculated from the assembly reports in Geneious (i.e. number of mapped reads divided by the total number of trimmed reads after removing ‘inferred and ambiguous contaminants’, duplicates, and chimeras. Read length of the on-target reads was calculated with the bioconda package bioawk (https://github.com/lh3/bioawk) from the fasta files produced by the iterative mapping (see Supporting Information—Appendix S4).

### Read authentication

Traditionally, ancient DNA is authenticated through the identification of deamination patterns at the read extremities (e.g. Jónsson *et al.* 2013). This often helps identify contamination from modern DNA (e.g. Jónsson *et al.* 2013). In our case, target capture and mapping of the reads against sample-specific references limit the risk of including fresh and degraded exogeneous contaminants in the final contigs. In addition, we have identified and removed likely cross-contaminants. Therefore, we identify deamination patterns to characterize our samples, not to identify contamination. Here, we use mapdamage2 (Jónsson *et al.* 2013) with default parameters.

## Results

**Figure 3.**
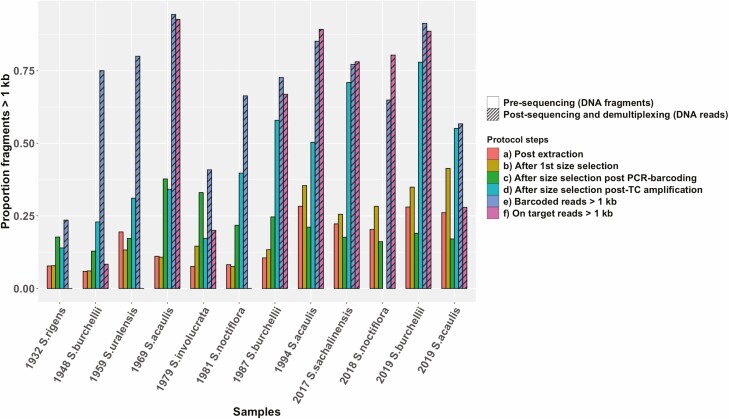
(A–D) Proportion of DNA fragments > 1 kb calculated between each round of AMPure XP beads size selection. The proportion of long fragments was calculated from the molarity of fragments > 1 kb assessed for each sample by Agilent Tapestation (see Supporting Information—Appendix S1; see Supporting Information—Appendix S5), divided by the total molarity per sample. (E and F) Proportions of actual read inserts > 1 kb. The absence of a purple (D) bar in 2018 *S. noctiflora* was due to a measurement failure. (E and F) Proportion of DNA reads > 1 kb, that is, after sequencing with SMRT PacBio Sequel and after demultiplexing. (E) Includes both off-target and on-target reads. (F) Includes only on-target reads.

Our results show a higher fragmentation level in samples collected before 1990, with 10.05 % (SD 0,045) DNA fragments > 1000 bp on average in samples post-extraction (see Supporting Information—Appendix S5; Fig. 3). Samples collected after 1990 exhibit 25 % (SD 0.067) DNA fragments > 1000 bp on average post-extraction (see Supporting Information—Appendix S5; Fig. 3). With some variation per step and per sample, the proportion of long fragments increases overall for all samples (Fig. 3, see Supporting Information—Appendix S5) between post-extraction (i.e. step ‘a’ in Fig. 3) and last size selection (i.e. step ‘d’ in Fig. 3). The overall increase of long DNA fragments between steps ‘a’ and ‘d’ ranges between 6.21 % and 49.88 % (see Supporting Information—Appendix S5). The increase is probably counteracted by the two PCR steps, as PCR is known to preferentially act on smaller fragments (e.g. Shagin *et al*. 1999).

The BLAST search of duplicate-chimera-free reads against the custom database enabled the identification of 126 ‘potential cross-contaminant’ reads (i.e. barcoded reads > 600 bp and with 95 % pairwise identity with their match in the custom database) in total (see Supporting Information—Appendices S6 and S7’). The percentage of ‘potential cross-contaminants’ of all lengths ranges from 0.03 % to 0.9 % of the barcoded reads with an average 0.3 % across the nine samples that yielded reads (see Supporting Information—appendices S6 and S7).

A total of 44 091 duplicate reads were identified from the fastq files, that is, 42.6 % of the sample-assigned (i.e. barcoded) reads (see Supporting Information—Appendix S7). The duplication level varies between 20 % (1994 *S. acaulis*) and 61 % (1981 *S. noctiflora*) (see Supporting Information—Appendix S7). Higher duplication levels are found for samples collected before 1990, with 50 % duplication on average (see Supporting Information—Appendix 6). Samples collected after 1990 exhibit on an average 38 % duplication level (see Supporting Information—Appendix 6). For the same number of post-enrichment PCR cycles (i.e. 25 cycles), the molarity of DNA fragments > 1000 bp was much lower for the samples collected before 1990, that is, 1.54 nmol/L of fragments > 1000 bp, than those collected after 1990, that is, 21.935 nmol/L of fragments > 1000 bp (see Supporting Information—Appendix S7).

**Figure 4.**
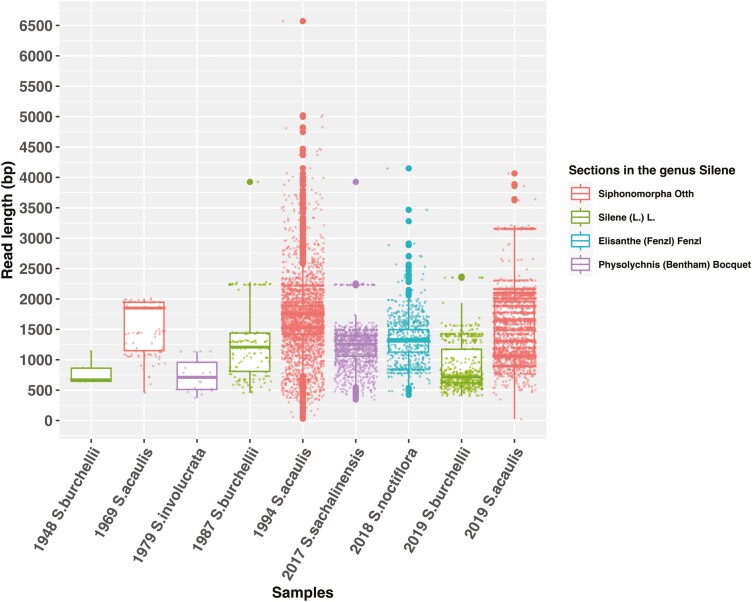
The read length is plotted in base pair (bp) for each on-target read (i.e. read mapped against the internal *Silene* references assembled *de novo*). The samples are identified by their sampling year in the sample name and the taxonomic section by colour.

A total of 103 463 sample-assigned ccs (i.e. circular-consensus-sequencing) reads were obtained from sequencing with one PacBioSMRT Sequel cell from the 12 included samples (see Supporting Information—Appendix S7). The average read quality is 99.99 % (i.e., Phred score 40, Supporting Information—Appendix S7) for each sample. The *de novo* assembly of the barcoded reads produced references for nine samples. Even though the limited sampling size does not allow for statistical testing of a correlation between age and sequencing success, three failed samples (i.e. ‘1932 *S. rigens*’, ‘1959 *S. uralensis*’, ‘1981 *S.noctiflora*’) were collected before 1990, while all samples collected after 1990 yielded on-target reads > 1000 bp. The oldest sample of the dataset, that is, ‘1932 *S. rigens*’ did not yield any on-target reads, as well as ‘1959 *S. uralensis*’ and ‘1981 *S.noctiflora*’. The iterative mapping of the 103 463 sample-assigned reads against the *de novo* references mapped 10 357 reads in total, that is, 10 % of the total barcoded reads are on-target (see Supporting Information—Appendix S7). All nine samples produced on-target reads longer than 1000 bp (see Supporting Information—Appendix S7; Fig. 4), with six samples yielding average and median on-target length longer than 1000 bp. The average length per sample of on-target reads varies from 739 bp to 1670 bp. On-target ratio varies between 1.6 % (12 reads) and 30.9 % (3469 reads).

One group of samples collected after 1990 had pre-sequencing molarity of long fragments (>1 kb) between 8.63 nmol/l and 38.18 nmol/l exhibit higher numbers of long on-target reads (Fig. 4; see Supporting Information—Appendix S7; see Supporting Information—Appendix S8), between 167 and 3402 reads (see Supporting Information—Appendix S7). This group has consistently higher sequencing success (measured as average read length, number on-target reads, number of recovered loci, read depth, total contig length) (see Supporting Information—Appendix S7). Within this group, the number of on-target reads > 1 kb varies from 598 to 3839, with on average length from 2367 bp to 6570 bp. The total contig length per sample ranges from 83139 to 437390 bp (see Supporting Information—Appendix S7), that is, about four times 104 000 bp which is the total target length. The average read depth varies from 5.76 to 23.36. The number of recovered loci varies from 34/48 to 48/48.

Samples collected before 1990 present much lower long fragments’ molarity, ranging between 0.68 nmol/l and 2.97 nmol/l (see Supporting Information—Appendix S7; see Supporting Information—Appendix S8) and few on-target reads > 1 kb (Fig. 4; see Supporting Information—Appendix S7; see Supporting Information—Appendix S8), between 1 and 114 reads. This group includes the three failed samples (see Supporting Information—Appendix S7). Besides the three samples that did not yield useful sequences, the number of on-target reads ranges from 20 to 145, with an average length from 739 to 1562 bp. The total contig length per sample varies from 18554 to 57204 bp (see Supporting Information—Appendix S7). The average read depth varies from 0.48 to 3.46. The number of recovered loci varies from 6/48 to 25/48 (see Supporting Information—Appendix S7).

Deamination analyses on the .bam files show two groups of samples, the ones collected before 1990 with higher deamination rates than those collected after this date (see Supporting Information—Appendix S3). Deamination patterns are noticeably increasing with the sampling age. The oldest sample (i.e. collected in 1932) shows a deamination frequency up to 1 at some positions of the 25 first bp of its reads, while younger specimens exhibit a deamination frequency between 0 and 0.15 for most of the first 25 bp (see Supporting Information—Appendix S3).

## Discussion

### Length of on-target reads and sequencing success assessment

Our results suggest that young herbarium specimens (i.e. 30 years old and younger) may hold an unsuspected high potential for long read sequencing, but suggest less promise for older specimens. Nine samples out of 12 yielded on-target reads longer than 1000 bp, with considerable variation in the number of on-target reads and read lengths. Specimens collected between 1990 and 2019 yield between 167 and 3403 on-target reads > 1 kb. They enabled recovering between 34 loci and 48 (i.e. all loci recovered). Three samples from specimens collected before 1990 did not yield on-target reads > 1 kb. The four other samples collected before this date yielded up to 144 reads and recovered up to 25 loci. Through short read sequencing, Hart *et al.* (2016) enriched hundreds of low-copy nuclear genes from the neotropical genus *Inga*, with herbarium specimens as old as 180 years. Michel *et al.* (2022) and Manzanilla *et al.* (2022) also had some success with short fragments from old herbarium specimens, but Woudstra *et al.* (2021) were less successful with the succulent genus *Aloe*. As our study was aimed at testing the possibility of retrieving long reads, we do not know how much sequence information might have been in the short, discarded fragments.

As post-capture DNA concentrations were too low to be quantified, we performed 25 PCR cycles as recommended by Witek *et al.* (2016). Michel *et al.* (2022) report up to 22 PCR cycles post-enrichment with short Illumina reads. Although post-enrichment amplification is, for instance, necessary for quality control and sequencing requirements, high duplication levels waste sequencing power. Avoidance of PCR amplification of unknown DNA quantities would make multiplexing unpredictable, in addition to not complying with the manufacturer’s recommendations. However, we are unaware of systematic studies testing the lower limits for DNA quantities for successful sequencing. After 25 cycles of post-enrichment amplification, our samples collected before 1990 yielded very low molarity of long DNA fragments.

SMRT PacBio Sequel outputs c. 500 000 hifi reads per sequencing run, while the Illumina platform can output millions of short reads. However, fewer but longer reads enabled us to retrieve most of the 48 markers for samples collected after 1990, that is, with good pre-sequencing concentrations of the captured DNA. This might be due to the fact that few accurate long reads compensate for having more short reads. This would not be advantageous with long reads exhibiting high error rates. However, the accuracy of short and long reads is about to be comparable, with less than one 1 % error rate on average per read in both platforms (e.g. Wenger *et al.* 2019; Stoler and Nekrutenko 2021).

### Preventing exogenous contamination and cross-contaminants

A simple size selection protocol enabled us to increase the proportion of long fragments for sequencing. Although three samples collected before 1990 showed an increased proportion of long DNA fragments during the quality controls, they did not yield long on-target reads. This suggests that they might mostly be off-target fragments, perhaps including exogenous DNA (e.g. microbial DNA present on herbarium sheets, Bieker *et al.* 2020). Characterization of deamination patterns is often used to identify modern contaminants in ancient DNA (e.g. Jónsson *et al.* 2013). Deamination rates in on-target reads from samples collected before 1990 are much higher than in the literature (Bieker *et al.* 2020). The cause of those high rates is unclear. It seems unlikely that contaminants were included in the contigs. Target capture is, in theory, a method preventing from sequencing contaminants and off-target fragments. However, post-hybridization washing is a critical step that can lead to the sequencing of barcoded off-target fragments, if not efficiently washed off. Assembly of sample-specific references is routinely performed in target enrichment analyses (e.g. Woudstra *et al.* 2021; Michel *et al.* 2022). These limit the risk of including exogenous DNA in the final sequences. However, even though alien contaminants are unlikely to be included in final sequences produced with target capture data, closely related cross-contaminants are problematic if not removed. Our approach uses a database of known sequences from taxa handled at the same time in the laboratory. This requires a significant genetic distance gap to the potential cross-contaminants. In this case, we have such a gap between the other taxonomic sections, but not within. Old herbarium material will often have low concentrations of endogenous long DNA fragments, and could therefore be more sensitive to cross-contamination from DNA from other organisms. Three of our older specimens did not yield any on-target reads at all, in one case despite having a relatively large number of sequenced reads (i.e. off-target reads). We consider our laboratory procedures efficient to prevent serious cross-contamination but insufficient to avoid exogenous contaminants with certainty. Herbarium specimens might, therefore, ideally be treated in the same fashion as ancient DNA to cross off this risk (Shepherd and Perrie 2014; Bieker *et al.* 2020), with a minimum requirement to separate pre- and post-PCR laboratories (Pääbo *et al.* 2004; Latorre *et al.* 2020).

### When is long-read sequencing worthwhile?

Even though the small sampling size of our study does not allow for a detailed model for the correlation between sample age and DNA fragmentation, this relationship has been demonstrated before (Rogers and Bendich 1985; Weiß *et al.* 2016). Hence, older samples are less suitable for long-read sequencing than younger ones. Samples with higher pre-sequencing molarity of long fragments also seem to be younger ones and yield more long on-target reads. Older samples exhibit lower concentrations and fewer or no long on-target reads. On average, older specimens tend to produce more fragmented libraries, less DNA, fewer long reads and fewer on-target reads in absolute numbers. However, our results indicate that, in younger specimens, the proportion of long on-target reads may be relatively high if the shorter fragments are significantly reduced prior to sequencing. Multiplexing heavily degraded and fresh samples may also reduce the success of retrieving on-target long fragments of the former category. We propose that the isolation and retrieval of long DNA fragments from degraded material should be more explored by future research.

Some old specimens might yield similar results to younger ones if they are dried without mechanical stress and well-preserved. Nevertheless, this might be specimen-specific, and probably unlikely with (sub)tropical specimens requiring active drying (e.g. drying in an oven). At least one of our samples (*S. acaulis*, 1994) shows comparable and even better average read length and locus recovery than younger ones. Even though time undeniably affects DNA quality, samples should not be discarded from long-read sequencing studies solely based on their age. Nevertheless, it is unlikely to obtain sequence quality comparable to those from fresh material, and one might have to handle disparate locus recovery (i.e. missing data) across samples. Several extractions per specimen might lead to better recovery than our results. However, old herbarium specimens are intrinsically precious and best practices tend to limit their destruction. Therefore, multiple extractions per specimen should be considered cautiously, especially for type material or old specimens sampled from vulnerable or extinct populations. Supernatants from size selection may be saved for additional short-read sequencing to complement long reads. Depending on financial and logistic resources, a combination of long- and short-reading sequencing techniques might be preferable.

### Conclusions and perspectives

Even though the superiority of long reads over short ones has been abundantly discussed in literature, short-read sequencing is often the only high-throughput approach used to unlock genetic information from degraded material. To this day, we are aware of only one review that discusses the possibility of sequencing tissue archives with long-read platforms (Blom 2021). No empirical study has been carried out on this question before ours. Our study shows that on-target DNA fragments > 1 kb can be sequenced in *Silene* herbarium specimens, at least if collected less than 30 years ago. Yet our approach remains limited for older samples. In regard to the small sampling size and taxonomic range of our study, our conclusions should be treated with vigilance, especially regarding older specimens. Extrapolation to other groups known for their fragmentation-prone drying methods, for example, tropical taxa , should be cautious. Nonetheless, even though our study is not aimed at promoting a switch of standard practice from short-read sequencing to long one, it may encourage further explorations of the potential of herbarium material with long-read sequencing.

## Supporting Information

The following additional information is available in the online version of this article –

‘Appendix S1’: APPENDIX_S1_tapestation.pdf Tapestation results of DNA fragments distribution.

‘Appendix S2-1’: APPENDIX_S2_1_gendistance_supermatrixTOT.xlsx matrix showing genetic distance between samples used in custom database. Used for cross-contaminants removal.

‘Appendix S2-2’: APPENDIX_S2_2_SileneDB16specimens.fasta sequences files of the samples used in custom database. Used for cross-contaminants removal.

‘Appendix S3’, ‘Appendix S5’, ‘Appendix S6’ and ‘Appendix S8’ are merged in Appendix S3_S5_S6_S8.pdf. They are figures respectively showing: ‘Appendix S3’ is a figure showing the deamination patterns; ‘Appendix S5’ is a table quantifying fragments distribution from the tapestation results; ‘Appendix S6’ is a figure showing the level of cross-contamination; ‘Appendix S8’ is a figure showing the link between molarity and number of long on-target reads.

’Appendix S4-1’: including excel sheets with read length before mapping (including on and off-target reads).

’Appendix S4-2’: including excel sheets with read length after mapping (including on-target reads).

’Appendix S7’: APPENDIX_S7.xlsx excel sheet with metrics related to sequencing success and cross contaminations.

Voucher_information.xlsx excel sheet with voucher information.

plad074_suppl_Supplementary_Appendix_S1_1

plad074_suppl_Supplementary_Appendix_S1_2

plad074_suppl_Supplementary_Appendix_S1_3

plad074_suppl_Supplementary_Appendix_S1_4

plad074_suppl_Supplementary_Appendix_S1_5

plad074_suppl_Supplementary_Appendix_S2_1

plad074_suppl_Supplementary_Appendix_S2_2

plad074_suppl_Supplementary_Appendix_S3_S5_S6_S8

plad074_suppl_Supplementary_Appendix_S4_1

plad074_suppl_Supplementary_Appendix_S4_2

plad074_suppl_Supplementary_Appendix_S4_3

plad074_suppl_Supplementary_Appendix_S4_4

plad074_suppl_Supplementary_Appendix_S4_5

plad074_suppl_Supplementary_Appendix_S4_6

plad074_suppl_Supplementary_Appendix_S4_7

plad074_suppl_Supplementary_Appendix_S4_8

plad074_suppl_Supplementary_Appendix_S4_9

plad074_suppl_Supplementary_Appendix_S4_10

plad074_suppl_Supplementary_Appendix_S4_11

plad074_suppl_Supplementary_Appendix_S4_12

plad074_suppl_Supplementary_Appendix_S4_13

plad074_suppl_Supplementary_Appendix_S4_14

plad074_suppl_Supplementary_Appendix_S4_15

plad074_suppl_Supplementary_Appendix_S4_16

plad074_suppl_Supplementary_Appendix_S4_17

plad074_suppl_Supplementary_Appendix_S4_18

plad074_suppl_Supplementary_Appendix_S4_19

plad074_suppl_Supplementary_Appendix_S7

plad074_suppl_Supplementary_Voucher_information

## Data Availability

Sequencing data in this study are available at https://zenodo.org/record/8419069.
