# Supplementary material for: Retrieval of long DNA reads from herbarium specimens
Source: AoB Plants. 2023 Nov 8;15(6):plad074. doi: 10.1093/aobpla/plad074 (PMC10735254; doi:10.1093/aobpla/plad074)
Supplement: plad074_suppl_Supplementary_Appendix_S1_1 [file plad074_suppl_supplementary_appendix_s1_1.pdf]

Filename: 2019-06-27-01 after CTAB extraction.gDNA

Gel Image

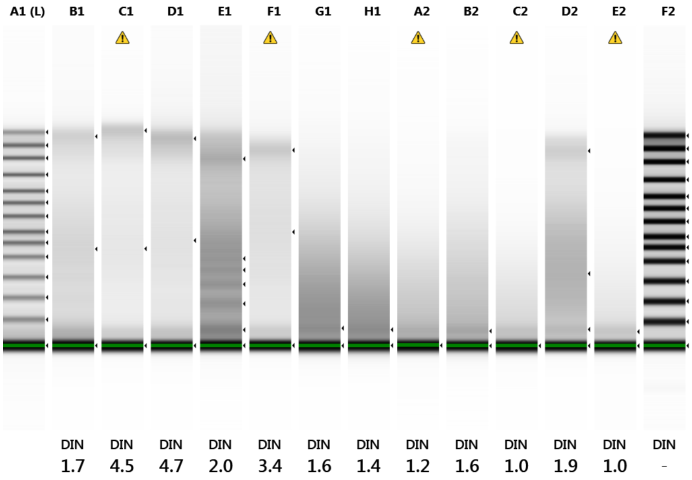

Default image (Contrast 100%)

Sample Info

Well	DIN	Conc. [ng/µl]	Sample Description	Alert	Observations
A1	-	20.0	Ladder		Ladder
B1	1.7	10.4	1 CAT1		
C1	4.5	7.30	2 CAT1	⚠	Sample concentration outside recommended range
D1	4.7	10.4	3 CAT1		
E1	2.0	28.9	4 CAT1		
F1	3.4	9.66	5 CAT2	⚠	Sample concentration outside recommended range
G1	1.6	16.3	6 CAT2		
H1	1.4	13.6	7 CAT2		
A2	1.2	6.91	8 CAT2	⚠	Sample concentration outside recommended range
B2	1.6	10.2	9 CAT3		
C2	1.0	3.59	10 CAT3	⚠	Sample concentration outside functional range for DIN
D2	1.9	17.5	11 CAT3		
E2	1.0	3.46	12 CAT3	⚠	Sample concentration outside functional range for DIN
F2	-	62.0	Ladder		Ladder run as sample

 © Agilent Technologies, Inc. 2016

A1: Ladder

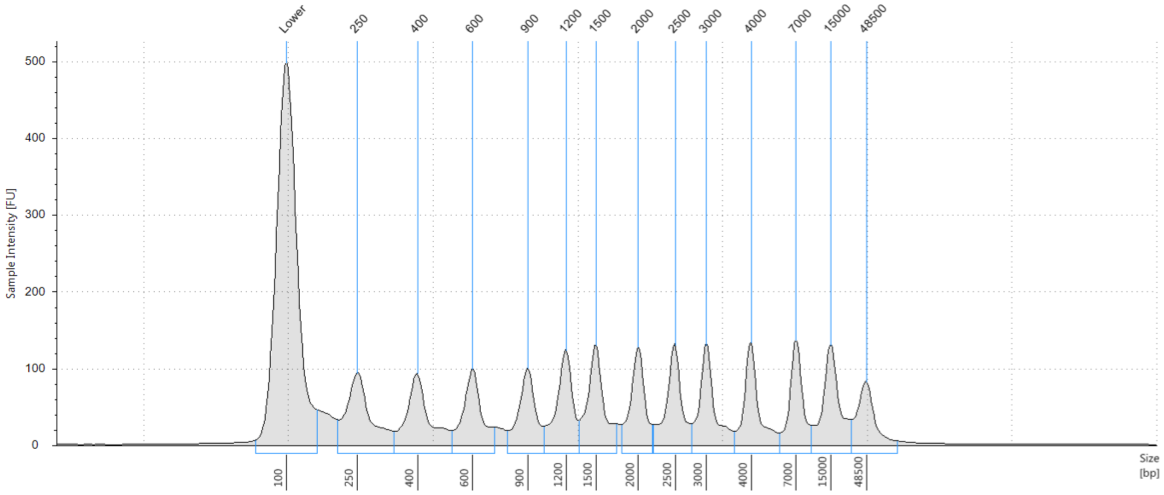

Sample Table

Well	DIN	Conc. [ng/µl]	Sample Description	Alert	Observations
A1	-	20.0	Ladder		Ladder

Peak Table

Size [bp]	Calibrated Conc. [ng/µl]	Assigned Conc. [ng/µl]	% Integrated Area	From [bp]	To [bp]	Peak Comment	Observations
100	8.50	8.50	-	67	149		Lower Marker
250	1.75	-	9.03	195	334		
400	1.57	-	8.11	334	516		
600	1.39	-	7.17	516	704		
900	1.30	-	6.72	774	1019		
1200	1.53	-	7.88	1019	1322		
1500	1.63	-	8.43	1322	1725		
2000	1.42	-	7.33	1786	2177		
2500	1.57	-	8.11	2193	2759		
3000	1.54	-	7.96	2759	3595		
4000	1.49	-	7.67	3595	5686		
7000	1.37	-	7.09	5686	9822		
15000	1.69	-	8.70	9822	23576		
48500	1.12	-	5.79	23576	>60000		

 © Agilent Technologies, Inc. 2016

19123
S. sachalinensis 2017

B1: 1 CAT1

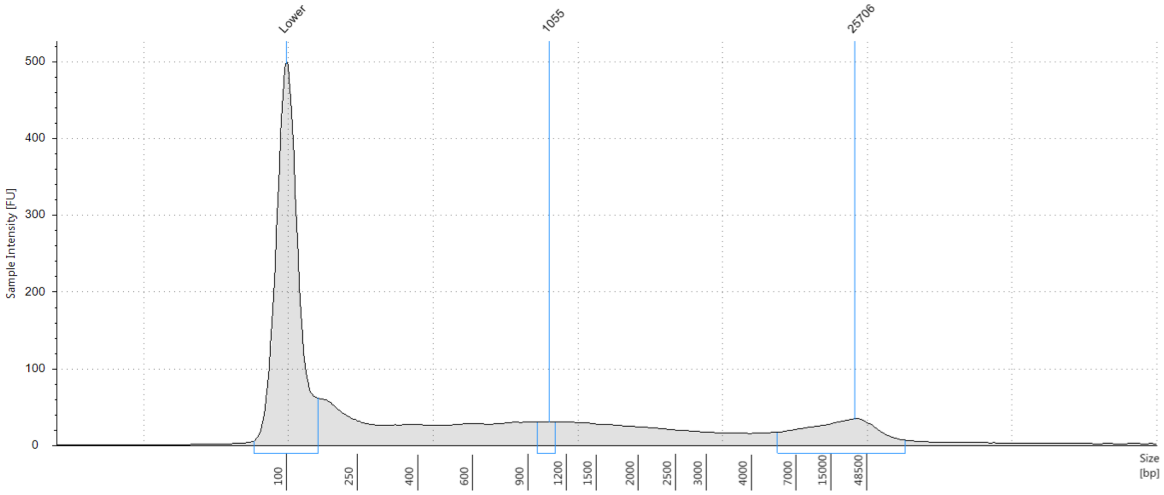

Sample Table

Well	DIN	Conc. [ng/µl]	Sample Description	Alert	Observations
B1	1.7	10.4	1 CAT1		

Peak Table

Size [bp]	Calibrated Conc. [ng/µl]	Assigned Conc. [ng/µl]	% Integrated Area	From [bp]	To [bp]	Peak Comment	Observations
100	8.50	8.50	-	65	151		Lower Marker
1055	0.389	-	12.27	966	1108		
25706	1.90	-	59.78	5558	>60000		
-	-	-	-	-	-		Sample Well

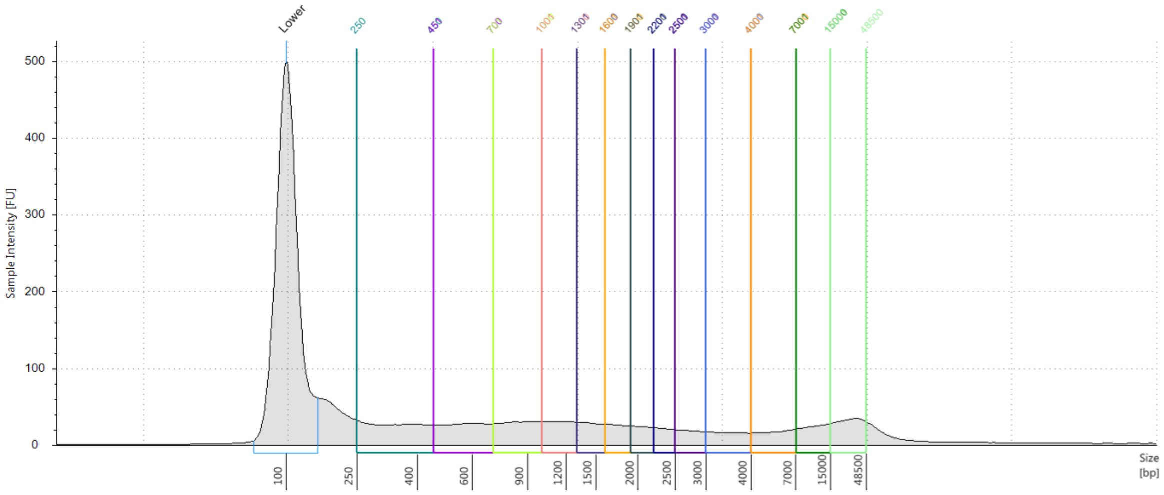

Region Table

From [bp]	To [bp]	Average Size [bp]	Conc. [ng/µl]	Region Molarity [nmol/l]	% of Total	Region Comment	Color
250	450	342	1.35	6.53	12.96		
451	700	574	1.06	3.00	10.14		
701	1000	850	0.954	1.81	9.16		
1001	1300	1155	0.724	1.01	6.96		
1301	1600	1460	0.532	0.585	5.11		

1601	1900	1759	0.460	0.421	4.42		
1901	2200	2065	0.358	0.280	3.44		
2201	2500	2370	0.302	0.207	2.90		
2501	3000	2759	0.372	0.221	3.58		
3001	4000	3488	0.465	0.221	4.47		
4001	7000	5541	0.515	0.157	4.95		
7001	15000	11038	0.555	0.0857	5.33		
15001	48500	25026	0.762	0.0534	7.32		

© Agilent Technologies, Inc. 2016

25463
S. acaulis 2019

C1: 2 CAT1

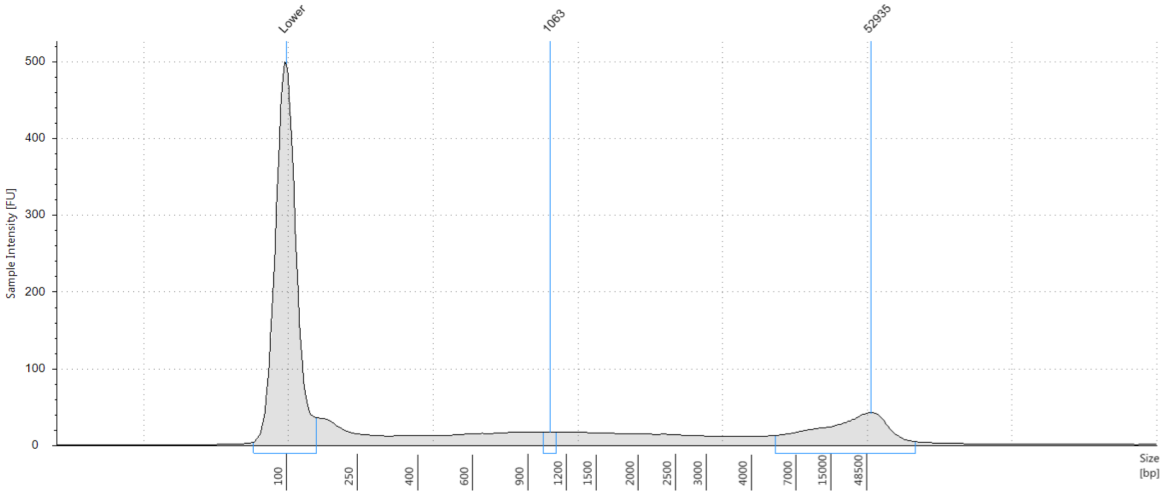

Sample Table

Well	DIN	Conc. [ng/µl]	Sample Description	Alert	Observations
C1	4.5	7.30	2 CAT1	⚠	Sample concentration outside recommended range

Peak Table

Size [bp]	Calibrated Conc. [ng/µl]	Assigned Conc. [ng/µl]	% Integrated Area	From [bp]	To [bp]	Peak Comment	Observations
100	8.50	8.50	-	65	148		Lower Marker
1063	0.167	-	5.17	1012	1117		
52935	2.27	-	70.42	5430	>60000		
-	-	-	-	-	-		Sample Well

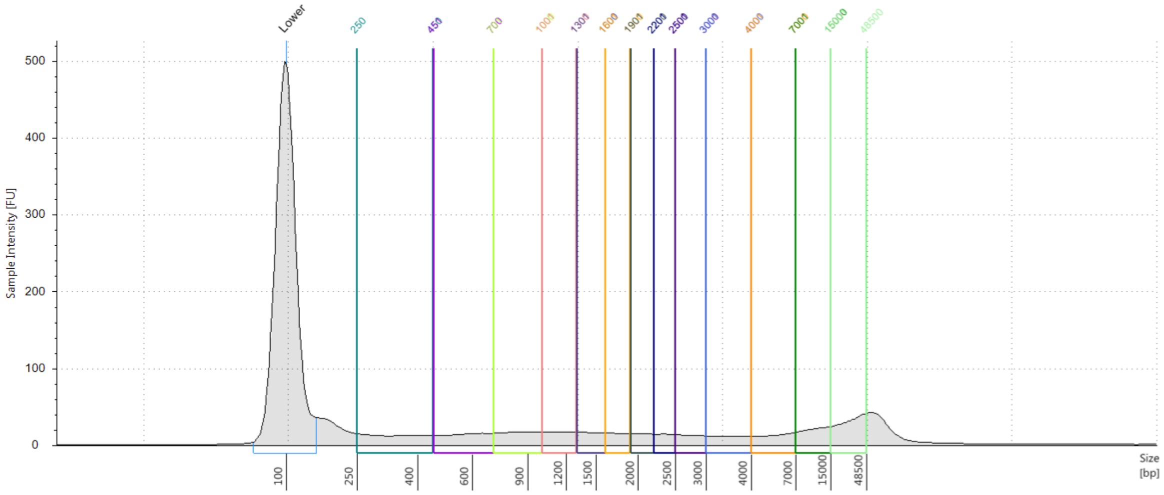

Region Table

From [bp]	To [bp]	Average Size [bp]	Conc. [ng/µl]	Region Molarity [nmol/l]	% of Total	Region Comment	Color
250	450	344	0.671	3.32	9.19		
451	700	576	0.575	1.66	7.88		
701	1000	851	0.547	1.05	7.49		
1001	1300	1153	0.422	0.597	5.78		

1301	1600	1463	0.334	0.372	4.58		
1601	1900	1764	0.271	0.251	3.72		
1901	2200	2069	0.247	0.195	3.39		
2201	2500	2366	0.212	0.147	2.91		
2501	3000	2764	0.281	0.168	3.85		
3001	4000	3511	0.355	0.169	4.87		
4001	7000	5549	0.406	0.124	5.57		
7001	15000	10925	0.507	0.0787	6.94		
15001	48500	26550	0.845	0.0564	11.58		

 © Agilent Technologies, Inc. 2016

23699
S. burchellii 2019

D1: 3 CAT1

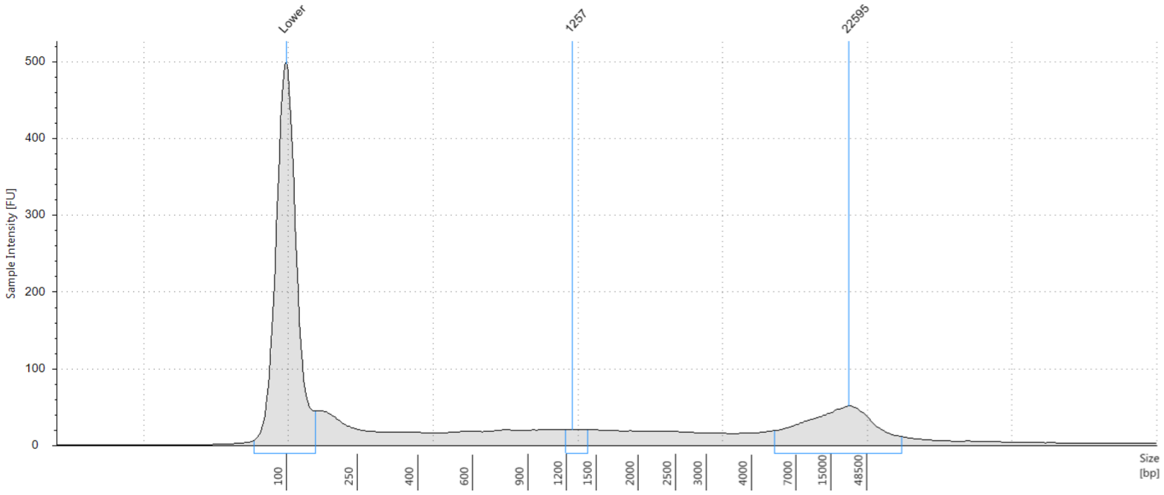

Sample Table

Well	DIN	Conc. [ng/µl]	Sample Description	Alert	Observations
D1	4.7	10.4	3 CAT1		

Peak Table

Size [bp]	Calibrated Conc. [ng/µl]	Assigned Conc. [ng/µl]	% Integrated Area	From [bp]	To [bp]	Peak Comment	Observations
100	8.50	8.50	-	66	147		Lower Marker
1257	0.334	-	6.46	1197	1412		
22595	2.91	-	56.15	5383	>60000		
-	-	-	-	-	-		Sample Well

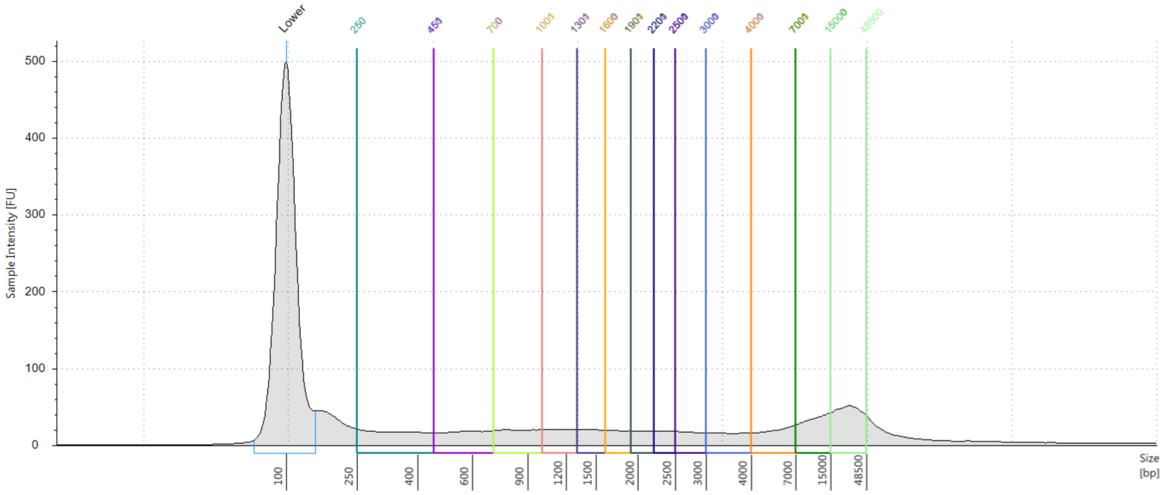

Region Table

From [bp]	To [bp]	Average Size [bp]	Conc. [ng/µl]	Region Molarity [nmol/l]	% of Total	Region Comment	Color
250	450	343	0.933	4.54	8.94		
451	700	574	0.743	2.13	7.12		
701	1000	850	0.690	1.32	6.61		
1001	1300	1156	0.516	0.720	4.94		
1301	1600	1459	0.419	0.462	4.01		

1601	1900	1759	0.358	0.328	3.43		
1901	2200	2065	0.317	0.247	3.03		
2201	2500	2363	0.297	0.203	2.85		
2501	3000	2762	0.375	0.220	3.59		
3001	4000	3509	0.500	0.233	4.79		
4001	7000	5592	0.638	0.189	6.11		
7001	15000	11092	0.884	0.133	8.46		
15001	48500	24805	1.24	0.0862	11.87		

© Agilent Technologies, Inc. 2016

20479
S. noctiflora 2018

E1: 4 CAT1

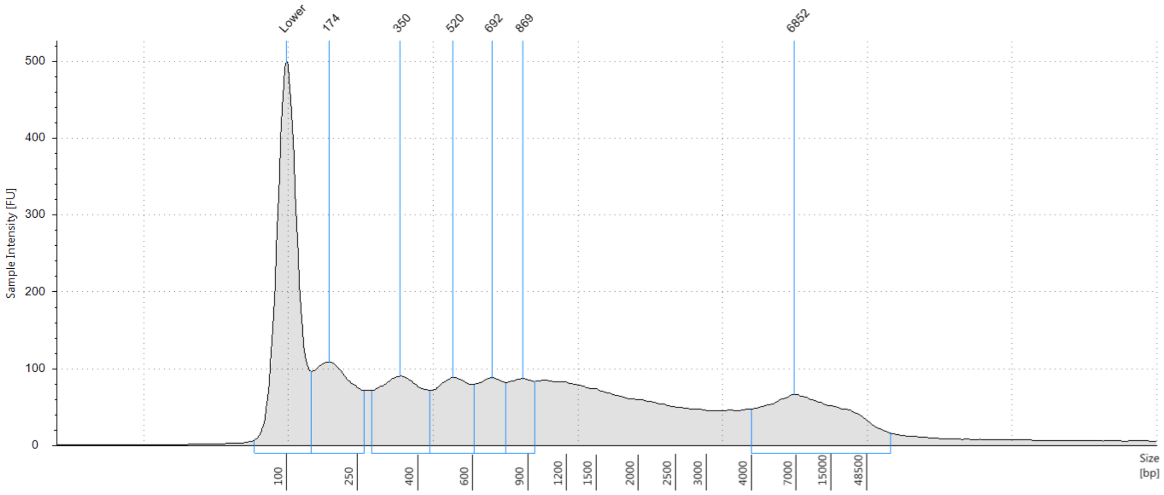

Sample Table

Well	DIN	Conc. [ng/µl]	Sample Description	Alert	Observations
E1	2.0	28.9	4 CAT1		

Peak Table

Size [bp]	Calibrated Conc. [ng/µl]	Assigned Conc. [ng/µl]	% Integrated Area	From [bp]	To [bp]	Peak Comment	Observations
100	8.50	8.50	-	66	138		Lower Marker
174	3.51	-	15.21	138	264		
350	3.29	-	14.24	280	439		
520	2.53	-	10.96	439	606		
692	1.95	-	8.43	606	768		
869	1.74	-	7.52	768	947		
6852	4.56	-	19.76	4028	>60000		
>60000	0.742	-	3.21	>60000	>60000		
-	-	-	-	-	-		Sample Well

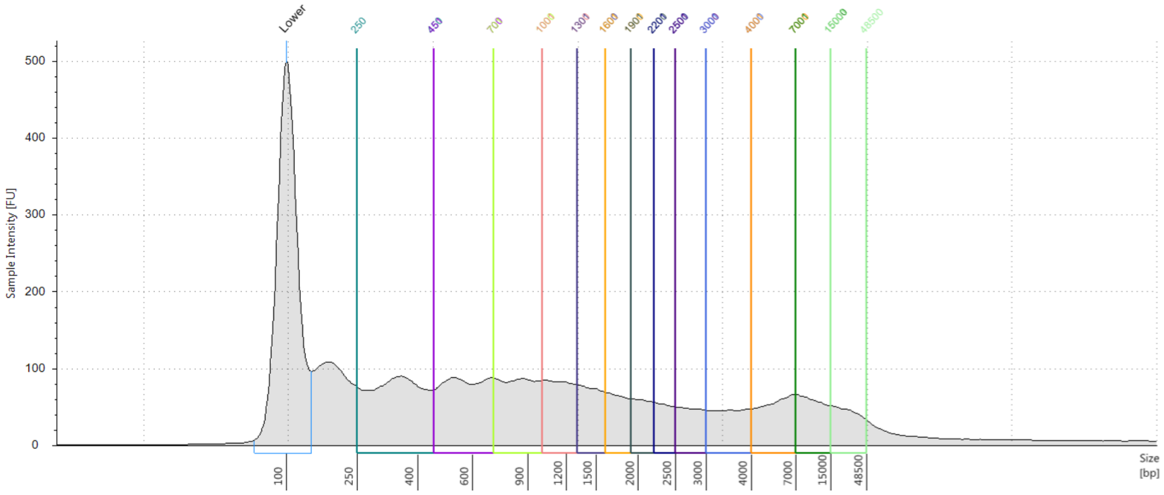

Region Table

From [bp]	To [bp]	Average Size [bp]	Conc. [ng/µl]	Region Molarity [nmol/l]	% of Total	Region Comment	Color
250	450	345	4.19	19.8	14.50		
451	700	573	3.54	9.94	12.27		

701	1000	849	2.86	5.39	9.92		
1001	1300	1150	2.05	2.84	7.11		
1301	1600	1456	1.51	1.66	5.24		
1601	1900	1757	1.20	1.09	4.16		
1901	2200	2067	0.981	0.758	3.40		
2201	2500	2358	0.788	0.535	2.73		
2501	3000	2762	0.999	0.583	3.46		
3001	4000	3504	1.43	0.663	4.96		
4001	7000	5553	1.78	0.527	6.16		
7001	15000	10696	1.45	0.229	5.01		
15001	48500	24069	1.09	0.0797	3.76		

 © Agilent Technologies, Inc. 2016

18108
S. acaulis 1994

F1: 5 CAT2

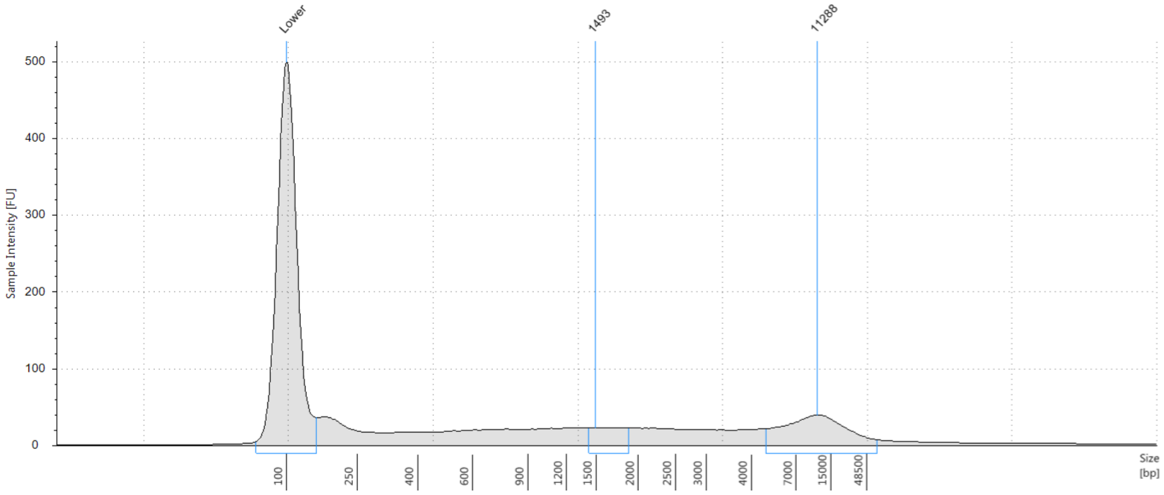

Sample Table

Well	DIN	Conc. [ng/µl]	Sample Description	Alert	Observations
F1	3.4	9.66	5 CAT2	⚠	Sample concentration outside recommended range

Peak Table

Size [bp]	Calibrated Conc. [ng/µl]	Assigned Conc. [ng/µl]	% Integrated Area	From [bp]	To [bp]	Peak Comment	Observations
100	8.50	8.50	-	67	147		Lower Marker
1493	0.679	-	20.59	1422	1875		
11288	2.08	-	62.98	4834	58109		
-	-	-	-	-	-		Sample Well

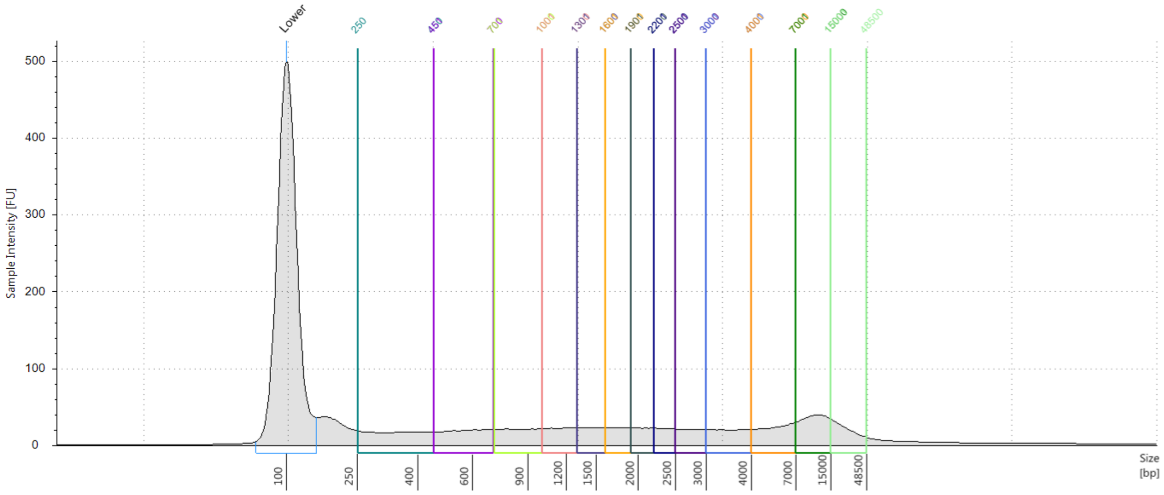

Region Table

From [bp]	To [bp]	Average Size [bp]	Conc. [ng/µl]	Region Molarity [nmol/l]	% of Total	Region Comment	Color
250	450	345	0.954	4.57	9.87		
451	700	574	0.827	2.33	8.56		
701	1000	851	0.736	1.39	7.62		
1001	1300	1154	0.575	0.796	5.96		

1301	1600	1458	0.486	0.530	5.03		
1601	1900	1758	0.445	0.402	4.60		
1901	2200	2065	0.393	0.302	4.07		
2201	2500	2363	0.361	0.242	3.73		
2501	3000	2767	0.477	0.274	4.94		
3001	4000	3515	0.671	0.306	6.94		
4001	7000	5584	0.798	0.233	8.26		
7001	15000	10989	0.954	0.144	9.87		
15001	48500	22579	0.542	0.0414	5.60		

 © Agilent Technologies, Inc. 2016

24702
S. burchellii 1987

G1: 6 CAT2

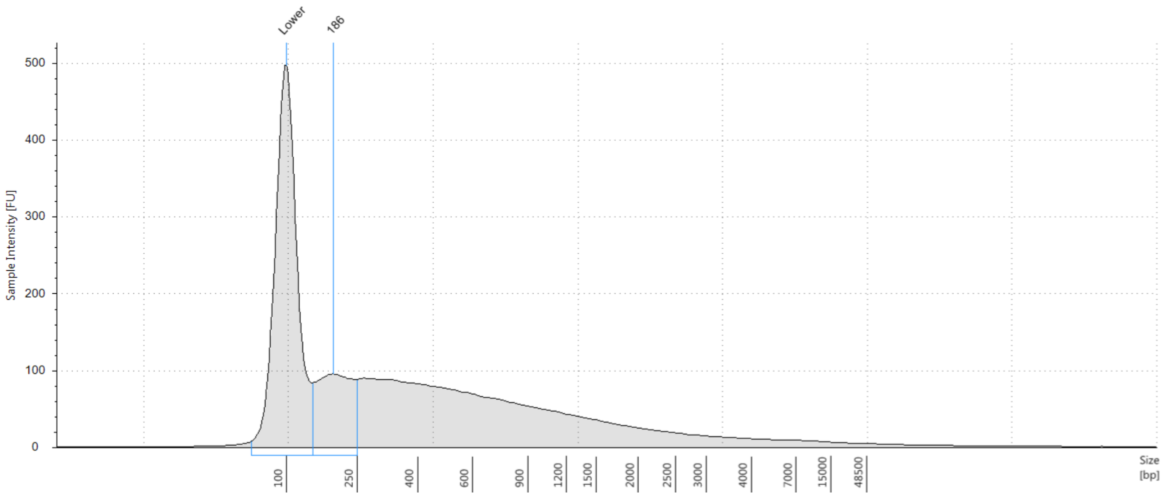

Sample Table

Well	DIN	Conc. [ng/µl]	Sample Description	Alert	Observations
G1	1.6	16.3	6 CAT2		

Peak Table

Size [bp]	Calibrated Conc. [ng/µl]	Assigned Conc. [ng/µl]	% Integrated Area	From [bp]	To [bp]	Peak Comment	Observations
100	8.50	8.50	-	64	141		Lower Marker
186	2.83	-	92.81	141	252		
-	-	-	-	-	-		Sample Well

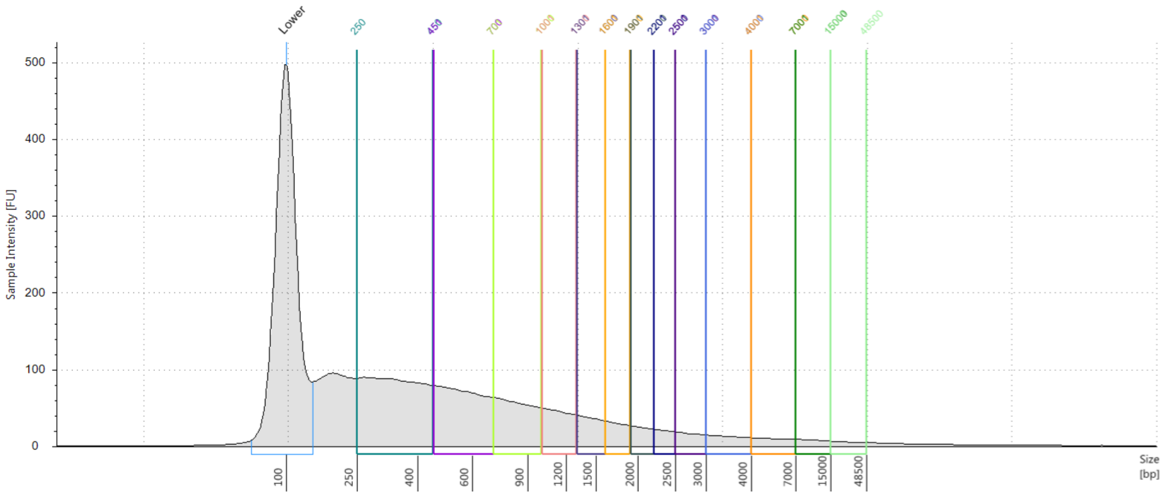

Region Table

From [bp]	To [bp]	Average Size [bp]	Conc. [ng/µl]	Region Molarity [nmol/l]	% of Total	Region Comment	Color
250	450	342	4.43	20.8	27.19		
451	700	569	2.92	8.16	17.93		
701	1000	843	1.85	3.48	11.38		
1001	1300	1150	1.12	1.54	6.88		
1301	1600	1459	0.710	0.770	4.36		

1601	1900	1754	0.521	0.472	3.20		
1901	2200	2062	0.405	0.313	2.48		
2201	2500	2363	0.301	0.204	1.85		
2501	3000	2752	0.343	0.202	2.11		
3001	4000	3476	0.380	0.180	2.33		
4001	7000	5386	0.291	0.0928	1.79		
7001	15000	10552	0.177	0.0301	1.09		
15001	48500	24266	0.124	0.00997	0.76		

 © Agilent Technologies, Inc. 2016

24712
S. noctiflora 1981

H1: 7 CAT2

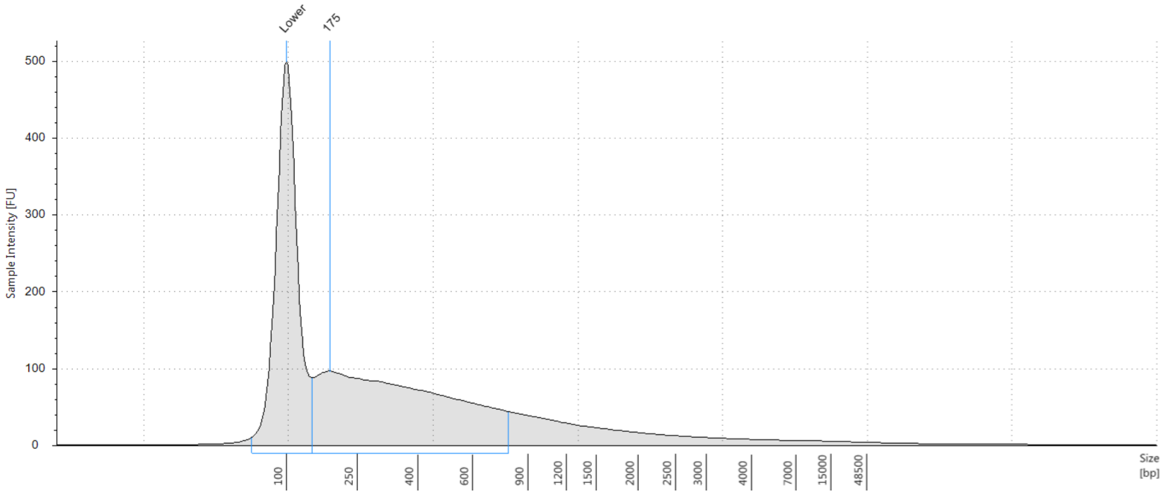

Sample Table

Well	DIN	Conc. [ng/µl]	Sample Description	Alert	Observations
H1	1.4	13.6	7 CAT2		

Peak Table

Size [bp]	Calibrated Conc. [ng/µl]	Assigned Conc. [ng/µl]	% Integrated Area	From [bp]	To [bp]	Peak Comment	Observations
100	8.50	8.50	-	64	139		Lower Marker
175	9.75	-	96.04	139	781		
-	-	-	-	-	-		Sample Well

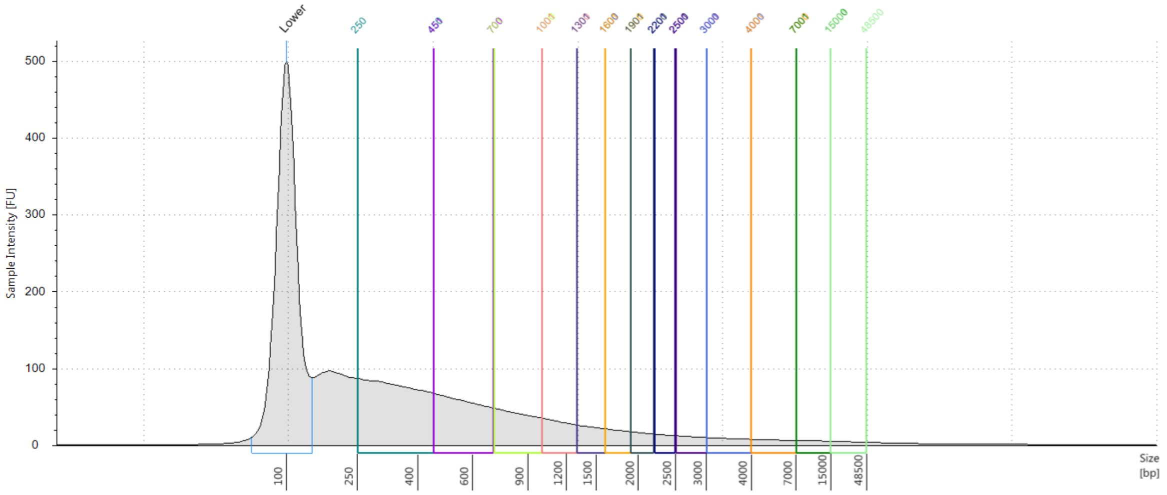

Region Table

From [bp]	To [bp]	Average Size [bp]	Conc. [ng/µl]	Region Molarity [nmol/l]	% of Total	Region Comment	Color
250	450	341	4.13	19.4	30.26		
451	700	564	2.36	6.65	17.31		
701	1000	842	1.35	2.54	9.89		
1001	1300	1143	0.748	1.04	5.48		
1301	1600	1452	0.470	0.515	3.45		

 © Agilent Technologies, Inc. 2016

1601	1900	1759	0.328	0.297	2.40		
1901	2200	2062	0.269	0.209	1.97		
2201	2500	2369	0.200	0.136	1.46		
2501	3000	2756	0.224	0.133	1.64		
3001	4000	3495	0.257	0.122	1.88		
4001	7000	5398	0.197	0.0635	1.45		
7001	15000	10637	0.129	0.0221	0.94		
15001	48500	24703	0.0988	0.00788	0.72		

© Agilent Technologies, Inc. 2016

17365
S. involucrata ssp furcata 1979

A2: 8 CAT2

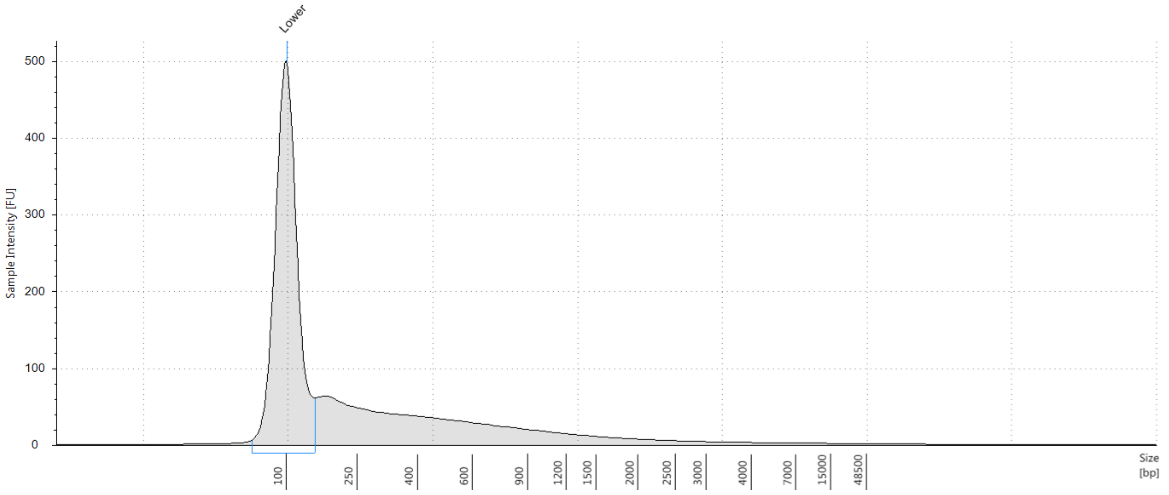

Sample Table

Well	DIN	Conc. [ng/µl]	Sample Description	Alert	Observations
A2	1.2	6.91	8 CAT2	⚠	Sample concentration outside recommended range

Peak Table

Size [bp]	Calibrated Conc. [ng/µl]	Assigned Conc. [ng/µl]	% Integrated Area	From [bp]	To [bp]	Peak Comment	Observations
100	8.50	8.50	-	64	144		Lower Marker
-	-	-	-	-	-		Sample Well

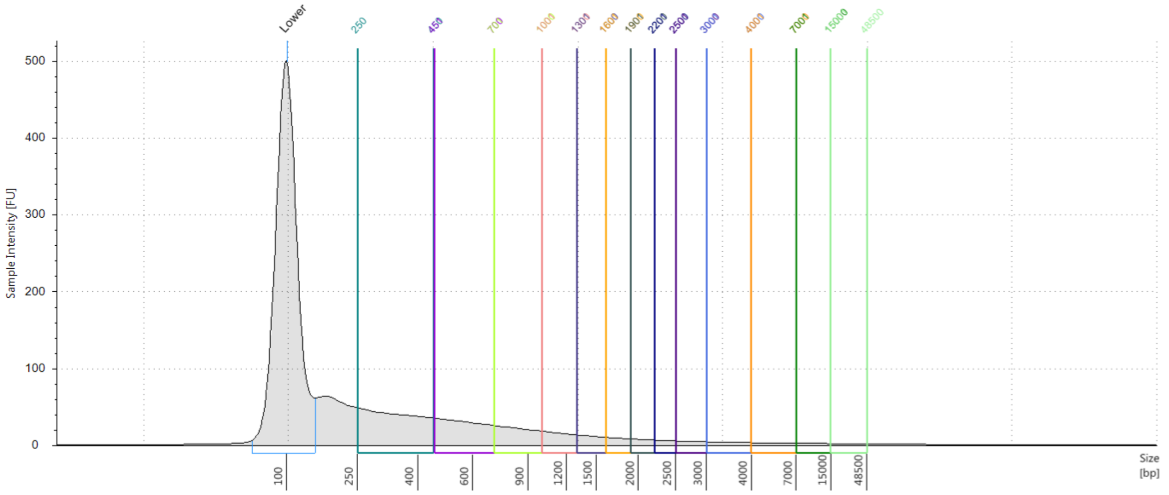

Region Table

From [bp]	To [bp]	Average Size [bp]	Conc. [ng/µl]	Region Molarity [nmol/l]	% of Total	Region Comment	Color
250	450	339	2.09	9.95	30.19		
451	700	564	1.23	3.50	17.82		
701	1000	839	0.705	1.35	10.19		
1001	1300	1143	0.366	0.517	5.30		
1301	1600	1456	0.230	0.257	3.32		
1601	1900	1756	0.146	0.137	2.12		

 © Agilent Technologies, Inc. 2016

1901	2200	2067	0.109	0.0881	1.57		
2201	2500	2362	0.0835	0.0602	1.21		
2501	3000	2756	0.0934	0.0593	1.35		
3001	4000	3478	0.0993	0.0521	1.44		
4001	7000	5364	0.0745	0.0274	1.08		
7001	15000	10684	0.0445	0.00892	0.64		
15001	48500	24942	0.0347	0.00336	0.50		

18313
S. acaulis 1969

B2: 9 CAT3

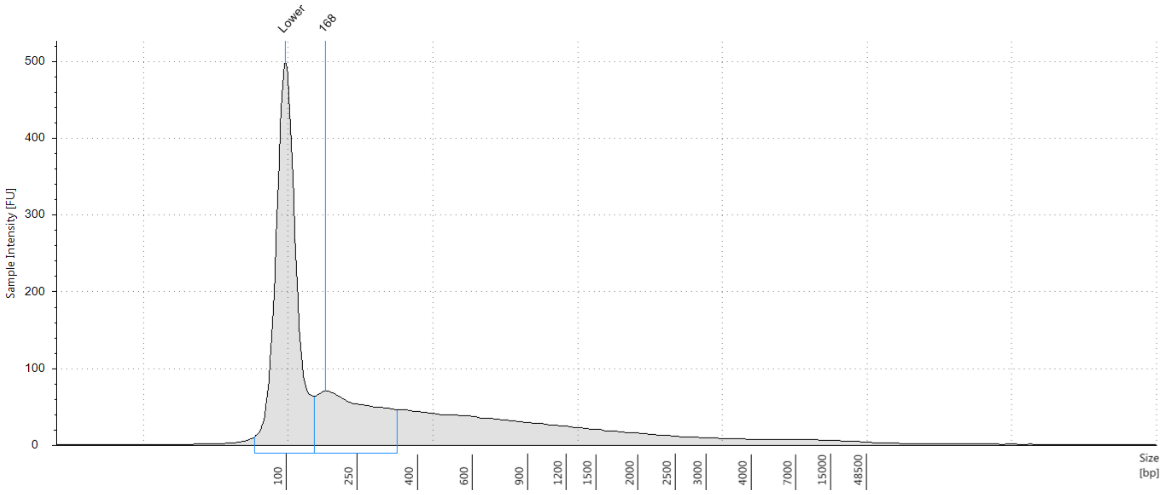

Sample Table

Well	DIN	Conc. [ng/µl]	Sample Description	Alert	Observations
B2	1.6	10.2	9 CAT3		

Peak Table

Size [bp]	Calibrated Conc. [ng/µl]	Assigned Conc. [ng/µl]	% Integrated Area	From [bp]	To [bp]	Peak Comment	Observations
100	8.50	8.50	-	67	145		Lower Marker
168	3.46	-	97.28	145	343		
-	-	-	-	-	-		Sample Well

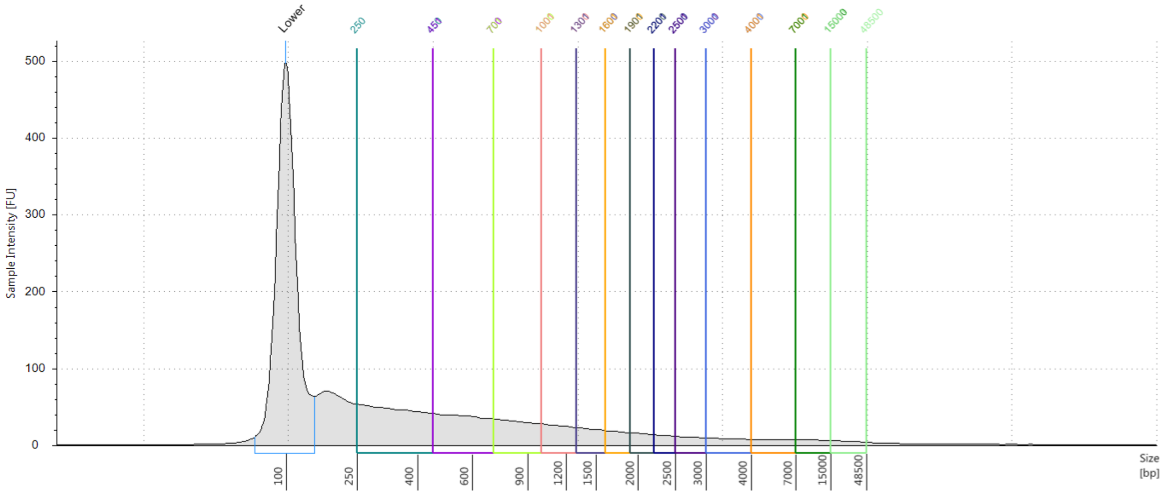

Region Table

From [bp]	To [bp]	Average Size [bp]	Conc. [ng/µl]	Region Molarity [nmol/l]	% of Total	Region Comment	Color
250	450	340	2.64	12.6	25.93		
451	700	568	1.69	4.79	16.65		
701	1000	846	1.08	2.06	10.65		
1001	1300	1150	0.653	0.913	6.42		
1301	1600	1458	0.441	0.488	4.33		

1601	1900	1761	0.333	0.307	3.27		
1901	2200	2069	0.257	0.203	2.52		
2201	2500	2364	0.192	0.134	1.88		
2501	3000	2755	0.230	0.140	2.26		
3001	4000	3493	0.264	0.130	2.59		
4001	7000	5411	0.208	0.0692	2.04		
7001	15000	10602	0.156	0.0274	1.53		
15001	48500	24168	0.119	0.0100	1.17		

C2: 10 CAT3

20329
S. burchellii
1948

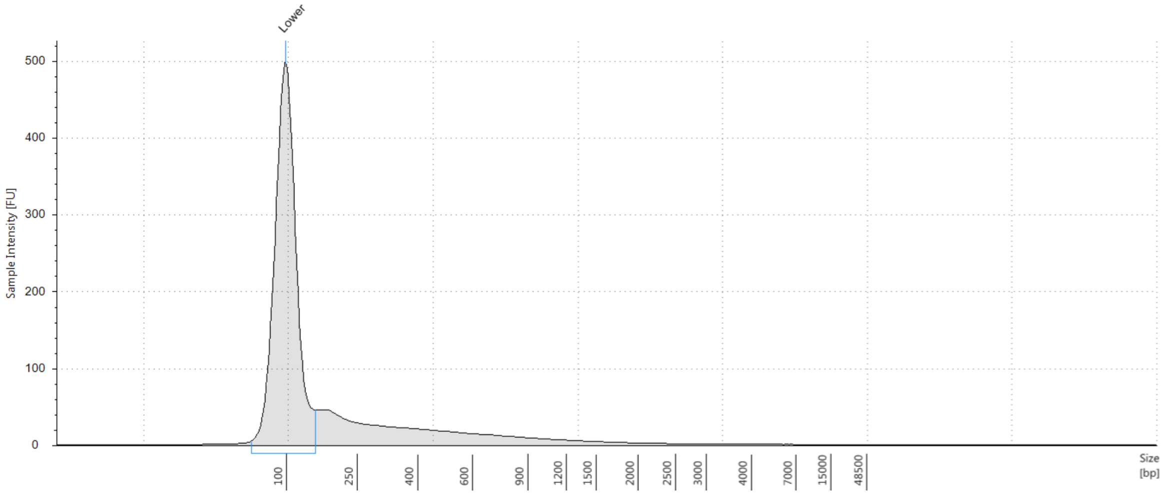

Sample Table

Well	DIN	Conc. [ng/µl]	Sample Description	Alert	Observations
C2	1.0	3.59	10 CAT3	⚠	Sample concentration outside functional range for DIN

Peak Table

Size [bp]	Calibrated Conc. [ng/µl]	Assigned Conc. [ng/µl]	% Integrated Area	From [bp]	To [bp]	Peak Comment	Observations
100	8.50	8.50	-	64	147		Lower Marker
-	-	-	-	-	-		Sample Well

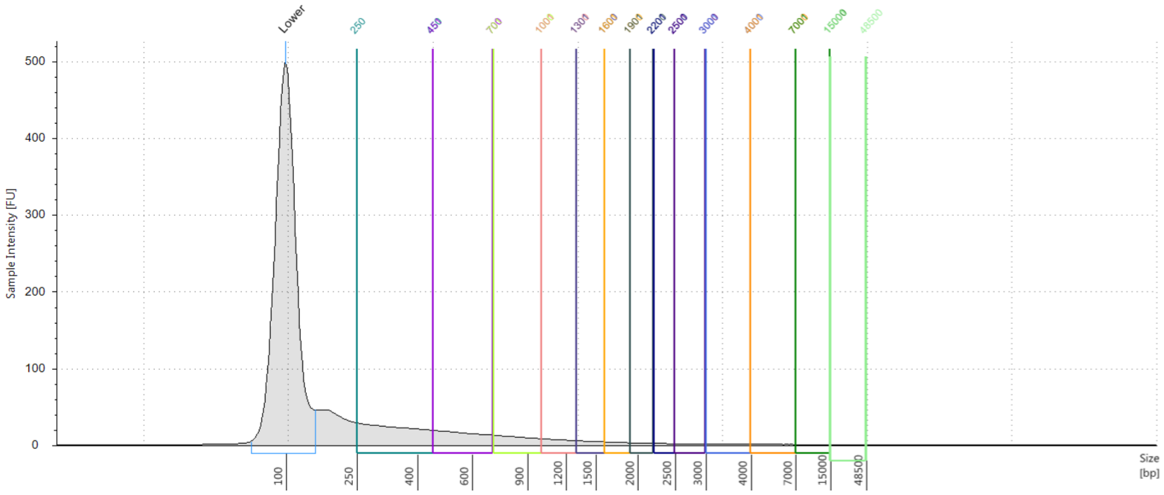

Region Table

From [bp]	To [bp]	Average Size [bp]	Conc. [ng/µl]	Region Molarity [nmol/l]	% of Total	Region Comment	Color
250	450	338	1.22	6.05	34.01		
451	700	563	0.644	1.94	17.92		
701	1000	838	0.332	0.694	9.25		
1001	1300	1145	0.153	0.247	4.27		
1301	1600	1452	0.0813	0.112	2.26		

© Agilent Technologies, Inc. 2016

1601	1900	1749	0.0502	0.0630	1.40		
1901	2200	2063	0.0309	0.0380	0.86		
2201	2500	2367	0.0183	0.0235	0.51		
2501	3000	2763	0.0154	0.0228	0.43		
3001	4000	3493	0.00855	0.0199	0.24		
4001	7000	5442	0.00140	0.0109	0.04		
7001	15000	10690	0.000979	0.00370	0.03		
15001	48500	24266	0.000861	0.00155	0.02		

 © Agilent Technologies, Inc. 2016

17368
S. uralensis
ssp uralensis
1959

D2: 11 CAT3

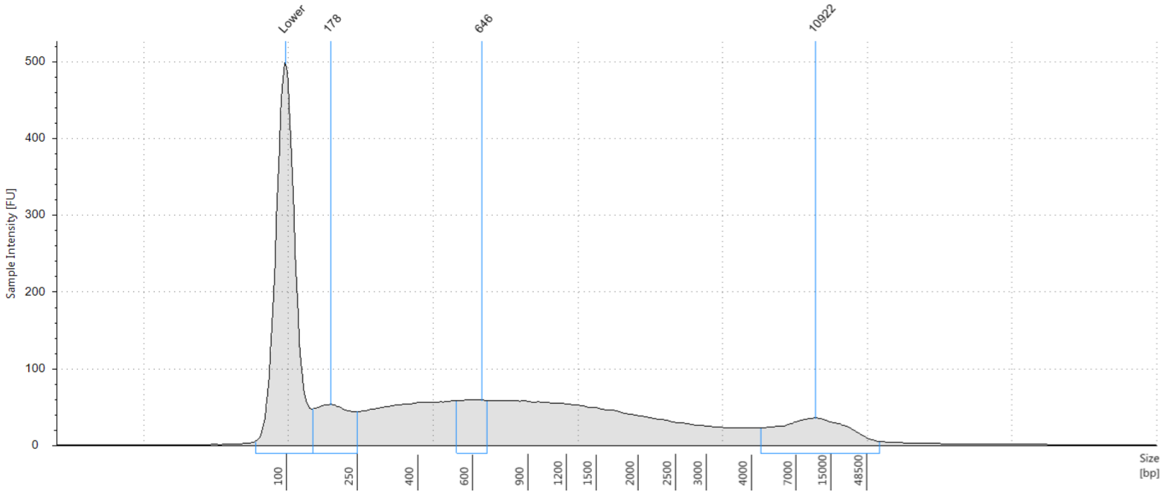

Sample Table

Well	DIN	Conc. [ng/µl]	Sample Description	Alert	Observations
D2	1.9	17.5	11 CAT3		

Peak Table

Size [bp]	Calibrated Conc. [ng/µl]	Assigned Conc. [ng/µl]	% Integrated Area	From [bp]	To [bp]	Peak Comment	Observations
100	8.50	8.50	-	67	142		Lower Marker
178	1.67	-	30.80	142	251		
646	1.40	-	25.80	536	670		
10922	2.15	-	39.63	4553	>60000		
-	-	-	-	-	-		Sample Well

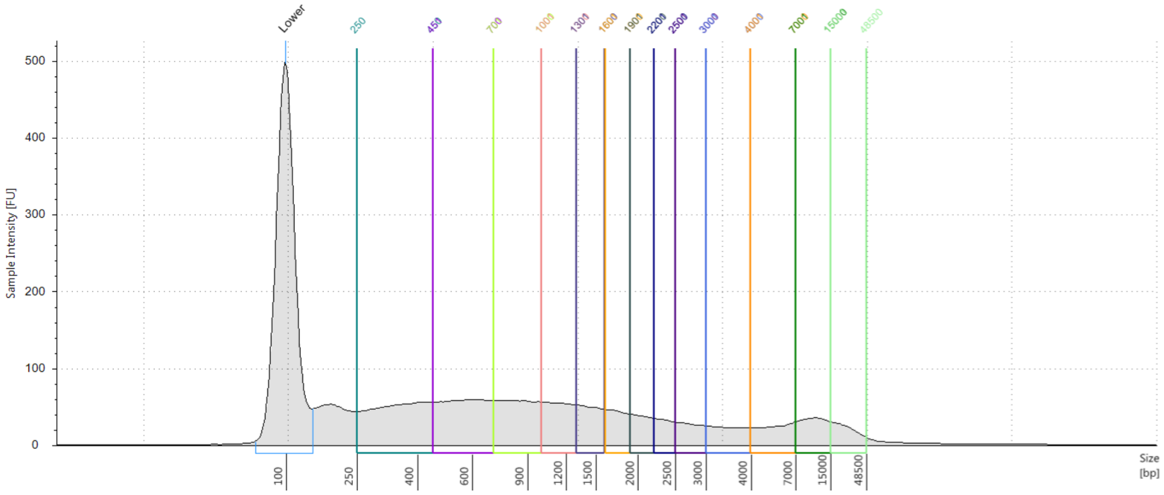

Region Table

From [bp]	To [bp]	Average Size [bp]	Conc. [ng/µl]	Region Molarity [nmol/l]	% of Total	Region Comment	Color
250	450	349	2.97	13.8	16.96		
451	700	572	2.65	7.42	15.15		
701	1000	847	2.14	4.03	12.23		
1001	1300	1155	1.50	2.07	8.60		

 © Agilent Technologies, Inc. 2016

1301	1600	1463	1.08	1.17	6.17		
1601	1900	1760	0.871	0.786	4.98		
1901	2200	2065	0.678	0.523	3.88		
2201	2500	2361	0.554	0.376	3.17		
2501	3000	2756	0.642	0.376	3.67		
3001	4000	3490	0.775	0.363	4.43		
4001	7000	5536	0.845	0.255	4.83		
7001	15000	10852	0.880	0.137	5.03		
15001	48500	22889	0.589	0.0456	3.37		

© Agilent Technologies, Inc. 2016

23558
S. rigens 1932

E2: 12 CAT3

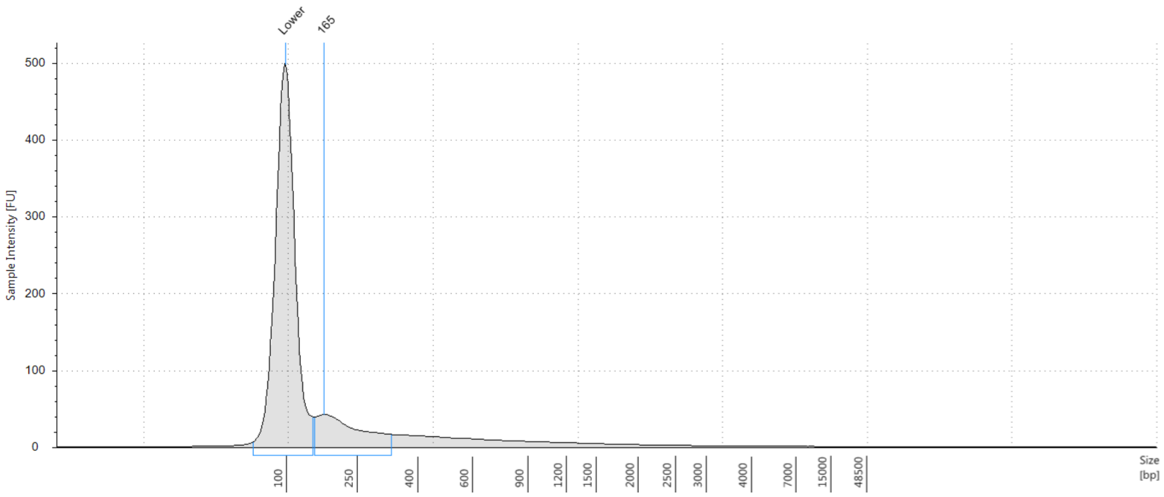

Sample Table

Well	DIN	Conc. [ng/µl]	Sample Description	Alert	Observations
E2	1.0	3.46	12 CAT3	⚠	Sample concentration outside functional range for DIN

Peak Table

Size [bp]	Calibrated Conc. [ng/µl]	Assigned Conc. [ng/µl]	% Integrated Area	From [bp]	To [bp]	Peak Comment	Observations
100	8.50	8.50	-	66	143		Lower Marker
165	1.61	-	100.00	145	329		

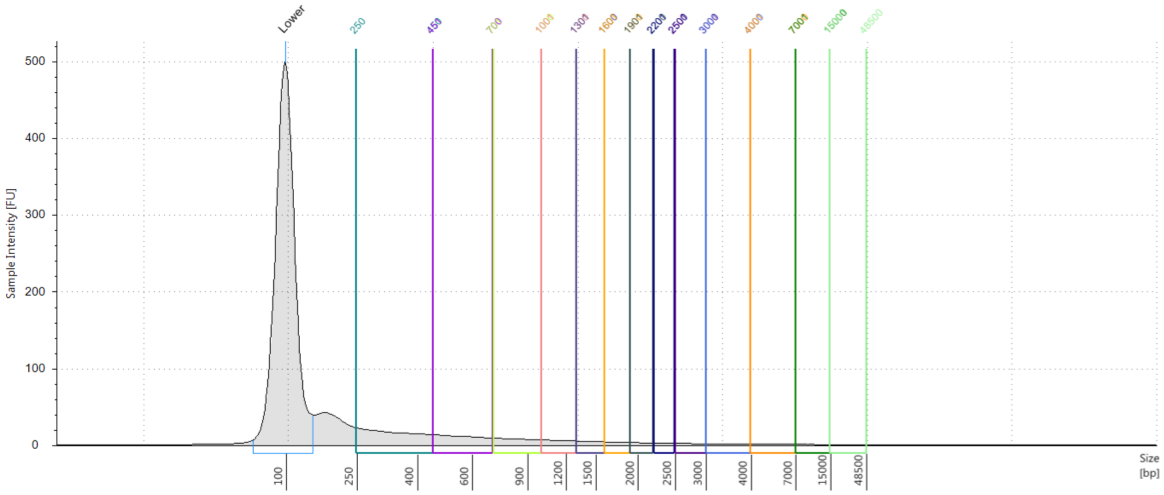

Region Table

From [bp]	To [bp]	Average Size [bp]	Conc. [ng/µl]	Region Molarity [nmol/l]	% of Total	Region Comment	Color
250	450	337	0.975	4.78	28.22		
451	700	565	0.500	1.47	14.47		
701	1000	842	0.284	0.566	8.21		
1001	1300	1150	0.154	0.229	4.46		
1301	1600	1459	0.0994	0.119	2.88		

© Agilent Technologies, Inc. 2016

1601	1900	1757	0.0678	0.0695	1.96		
1901	2200	2061	0.0505	0.0458	1.46		
2201	2500	2362	0.0363	0.0299	1.05		
2501	3000	2754	0.0412	0.0309	1.19		
3001	4000	3487	0.0399	0.0267	1.15		
4001	7000	5388	0.0263	0.0136	0.76		
7001	15000	10731	0.0146	0.00459	0.42		
15001	48500	24890	0.00932	0.00174	0.27		

© Agilent Technologies, Inc. 2016

F2: Ladder

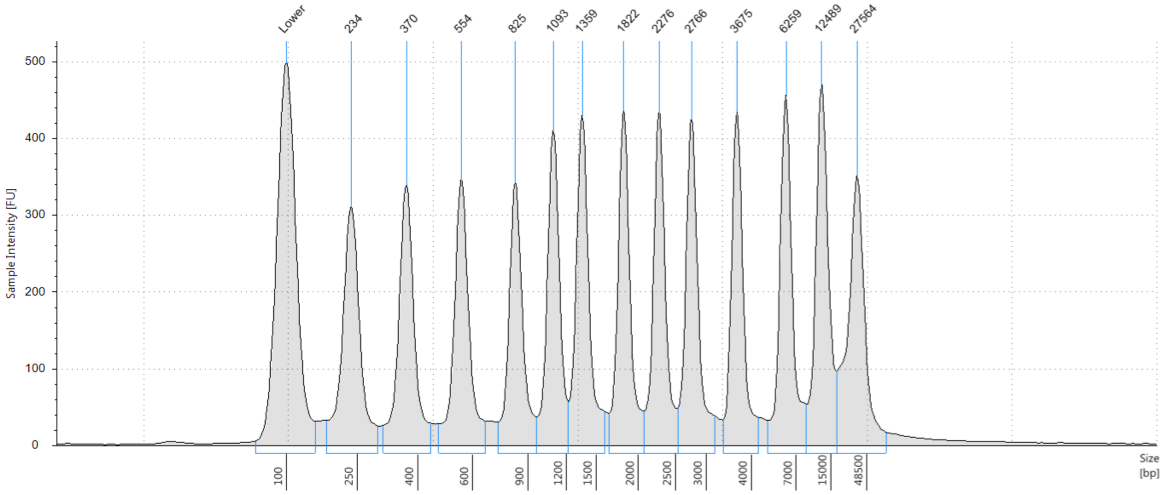

Sample Table

Well	DIN	Conc. [ng/µl]	Sample Description	Alert	Observations
F2	-	62.0	Ladder		Ladder run as sample

Peak Table

Size [bp]	Calibrated Conc. [ng/µl]	Assigned Conc. [ng/µl]	% Integrated Area	From [bp]	To [bp]	Peak Comment	Observations
100	8.50	8.50	-	68	146		Lower Marker
234	4.63	-	7.60	169	295		
370	4.50	-	7.39	307	444		
554	4.43	-	7.27	469	661		
825	4.08	-	6.69	725	967		
1093	4.46	-	7.33	967	1224		
1359	4.87	-	8.00	1224	1602		
1822	4.67	-	7.66	1644	2078		
2276	4.64	-	7.61	2078	2548		
2766	4.63	-	7.60	2548	3178		
3675	4.52	-	7.41	3363	4430		
6259	4.94	-	8.10	4945	8954		
12489	5.34	-	8.77	8954	17420		
27564	5.18	-	8.50	17420	>60000		
-	-	-	-	-	-		Sample Well

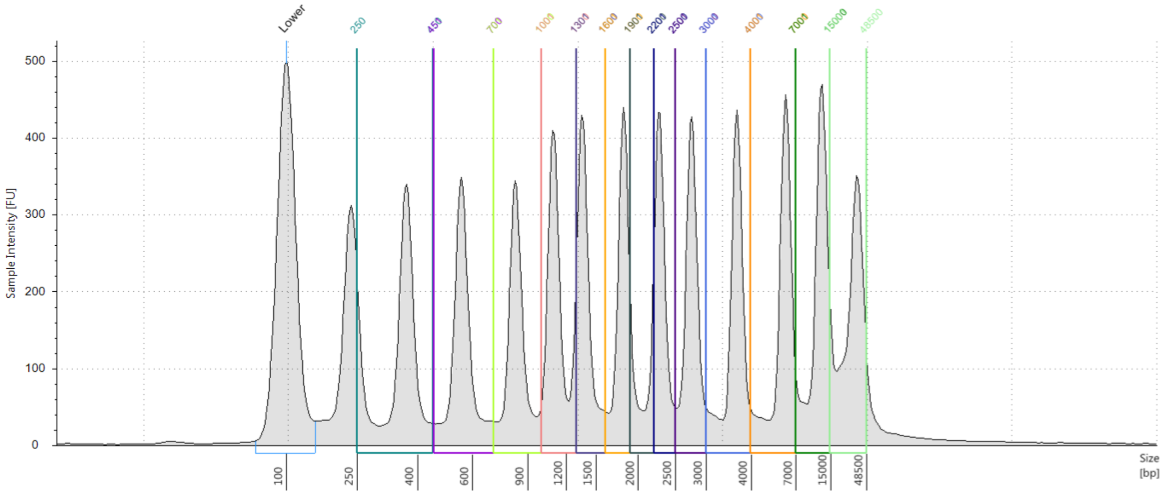

Region Table

From [bp]	To [bp]	Average Size [bp]	Conc. [ng/µl]	Region Molarity [nmol/l]	% of Total	Region Comment	Color
250	450	350	5.92	28.0	9.55		
451	700	563	4.71	13.4	7.60		
701	1000	839	4.31	8.20	6.95		
1001	1300	1135	5.17	7.19	8.34		
1301	1600	1400	4.22	4.75	6.80		
1601	1900	1806	4.04	3.51	6.52		
1901	2200	2075	1.72	1.35	2.78		
2201	2500	2314	3.97	2.69	6.41		
2501	3000	2769	4.46	2.53	7.19		
3001	4000	3636	4.80	2.09	7.74		
4001	7000	6067	4.94	1.31	7.97		
7001	15000	12171	5.26	0.703	8.49		
15001	48500	27043	5.31	0.340	8.56		

 © Agilent Technologies, Inc. 2016

Calibration

Molecular Weight Settings

Fitting type: Genomic DNA Sizing
Alignment type: From lower marker

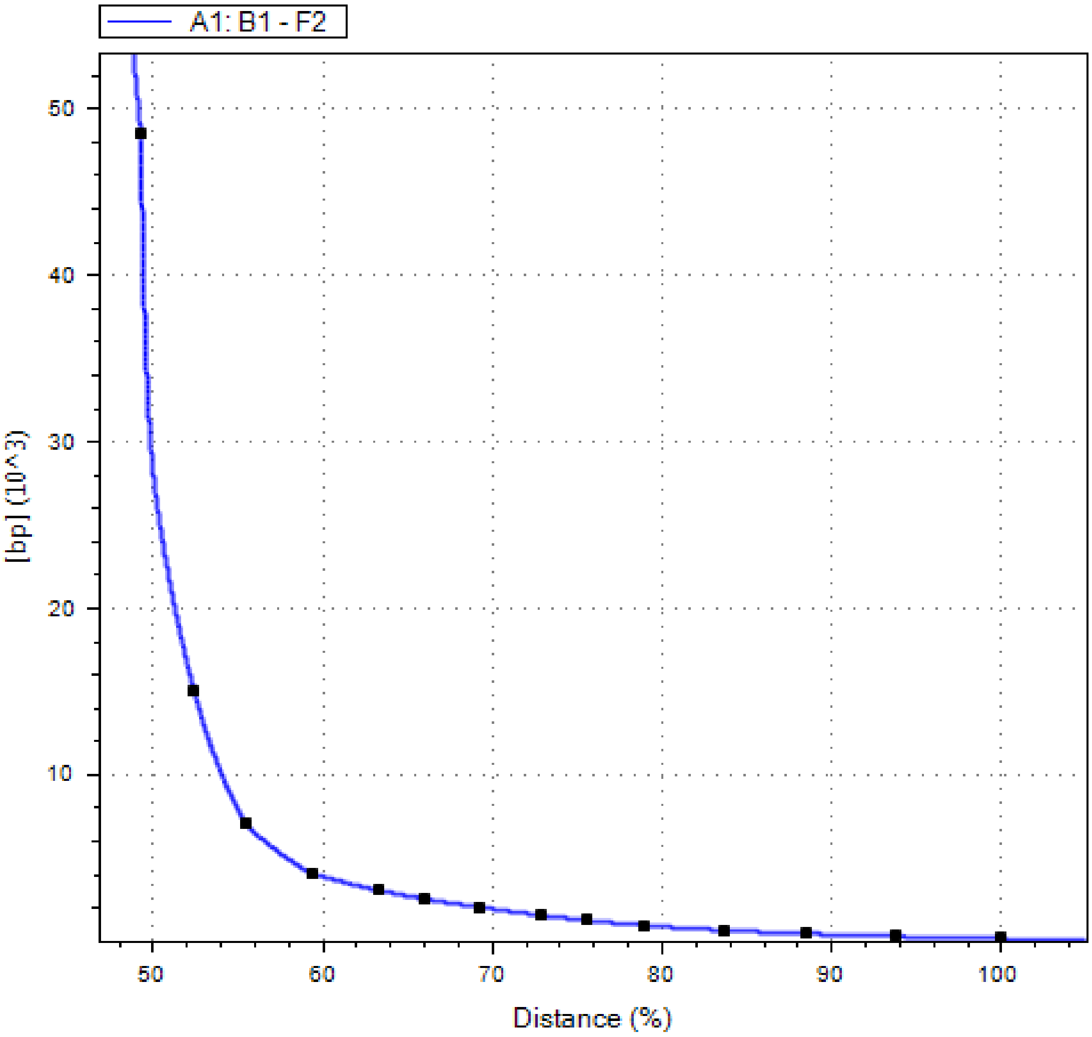

Concentration Settings

Calibration mode: Lower Marker
Normalise peaks from: Lower Marker
Fitting type: Linear Regression

Experiment Properties

Run Properties

**Analysis Software Version**	2.1.38.8716
**Filename**	C:\Users\admin\Desktop\AnneSophie\2019-06-27-01 after CTAB extraction.gDNA
**Assay**	Genomic DNA
**Run End Date**	27-Jun-2019 4:30 PM
**Last Saved Under Version**	2.1.38.8716
**DIN Version**	2.1.38.8716
**Study**	
**Comments**	

ScreenTape Device 1

**Username**	admin
**ScreenTape Device ID**	01-S025-190227-01-001692
**Expiry Date**	11-Jul-2019
**ScreenTape Device History**	First run 27-Jun-2019, 1 run performed
**Temperature [°C]**	23.5
**Electrophoresis Time [s]**	225
**Instrument Type**	6655
**Instrument Serial Number**	03-PM405
**Notes**	
**ScreenTape Device Run Date**	27-Jun-2019 4:08 PM

Controller Environment

**Computer**	LAB3210150
**Instrument Controller Software Version**	A.02.01 SR1
**First Run Analysis Version**	2.1.38.8716
**Operating System**	Microsoft Windows 7 Enterprise
