# Supplementary material for: Retrieval of long DNA reads from herbarium specimens
Source: AoB Plants. 2023 Nov 8;15(6):plad074. doi: 10.1093/aobpla/plad074 (PMC10735254; doi:10.1093/aobpla/plad074)
Supplement: plad074_suppl_Supplementary_Appendix_S1_2 [file plad074_suppl_supplementary_appendix_s1_2.pdf]

Filename: 2019-09-06-01_after_first_size_selection.gDNA

Gel Image

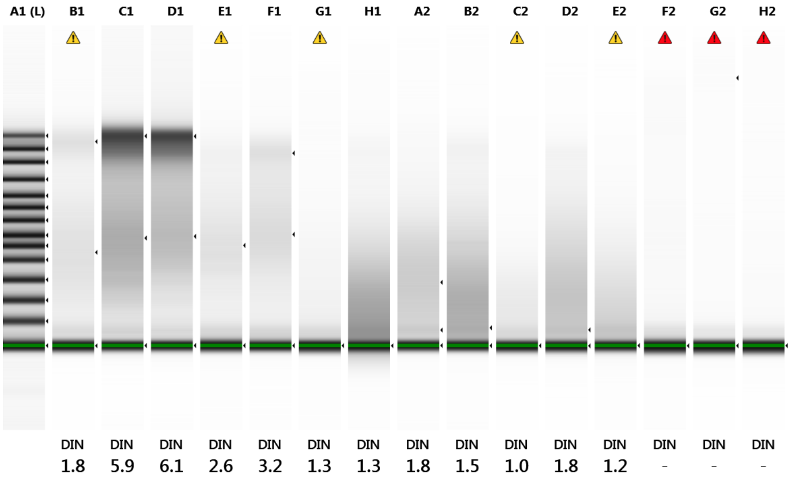

Default image (Contrast 100%)

Sample Info

Well	DIN	Conc. [ng/µl]	Sample Description	Alert	Observations
A1	-	63.3	Ladder		Ladder
B1	1.8	9.59	cat1: 1	⚠	Sample concentration outside recommended range
C1	5.9	41.4	cat1: 2		
D1	6.1	36.1	cat1: 3		
E1	2.6	7.27	cat1: 4	⚠	Sample concentration outside recommended range
F1	3.2	10.5	cat2: 1		
G1	1.3	3.10	cat2: 2	⚠	Sample concentration outside functional range for DIN
H1	1.3	15.3	cat2: 3		
A2	1.8	13.0	cat2: 4		
B2	1.5	17.5	cat3: 1		
C2	1.0	3.55	cat3: 2	⚠	Sample concentration outside functional range for DIN
D2	1.8	13.1	cat3: 3		
E2	1.2	7.48	cat3: 4	⚠	Sample concentration outside recommended range
F2	-	2.41	J1	⚠	Sample concentration outside functional range for DIN
G2	-	1.57	J2	⚠	Sample concentration outside functional range for DIN
H2	-	1.47	J3	⚠	Sample concentration outside functional range for DIN

 © Agilent Technologies, Inc. 2016

A1: Ladder

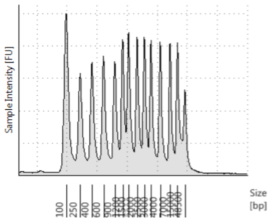

B1: cat1: 1

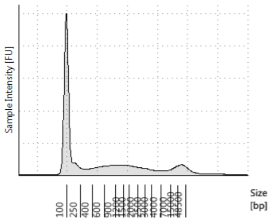

C1: cat1: 2

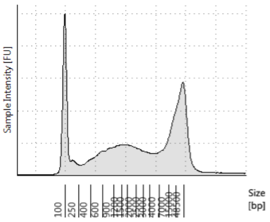

D1: cat1: 3

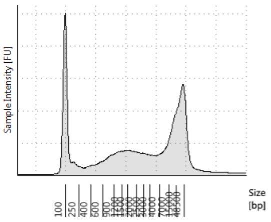

E1: cat1: 4

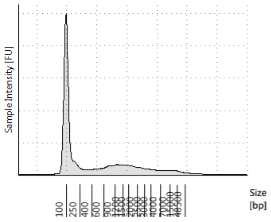

F1: cat2: 1

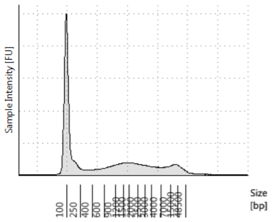

G1: cat2: 2

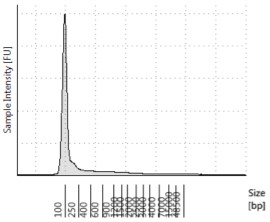

H1: cat2: 3

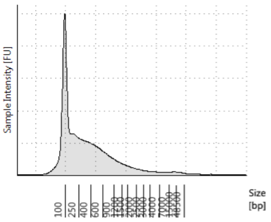

A2: cat2: 4

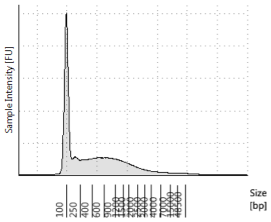

B2: cat3: 1

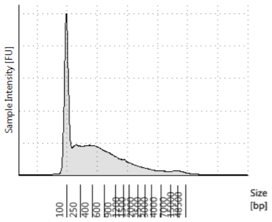

C2: cat3: 2

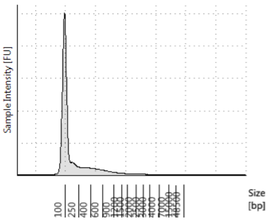

D2: cat3: 3

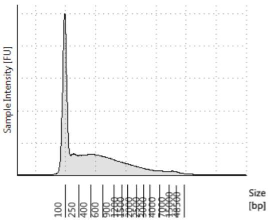

E2: cat3: 4

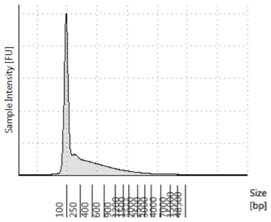

F2: J1

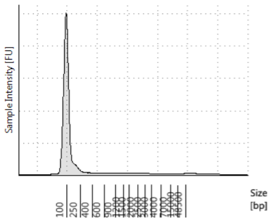

G2: J2

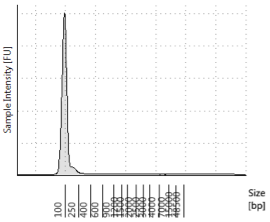

H2: J3

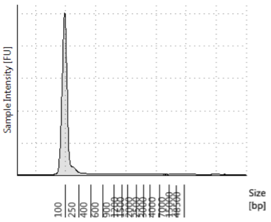

 © Agilent Technologies, Inc. 2016

A1: Ladder

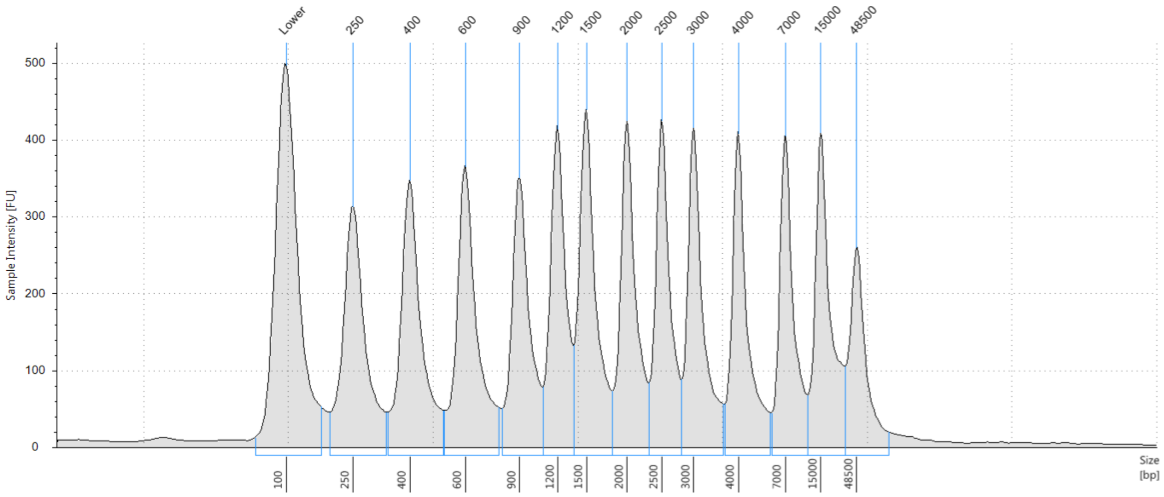

Sample Table

Well	DIN	Conc. [ng/µl]	Sample Description	Alert	Observations
A1	-	63.3	Ladder		Ladder

Peak Table

Size [bp]	Calibrated Conc. [ng/µl]	Assigned Conc. [ng/µl]	% Integrated Area	From [bp]	To [bp]	Peak Comment	Observations
100	8.50	8.50	-	65	161		Lower Marker
250	5.10	-	8.26	181	329		
400	5.24	-	8.48	333	510		
600	5.32	-	8.62	515	769		
900	4.63	-	7.50	791	1074		
1200	4.86	-	7.87	1074	1361		
1500	5.50	-	8.91	1361	1801		
2000	4.94	-	8.00	1801	2308		
2500	4.63	-	7.50	2308	2797		
3000	4.80	-	7.77	2797	3634		
4000	4.61	-	7.46	3663	5866		
7000	4.23	-	6.86	5953	11234		
15000	4.78	-	7.73	11234	25520		
48500	3.12	-	5.04	25520	>60000		

 © Agilent Technologies, Inc. 2016

19123
S. sachalinensis 2017

B1: cat1: 1

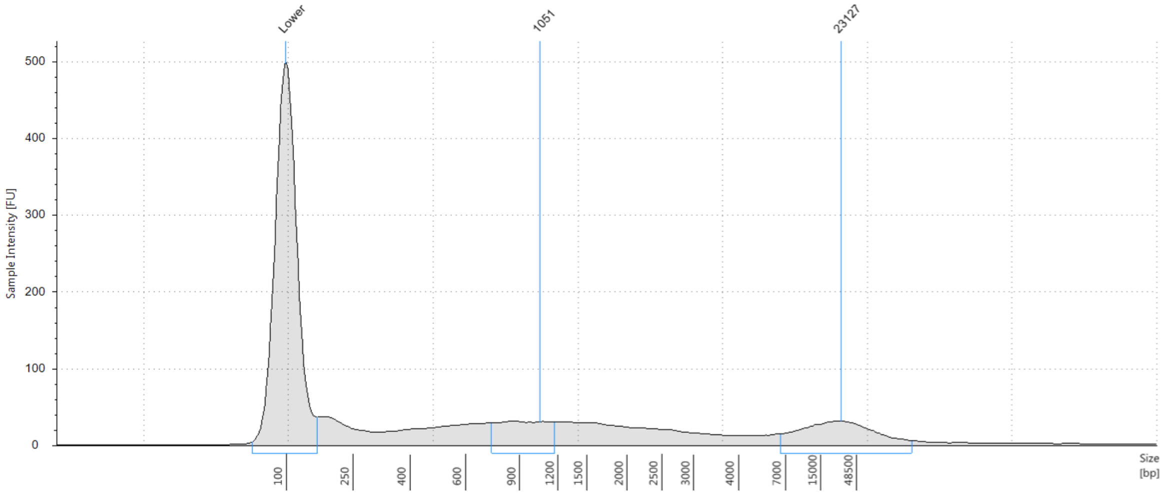

Sample Table

Well	DIN	Conc. [ng/µl]	Sample Description	Alert	Observations
B1	1.8	9.59	cat1: 1	⚠	Sample concentration outside recommended range

Peak Table

Size [bp]	Calibrated Conc. [ng/µl]	Assigned Conc. [ng/µl]	% Integrated Area	From [bp]	To [bp]	Peak Comment	Observations
100	8.50	8.50	-	63	153		Lower Marker
1051	1.29	-	28.32	732	1175		
23127	1.80	-	39.47	6628	>60000		
>60000	0.154	-	3.38	>60000	>60000		
-	-	-	-	-	-		Sample Well

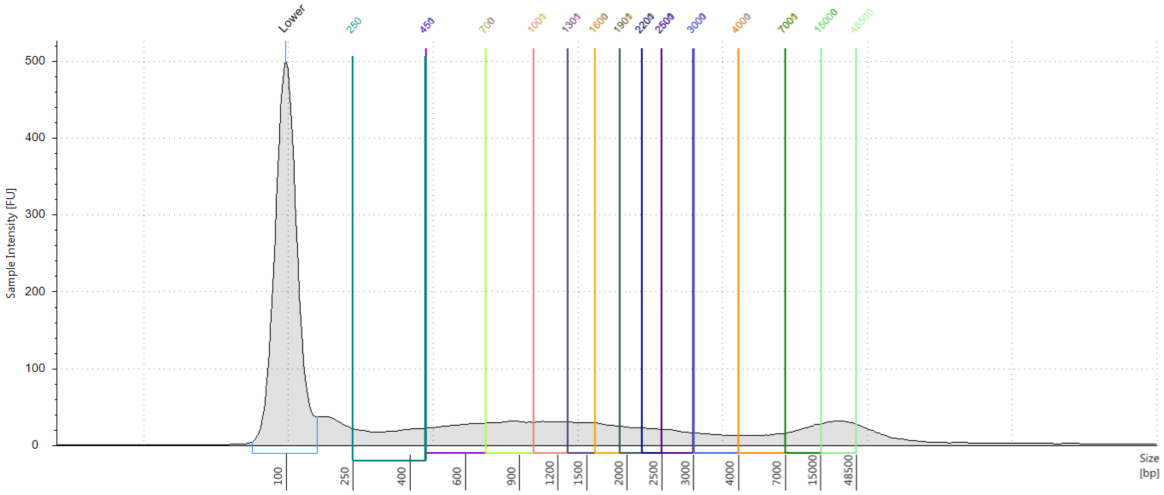

Region Table

From [bp]	To [bp]	Average Size [bp]	Conc. [ng/µl]	Region Molarity [nmol/l]	% of Total	Region Comment	Color
250	450	348	0.941	4.42	9.81		
451	700	576	1.06	2.93	11.02		
701	1000	850	0.959	1.79	10.01		
1001	1300	1151	0.720	0.986	7.51		

 © Agilent Technologies, Inc. 2016

1301	1600	1460	0.552	0.596	5.76		
1601	1900	1758	0.453	0.406	4.73		
1901	2200	2070	0.360	0.274	3.76		
2201	2500	2372	0.297	0.198	3.10		
2501	3000	2750	0.390	0.225	4.06		
3001	4000	3474	0.418	0.193	4.36		
4001	7000	5474	0.422	0.127	4.40		
7001	15000	11133	0.529	0.0792	5.52		
15001	48500	24645	0.735	0.0515	7.67		

 © Agilent Technologies, Inc. 2016

25463
S. acaulis 2019

C1: cat1: 2

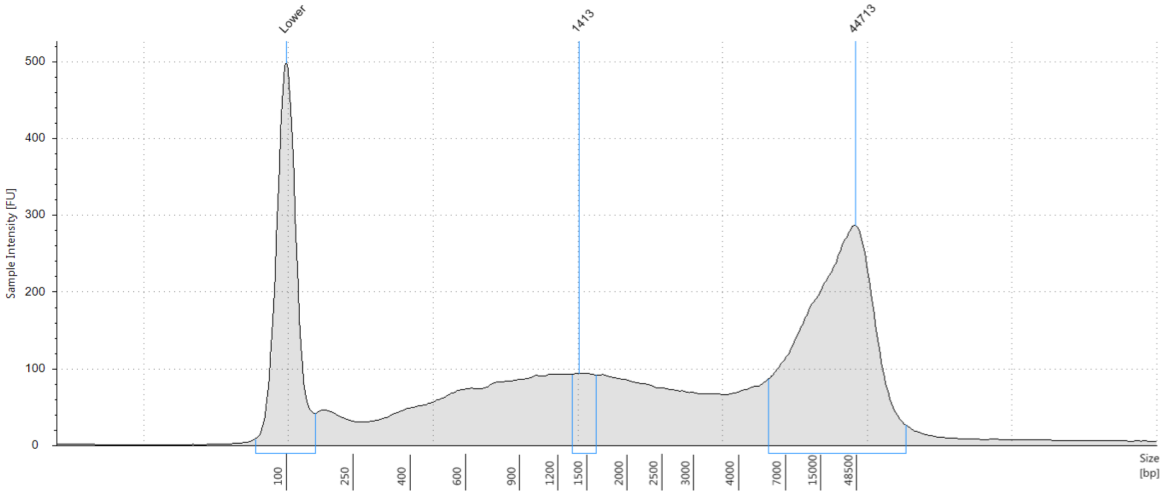

Sample Table

Well	DIN	Conc. [ng/µl]	Sample Description	Alert	Observations
C1	5.9	41.4	cat1: 2		

Peak Table

Size [bp]	Calibrated Conc. [ng/µl]	Assigned Conc. [ng/µl]	% Integrated Area	From [bp]	To [bp]	Peak Comment	Observations
100	8.50	8.50	-	65	148		Lower Marker
1413	1.68	-	6.60	1345	1608		
44713	16.6	-	65.35	5749	>60000		
-	-	-	-	-	-		Sample Well

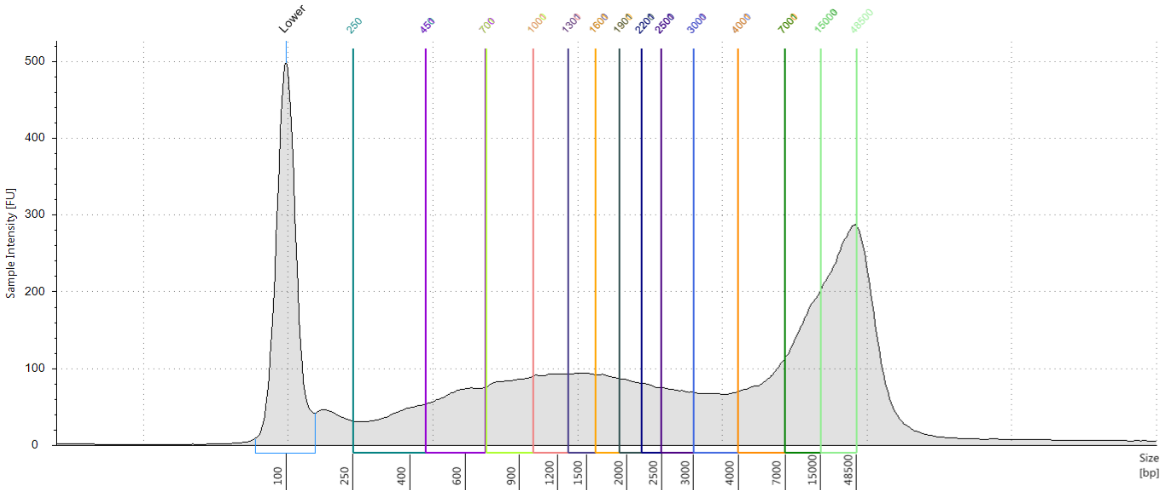

Region Table

From [bp]	To [bp]	Average Size [bp]	Conc. [ng/µl]	Region Molarity [nmol/l]	% of Total	Region Comment	Color
250	450	357	2.04	9.66	4.93		
451	700	576	2.93	8.28	7.08		
701	1000	849	2.92	5.53	7.06		
1001	1300	1156	2.31	3.19	5.58		
1301	1600	1462	1.93	2.10	4.66		

 © Agilent Technologies, Inc. 2016

1601	1900	1757	1.61	1.46	3.90		
1901	2200	2060	1.42	1.10	3.44		
2201	2500	2364	1.18	0.795	2.85		
2501	3000	2763	1.72	0.994	4.15		
3001	4000	3509	2.15	0.988	5.19		
4001	7000	5580	2.97	0.871	7.16		
7001	15000	11133	4.18	0.620	10.10		
15001	48500	25546	6.79	0.460	16.39		

 © Agilent Technologies, Inc. 2016

23699
S. burchellii 2019

D1: cat1: 3

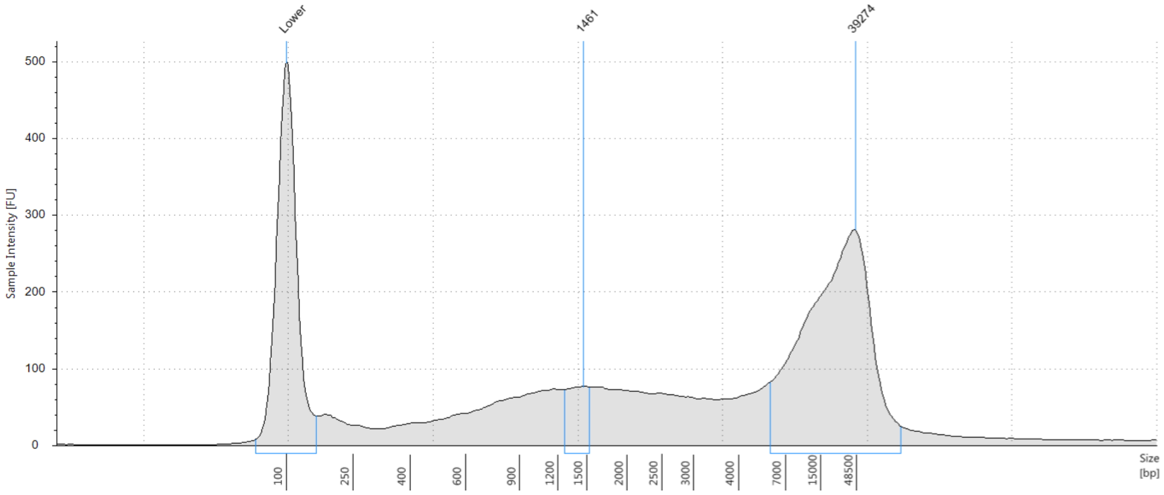

Sample Table

Well	DIN	Conc. [ng/µl]	Sample Description	Alert	Observations
D1	6.1	36.1	cat1: 3		

Peak Table

Size [bp]	Calibrated Conc. [ng/µl]	Assigned Conc. [ng/µl]	% Integrated Area	From [bp]	To [bp]	Peak Comment	Observations
100	8.50	8.50	-	65	150		Lower Marker
1461	1.41	-	6.58	1263	1530		
39274	15.3	-	71.23	5816	>60000		
-	-	-	-	-	-		Sample Well

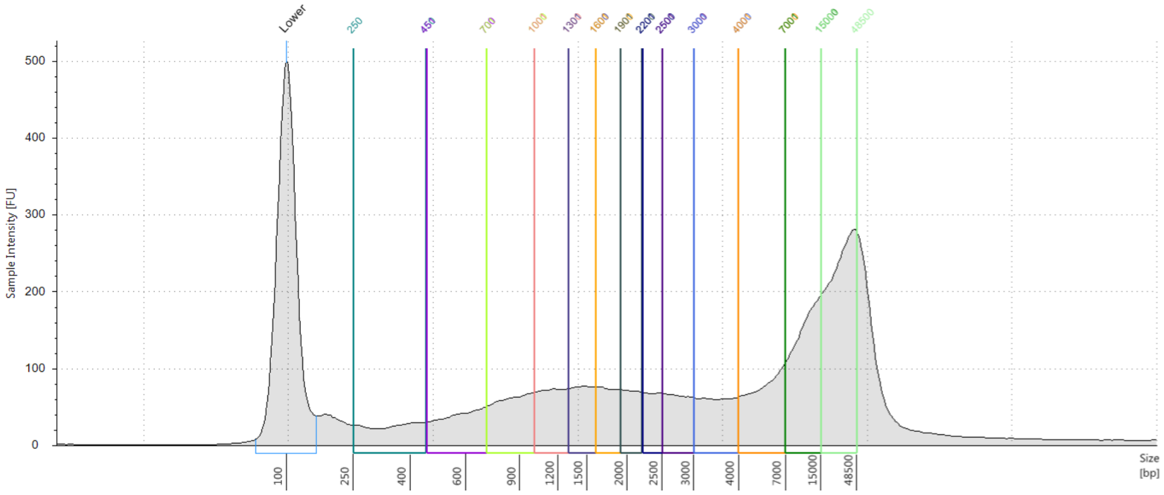

Region Table

From [bp]	To [bp]	Average Size [bp]	Conc. [ng/µl]	Region Molarity [nmol/l]	% of Total	Region Comment	Color
250	450	350	1.29	6.38	3.57		
451	700	582	1.68	4.79	4.67		
701	1000	857	2.16	4.07	5.98		
1001	1300	1159	1.86	2.57	5.17		
1301	1600	1468	1.56	1.69	4.31		

1601	1900	1760	1.32	1.19	3.65		
1901	2200	2061	1.20	0.926	3.32		
2201	2500	2362	1.03	0.692	2.84		
2501	3000	2755	1.55	0.897	4.29		
3001	4000	3502	1.99	0.914	5.52		
4001	7000	5573	2.73	0.801	7.57		
7001	15000	11164	4.20	0.621	11.64		
15001	48500	25690	6.58	0.442	18.23		

20479
S. noctiflora 2018

E1: cat1: 4

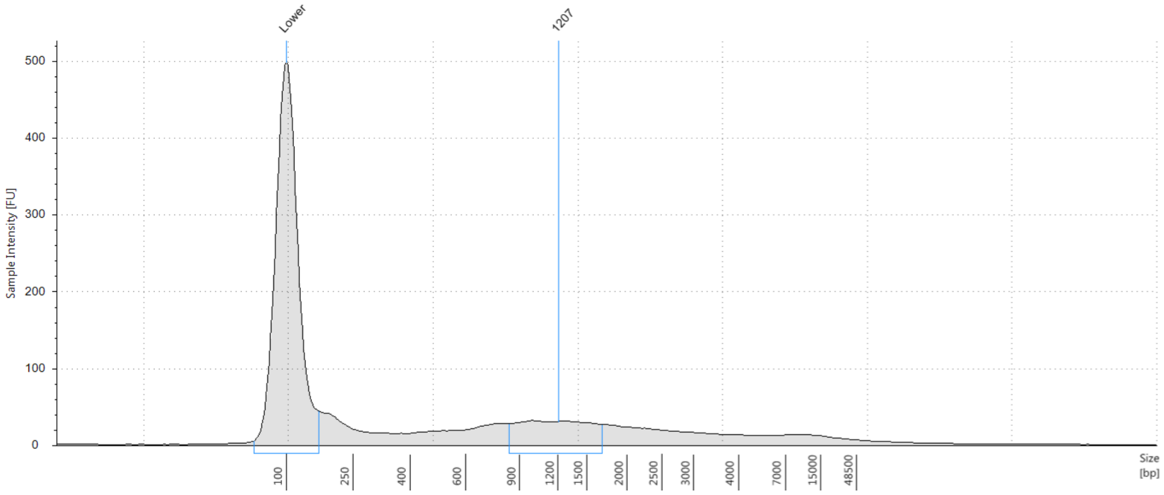

Sample Table

Well	DIN	Conc. [ng/µl]	Sample Description	Alert	Observations
E1	2.6	7.27	cat1: 4	⚠	Sample concentration outside recommended range

Peak Table

Size [bp]	Calibrated Conc. [ng/µl]	Assigned Conc. [ng/µl]	% Integrated Area	From [bp]	To [bp]	Peak Comment	Observations
100	8.50	8.50	-	64	156		Lower Marker
1207	1.82	-	94.02	829	1681		
-	-	-	-	-	-		Sample Well

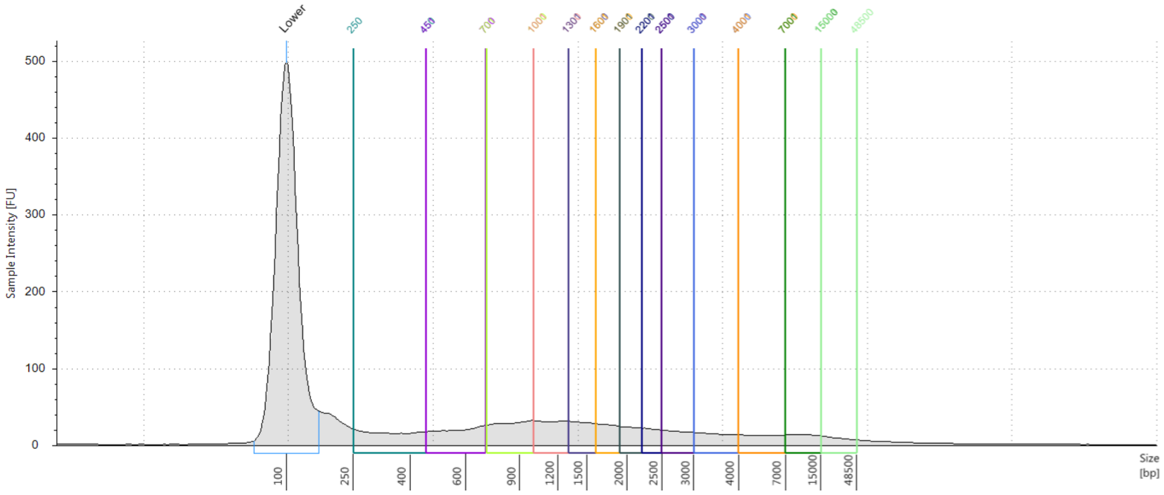

Region Table

From [bp]	To [bp]	Average Size [bp]	Conc. [ng/µl]	Region Molarity [nmol/l]	% of Total	Region Comment	Color
250	450	344	0.750	3.69	10.32		
451	700	577	0.778	2.22	10.70		
701	1000	850	0.898	1.71	12.36		
1001	1300	1156	0.691	0.959	9.52		
1301	1600	1460	0.544	0.597	7.49		

 © Agilent Technologies, Inc. 2016

1601	1900	1756	0.420	0.384	5.78		
1901	2200	2060	0.348	0.272	4.79		
2201	2500	2363	0.279	0.191	3.85		
2501	3000	2760	0.376	0.222	5.17		
3001	4000	3491	0.409	0.195	5.63		
4001	7000	5452	0.388	0.122	5.34		
7001	15000	10706	0.297	0.0486	4.09		
15001	48500	23478	0.205	0.0165	2.82		

© Agilent Technologies, Inc. 2016

18108
S. acaulis 1994

F1: cat2: 1

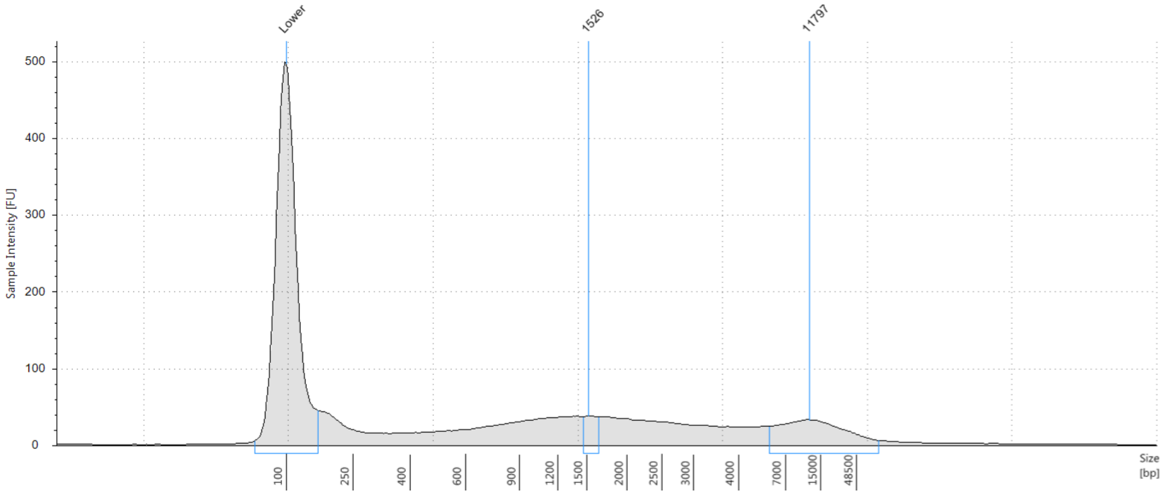

Sample Table

Well	DIN	Conc. [ng/µl]	Sample Description	Alert	Observations
F1	3.2	10.5	cat2: 1		

Peak Table

Size [bp]	Calibrated Conc. [ng/µl]	Assigned Conc. [ng/µl]	% Integrated Area	From [bp]	To [bp]	Peak Comment	Observations
100	8.50	8.50	-	65	154		Lower Marker
1526	0.425	-	18.29	1470	1638		
11797	1.76	-	75.72	5809	>60000		
-	-	-	-	-	-		Sample Well

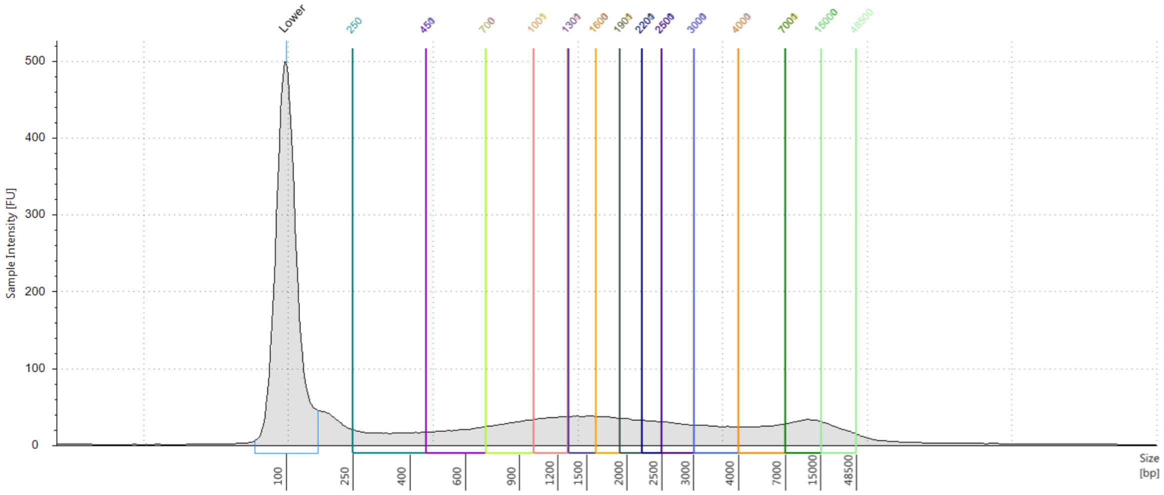

Region Table

From [bp]	To [bp]	Average Size [bp]	Conc. [ng/µl]	Region Molarity [nmol/l]	% of Total	Region Comment	Color
250	450	347	0.812	3.95	7.77		
451	700	580	0.833	2.36	7.97		
701	1000	860	0.974	1.82	9.32		
1001	1300	1157	0.869	1.20	8.32		
1301	1600	1463	0.751	0.815	7.18		

1601	1900	1759	0.633	0.571	6.05		
1901	2200	2064	0.554	0.426	5.30		
2201	2500	2370	0.459	0.308	4.39		
2501	3000	2758	0.630	0.365	6.02		
3001	4000	3501	0.789	0.364	7.55		
4001	7000	5498	0.809	0.243	7.74		
7001	15000	10786	0.785	0.122	7.51		
15001	48500	23063	0.581	0.0445	5.56		

24702
S. burchellii 1987

G1: cat2: 2

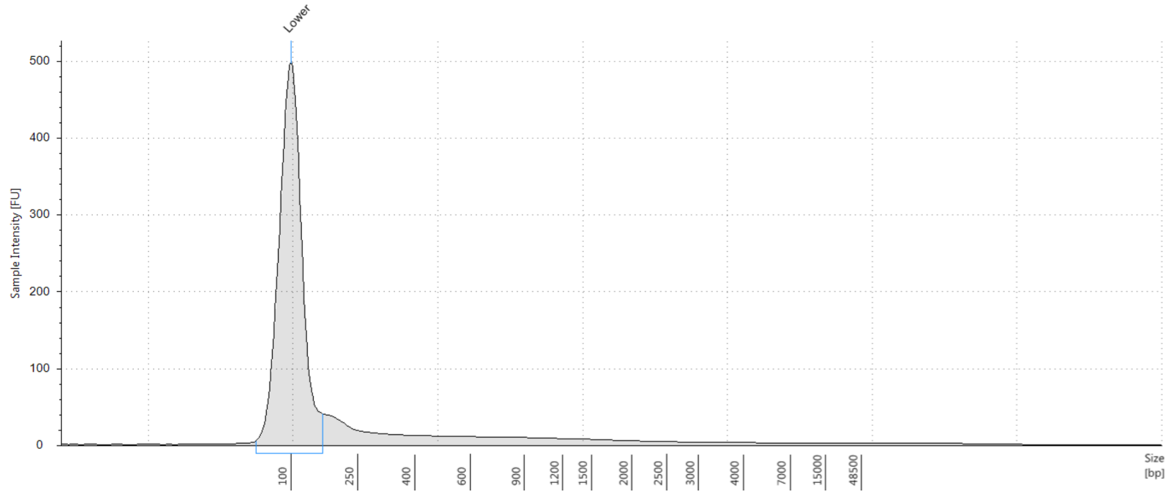

Sample Table

Well	DIN	Conc. [ng/µl]	Sample Description	Alert	Observations
G1	1.3	3.10	cat2: 2	⚠	Sample concentration outside functional range for DIN

Peak Table

Size [bp]	Calibrated Conc. [ng/µl]	Assigned Conc. [ng/µl]	% Integrated Area	From [bp]	To [bp]	Peak Comment	Observations
100	8.50	8.50	-	62	154		Lower Marker
-	-	-	-	-	-		Sample Well

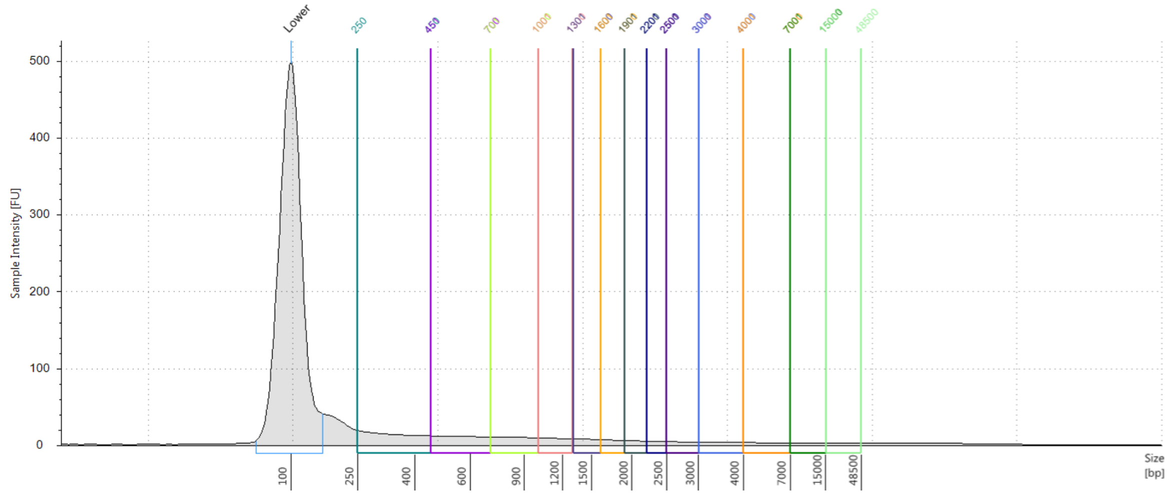

Region Table

From [bp]	To [bp]	Average Size [bp]	Conc. [ng/µl]	Region Molarity [nmol/l]	% of Total	Region Comment	Color
250	450	340	0.646	3.22	20.84		
451	700	569	0.419	1.25	13.53		
701	1000	849	0.296	0.593	9.54		
1001	1300	1151	0.184	0.274	5.93		
1301	1600	1459	0.131	0.156	4.24		

1601	1900	1757	0.0928	0.0935	2.99		
1901	2200	2063	0.0746	0.0654	2.41		
2201	2500	2370	0.0580	0.0451	1.87		
2501	3000	2758	0.0744	0.0515	2.40		
3001	4000	3491	0.0878	0.0503	2.83		
4001	7000	5439	0.0753	0.0296	2.43		
7001	15000	10648	0.0503	0.0107	1.62		
15001	48500	24788	0.0508	0.00491	1.64		

 © Agilent Technologies, Inc. 2016

24712
S. noctiflora 1981

H1: cat2: 3

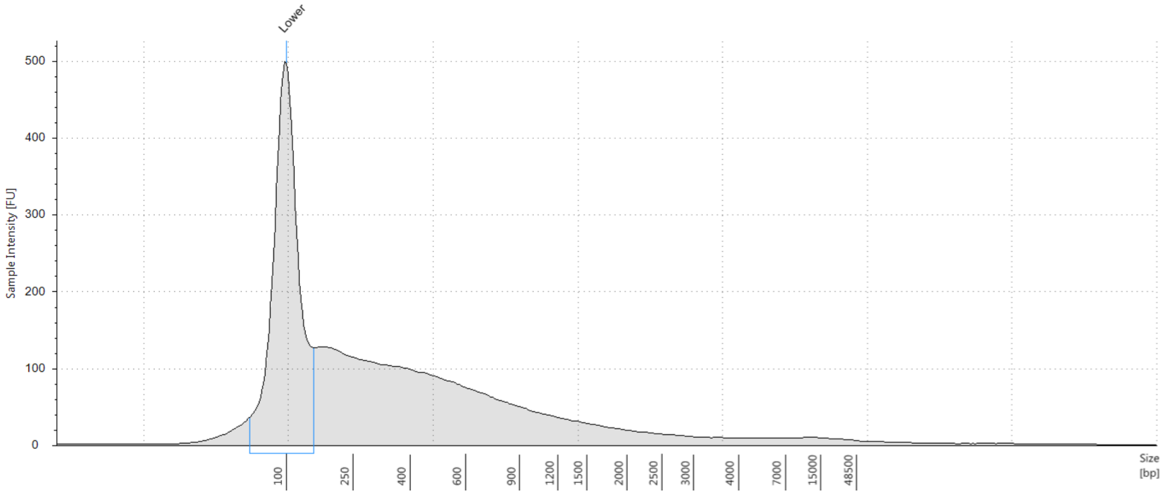

Sample Table

Well	DIN	Conc. [ng/µl]	Sample Description	Alert	Observations
H1	1.3	15.3	cat2: 3		

Peak Table

Size [bp]	Calibrated Conc. [ng/µl]	Assigned Conc. [ng/µl]	% Integrated Area	From [bp]	To [bp]	Peak Comment	Observations
100	8.50	8.50	-	61	146		Lower Marker
-	-	-	-	-	-		Sample Well

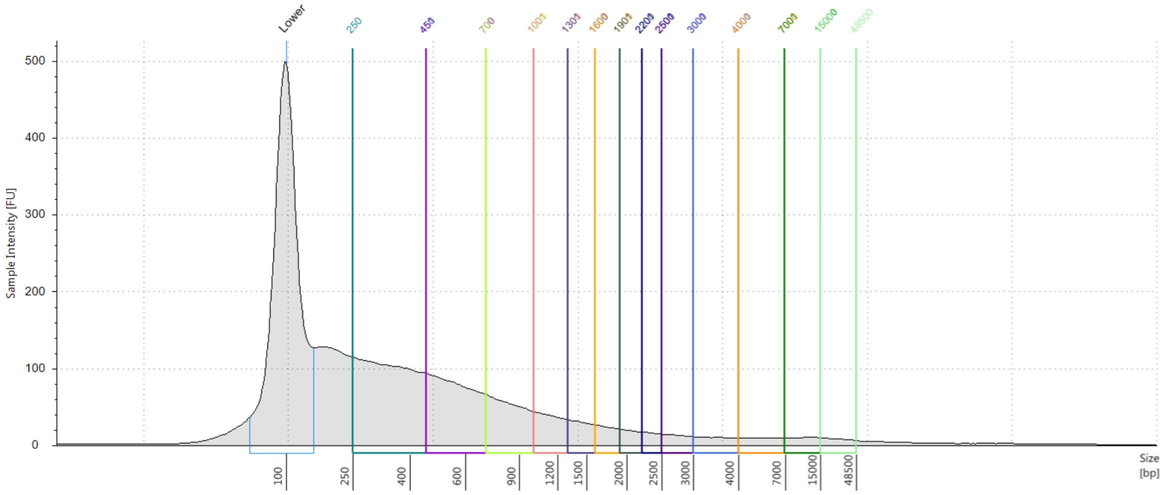

Region Table

From [bp]	To [bp]	Average Size [bp]	Conc. [ng/µl]	Region Molarity [nmol/l]	% of Total	Region Comment	Color
250	450	342	4.49	21.1	29.29		
451	700	563	2.87	8.08	18.68		
701	1000	840	1.54	2.90	10.03		
1001	1300	1147	0.779	1.08	5.08		
1301	1600	1457	0.494	0.539	3.22		
1601	1900	1757	0.339	0.308	2.21		

 © Agilent Technologies, Inc. 2016

1901	2200	2065	0.256	0.199	1.67		
2201	2500	2363	0.180	0.123	1.17		
2501	3000	2751	0.242	0.144	1.58		
3001	4000	3495	0.250	0.119	1.63		
4001	7000	5424	0.237	0.0751	1.55		
7001	15000	10878	0.202	0.0327	1.32		
15001	48500	24056	0.158	0.0121	1.03		

© Agilent Technologies, Inc. 2016

17365
S. involucrata ssp furcata 1979

A2: cat2: 4

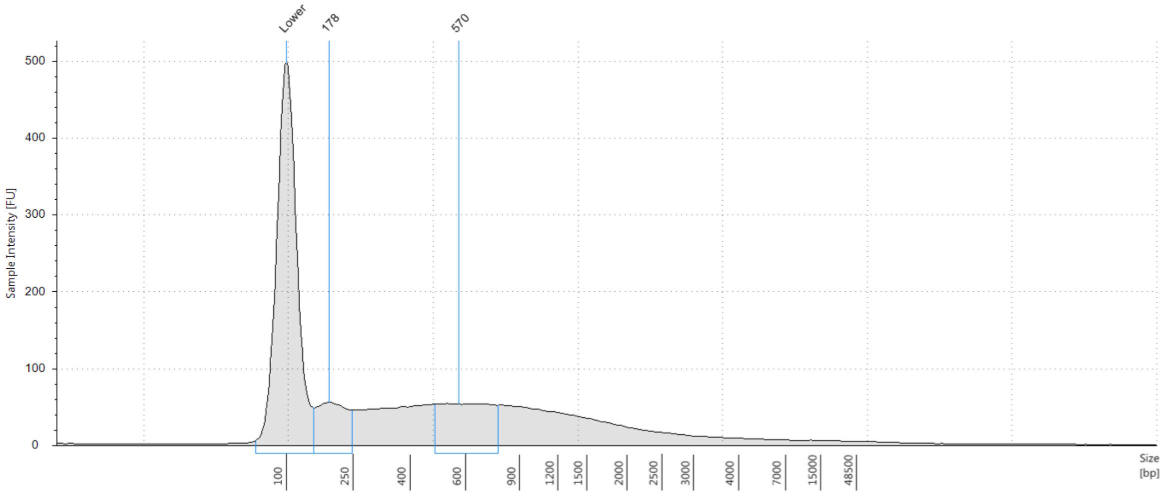

Sample Table

Well	DIN	Conc. [ng/µl]	Sample Description	Alert	Observations
A2	1.8	13.0	cat2: 4		

Peak Table

Size [bp]	Calibrated Conc. [ng/µl]	Assigned Conc. [ng/µl]	% Integrated Area	From [bp]	To [bp]	Peak Comment	Observations
100	8.50	8.50	-	65	145		Lower Marker
178	1.48	-	35.72	145	246		
570	2.50	-	60.33	480	765		
-	-	-	-	-	-		Sample Well

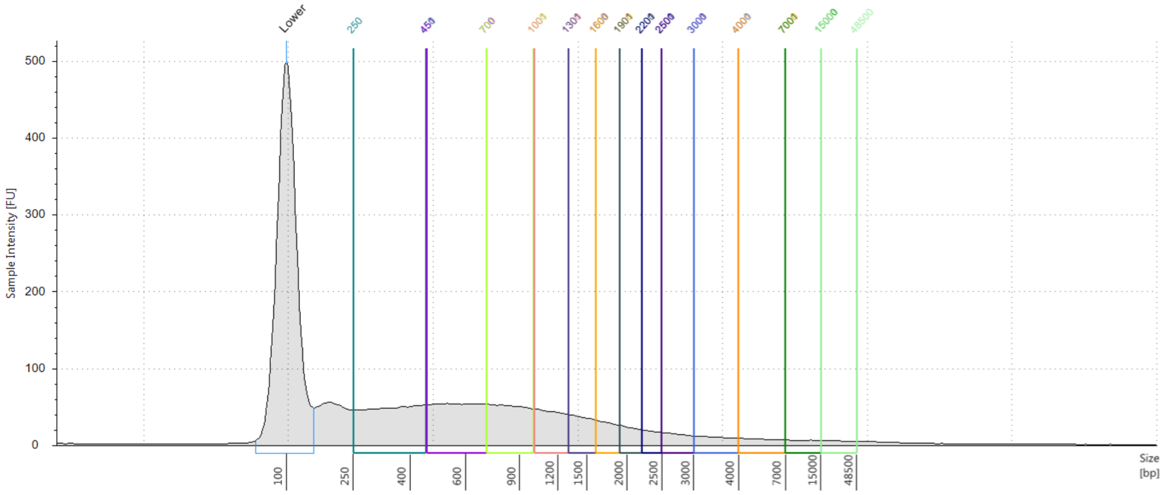

Region Table

From [bp]	To [bp]	Average Size [bp]	Conc. [ng/µl]	Region Molarity [nmol/l]	% of Total	Region Comment	Color
250	450	347	2.62	12.1	20.15		
451	700	572	2.36	6.49	18.12		
701	1000	845	1.78	3.31	13.69		
1001	1300	1150	1.10	1.49	8.44		
1301	1600	1453	0.753	0.809	5.79		

1601	1900	1752	0.548	0.488	4.21		
1901	2200	2061	0.384	0.291	2.95		
2201	2500	2364	0.276	0.183	2.12		
2501	3000	2751	0.343	0.196	2.64		
3001	4000	3487	0.339	0.155	2.61		
4001	7000	5403	0.272	0.0825	2.09		
7001	15000	10832	0.169	0.0265	1.30		
15001	48500	25117	0.145	0.0103	1.11		

 © Agilent Technologies, Inc. 2016

18313
S. acaulis 1969

B2: cat3: 1

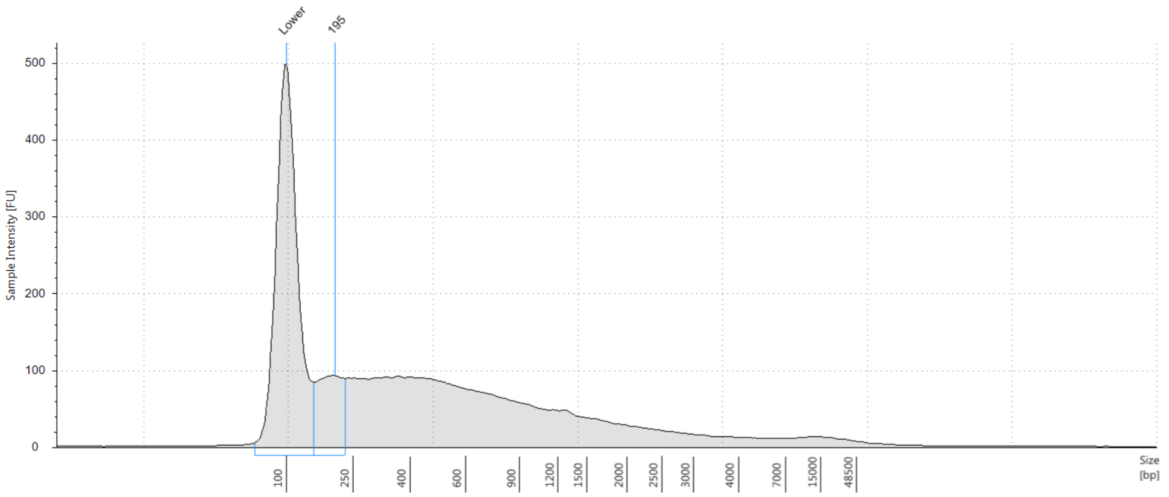

Sample Table

Well	DIN	Conc. [ng/µl]	Sample Description	Alert	Observations
B2	1.5	17.5	cat3: 1		

Peak Table

Size [bp]	Calibrated Conc. [ng/µl]	Assigned Conc. [ng/µl]	% Integrated Area	From [bp]	To [bp]	Peak Comment	Observations
100	8.50	8.50	-	65	146		Lower Marker
195	2.00	-	86.97	146	224		
-	-	-	-	-	-		Sample Well

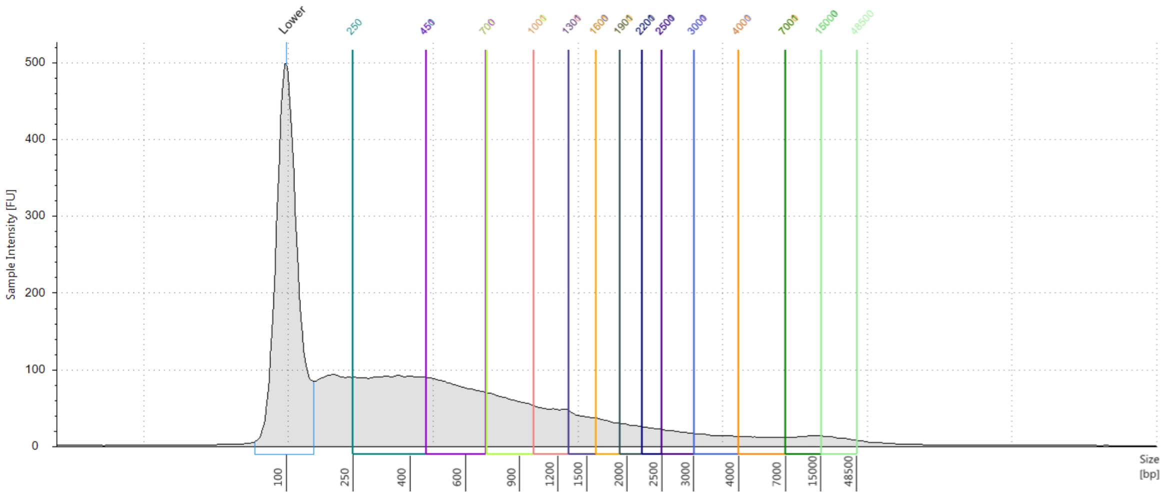

Region Table

From [bp]	To [bp]	Average Size [bp]	Conc. [ng/µl]	Region Molarity [nmol/l]	% of Total	Region Comment	Color
250	450	345	4.55	21.2	26.07		
451	700	567	3.33	9.31	19.03		
701	1000	843	2.03	3.82	11.64		
1001	1300	1154	1.20	1.65	6.88		
1301	1600	1458	0.740	0.805	4.24		

 © Agilent Technologies, Inc. 2016

1601	1900	1760	0.550	0.497	3.15		
1901	2200	2065	0.437	0.338	2.50		
2201	2500	2370	0.331	0.224	1.89		
2501	3000	2753	0.408	0.240	2.34		
3001	4000	3480	0.434	0.206	2.49		
4001	7000	5436	0.379	0.119	2.17		
7001	15000	10903	0.299	0.0479	1.71		
15001	48500	23463	0.260	0.0204	1.49		

© Agilent Technologies, Inc. 2016

20329
S. burchellii
1948

C2: cat3: 2

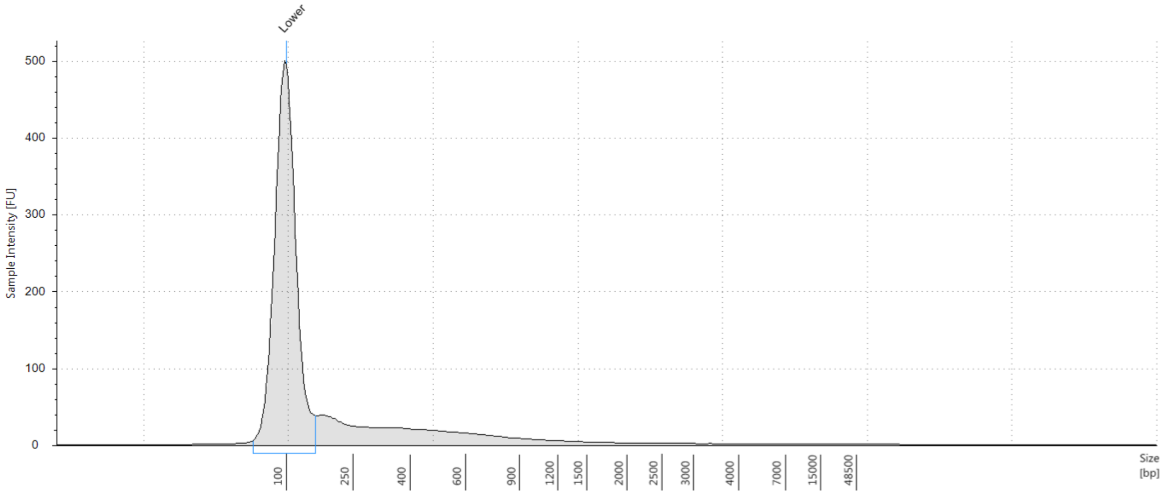

Sample Table

Well	DIN	Conc. [ng/µl]	Sample Description	Alert	Observations
C2	1.0	3.55	cat3: 2	⚠	Sample concentration outside functional range for DIN

Peak Table

Size [bp]	Calibrated Conc. [ng/µl]	Assigned Conc. [ng/µl]	% Integrated Area	From [bp]	To [bp]	Peak Comment	Observations
100	8.50	8.50	-	63	150		Lower Marker
-	-	-	-	-	-		Sample Well

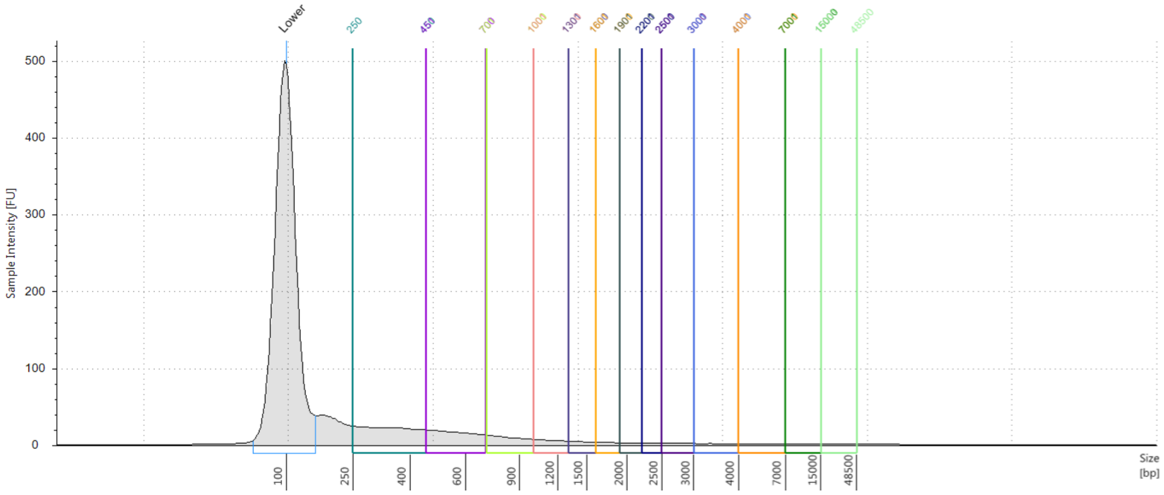

Region Table

From [bp]	To [bp]	Average Size [bp]	Conc. [ng/µl]	Region Molarity [nmol/l]	% of Total	Region Comment	Color
250	450	343	1.12	5.32	31.40		
451	700	561	0.687	1.99	19.35		
701	1000	836	0.327	0.650	9.20		
1001	1300	1149	0.140	0.209	3.93		
1301	1600	1458	0.0777	0.0947	2.19		

1601	1900	1755	0.0534	0.0561	1.50		
1901	2200	2057	0.0402	0.0371	1.13		
2201	2500	2370	0.0310	0.0255	0.87		
2501	3000	2755	0.0435	0.0320	1.22		
3001	4000	3494	0.0484	0.0299	1.36		
4001	7000	5406	0.0413	0.0180	1.16		
7001	15000	10806	0.0270	0.00638	0.76		
15001	48500	24619	0.0244	0.00275	0.69		

© Agilent Technologies, Inc. 2016

17368
S. uralensis
ssp uralensis
1959

D2: cat3: 3

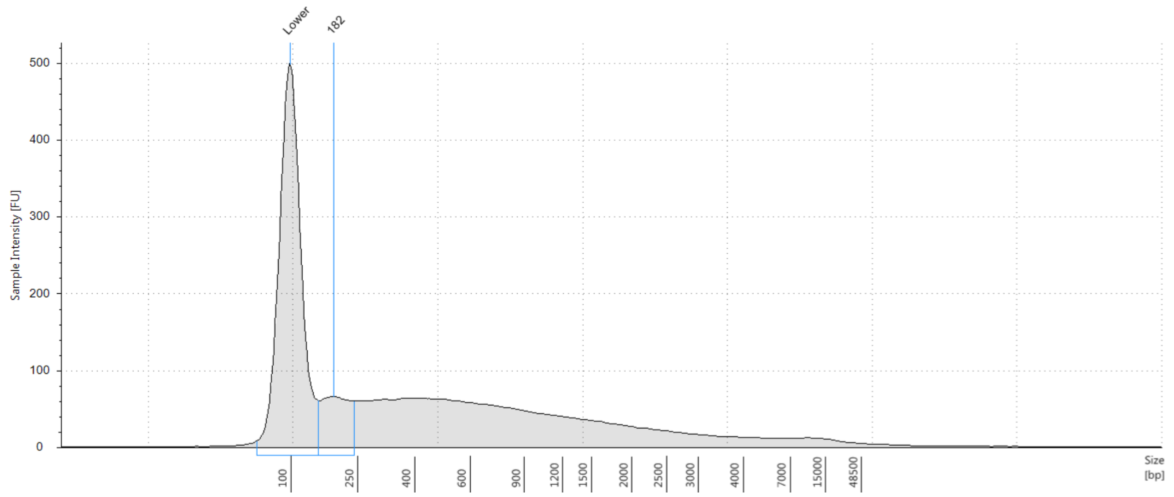

Sample Table

Well	DIN	Conc. [ng/µl]	Sample Description	Alert	Observations
D2	1.8	13.1	cat3: 3		

Peak Table

Size [bp]	Calibrated Conc. [ng/µl]	Assigned Conc. [ng/µl]	% Integrated Area	From [bp]	To [bp]	Peak Comment	Observations
100	8.50	8.50	-	63	147		Lower Marker
182	1.56	-	98.77	147	241		
-	-	-	-	-	-		Sample Well

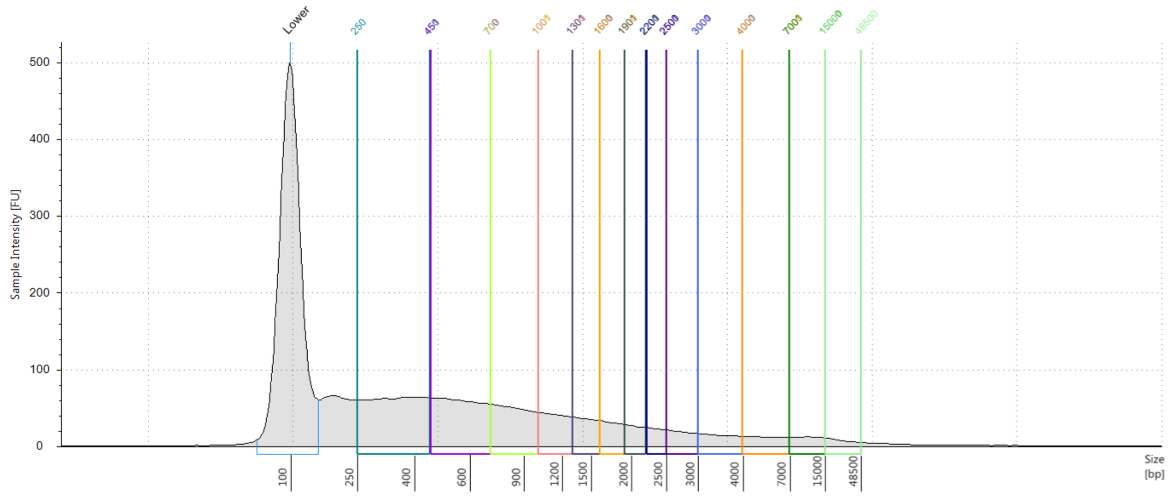

Region Table

From [bp]	To [bp]	Average Size [bp]	Conc. [ng/µl]	Region Molarity [nmol/l]	% of Total	Region Comment	Color
250	450	348	3.08	14.3	23.48		
451	700	569	2.43	6.81	18.53		
701	1000	845	1.60	3.02	12.22		
1001	1300	1151	0.975	1.35	7.43		
1301	1600	1458	0.662	0.722	5.05		

© Agilent Technologies, Inc. 2016

1601	1900	1753	0.519	0.471	3.95		
1901	2200	2064	0.417	0.323	3.18		
2201	2500	2367	0.304	0.206	2.32		
2501	3000	2751	0.402	0.237	3.06		
3001	4000	3486	0.423	0.201	3.23		
4001	7000	5441	0.371	0.117	2.83		
7001	15000	10871	0.275	0.0446	2.10		
15001	48500	23684	0.162	0.0133	1.24		

© Agilent Technologies, Inc. 2016

23558
S. rigens 1932

E2: cat3: 4

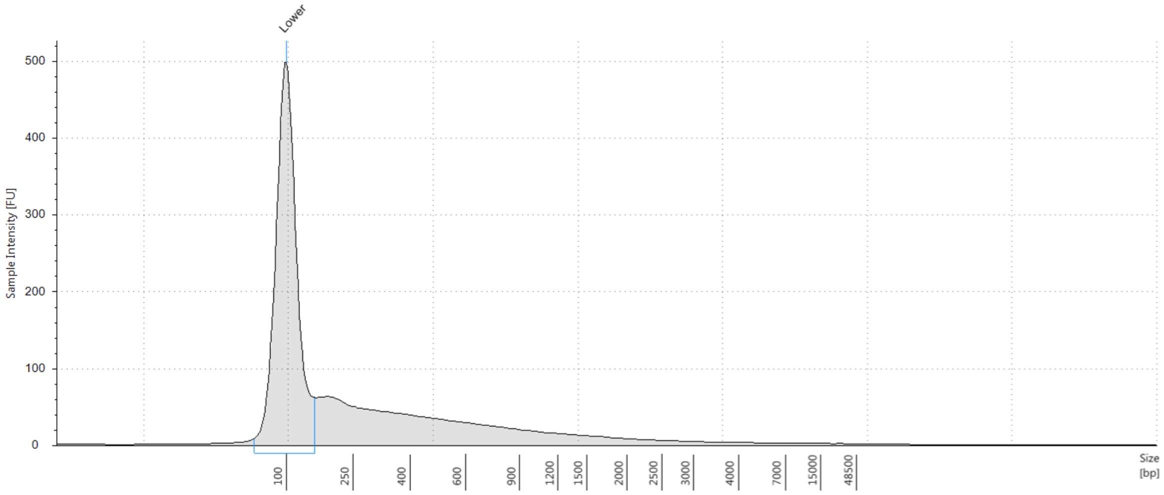

Sample Table

Well	DIN	Conc. [ng/µl]	Sample Description	Alert	Observations
E2	1.2	7.48	cat3: 4		Sample concentration outside recommended range

Peak Table

Size [bp]	Calibrated Conc. [ng/µl]	Assigned Conc. [ng/µl]	% Integrated Area	From [bp]	To [bp]	Peak Comment	Observations
100	8.50	8.50	-	64	148		Lower Marker
-	-	-	-	-	-		Sample Well

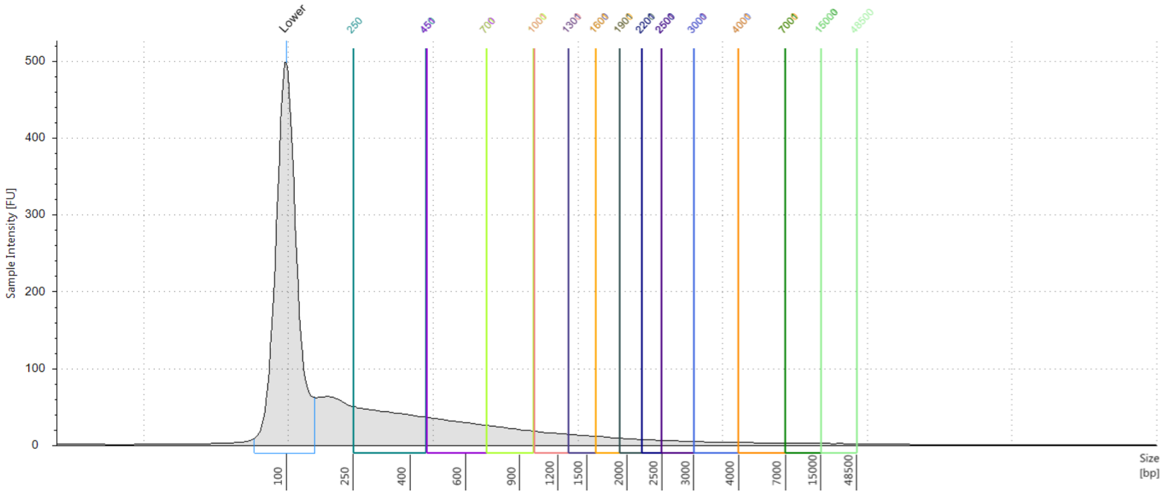

Region Table

From [bp]	To [bp]	Average Size [bp]	Conc. [ng/µl]	Region Molarity [nmol/l]	% of Total	Region Comment	Color
250	450	341	2.24	10.6	29.88		
451	700	564	1.30	3.69	17.44		
701	1000	840	0.740	1.41	9.89		
1001	1300	1150	0.397	0.555	5.31		
1301	1600	1461	0.245	0.271	3.28		
1601	1900	1758	0.177	0.164	2.36		

 © Agilent Technologies, Inc. 2016

1901	2200	2068	0.124	0.0991	1.66		
2201	2500	2363	0.0921	0.0650	1.23		
2501	3000	2752	0.117	0.0729	1.57		
3001	4000	3481	0.118	0.0599	1.57		
4001	7000	5388	0.0929	0.0328	1.24		
7001	15000	10672	0.0530	0.0101	0.71		
15001	48500	24350	0.0454	0.00414	0.61		

© Agilent Technologies, Inc. 2016

F2: J1

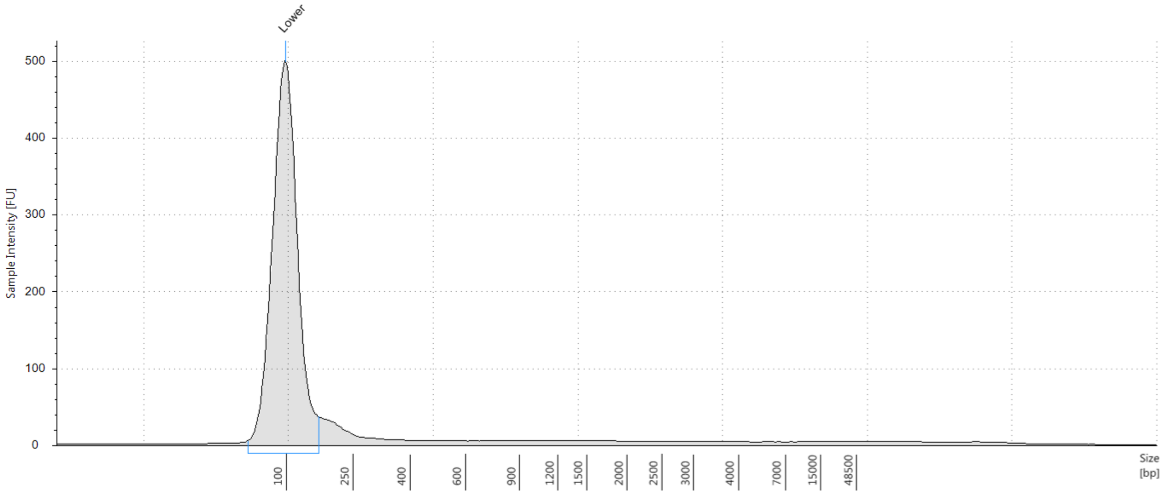

Sample Table

Well	DIN	Conc. [ng/µl]	Sample Description	Alert	Observations
F2	-	2.41	J1	⚠	Sample concentration outside functional range for DIN

Peak Table

Size [bp]	Calibrated Conc. [ng/µl]	Assigned Conc. [ng/µl]	% Integrated Area	From [bp]	To [bp]	Peak Comment	Observations
100	8.50	8.50	-	59	157		Lower Marker
-	-	-	-	-	-		Sample Well

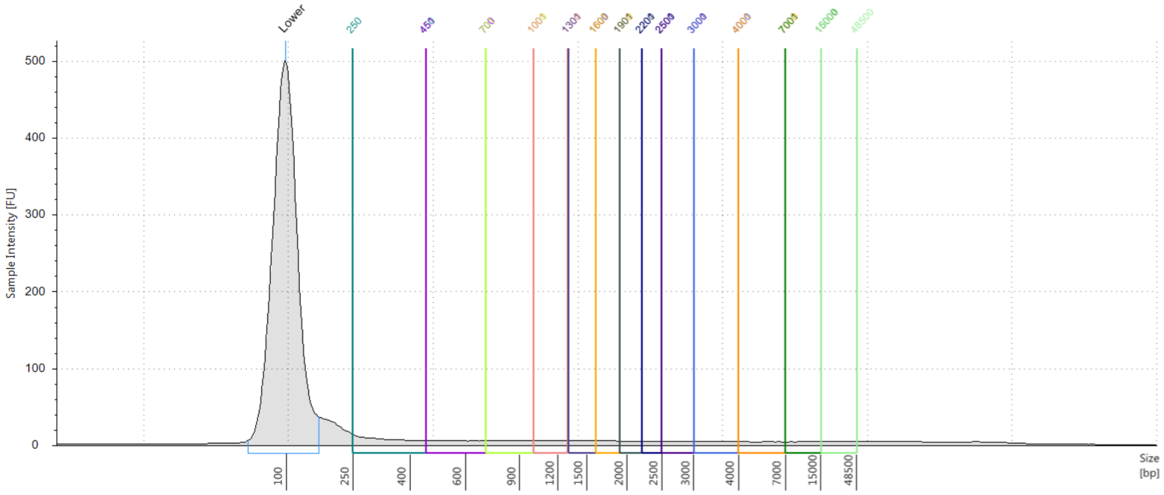

Region Table

From [bp]	To [bp]	Average Size [bp]	Conc. [ng/µl]	Region Molarity [nmol/l]	% of Total	Region Comment	Color
250	450	334	0.319	1.71	13.22		
451	700	569	0.174	0.566	7.20		
701	1000	852	0.139	0.300	5.76		
1001	1300	1152	0.106	0.167	4.38		
1301	1600	1460	0.0883	0.109	3.66		

1601	1900	1758	0.0729	0.0754	3.02		
1901	2200	2069	0.0636	0.0565	2.64		
2201	2500	2365	0.0527	0.0410	2.18		
2501	3000	2760	0.0820	0.0557	3.40		
3001	4000	3510	0.100	0.0547	4.16		
4001	7000	5454	0.101	0.0368	4.20		
7001	15000	10802	0.0794	0.0148	3.29		
15001	48500	25350	0.0879	0.00731	3.64		

 © Agilent Technologies, Inc. 2016

G2: J2

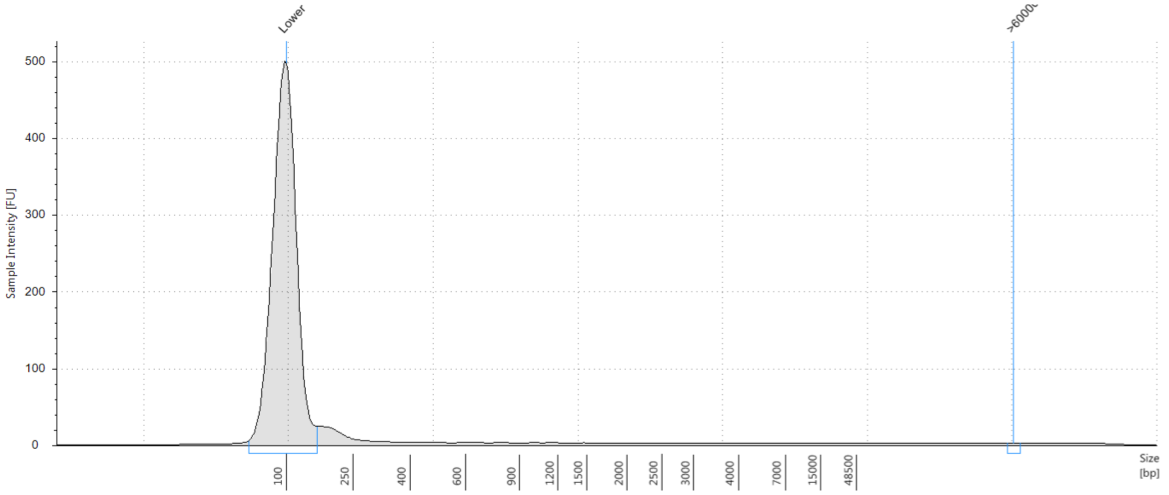

Sample Table

Well	DIN	Conc. [ng/µl]	Sample Description	Alert	Observations
G2	-	1.57	J2	⚠	Sample concentration outside functional range for DIN

Peak Table

Size [bp]	Calibrated Conc. [ng/µl]	Assigned Conc. [ng/µl]	% Integrated Area	From [bp]	To [bp]	Peak Comment	Observations
100	8.50	8.50	-	60	153		Lower Marker
>60000	0.0211	-	59.77	>60000	>60000		
-	-	-	-	-	-		Sample Well

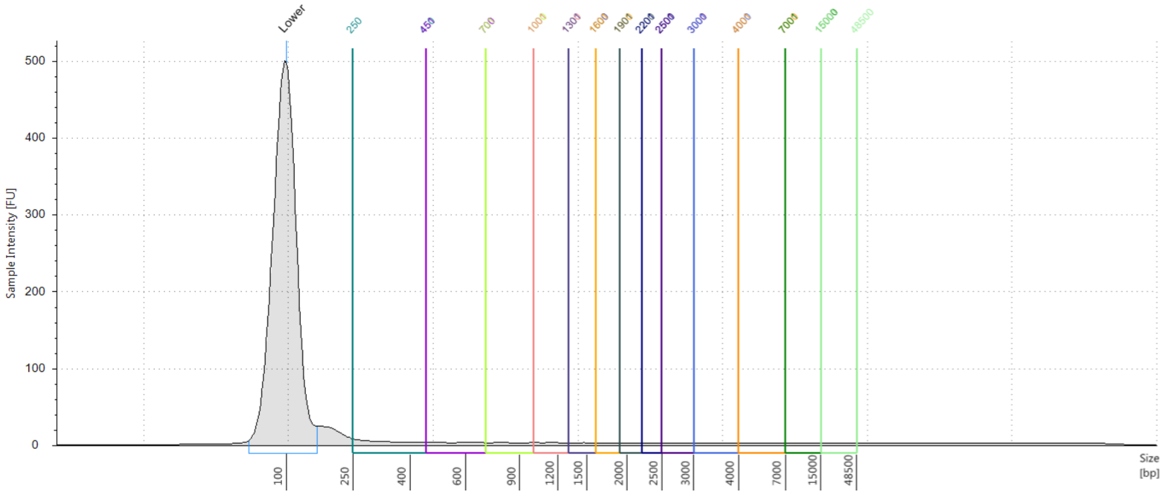

Region Table

From [bp]	To [bp]	Average Size [bp]	Conc. [ng/µl]	Region Molarity [nmol/l]	% of Total	Region Comment	Color
250	450	333	0.190	1.07	12.11		
451	700	571	0.104	0.361	6.64		
701	1000	852	0.0796	0.185	5.06		
1001	1300	1151	0.0568	0.0967	3.61		
1301	1600	1462	0.0461	0.0622	2.93		

© Agilent Technologies, Inc. 2016

1601	1900	1767	0.0378	0.0427	2.40		
1901	2200	2074	0.0335	0.0322	2.13		
2201	2500	2368	0.0289	0.0245	1.84		
2501	3000	2762	0.0431	0.0322	2.74		
3001	4000	3494	0.0525	0.0320	3.34		
4001	7000	5463	0.0530	0.0214	3.37		
7001	15000	10868	0.0423	0.00880	2.69		
15001	48500	25642	0.0437	0.00403	2.78		

 © Agilent Technologies, Inc. 2016

H2: J3

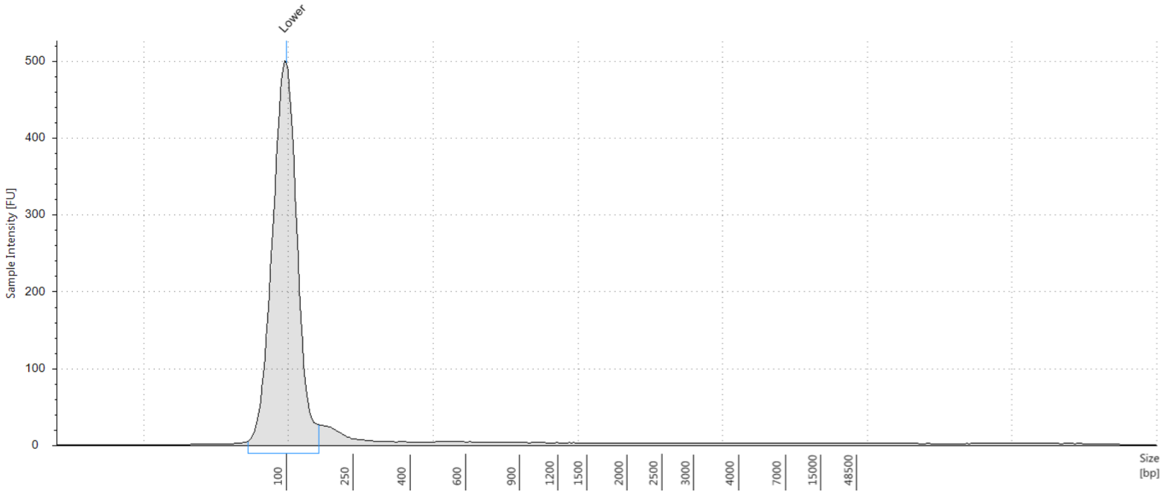

Sample Table

Well	DIN	Conc. [ng/µl]	Sample Description	Alert	Observations
H2	-	1.47	J3	⚠	Sample concentration outside functional range for DIN

Peak Table

Size [bp]	Calibrated Conc. [ng/µl]	Assigned Conc. [ng/µl]	% Integrated Area	From [bp]	To [bp]	Peak Comment	Observations
100	8.50	8.50	-	59	156		Lower Marker
-	-	-	-	-	-		Sample Well

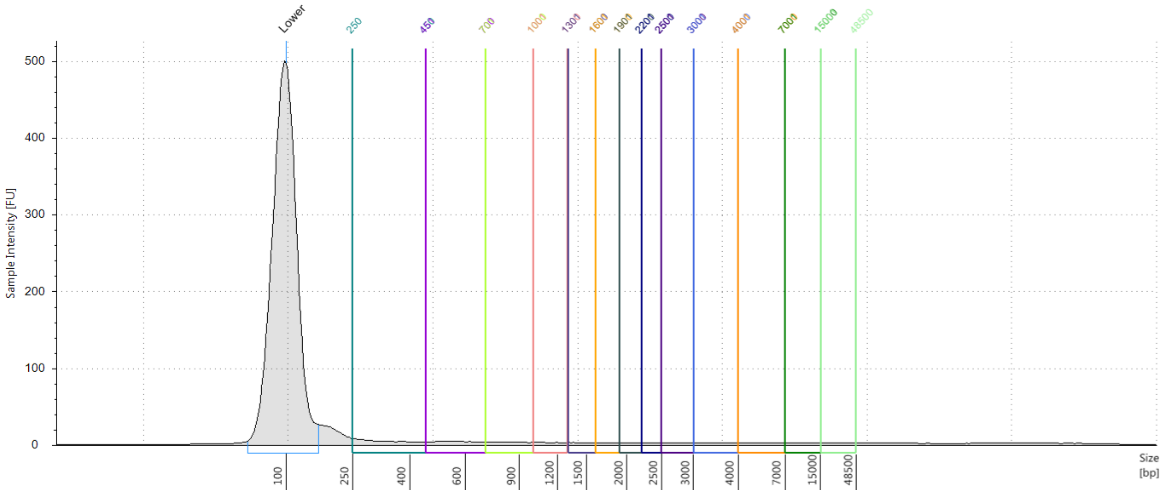

Region Table

From [bp]	To [bp]	Average Size [bp]	Conc. [ng/µl]	Region Molarity [nmol/l]	% of Total	Region Comment	Color
250	450	335	0.202	1.12	13.71		
451	700	569	0.135	0.446	9.15		
701	1000	843	0.0853	0.198	5.78		
1001	1300	1152	0.0561	0.0969	3.81		
1301	1600	1461	0.0421	0.0576	2.85		

 © Agilent Technologies, Inc. 2016

1601	1900	1761	0.0359	0.0413	2.44		
1901	2200	2064	0.0295	0.0295	2.00		
2201	2500	2360	0.0253	0.0223	1.72		
2501	3000	2759	0.0410	0.0311	2.78		
3001	4000	3504	0.0548	0.0334	3.72		
4001	7000	5406	0.0527	0.0216	3.57		
7001	15000	10809	0.0434	0.00903	2.95		
15001	48500	25634	0.0451	0.00414	3.06		

© Agilent Technologies, Inc. 2016

Calibration

Molecular Weight Settings

Fitting type: Genomic DNA Sizing
Alignment type: From lower marker

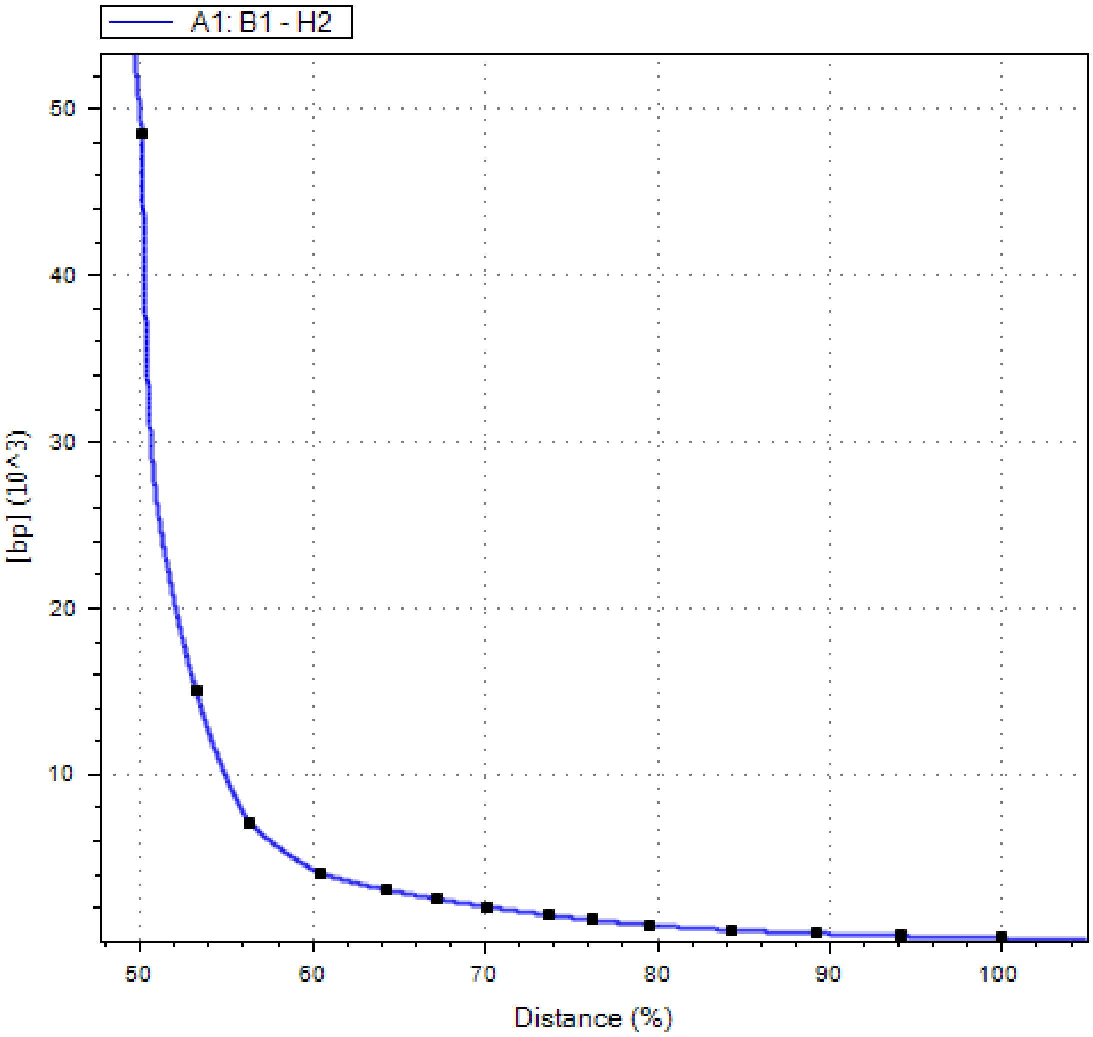

Concentration Settings

Calibration mode: Lower Marker
Normalise peaks from: Lower Marker
Fitting type: Linear Regression

Experiment Properties

Run Properties

**Analysis Software Version**	2.1.38.8716
**Filename**	C:\Users\admin\Desktop\AnneSophie\2019-september-october-herbarium\after_first_size_selection\2019-09-06-01_after_first_size_selection.gDNA
**Assay**	Genomic DNA
**Run End Date**	06-Sep-2019 5:06 PM
**Last Saved Under Version**	2.1.38.8716
**DIN Version**	2.1.38.8716
**Study**	
**Comments**	

ScreenTape Device 1

**Username**	admin
**ScreenTape Device ID**	01-S025-190812-01-000056
**Expiry Date**	20-Sep-2019
**ScreenTape Device History**	First run 06-Sep-2019, 1 run performed
**Temperature [°C]**	24
**Electrophoresis Time [s]**	222
**Instrument Type**	6655
**Instrument Serial Number**	03-PM405
**Notes**	
**ScreenTape Device Run Date**	06-Sep-2019 4:44 PM

Controller Environment

**Computer**	LAB3210150
**Instrument Controller Software Version**	A.02.01 SR1
**First Run Analysis Version**	2.1.38.8716
**Operating System**	Microsoft Windows 7 Enterprise
