# Supplementary material for: Retrieval of long DNA reads from herbarium specimens
Source: AoB Plants. 2023 Nov 8;15(6):plad074. doi: 10.1093/aobpla/plad074 (PMC10735254; doi:10.1093/aobpla/plad074)
Supplement: plad074_suppl_Supplementary_Appendix_S1_3 [file plad074_suppl_supplementary_appendix_s1_3.pdf]

Filename: after_PCRbarcoding.gDNA

Gel Image

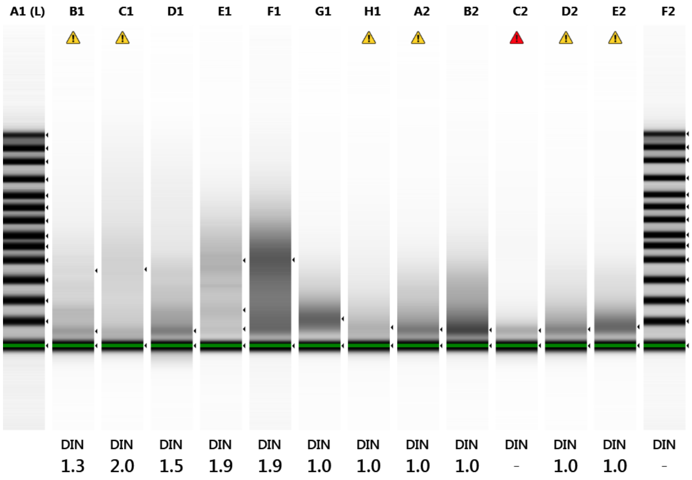

Default image (Contrast 100%)

Sample Info

Well	DIN	Conc. [ng/µl]	Sample Description	Alert	Observations
A1	-	74.1	Ladder		Ladder
B1	1.3	7.51	Cat1: 1	⚠	Sample concentration outside recommended range
C1	2.0	9.04	Cat1: 2	⚠	Sample concentration outside recommended range
D1	1.5	10.4	Cat1: 3		
E1	1.9	11.8	Cat1: 4		
F1	1.9	28.4	Cat2: 1		
G1	1.0	10.2	Cat2: 2		
H1	1.0	4.57	Cat2: 3	⚠	Sample concentration outside functional range for DIN
A2	1.0	8.16	Cat2: 4	⚠	Sample concentration outside recommended range
B2	1.0	13.9	Cat3: 1		
C2	-	2.95	Cat3: 2	⚠	Sample concentration outside functional range for DIN
D2	1.0	6.28	Cat3: 3	⚠	Sample concentration outside recommended range
E2	1.0	8.51	Cat3: 4	⚠	Sample concentration outside recommended range
F2	-	70.8	Ladder		Ladder run as sample

 © Agilent Technologies, Inc. 2016

A1: Ladder

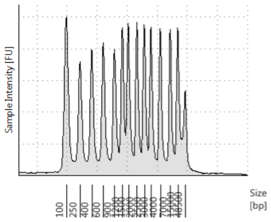

B1: Cat1: 1

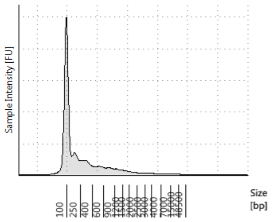

C1: Cat1: 2

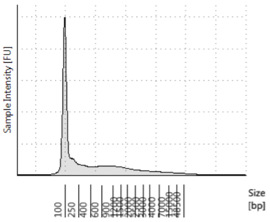

D1: Cat1: 3

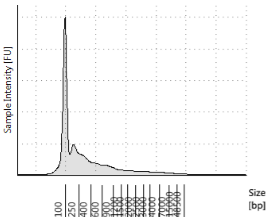

E1: Cat1: 4

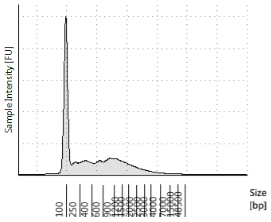

F1: Cat2: 1

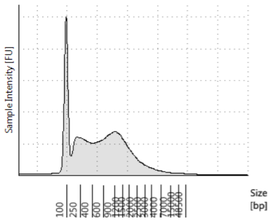

G1: Cat2: 2

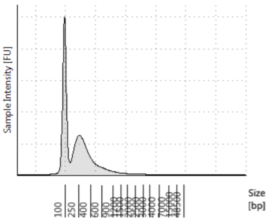

H1: Cat2: 3

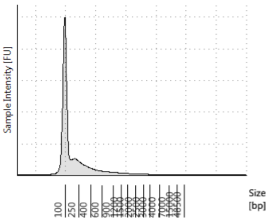

A2: Cat2: 4

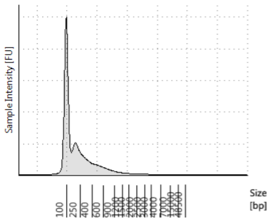

B2: Cat3: 1

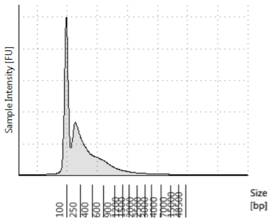

C2: Cat3: 2

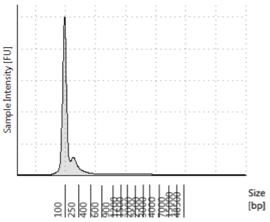

D2: Cat3: 3

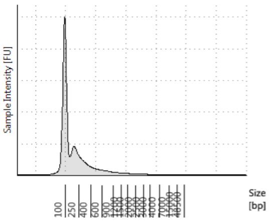

E2: Cat3: 4

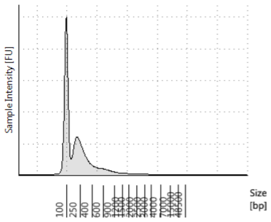

F2: Ladder

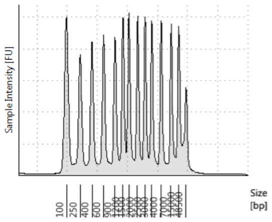

 © Agilent Technologies, Inc. 2016

A1: Ladder

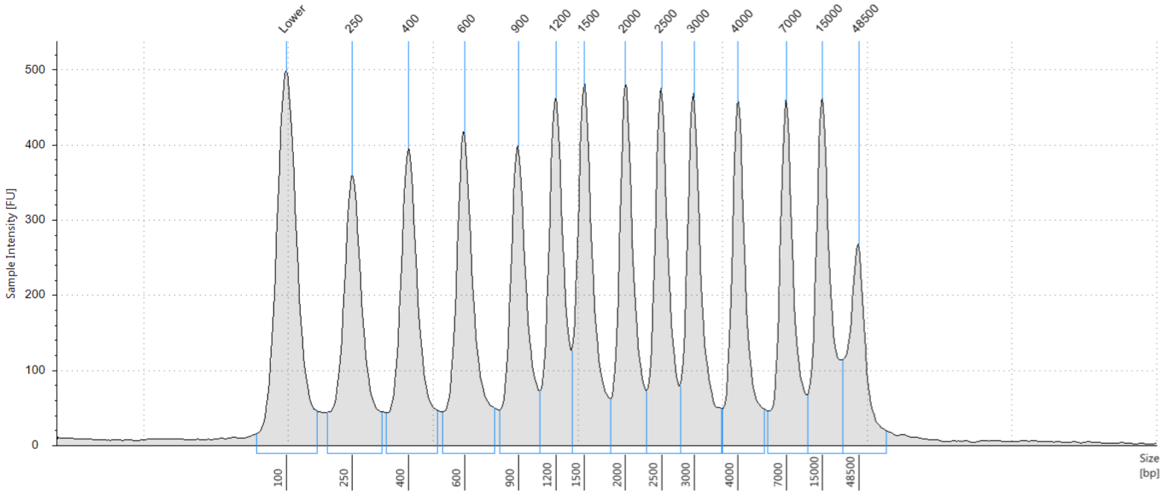

Sample Table

Well	DIN	Conc. [ng/µl]	Sample Description	Alert	Observations
A1	-	74.1	Ladder		Ladder

Peak Table

Size [bp]	Calibrated Conc. [ng/µl]	Assigned Conc. [ng/µl]	% Integrated Area	From [bp]	To [bp]	Peak Comment	Observations
100	8.50	8.50	-	66	154		Lower Marker
250	5.77	-	8.04	177	321		
400	5.87	-	8.18	331	492		
600	6.08	-	8.48	510	752		
900	5.34	-	7.44	781	1059		
1200	5.74	-	8.00	1059	1361		
1500	6.22	-	8.66	1361	1801		
2000	5.74	-	8.00	1801	2279		
2500	5.54	-	7.72	2279	2777		
3000	5.63	-	7.84	2777	3595		
4000	5.34	-	7.45	3624	5407		
7000	5.26	-	7.34	5644	10943		
15000	5.59	-	7.79	10943	22849		
48500	3.64	-	5.07	22849	>60000		

 © Agilent Technologies, Inc. 2016

B1: Cat1: 1

1994 acaulis

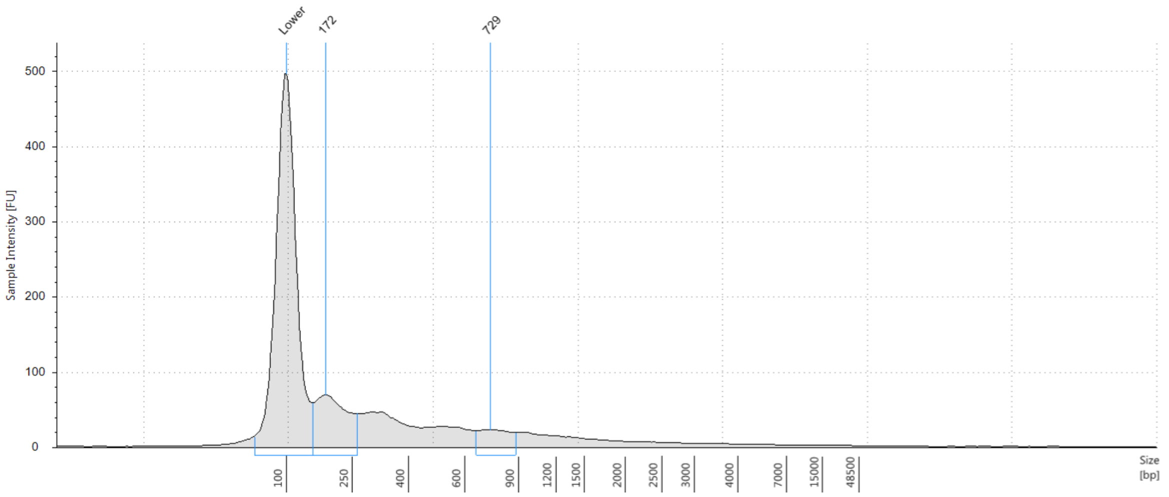

Sample Table

Well	DIN	Conc. [ng/µl]	Sample Description	Alert	Observations
B1	1.3	7.51	Cat1: 1	⚠	Sample concentration outside recommended range

Peak Table

Size [bp]	Calibrated Conc. [ng/µl]	Assigned Conc. [ng/µl]	% Integrated Area	From [bp]	To [bp]	Peak Comment	Observations
100	8.50	8.50	-	64	145		Lower Marker
172	1.89	-	73.97	145	260		
729	0.637	-	24.87	656	883		
-	-	-	-	-	-		Sample Well

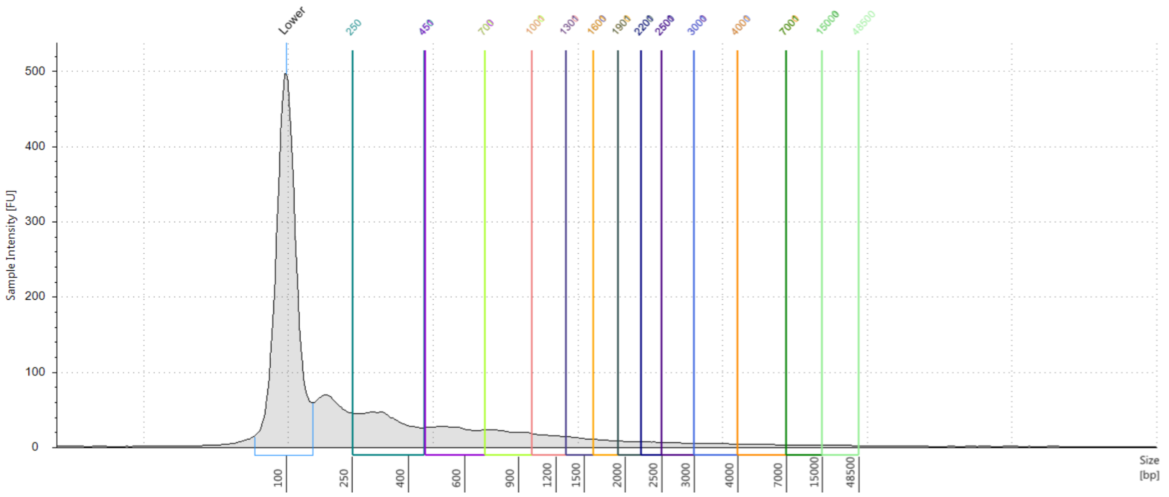

Region Table

From [bp]	To [bp]	Average Size [bp]	Conc. [ng/µl]	Region Molarity [nmol/l]	% of Total	Region Comment	Color
250	450	336	2.01	9.62	26.79		
451	700	564	1.10	3.11	14.67		
701	1000	840	0.724	1.37	9.64		
1001	1300	1146	0.399	0.554	5.32		

 © Agilent Technologies, Inc. 2016

1301	1600	1454	0.249	0.274	3.32		
1601	1900	1755	0.167	0.152	2.22		
1901	2200	2059	0.126	0.0991	1.68		
2201	2500	2365	0.106	0.0730	1.41		
2501	3000	2757	0.132	0.0784	1.75		
3001	4000	3492	0.147	0.0704	1.95		
4001	7000	5383	0.120	0.0391	1.60		
7001	15000	10563	0.0674	0.0118	0.90		
15001	48500	24243	0.0556	0.00457	0.74		

© Agilent Technologies, Inc. 2016

C1: Cat1: 2

1987 burchellii

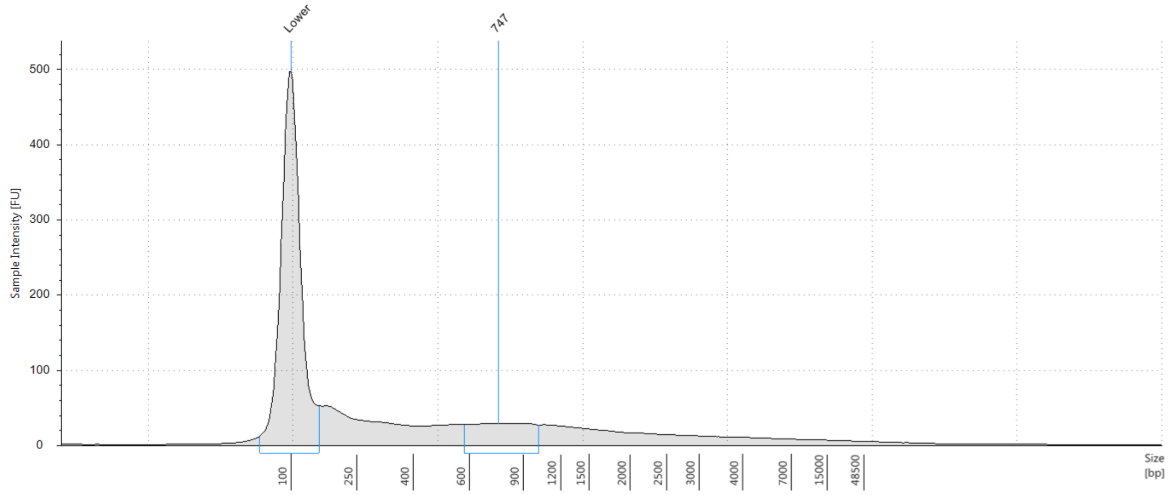

Sample Table

Well	DIN	Conc. [ng/µl]	Sample Description	Alert	Observations
C1	2.0	9.04	Cat1: 2	⚠	Sample concentration outside recommended range

Peak Table

Size [bp]	Calibrated Conc. [ng/µl]	Assigned Conc. [ng/µl]	% Integrated Area	From [bp]	To [bp]	Peak Comment	Observations
100	8.50	8.50	-	65	147		Lower Marker
747	1.60	-	98.28	579	1015		
-	-	-	-	-	-		Sample Well

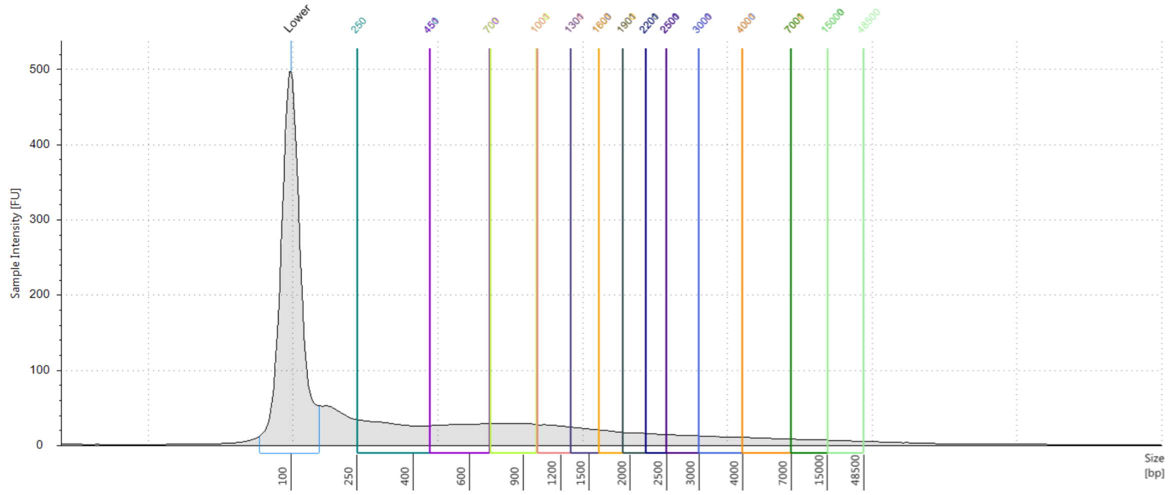

Region Table

From [bp]	To [bp]	Average Size [bp]	Conc. [ng/µl]	Region Molarity [nmol/l]	% of Total	Region Comment	Color
250	450	341	1.59	7.50	17.60		
451	700	573	1.27	3.50	14.02		
701	1000	849	1.02	1.90	11.31		
1001	1300	1151	0.686	0.934	7.58		
1301	1600	1457	0.477	0.513	5.27		

 © Agilent Technologies, Inc. 2016

1601	1900	1760	0.366	0.326	4.04		
1901	2200	2071	0.288	0.218	3.19		
2201	2500	2363	0.232	0.154	2.57		
2501	3000	2755	0.329	0.188	3.63		
3001	4000	3497	0.379	0.172	4.19		
4001	7000	5373	0.337	0.102	3.73		
7001	15000	10572	0.208	0.0332	2.30		
15001	48500	24133	0.160	0.0118	1.77		

© Agilent Technologies, Inc. 2016

D1: Cat1: 3

1981noctiflora

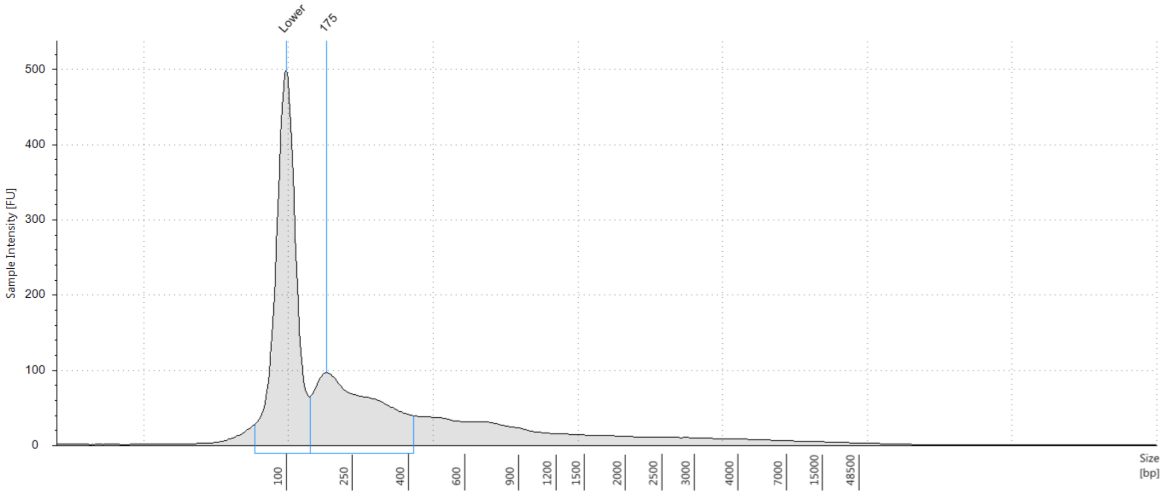

Sample Table

Well	DIN	Conc. [ng/µl]	Sample Description	Alert	Observations
D1	1.5	10.4	Cat1: 3		

Peak Table

Size [bp]	Calibrated Conc. [ng/µl]	Assigned Conc. [ng/µl]	% Integrated Area	From [bp]	To [bp]	Peak Comment	Observations
100	8.50	8.50	-	65	139		Lower Marker
175	5.03	-	99.59	139	415		
-	-	-	-	-	-		Sample Well

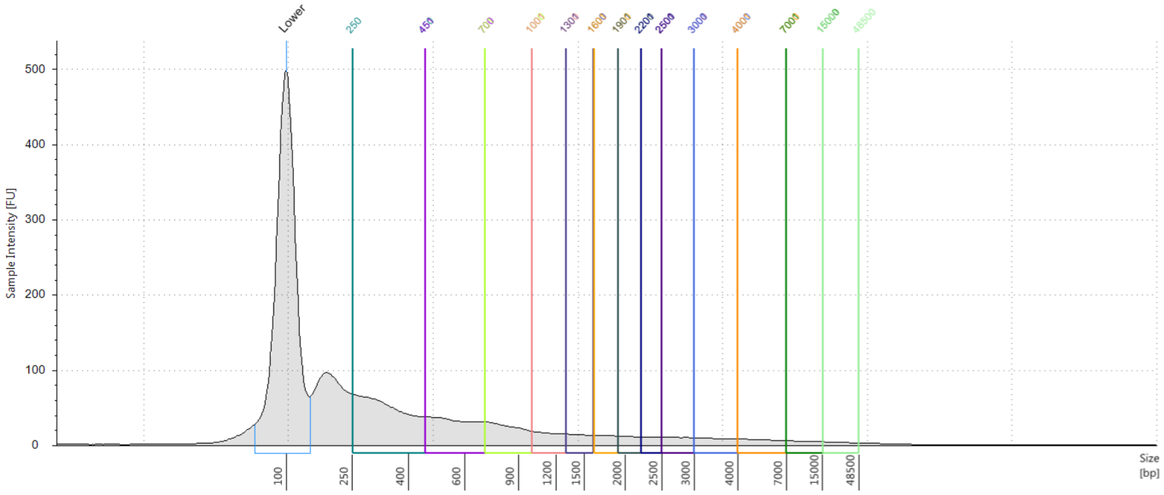

Region Table

From [bp]	To [bp]	Average Size [bp]	Conc. [ng/µl]	Region Molarity [nmol/l]	% of Total	Region Comment	Color
250	450	334	2.78	13.4	26.89		
451	700	565	1.47	4.14	14.17		
701	1000	836	0.888	1.69	8.58		
1001	1300	1155	0.404	0.562	3.90		
1301	1600	1459	0.270	0.298	2.61		

© Agilent Technologies, Inc. 2016

1601	1900	1764	0.224	0.205	2.16		
1901	2200	2064	0.193	0.151	1.86		
2201	2500	2367	0.176	0.120	1.70		
2501	3000	2759	0.236	0.139	2.28		
3001	4000	3491	0.288	0.136	2.78		
4001	7000	5374	0.249	0.0793	2.40		
7001	15000	10613	0.126	0.0216	1.22		
15001	48500	23793	0.0921	0.00760	0.89		

© Agilent Technologies, Inc. 2016

1979 S. involucrata

E1: Cat1: 4

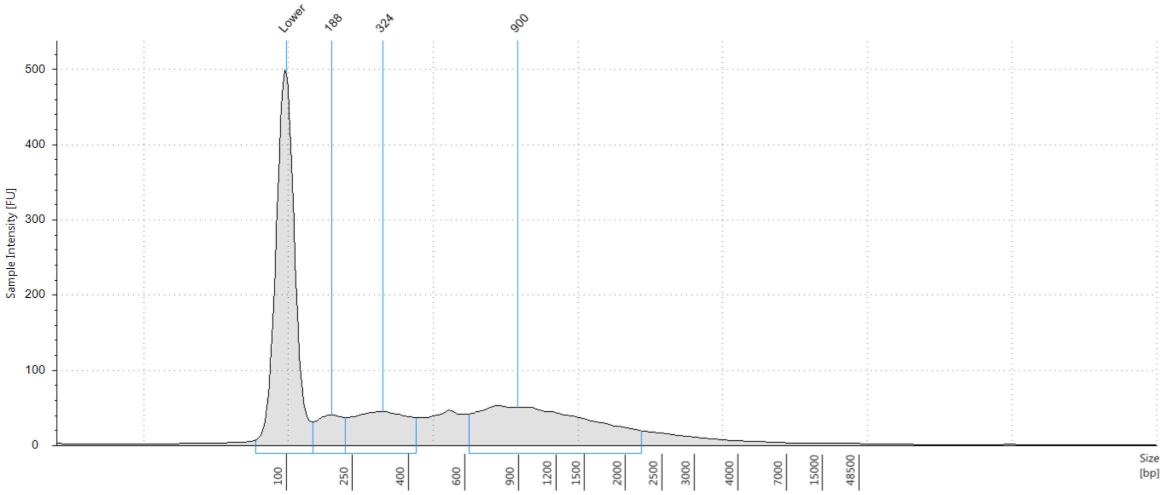

Sample Table

Well	DIN	Conc. [ng/µl]	Sample Description	Alert	Observations
E1	1.9	11.8	Cat1: 4		

Peak Table

Size [bp]	Calibrated Conc. [ng/µl]	Assigned Conc. [ng/µl]	% Integrated Area	From [bp]	To [bp]	Peak Comment	Observations
100	8.50	8.50	-	66	144		Lower Marker
188	0.982	-	11.37	144	227		
324	2.29	-	26.51	227	423		
900	5.37	-	62.11	621	2212		

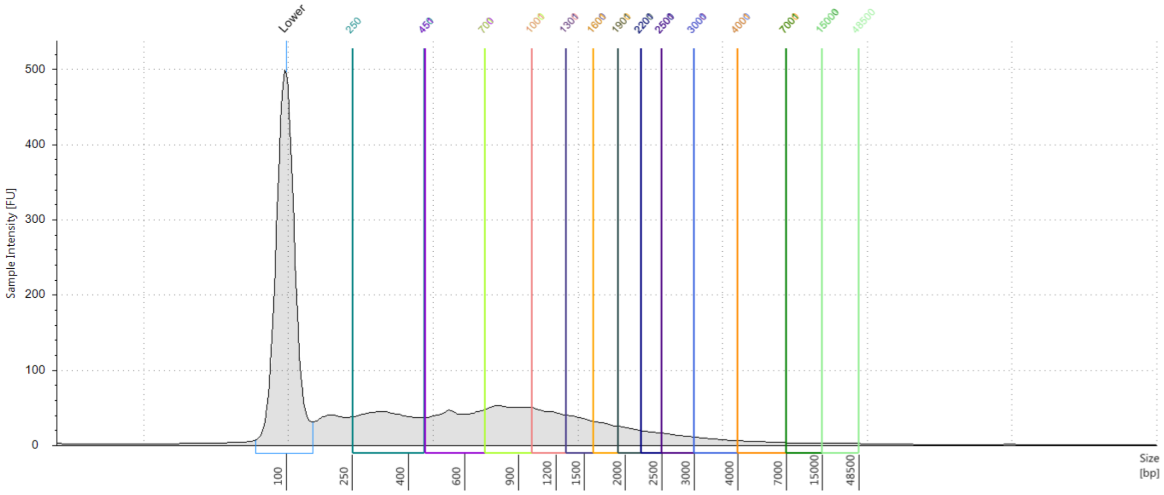

Region Table

From [bp]	To [bp]	Average Size [bp]	Conc. [ng/µl]	Region Molarity [nmol/l]	% of Total	Region Comment	Color
250	450	343	2.32	10.9	19.61		
451	700	574	2.00	5.53	16.87		
701	1000	847	1.90	3.54	16.02		
1001	1300	1145	1.23	1.69	10.39		
1301	1600	1452	0.821	0.890	6.93		

 © Agilent Technologies, Inc. 2016

1601	1900	1755	0.585	0.526	4.94		
1901	2200	2064	0.413	0.317	3.49		
2201	2500	2361	0.289	0.195	2.44		
2501	3000	2743	0.344	0.202	2.91		
3001	4000	3464	0.272	0.131	2.30		
4001	7000	5297	0.159	0.0540	1.35		
7001	15000	10673	0.0753	0.0140	0.64		
15001	48500	24205	0.0576	0.00508	0.49		

© Agilent Technologies, Inc. 2016

F1: Cat2: 1

1969 acaulis

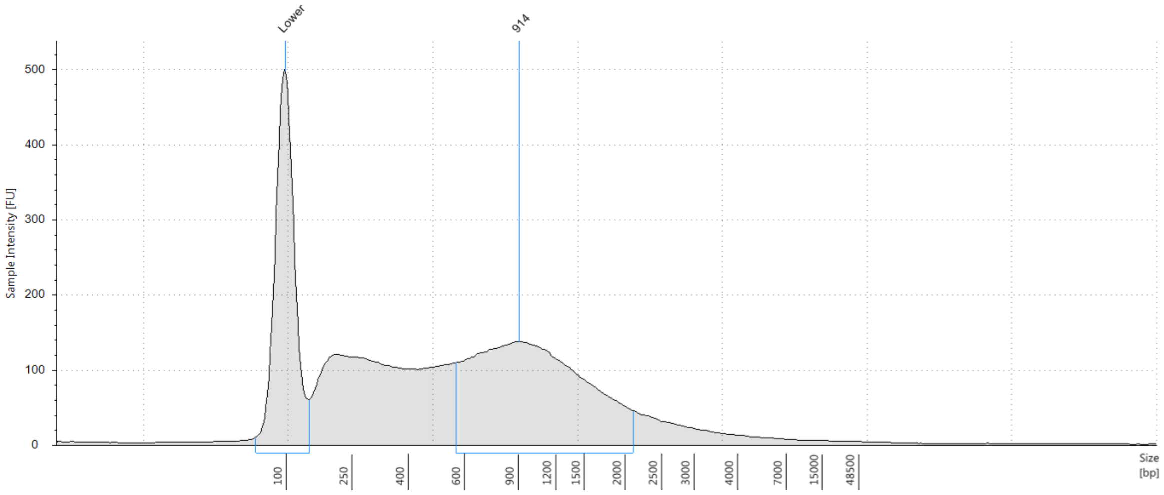

Sample Table

Well	DIN	Conc. [ng/µl]	Sample Description	Alert	Observations
F1	1.9	28.4	Cat2: 1		

Peak Table

Size [bp]	Calibrated Conc. [ng/µl]	Assigned Conc. [ng/µl]	% Integrated Area	From [bp]	To [bp]	Peak Comment	Observations
100	8.50	8.50	-	66	139		Lower Marker
914	14.1	-	100.00	568	2109		

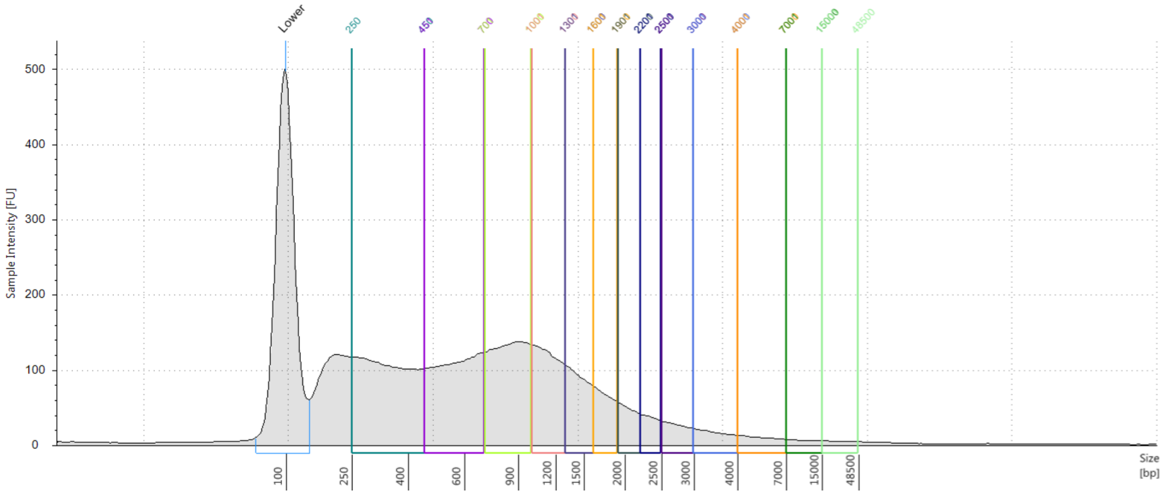

Region Table

From [bp]	To [bp]	Average Size [bp]	Conc. [ng/µl]	Region Molarity [nmol/l]	% of Total	Region Comment	Color
250	450	343	5.90	28.1	20.75		
451	700	575	5.07	14.2	17.83		
701	1000	851	4.84	9.08	17.03		
1001	1300	1146	3.20	4.44	11.25		
1301	1600	1459	1.96	2.14	6.89		
1601	1900	1757	1.30	1.19	4.57		

© Agilent Technologies, Inc. 2016

1901	2200	2061	0.838	0.664	2.95		
2201	2500	2360	0.591	0.417	2.08		
2501	3000	2748	0.630	0.393	2.22		
3001	4000	3457	0.501	0.266	1.76		
4001	7000	5316	0.298	0.120	1.05		
7001	15000	10738	0.117	0.0293	0.41		
15001	48500	24450	0.0797	0.0108	0.28		

 © Agilent Technologies, Inc. 2016

G1: Cat2: 2

1948 burchellii

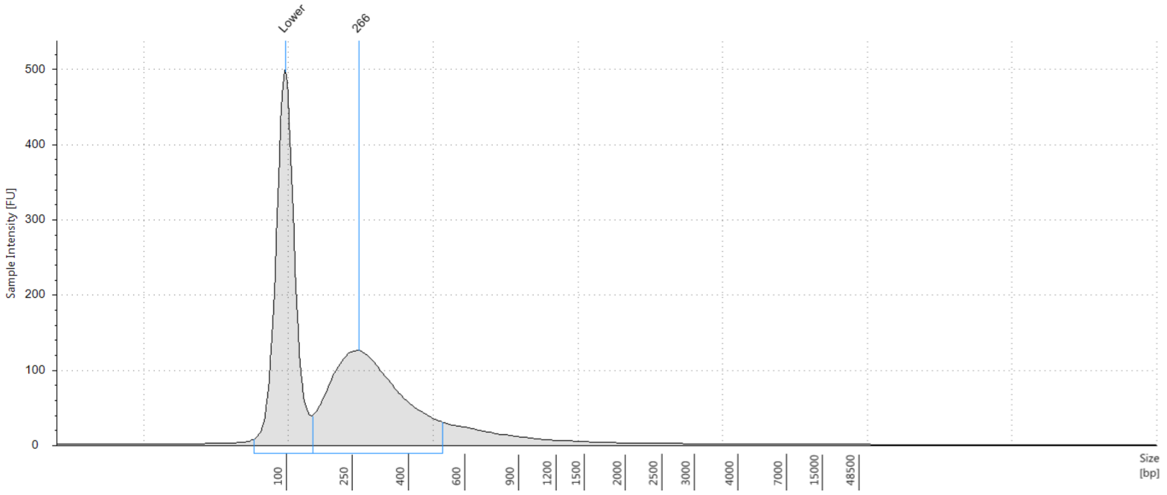

Sample Table

Well	DIN	Conc. [ng/µl]	Sample Description	Alert	Observations
G1	1.0	10.2	Cat2: 2		

Peak Table

Size [bp]	Calibrated Conc. [ng/µl]	Assigned Conc. [ng/µl]	% Integrated Area	From [bp]	To [bp]	Peak Comment	Observations
100	8.50	8.50	-	65	145		Lower Marker
266	8.14	-	100.00	145	514		

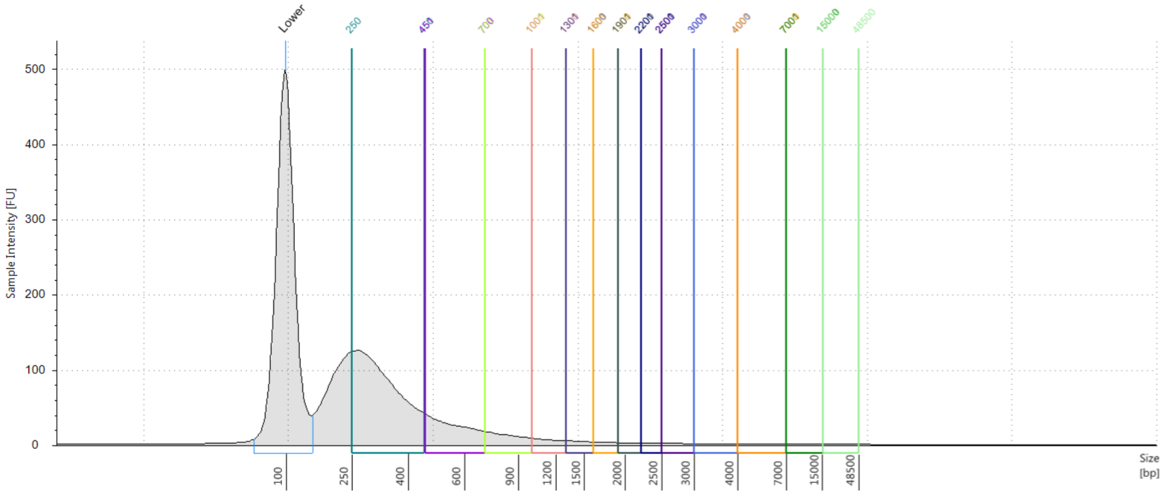

Region Table

From [bp]	To [bp]	Average Size [bp]	Conc. [ng/µl]	Region Molarity [nmol/l]	% of Total	Region Comment	Color
250	450	327	4.96	24.3	48.66		
451	700	555	1.31	3.81	12.86		
701	1000	835	0.481	0.945	4.72		
1001	1300	1148	0.183	0.271	1.79		
1301	1600	1454	0.0997	0.121	0.98		
1601	1900	1752	0.0683	0.0716	0.67		

 © Agilent Technologies, Inc. 2016

1901	2200	2066	0.0490	0.0452	0.48		
2201	2500	2363	0.0384	0.0317	0.38		
2501	3000	2751	0.0477	0.0361	0.47		
3001	4000	3482	0.0469	0.0306	0.46		
4001	7000	5376	0.0362	0.0180	0.35		
7001	15000	10662	0.0220	0.00608	0.22		
15001	48500	24708	0.0197	0.00264	0.19		

H1: Cat2: 3

1959 uralensis

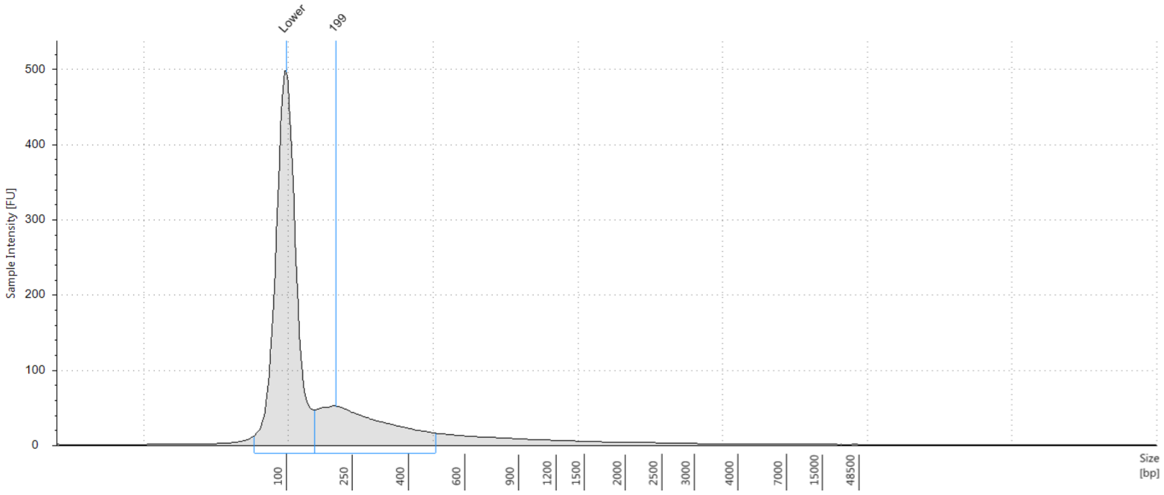

Sample Table

Well	DIN	Conc. [ng/µl]	Sample Description	Alert	Observations
H1	1.0	4.57	Cat2: 3	⚠	Sample concentration outside functional range for DIN

Peak Table

Size [bp]	Calibrated Conc. [ng/µl]	Assigned Conc. [ng/µl]	% Integrated Area	From [bp]	To [bp]	Peak Comment	Observations
100	8.50	8.50	-	64	148		Lower Marker
199	3.06	-	98.99	148	486		
-	-	-	-	-	-		Sample Well

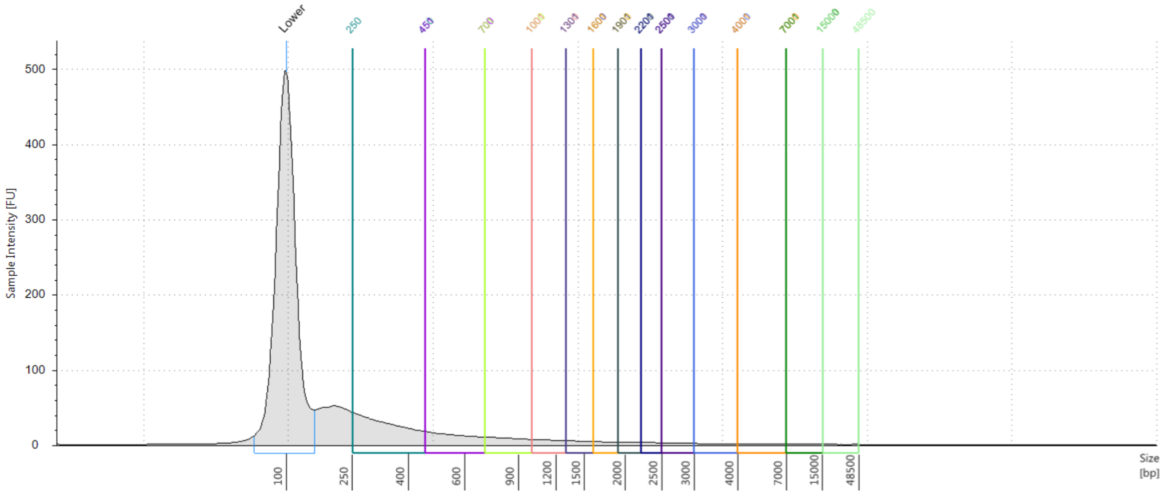

Region Table

From [bp]	To [bp]	Average Size [bp]	Conc. [ng/µl]	Region Molarity [nmol/l]	% of Total	Region Comment	Color
250	450	331	1.56	7.70	34.25		
451	700	558	0.593	1.75	12.98		
701	1000	842	0.298	0.593	6.53		
1001	1300	1148	0.165	0.245	3.62		
1301	1600	1460	0.106	0.127	2.32		

1601	1900	1762	0.0718	0.0729	1.57		
1901	2200	2066	0.0594	0.0524	1.30		
2201	2500	2365	0.0446	0.0353	0.98		
2501	3000	2748	0.0505	0.0367	1.11		
3001	4000	3480	0.0440	0.0289	0.96		
4001	7000	5388	0.0313	0.0158	0.69		
7001	15000	10840	0.0180	0.00519	0.39		
15001	48500	25141	0.0160	0.00227	0.35		

A2: Cat2: 4

1932 S. rigens

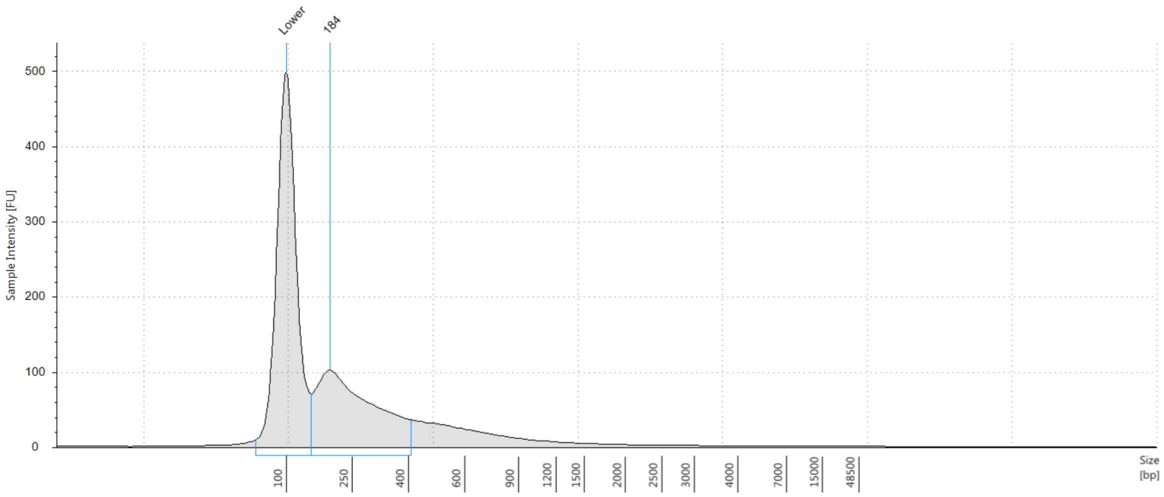

Sample Table

Well	DIN	Conc. [ng/µl]	Sample Description	Alert	Observations
A2	1.0	8.16	Cat2: 4	⚠	Sample concentration outside recommended range

Peak Table

Size [bp]	Calibrated Conc. [ng/µl]	Assigned Conc. [ng/µl]	% Integrated Area	From [bp]	To [bp]	Peak Comment	Observations
100	8.50	8.50	-	66	142		Lower Marker
184	5.01	-	100.00	142	408		

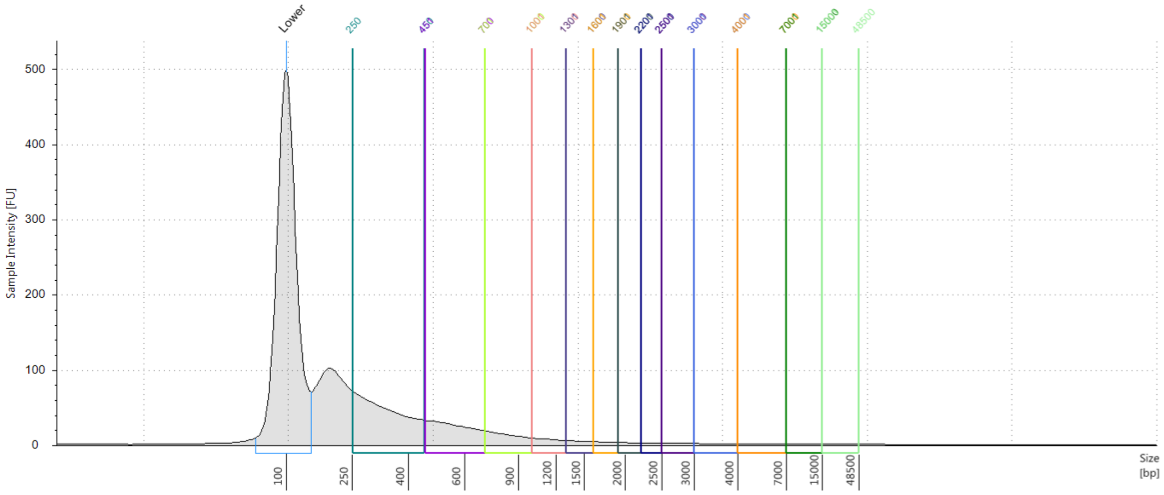

Region Table

From [bp]	To [bp]	Average Size [bp]	Conc. [ng/µl]	Region Molarity [nmol/l]	% of Total	Region Comment	Color
250	450	333	2.65	12.7	32.43		
451	700	559	1.19	3.36	14.56		
701	1000	830	0.492	0.938	6.03		
1001	1300	1139	0.203	0.283	2.49		
1301	1600	1451	0.114	0.126	1.40		
1601	1900	1756	0.0804	0.0737	0.99		

1901	2200	2068	0.0585	0.0459	0.72		
2201	2500	2362	0.0473	0.0326	0.58		
2501	3000	2750	0.0606	0.0364	0.74		
3001	4000	3492	0.0643	0.0311	0.79		
4001	7000	5416	0.0540	0.0176	0.66		
7001	15000	10767	0.0381	0.00651	0.47		
15001	48500	24447	0.0356	0.00278	0.44		

 © Agilent Technologies, Inc. 2016

B2: Cat3: 1

2017 S. sachalinensis

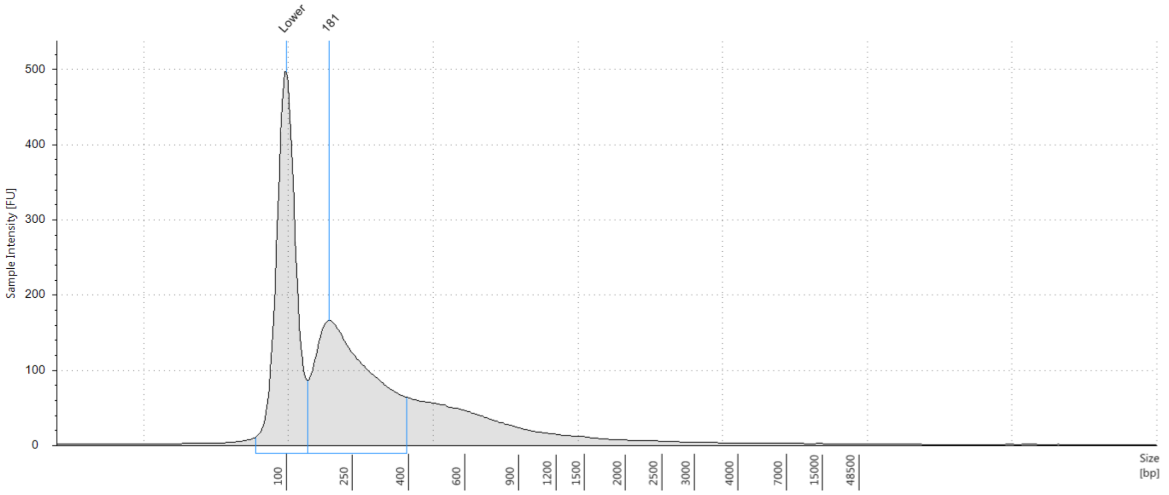

Sample Table

Well	DIN	Conc. [ng/µl]	Sample Description	Alert	Observations
B2	1.0	13.9	Cat3: 1		

Peak Table

Size [bp]	Calibrated Conc. [ng/µl]	Assigned Conc. [ng/µl]	% Integrated Area	From [bp]	To [bp]	Peak Comment	Observations
100	8.50	8.50	-	66	135		Lower Marker
181	8.51	-	100.00	135	396		

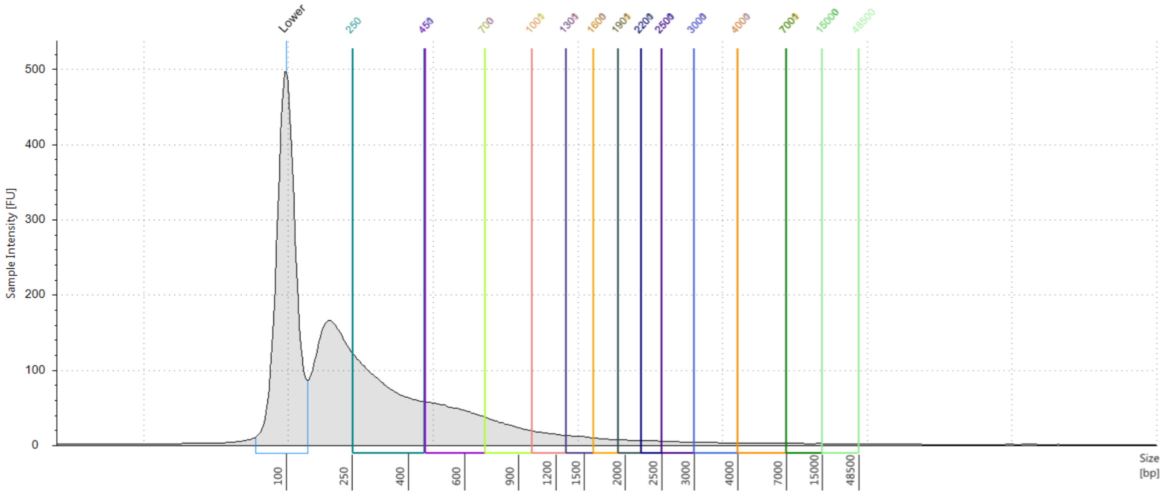

Region Table

From [bp]	To [bp]	Average Size [bp]	Conc. [ng/µl]	Region Molarity [nmol/l]	% of Total	Region Comment	Color
250	450	333	4.53	22.0	32.51		
451	700	562	2.22	6.34	15.95		
701	1000	831	0.975	1.90	7.00		
1001	1300	1141	0.405	0.587	2.90		
1301	1600	1451	0.238	0.277	1.70		
1601	1900	1755	0.152	0.151	1.09		

 © Agilent Technologies, Inc. 2016

1901	2200	2068	0.109	0.0946	0.78		
2201	2500	2361	0.0816	0.0635	0.59		
2501	3000	2749	0.0960	0.0681	0.69		
3001	4000	3487	0.0882	0.0543	0.63		
4001	7000	5392	0.0661	0.0302	0.47		
7001	15000	10740	0.0389	0.0102	0.28		
15001	48500	24556	0.0307	0.00398	0.22		

C2: Cat3: 2

2019 S. acaulis

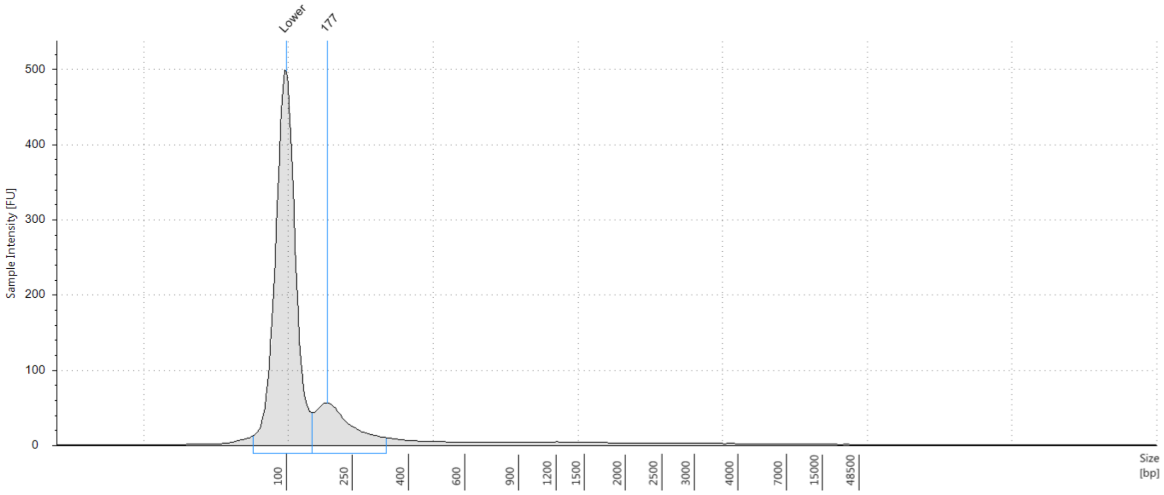

Sample Table

Well	DIN	Conc. [ng/µl]	Sample Description	Alert	Observations
C2	-	2.95	Cat3: 2	⚠	Sample concentration outside functional range for DIN

Peak Table

Size [bp]	Calibrated Conc. [ng/µl]	Assigned Conc. [ng/µl]	% Integrated Area	From [bp]	To [bp]	Peak Comment	Observations
100	8.50	8.50	-	63	143		Lower Marker
177	1.73	-	100.00	143	332		

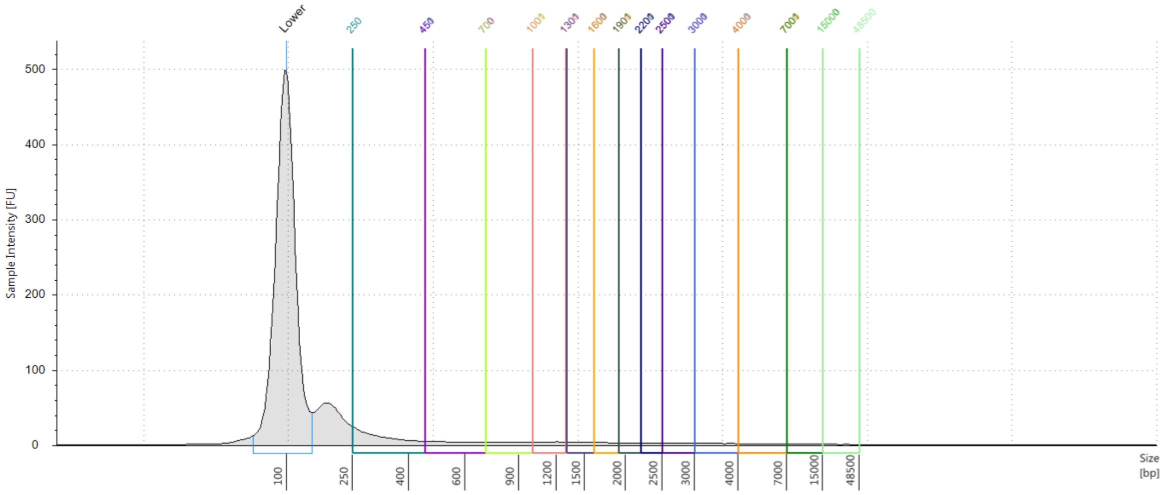

Region Table

From [bp]	To [bp]	Average Size [bp]	Conc. [ng/µl]	Region Molarity [nmol/l]	% of Total	Region Comment	Color
250	450	320	0.573	2.95	19.41		
451	700	565	0.174	0.527	5.88		
701	1000	851	0.130	0.263	4.41		
1001	1300	1153	0.103	0.151	3.50		
1301	1600	1457	0.0805	0.0938	2.73		

 © Agilent Technologies, Inc. 2016

1601	1900	1757	0.0589	0.0577	1.99		
1901	2200	2067	0.0501	0.0421	1.70		
2201	2500	2365	0.0410	0.0304	1.39		
2501	3000	2758	0.0581	0.0374	1.97		
3001	4000	3485	0.0677	0.0354	2.29		
4001	7000	5307	0.0510	0.0192	1.73		
7001	15000	10593	0.0260	0.00551	0.88		
15001	48500	23917	0.0223	0.00227	0.76		

D2: Cat3: 3

2019 S. burchellii

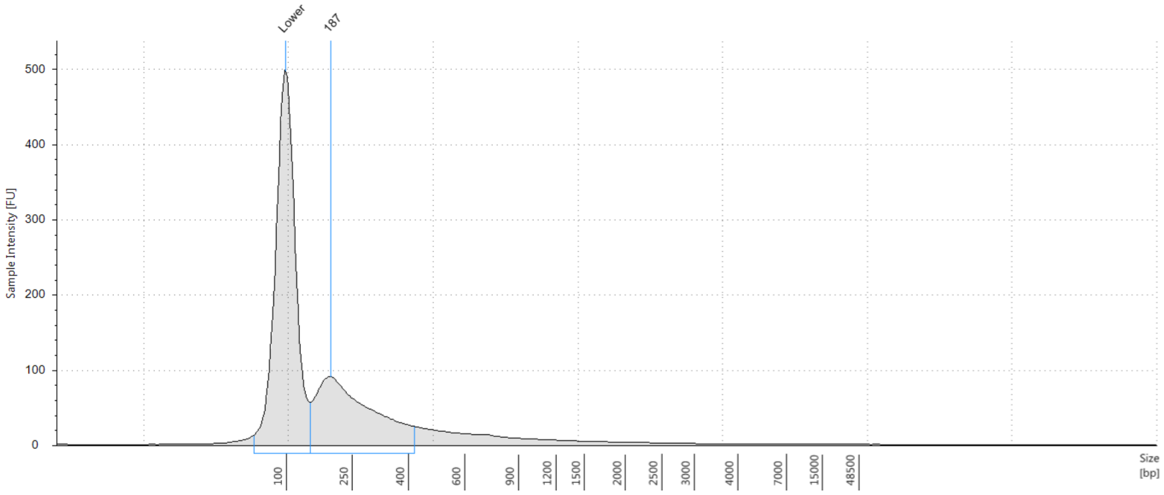

Sample Table

Well	DIN	Conc. [ng/µl]	Sample Description	Alert	Observations
D2	1.0	6.28	Cat3: 3	⚠	Sample concentration outside recommended range

Peak Table

Size [bp]	Calibrated Conc. [ng/µl]	Assigned Conc. [ng/µl]	% Integrated Area	From [bp]	To [bp]	Peak Comment	Observations
100	8.50	8.50	-	64	140		Lower Marker
187	4.28	-	99.86	140	419		
-	-	-	-	-	-		Sample Well

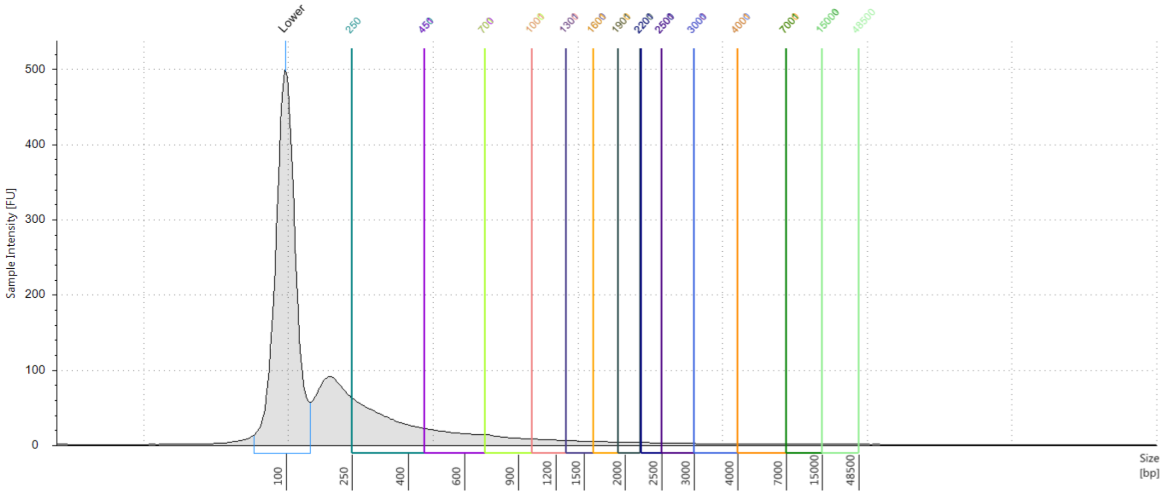

Region Table

From [bp]	To [bp]	Average Size [bp]	Conc. [ng/µl]	Region Molarity [nmol/l]	% of Total	Region Comment	Color
250	450	329	2.05	10.1	32.62		
451	700	559	0.752	2.20	11.98		
701	1000	834	0.353	0.707	5.63		
1001	1300	1151	0.168	0.250	2.68		
1301	1600	1455	0.103	0.125	1.64		

 © Agilent Technologies, Inc. 2016

1601	1900	1757	0.0780	0.0806	1.24		
1901	2200	2067	0.0535	0.0485	0.85		
2201	2500	2355	0.0428	0.0351	0.68		
2501	3000	2755	0.0488	0.0372	0.78		
3001	4000	3486	0.0441	0.0295	0.70		
4001	7000	5340	0.0314	0.0169	0.50		
7001	15000	10618	0.0162	0.00536	0.26		
15001	48500	24207	0.0153	0.00238	0.24		

 © Agilent Technologies, Inc. 2016

E2: Cat3: 4

2018 S. noctiflora

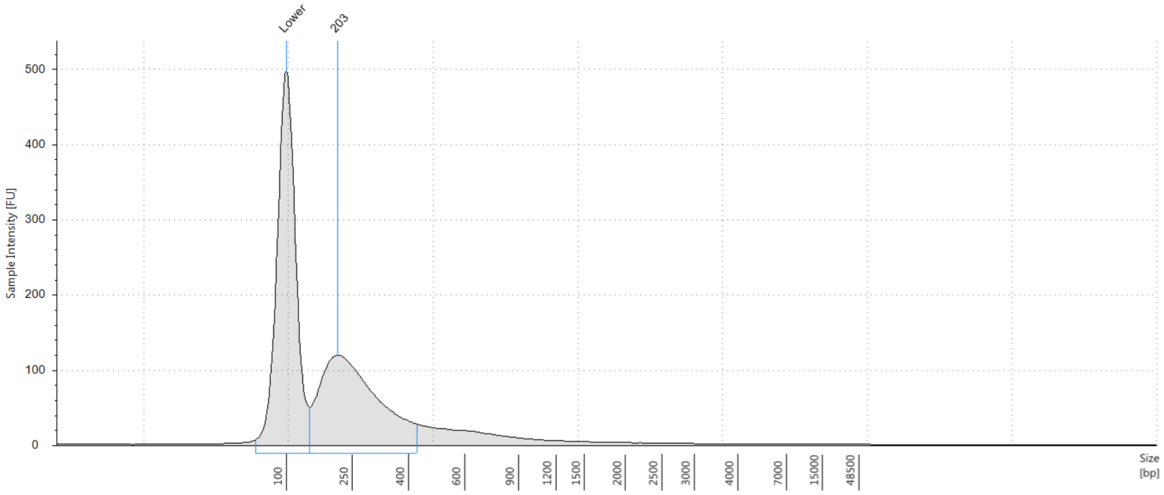

Sample Table

Well	DIN	Conc. [ng/µl]	Sample Description	Alert	Observations
E2	1.0	8.51	Cat3: 4	⚠	Sample concentration outside recommended range

Peak Table

Size [bp]	Calibrated Conc. [ng/µl]	Assigned Conc. [ng/µl]	% Integrated Area	From [bp]	To [bp]	Peak Comment	Observations
100	8.50	8.50	-	65	138		Lower Marker
203	6.31	-	99.82	138	424		
-	-	-	-	-	-		Sample Well

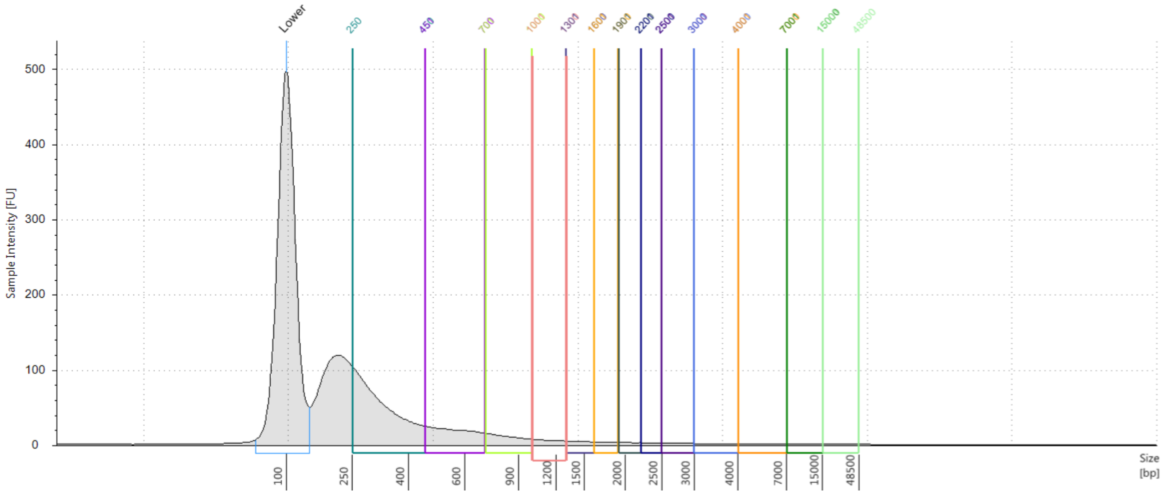

Region Table

From [bp]	To [bp]	Average Size [bp]	Conc. [ng/µl]	Region Molarity [nmol/l]	% of Total	Region Comment	Color
250	450	321	3.16	15.9	37.15		
451	700	561	0.960	2.80	11.29		
701	1000	830	0.416	0.843	4.89		
1001	1300	1149	0.161	0.250	1.90		
1301	1600	1458	0.0932	0.118	1.10		

1601	1900	1759	0.0727	0.0790	0.85		
1901	2200	2066	0.0495	0.0479	0.58		
2201	2500	2361	0.0435	0.0380	0.51		
2501	3000	2756	0.0456	0.0376	0.54		
3001	4000	3483	0.0432	0.0322	0.51		
4001	7000	5379	0.0317	0.0190	0.37		
7001	15000	10812	0.0201	0.00681	0.24		
15001	48500	24759	0.0159	0.00274	0.19		

F2: Ladder

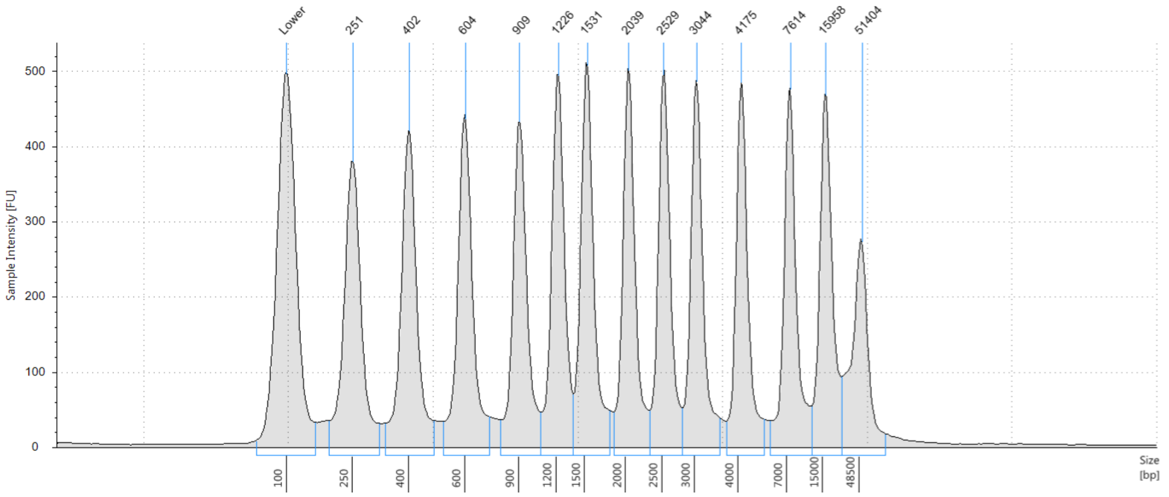

Sample Table

Well	DIN	Conc. [ng/µl]	Sample Description	Alert	Observations
F2	-	70.8	Ladder		Ladder run as sample

Peak Table

Size [bp]	Calibrated Conc. [ng/µl]	Assigned Conc. [ng/µl]	% Integrated Area	From [bp]	To [bp]	Peak Comment	Observations
100	8.50	8.50	-	66	149		Lower Marker
251	5.69	-	8.18	181	313		
402	5.75	-	8.25	329	482		
604	5.73	-	8.24	513	724		
909	5.32	-	7.65	788	1071		
1226	5.69	-	8.17	1071	1378		
1531	5.90	-	8.47	1378	1792		
2039	5.51	-	7.91	1840	2325		
2529	5.37	-	7.71	2325	2811		
3044	5.26	-	7.55	2811	3562		
4175	5.07	-	7.28	3712	5413		
7614	5.20	-	7.47	5818	11933		
15958	5.25	-	7.54	11933	22504		
51404	3.90	-	5.60	22504	>60000		

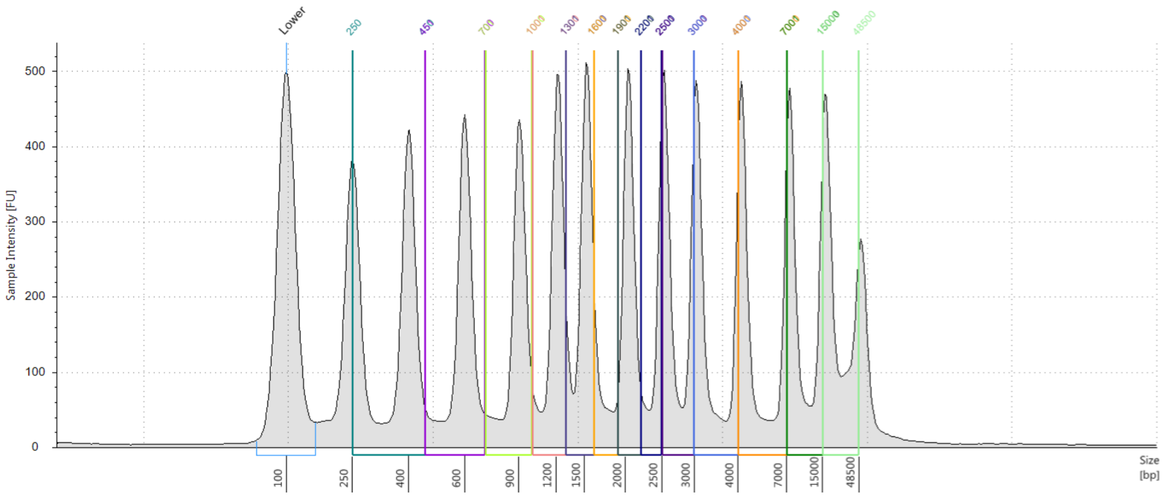

Region Table

From [bp]	To [bp]	Average Size [bp]	Conc. [ng/µl]	Region Molarity [nmol/l]	% of Total	Region Comment	Color
250	450	355	8.71	40.8	12.29		
451	700	596	6.09	16.4	8.60		
701	1000	898	5.38	9.54	7.60		
1001	1300	1209	5.57	7.25	7.86		
1301	1600	1507	5.59	5.83	7.89		
1601	1900	1739	1.37	1.29	1.93		
1901	2200	2050	4.99	3.80	7.04		
2201	2500	2434	2.45	1.59	3.46		
2501	3000	2741	5.76	3.33	8.13		
3001	4000	3431	5.32	2.50	7.51		
4001	7000	5108	5.50	1.80	7.77		
7001	15000	10106	5.70	0.972	8.05		
15001	48500	23334	5.85	0.454	8.27		

 © Agilent Technologies, Inc. 2016

Calibration

Molecular Weight Settings

Fitting type: Genomic DNA Sizing
Alignment type: From lower marker

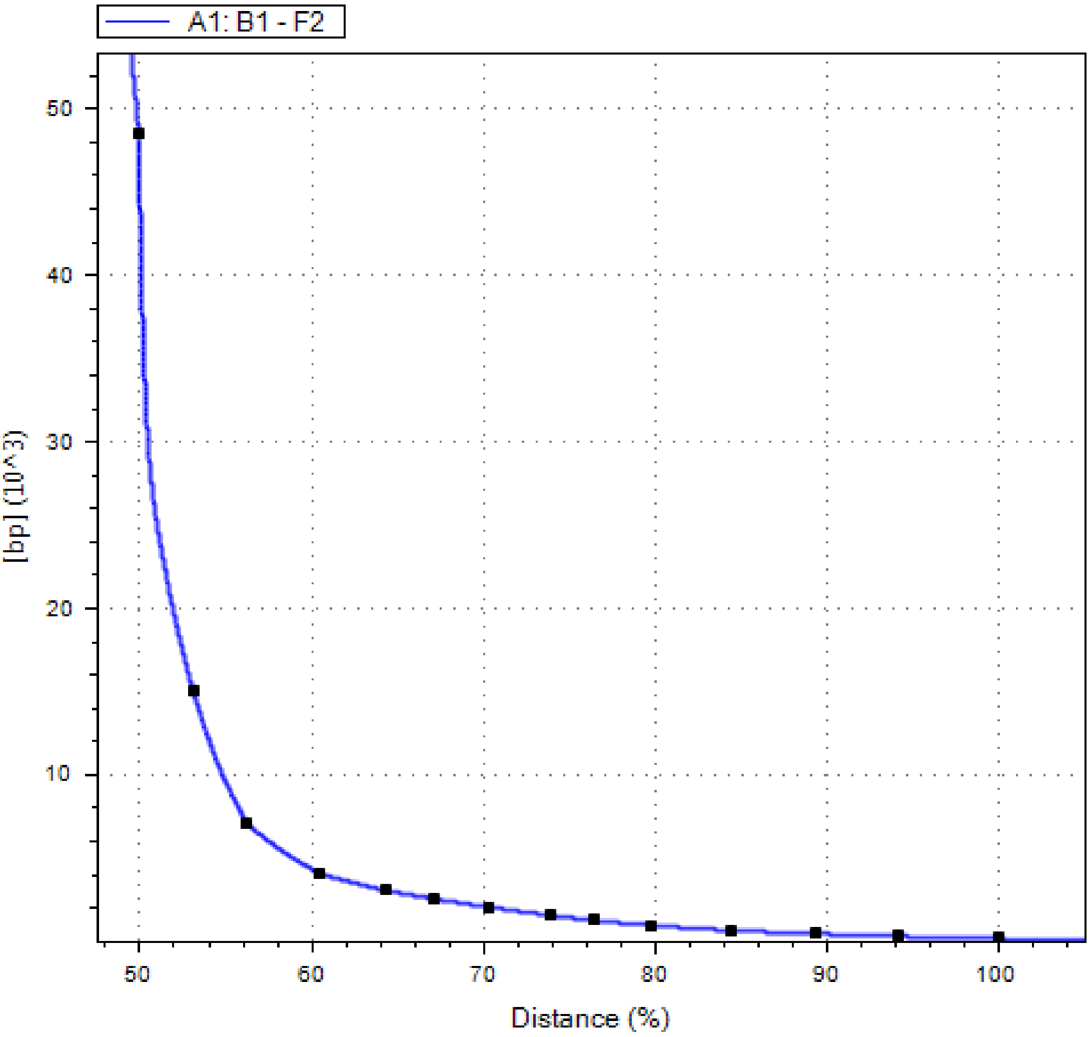

Concentration Settings

Calibration mode: Lower Marker
Normalise peaks from: Lower Marker
Fitting type: Linear Regression

 © Agilent Technologies, Inc. 2016

Experiment Properties

Run Properties

**Analysis Software Version**	2.1.38.8716
**Filename**	C:\Users\admin\Desktop\AnneSophie\2019-september-october-herbarium\after_PCRbarcoding.gDNA
**Assay**	Genomic DNA
**Run End Date**	07-Oct-2019 12:24 PM
**Last Saved Under Version**	2.1.38.8716
**DIN Version**	2.1.38.8716
**Study**	
**Comments**	

ScreenTape Device 1

**Username**	admin
**ScreenTape Device ID**	01-S025-190812-01-000054
**Expiry Date**	21-Oct-2019
**ScreenTape Device History**	First run 07-Oct-2019, 1 run performed
**Temperature [°C]**	22.5
**Electrophoresis Time [s]**	229
**Instrument Type**	6655
**Instrument Serial Number**	03-PM405
**Notes**	
**ScreenTape Device Run Date**	07-Oct-2019 12:02 PM

Controller Environment

**Computer**	LAB3210150
**Instrument Controller Software Version**	A.02.01 SR1
**First Run Analysis Version**	2.1.38.8716
**Operating System**	Microsoft Windows 7 Enterprise
