# Supplementary material for: Retrieval of long DNA reads from herbarium specimens
Source: AoB Plants. 2023 Nov 8;15(6):plad074. doi: 10.1093/aobpla/plad074 (PMC10735254; doi:10.1093/aobpla/plad074)
Supplement: plad074_suppl_Supplementary_Appendix_S1_4 [file plad074_suppl_supplementary_appendix_s1_4.pdf]

Filename: 2019-10-07-01_after_0.4_size_selection.gDNA

Gel Image

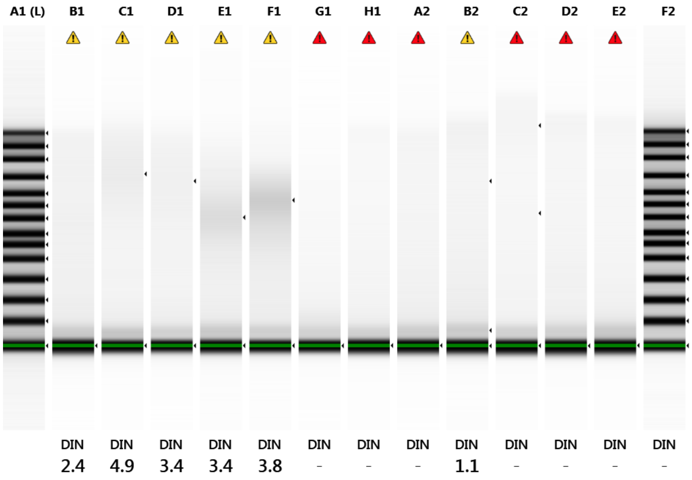

Default image (Contrast 100%)

Sample Info

Well	DIN	Conc. [ng/µl]	Sample Description	Alert	Observations
A1	-	68.8	Ladder		Ladder
B1	2.4	3.37	Cat1: 1		Sample concentration outside functional range for DIN
C1	4.9	3.68	Cat1: 2		Sample concentration outside functional range for DIN
D1	3.4	3.50	Cat1: 3		Sample concentration outside functional range for DIN
E1	3.4	5.12	Cat1: 4		Sample concentration outside recommended range
F1	3.8	6.18	Cat2: 1		Sample concentration outside recommended range
G1	-	2.06	Cat2: 2		Sample concentration outside functional range for DIN
H1	-	2.18	Cat2: 3		Sample concentration outside functional range for DIN
A2	-	2.90	Cat2: 4		Sample concentration outside functional range for DIN
B2	1.1	3.35	Cat3: 1		Sample concentration outside functional range for DIN
C2	-	2.81	Cat3: 2		Sample concentration outside functional range for DIN
D2	-	2.80	Cat3: 3		Sample concentration outside functional range for DIN
E2	-	2.84	Cat3: 4		Sample concentration outside functional range for DIN
F2	-	78.0	Ladder		Ladder run as sample

 © Agilent Technologies, Inc. 2016

A1: Ladder

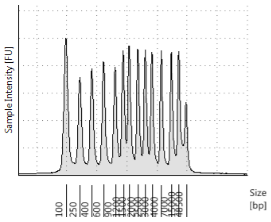

B1: Cat1: 1

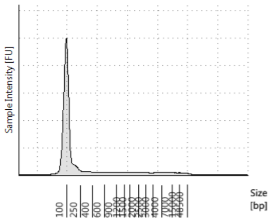

C1: Cat1: 2

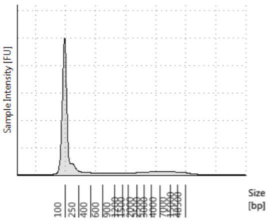

D1: Cat1: 3

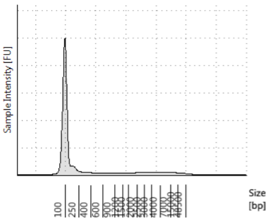

E1: Cat1: 4

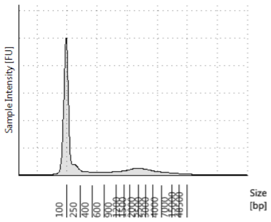

F1: Cat2: 1

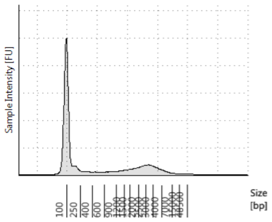

G1: Cat2: 2

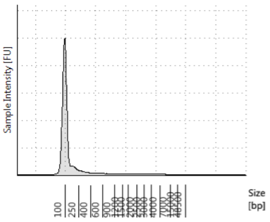

H1: Cat2: 3

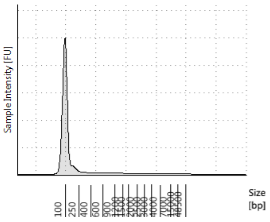

A2: Cat2: 4

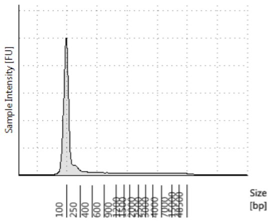

B2: Cat3: 1

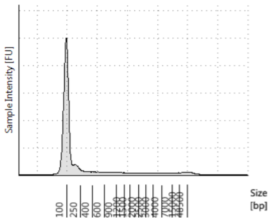

C2: Cat3: 2

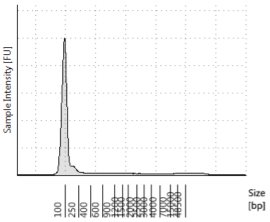

D2: Cat3: 3

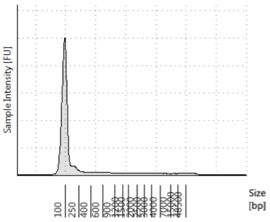

E2: Cat3: 4

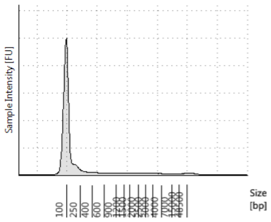

F2: Ladder

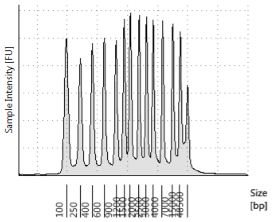

 © Agilent Technologies, Inc. 2016

A1: Ladder

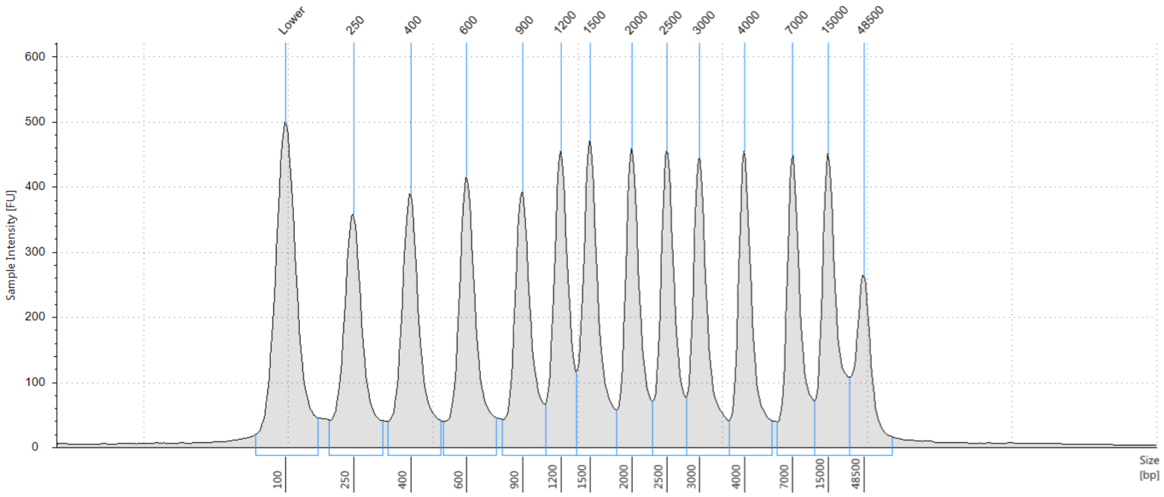

Sample Table

Well	DIN	Conc. [ng/µl]	Sample Description	Alert	Observations
A1	-	68.8	Ladder		Ladder

Peak Table

Size [bp]	Calibrated Conc. [ng/µl]	Assigned Conc. [ng/µl]	% Integrated Area	From [bp]	To [bp]	Peak Comment	Observations
100	8.50	8.50	-	66	154		Lower Marker
250	5.47	-	8.04	180	318		
400	5.66	-	8.33	331	499		
600	5.78	-	8.50	508	742		
900	5.19	-	7.63	777	1070		
1200	5.36	-	7.88	1070	1348		
1500	6.01	-	8.84	1348	1801		
2000	5.36	-	7.89	1801	2283		
2500	5.25	-	7.72	2283	2797		
3000	5.20	-	7.66	2797	3624		
4000	5.07	-	7.46	3624	5531		
7000	4.94	-	7.27	5866	11119		
15000	5.40	-	7.94	11119	23855		
48500	3.30	-	4.85	23855	>60000		

 © Agilent Technologies, Inc. 2016

B1: Cat1: 1 1994 acaulis

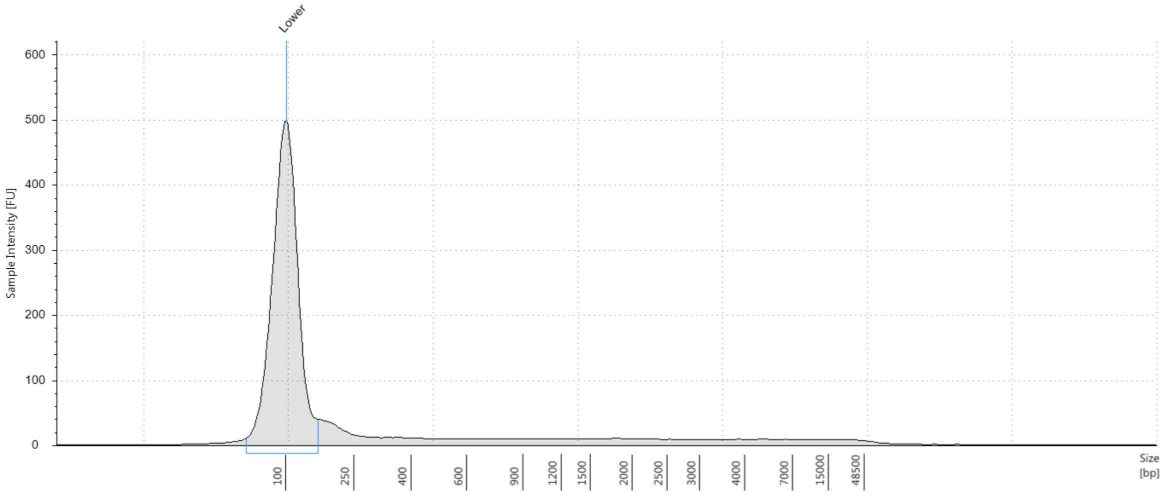

Sample Table

Well	DIN	Conc. [ng/µl]	Sample Description	Alert	Observations
B1	2.4	3.37	Cat1: 1	⚠	Sample concentration outside functional range for DIN

Peak Table

Size [bp]	Calibrated Conc. [ng/µl]	Assigned Conc. [ng/µl]	% Integrated Area	From [bp]	To [bp]	Peak Comment	Observations
100	8.50	8.50	-	58	155		Lower Marker
-	-	-	-	-	-		Sample Well

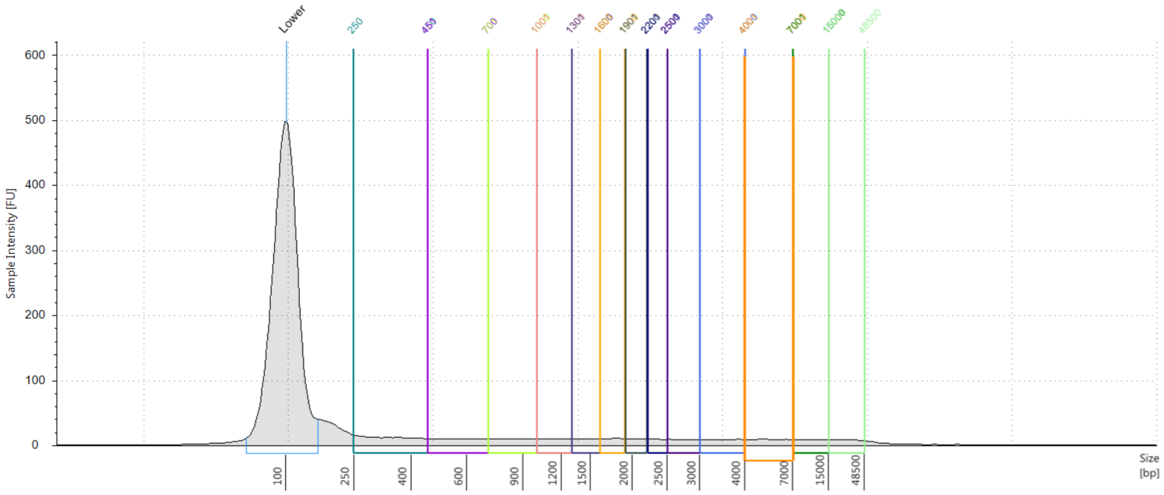

Region Table

From [bp]	To [bp]	Average Size [bp]	Conc. [ng/µl]	Region Molarity [nmol/l]	% of Total	Region Comment	Color
250	450	341	0.491	2.48	14.57		
451	700	572	0.326	0.985	9.69		
701	1000	846	0.254	0.518	7.56		
1001	1300	1152	0.180	0.268	5.35		
1301	1600	1459	0.149	0.174	4.41		

 © Agilent Technologies, Inc. 2016

1601	1900	1762	0.145	0.139	4.29		
1901	2200	2066	0.118	0.0973	3.51		
2201	2500	2359	0.0999	0.0722	2.97		
2501	3000	2760	0.150	0.0939	4.47		
3001	4000	3504	0.213	0.105	6.32		
4001	7000	5443	0.235	0.0764	6.99		
7001	15000	10837	0.168	0.0283	4.99		
15001	48500	24256	0.153	0.0120	4.56		

C1: Cat1: 2 1987 burchellii

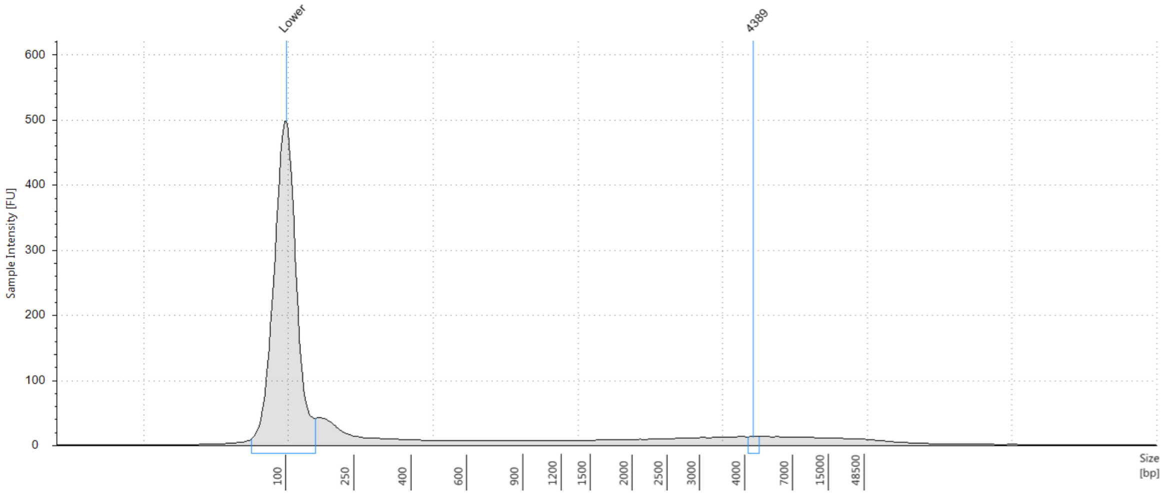

Sample Table

Well	DIN	Conc. [ng/µl]	Sample Description	Alert	Observations
C1	4.9	3.68	Cat1: 2	⚠	Sample concentration outside functional range for DIN

Peak Table

Size [bp]	Calibrated Conc. [ng/µl]	Assigned Conc. [ng/µl]	% Integrated Area	From [bp]	To [bp]	Peak Comment	Observations
100	8.50	8.50	-	62	148		Lower Marker
4389	0.0977	-	80.46	4141	4719		
-	-	-	-	-	-		Sample Well

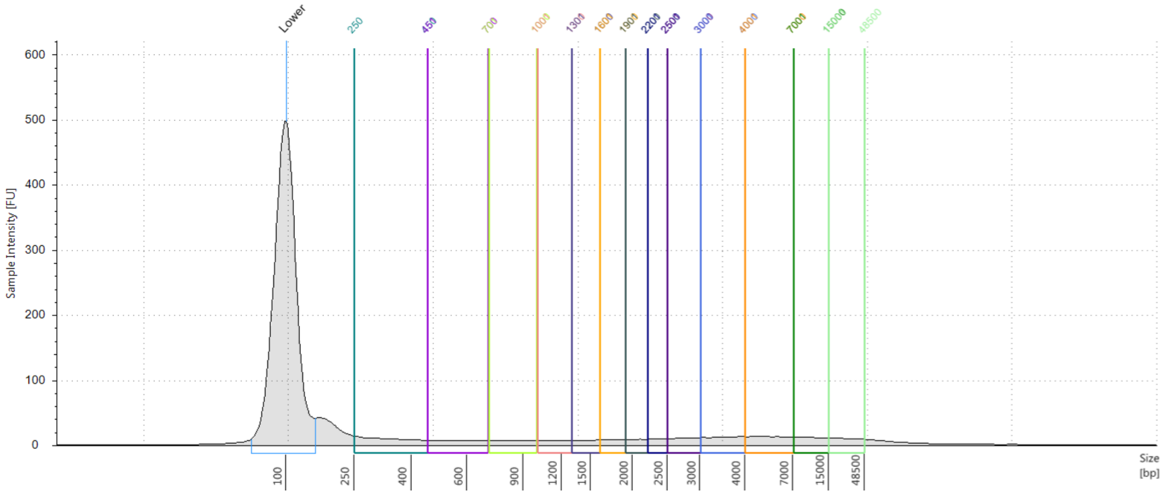

Region Table

From [bp]	To [bp]	Average Size [bp]	Conc. [ng/µl]	Region Molarity [nmol/l]	% of Total	Region Comment	Color
250	450	336	0.416	2.36	11.32		
451	700	572	0.251	0.875	6.83		
701	1000	849	0.191	0.451	5.21		
1001	1300	1155	0.137	0.234	3.73		
1301	1600	1463	0.123	0.164	3.36		

 © Agilent Technologies, Inc. 2016

1601	1900	1766	0.117	0.126	3.17		
1901	2200	2065	0.110	0.0997	2.98		
2201	2500	2370	0.109	0.0847	2.97		
2501	3000	2771	0.219	0.143	5.94		
3001	4000	3520	0.327	0.166	8.90		
4001	7000	5441	0.382	0.128	10.40		
7001	15000	10715	0.247	0.0435	6.70		
15001	48500	24788	0.214	0.0176	5.82		

© Agilent Technologies, Inc. 2016

D1: Cat1: 3 1981noctiflora

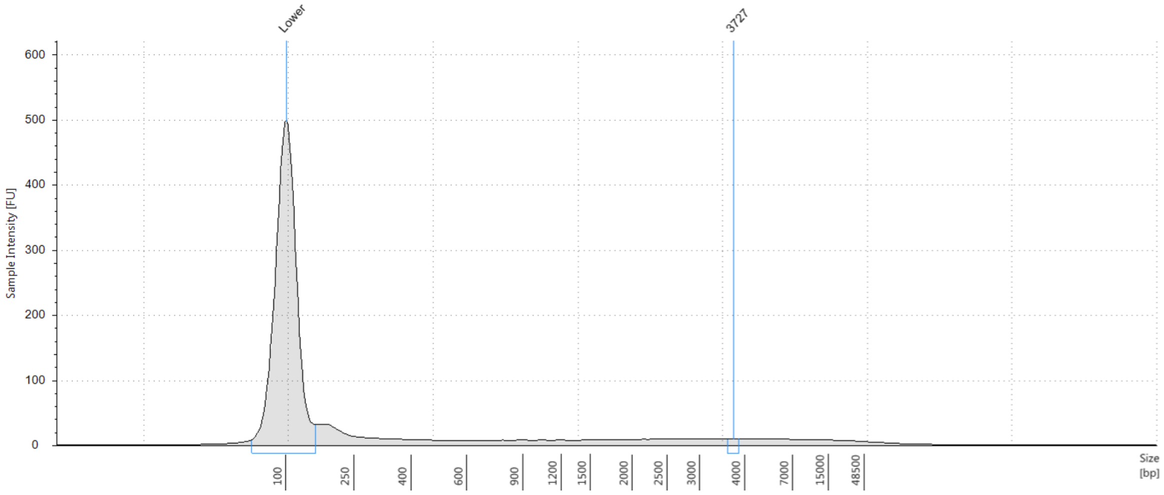

Sample Table

Well	DIN	Conc. [ng/µl]	Sample Description	Alert	Observations
D1	3.4	3.50	Cat1: 3	⚠	Sample concentration outside functional range for DIN

Peak Table

Size [bp]	Calibrated Conc. [ng/µl]	Assigned Conc. [ng/µl]	% Integrated Area	From [bp]	To [bp]	Peak Comment	Observations
100	8.50	8.50	-	63	149		Lower Marker
3727	0.0796	-	90.81	3577	3851		
-	-	-	-	-	-		Sample Well

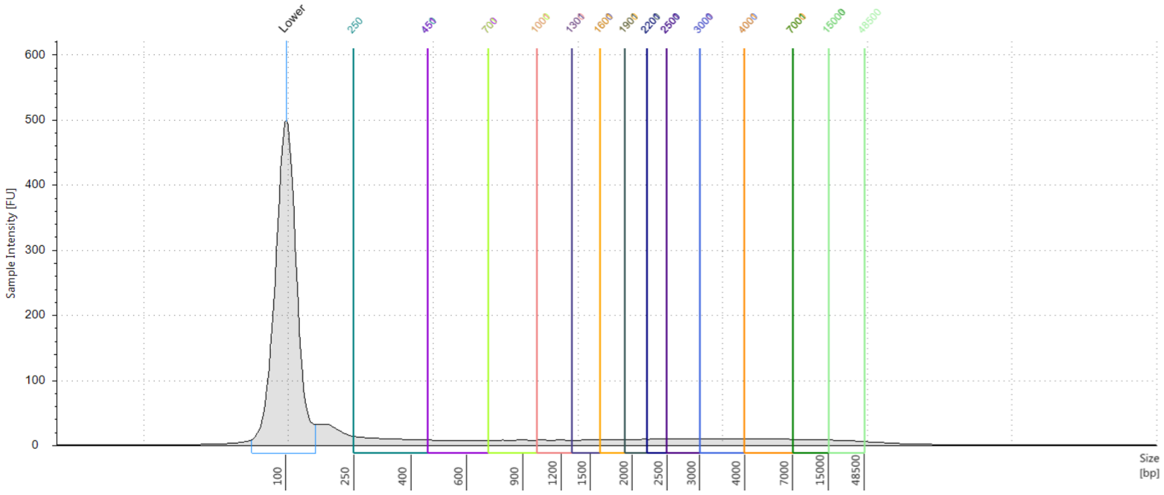

Region Table

From [bp]	To [bp]	Average Size [bp]	Conc. [ng/µl]	Region Molarity [nmol/l]	% of Total	Region Comment	Color
250	450	337	0.467	2.47	13.36		
451	700	570	0.281	0.901	8.02		
701	1000	847	0.233	0.497	6.66		
1001	1300	1150	0.169	0.264	4.84		
1301	1600	1469	0.140	0.171	4.01		

 © Agilent Technologies, Inc. 2016

1601	1900	1763	0.127	0.128	3.64		
1901	2200	2067	0.133	0.113	3.81		
2201	2500	2368	0.119	0.0871	3.40		
2501	3000	2758	0.208	0.131	5.94		
3001	4000	3504	0.282	0.140	8.05		
4001	7000	5416	0.292	0.0966	8.35		
7001	15000	10690	0.196	0.0342	5.61		
15001	48500	24150	0.154	0.0128	4.41		

 © Agilent Technologies, Inc. 2016

E1: Cat1: 4 1979 S. involucrata

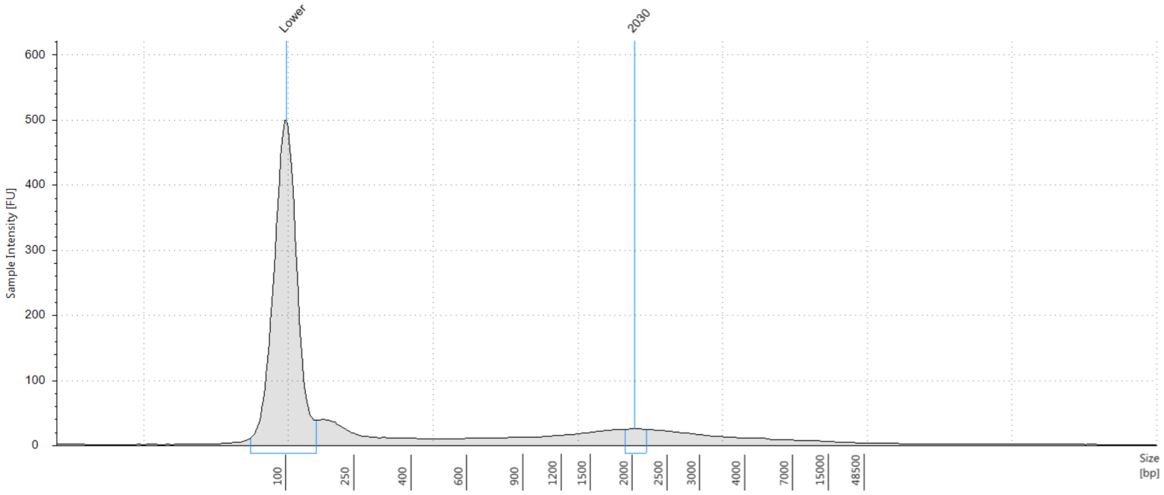

Sample Table

Well	DIN	Conc. [ng/µl]	Sample Description	Alert	Observations
E1	3.4	5.12	Cat1: 4	⚠	Sample concentration outside recommended range

Peak Table

Size [bp]	Calibrated Conc. [ng/µl]	Assigned Conc. [ng/µl]	% Integrated Area	From [bp]	To [bp]	Peak Comment	Observations
100	8.50	8.50	-	61	150		Lower Marker
2030	0.342	-	88.08	1898	2184		
-	-	-	-	-	-		Sample Well

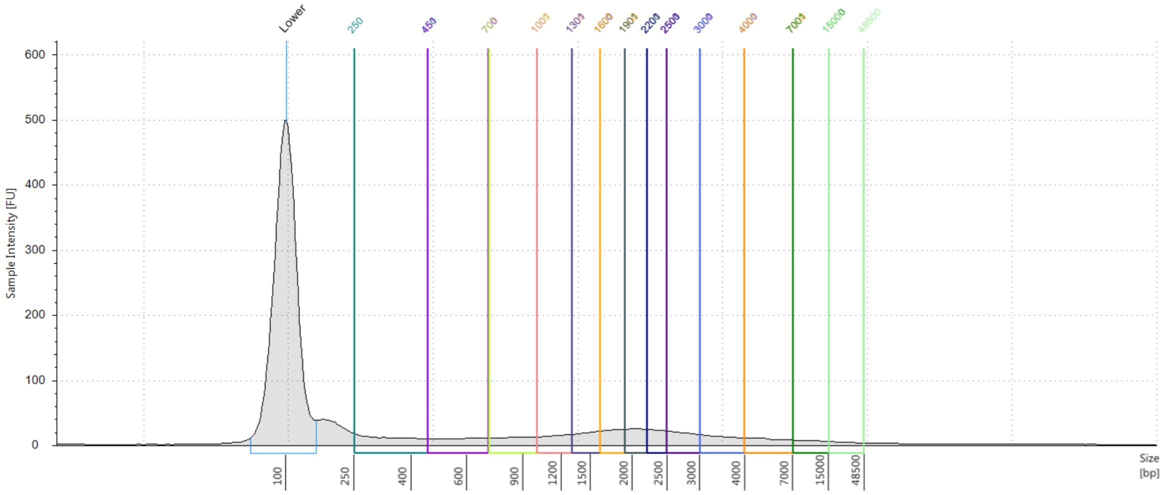

Region Table

From [bp]	To [bp]	Average Size [bp]	Conc. [ng/µl]	Region Molarity [nmol/l]	% of Total	Region Comment	Color
250	450	339	0.519	2.62	10.14		
451	700	575	0.369	1.10	7.21		
701	1000	851	0.356	0.701	6.96		
1001	1300	1160	0.320	0.453	6.25		
1301	1600	1471	0.350	0.384	6.83		

 © Agilent Technologies, Inc. 2016

1601	1900	1769	0.377	0.342	7.38		
1901	2200	2064	0.342	0.265	6.68		
2201	2500	2358	0.301	0.204	5.88		
2501	3000	2751	0.404	0.238	7.90		
3001	4000	3476	0.357	0.170	6.98		
4001	7000	5333	0.278	0.0907	5.44		
7001	15000	10623	0.145	0.0254	2.84		
15001	48500	23948	0.0852	0.00735	1.66		

F1: Cat2: 1 1969 acaulis

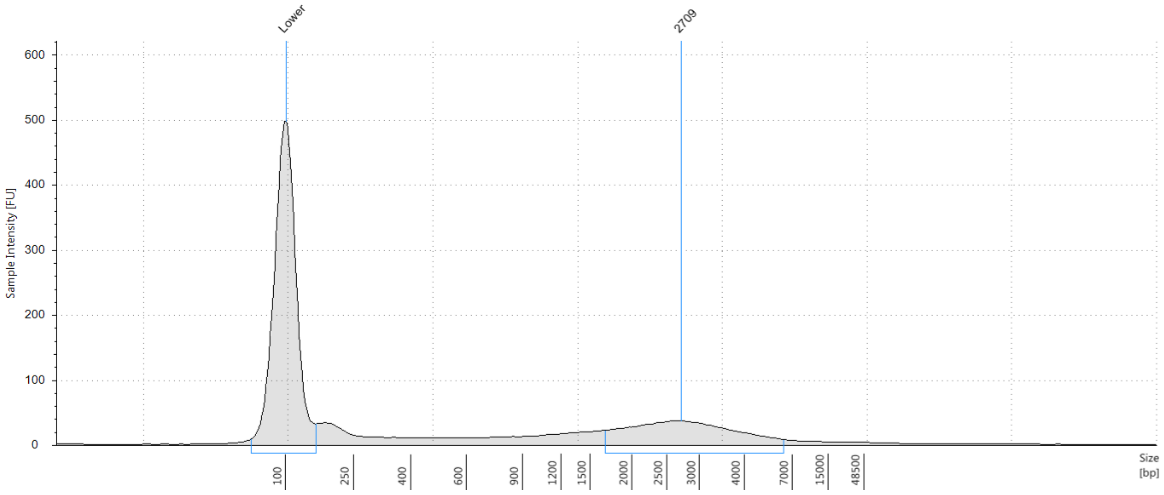

Sample Table

Well	DIN	Conc. [ng/µl]	Sample Description	Alert	Observations
F1	3.8	6.18	Cat2: 1	⚠	Sample concentration outside recommended range

Peak Table

Size [bp]	Calibrated Conc. [ng/µl]	Assigned Conc. [ng/µl]	% Integrated Area	From [bp]	To [bp]	Peak Comment	Observations
100	8.50	8.50	-	62	150		Lower Marker
2709	3.02	-	98.99	1661	6290		
-	-	-	-	-	-		Sample Well

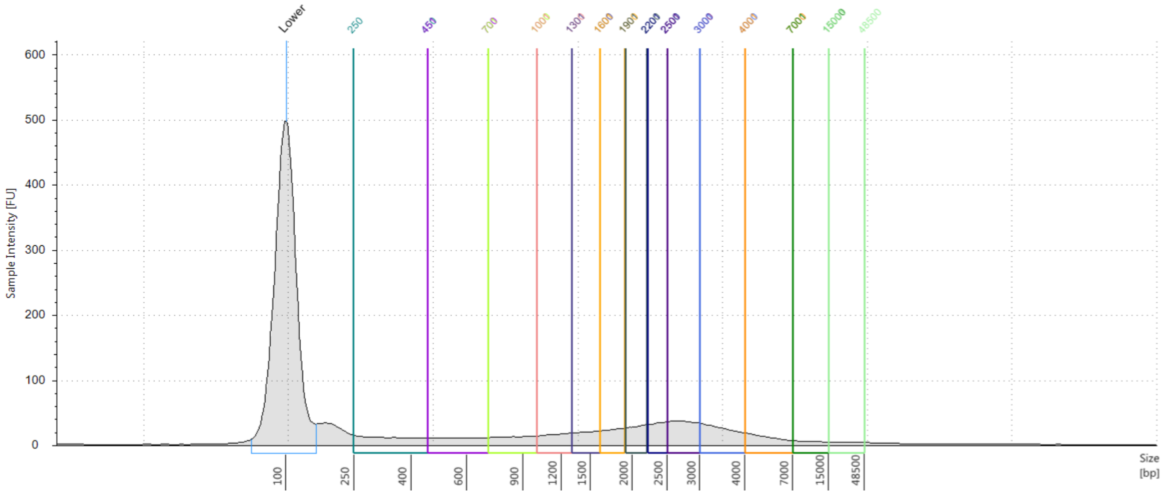

Region Table

From [bp]	To [bp]	Average Size [bp]	Conc. [ng/µl]	Region Molarity [nmol/l]	% of Total	Region Comment	Color
250	450	344	0.543	2.81	8.79		
451	700	574	0.417	1.28	6.75		
701	1000	856	0.395	0.797	6.40		
1001	1300	1159	0.360	0.521	5.83		
1301	1600	1468	0.371	0.417	6.00		

1601	1900	1762	0.406	0.375	6.57		
1901	2200	2068	0.445	0.347	7.20		
2201	2500	2371	0.469	0.317	7.59		
2501	3000	2755	0.794	0.461	12.85		
3001	4000	3449	0.772	0.366	12.50		
4001	7000	5179	0.368	0.126	5.97		
7001	15000	10671	0.112	0.0221	1.82		
15001	48500	24476	0.0771	0.00747	1.25		

© Agilent Technologies, Inc. 2016

G1: Cat2: 2 1948 burchellii

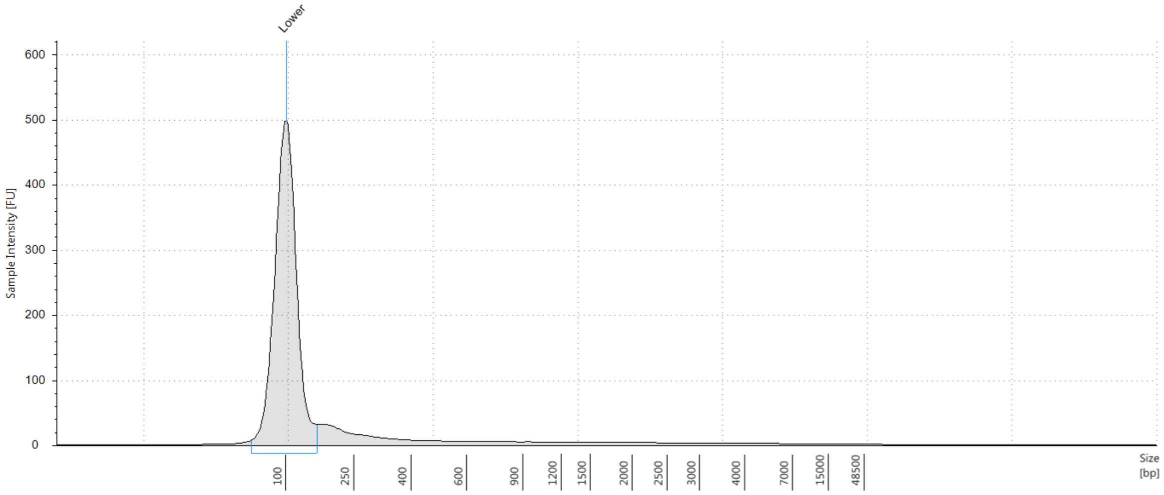

Sample Table

Well	DIN	Conc. [ng/µl]	Sample Description	Alert	Observations
G1	-	2.06	Cat2: 2	⚠	Sample concentration outside functional range for DIN

Peak Table

Size [bp]	Calibrated Conc. [ng/µl]	Assigned Conc. [ng/µl]	% Integrated Area	From [bp]	To [bp]	Peak Comment	Observations
100	8.50	8.50	-	63	152		Lower Marker

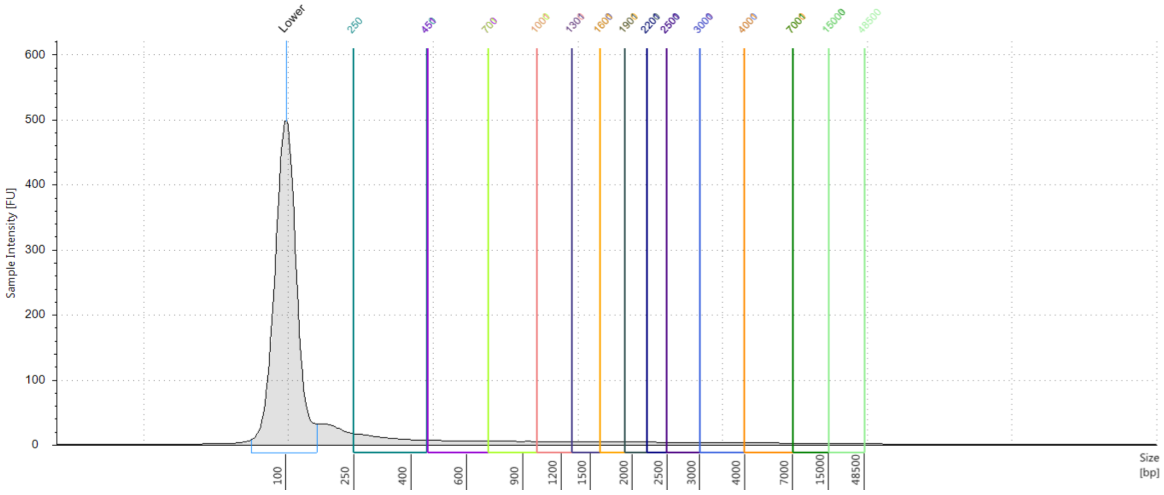

Region Table

From [bp]	To [bp]	Average Size [bp]	Conc. [ng/µl]	Region Molarity [nmol/l]	% of Total	Region Comment	Color
250	450	330	0.502	2.69	24.33		
451	700	567	0.216	0.721	10.47		
701	1000	842	0.146	0.337	7.08		
1001	1300	1155	0.0954	0.164	4.62		
1301	1600	1467	0.0734	0.0994	3.56		
1601	1900	1762	0.0592	0.0676	2.87		

 © Agilent Technologies, Inc. 2016

1901	2200	2060	0.0493	0.0490	2.39		
2201	2500	2371	0.0408	0.0358	1.98		
2501	3000	2765	0.0646	0.0498	3.13		
3001	4000	3497	0.0744	0.0473	3.61		
4001	7000	5348	0.0615	0.0286	2.98		
7001	15000	10567	0.0258	0.00808	1.25		
15001	48500	24597	0.0179	0.00307	0.87		

H1: Cat2: 3 1959 uralensis

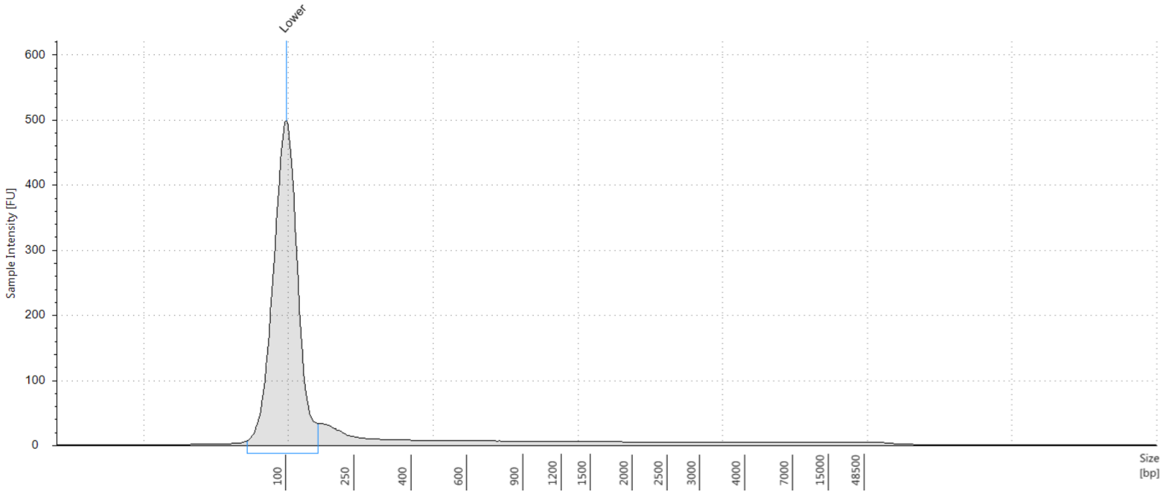

Sample Table

Well	DIN	Conc. [ng/µl]	Sample Description	Alert	Observations
H1	-	2.18	Cat2: 3	⚠	Sample concentration outside functional range for DIN

Peak Table

Size [bp]	Calibrated Conc. [ng/µl]	Assigned Conc. [ng/µl]	% Integrated Area	From [bp]	To [bp]	Peak Comment	Observations
100	8.50	8.50	-	59	153		Lower Marker
-	-	-	-	-	-		Sample Well

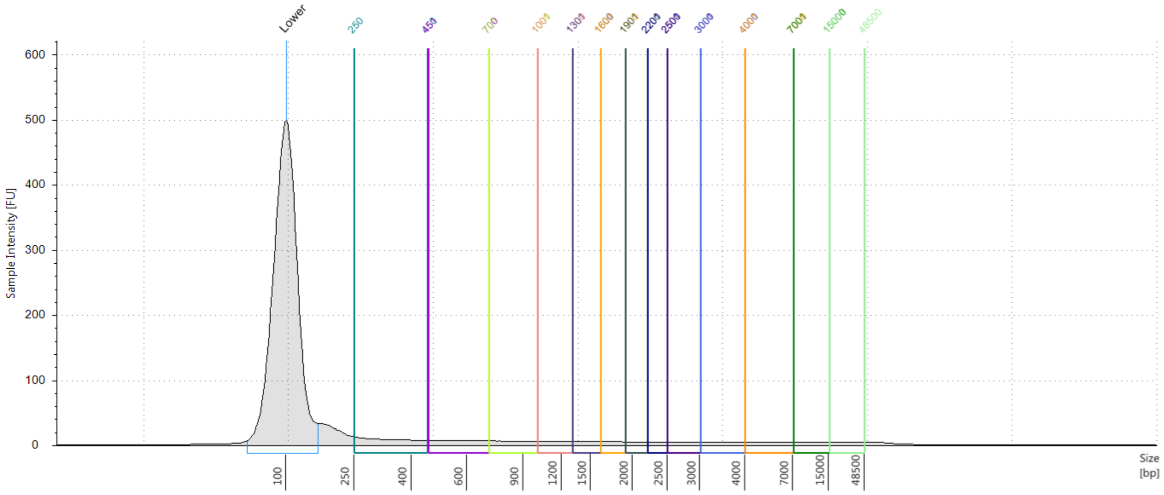

Region Table

From [bp]	To [bp]	Average Size [bp]	Conc. [ng/µl]	Region Molarity [nmol/l]	% of Total	Region Comment	Color
250	450	339	0.370	1.94	16.95		
451	700	568	0.233	0.740	10.69		
701	1000	844	0.169	0.363	7.71		
1001	1300	1151	0.115	0.182	5.26		
1301	1600	1462	0.0822	0.104	3.76		

 © Agilent Technologies, Inc. 2016

1601	1900	1756	0.0721	0.0761	3.30		
1901	2200	2062	0.0610	0.0552	2.79		
2201	2500	2361	0.0521	0.0414	2.39		
2501	3000	2755	0.0781	0.0543	3.58		
3001	4000	3495	0.102	0.0566	4.66		
4001	7000	5436	0.106	0.0392	4.87		
7001	15000	10772	0.0814	0.0154	3.73		
15001	48500	24686	0.0878	0.00745	4.02		

A2: Cat2: 4 1932 S. rigens

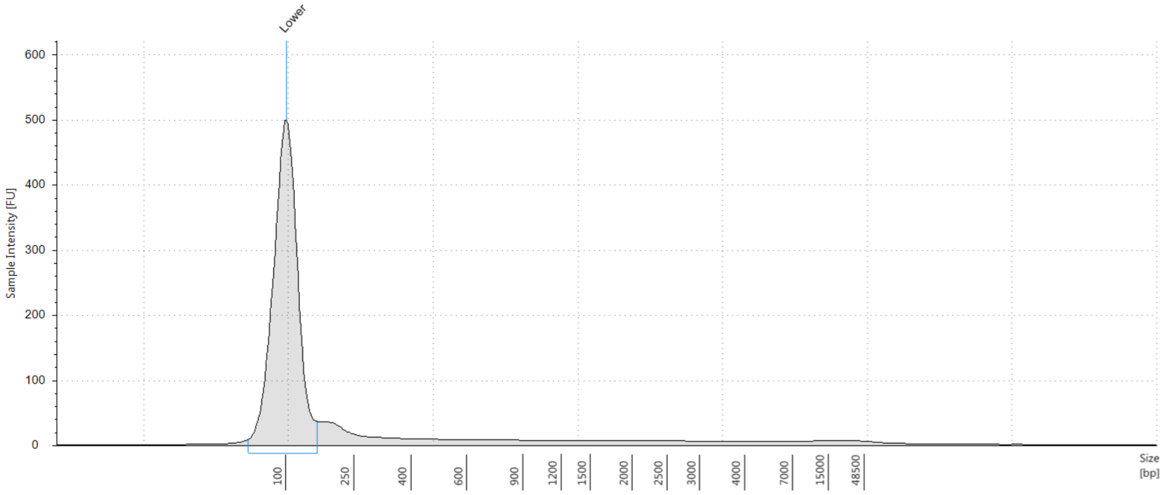

Sample Table

Well	DIN	Conc. [ng/µl]	Sample Description	Alert	Observations
A2	-	2.90	Cat2: 4	⚠	Sample concentration outside functional range for DIN

Peak Table

Size [bp]	Calibrated Conc. [ng/µl]	Assigned Conc. [ng/µl]	% Integrated Area	From [bp]	To [bp]	Peak Comment	Observations
100	8.50	8.50	-	59	152		Lower Marker
-	-	-	-	-	-		Sample Well

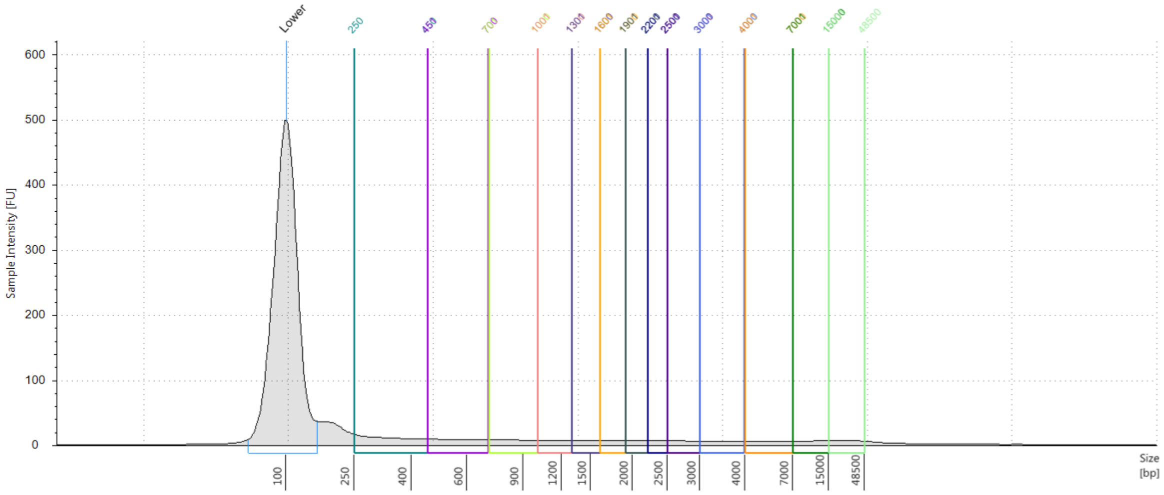

Region Table

From [bp]	To [bp]	Average Size [bp]	Conc. [ng/µl]	Region Molarity [nmol/l]	% of Total	Region Comment	Color
250	450	338	0.478	2.49	16.52		
451	700	571	0.296	0.926	10.21		
701	1000	848	0.207	0.441	7.16		
1001	1300	1157	0.142	0.221	4.89		
1301	1600	1464	0.111	0.137	3.83		

 © Agilent Technologies, Inc. 2016

1601	1900	1759	0.0935	0.0965	3.23		
1901	2200	2063	0.0861	0.0761	2.97		
2201	2500	2366	0.0711	0.0548	2.45		
2501	3000	2756	0.116	0.0773	3.99		
3001	4000	3507	0.148	0.0789	5.12		
4001	7000	5453	0.159	0.0560	5.51		
7001	15000	10968	0.123	0.0219	4.24		
15001	48500	24469	0.126	0.0103	4.35		

© Agilent Technologies, Inc. 2016

B2: Cat3: 1 2017 S. sachalinensis

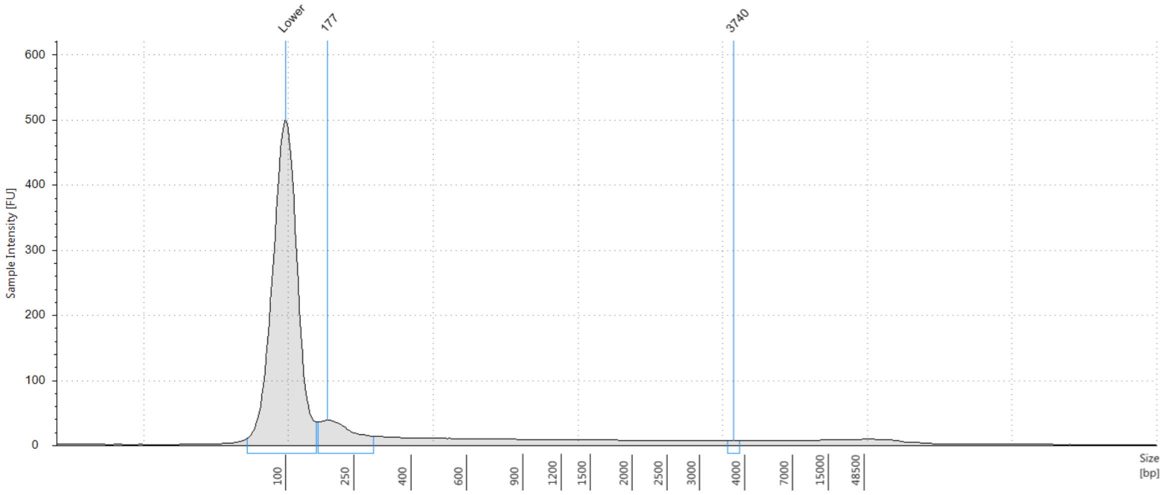

Sample Table

Well	DIN	Conc. [ng/µl]	Sample Description	Alert	Observations
B2	1.1	3.35	Cat3: 1	⚠	Sample concentration outside functional range for DIN

Peak Table

Size [bp]	Calibrated Conc. [ng/µl]	Assigned Conc. [ng/µl]	% Integrated Area	From [bp]	To [bp]	Peak Comment	Observations
100	8.50	8.50	-	59	152		Lower Marker
177	0.860	-	92.00	155	295		
3740	0.0500	-	5.35	3594	3892		
-	-	-	-	-	-		Sample Well

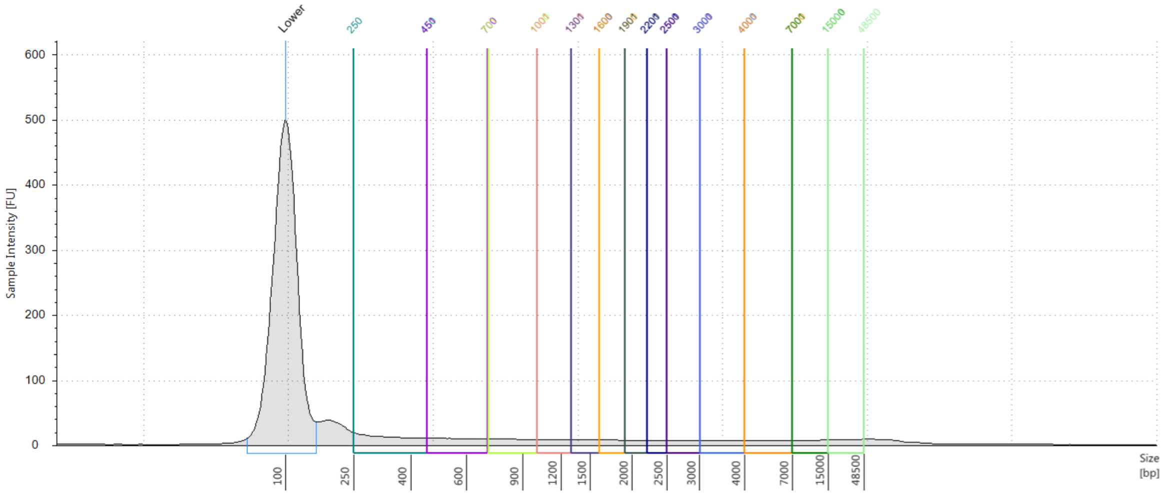

Region Table

From [bp]	To [bp]	Average Size [bp]	Conc. [ng/µl]	Region Molarity [nmol/l]	% of Total	Region Comment	Color
250	450	337	0.534	2.79	15.93		
451	700	569	0.336	1.06	10.01		
701	1000	845	0.234	0.501	6.98		
1001	1300	1150	0.159	0.250	4.73		

 © Agilent Technologies, Inc. 2016

1301	1600	1459	0.123	0.154	3.68		
1601	1900	1756	0.104	0.108	3.10		
1901	2200	2060	0.0900	0.0805	2.68		
2201	2500	2361	0.0789	0.0617	2.35		
2501	3000	2761	0.128	0.0862	3.82		
3001	4000	3507	0.165	0.0885	4.92		
4001	7000	5454	0.179	0.0630	5.33		
7001	15000	10914	0.144	0.0257	4.28		
15001	48500	25527	0.165	0.0130	4.91		

 © Agilent Technologies, Inc. 2016

C2: Cat3: 2 2019 S. acaulis

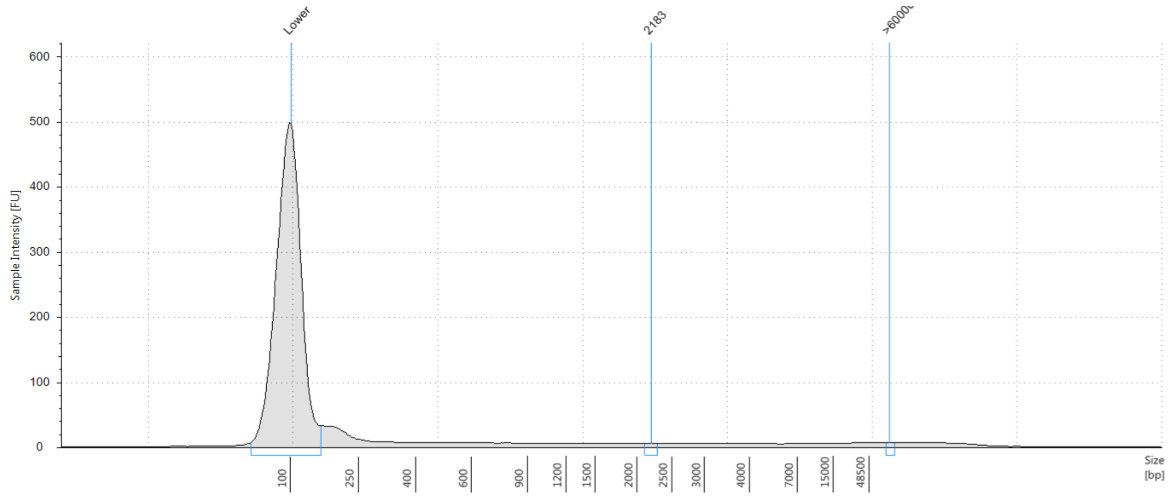

Sample Table

Well	DIN	Conc. [ng/µl]	Sample Description	Alert	Observations
C2	-	2.81	Cat3: 2	⚠	Sample concentration outside functional range for DIN

Peak Table

Size [bp]	Calibrated Conc. [ng/µl]	Assigned Conc. [ng/µl]	% Integrated Area	From [bp]	To [bp]	Peak Comment	Observations
100	8.50	8.50	-	58	150		Lower Marker
2183	0.0421	-	46.82	2096	2274		
>60000	0.0390	-	43.42	>60000	>60000		
-	-	-	-	-	-		Sample Well

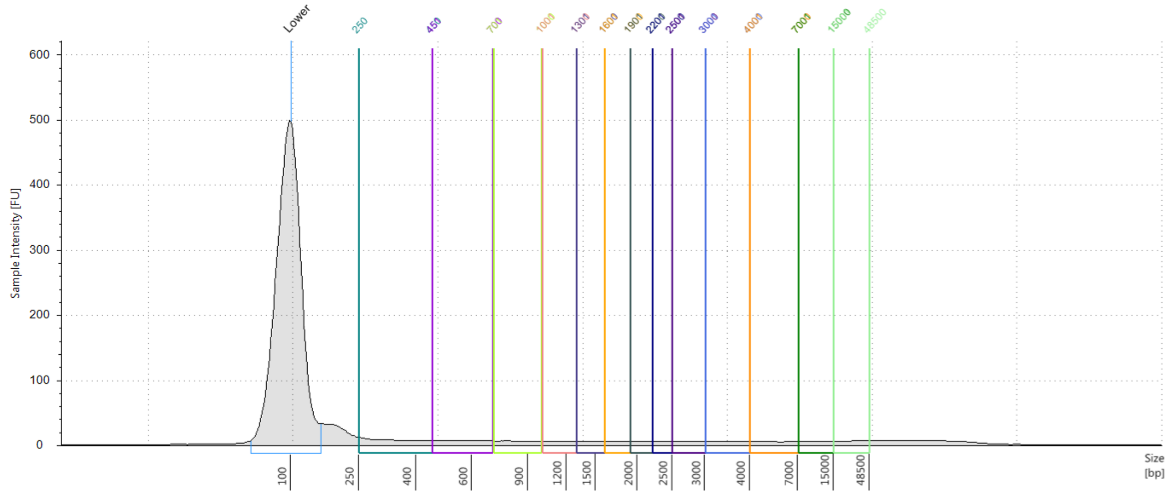

Region Table

From [bp]	To [bp]	Average Size [bp]	Conc. [ng/µl]	Region Molarity [nmol/l]	% of Total	Region Comment	Color
250	450	340	0.347	1.76	12.34		
451	700	569	0.241	0.729	8.56		
701	1000	849	0.170	0.348	6.06		
1001	1300	1154	0.114	0.171	4.07		

 © Agilent Technologies, Inc. 2016

1301	1600	1461	0.0954	0.113	3.39		
1601	1900	1764	0.0811	0.0795	2.88		
1901	2200	2064	0.0678	0.0568	2.41		
2201	2500	2369	0.0598	0.0436	2.12		
2501	3000	2763	0.101	0.0636	3.59		
3001	4000	3508	0.127	0.0636	4.51		
4001	7000	5446	0.140	0.0462	4.96		
7001	15000	10871	0.110	0.0185	3.91		
15001	48500	25582	0.131	0.00980	4.67		

D2: Cat3: 3 2019 S. burchellii

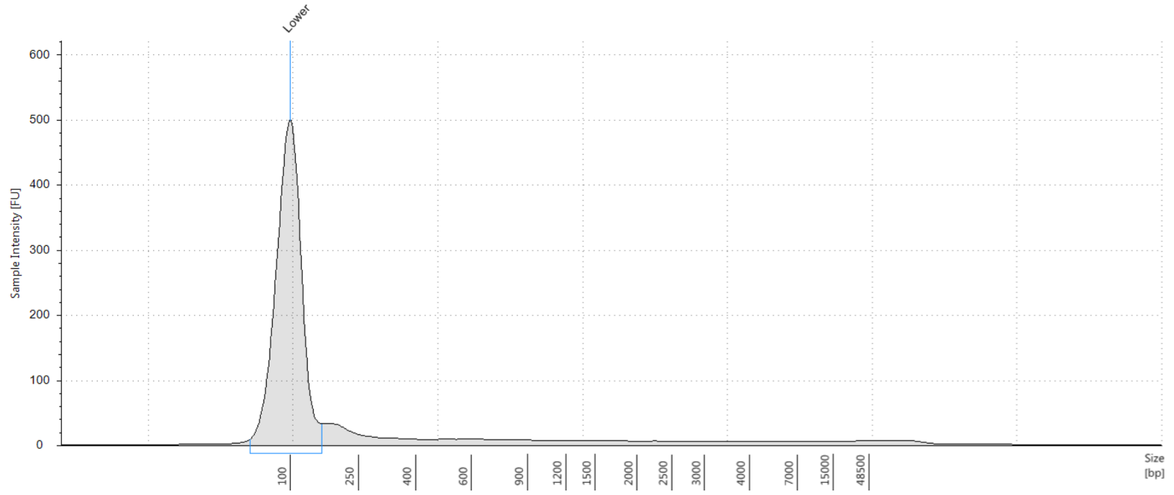

Sample Table

Well	DIN	Conc. [ng/µl]	Sample Description	Alert	Observations
D2	-	2.80	Cat3: 3	⚠	Sample concentration outside functional range for DIN

Peak Table

Size [bp]	Calibrated Conc. [ng/µl]	Assigned Conc. [ng/µl]	% Integrated Area	From [bp]	To [bp]	Peak Comment	Observations
100	8.50	8.50	-	58	153		Lower Marker
-	-	-	-	-	-		Sample Well

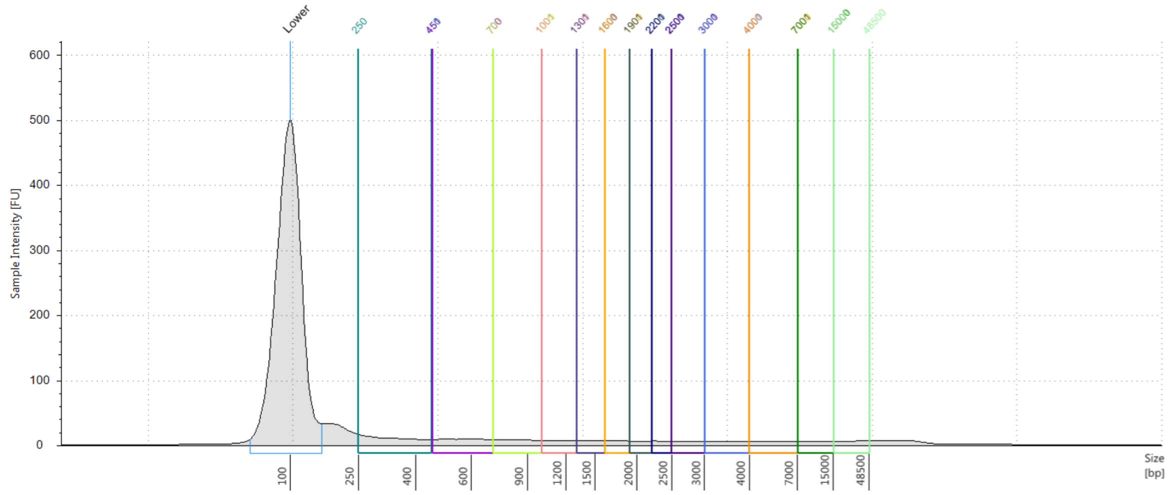

Region Table

From [bp]	To [bp]	Average Size [bp]	Conc. [ng/µl]	Region Molarity [nmol/l]	% of Total	Region Comment	Color
250	450	337	0.439	2.29	15.66		
451	700	571	0.296	0.908	10.56		
701	1000	843	0.214	0.450	7.65		
1001	1300	1150	0.132	0.204	4.70		
1301	1600	1465	0.101	0.124	3.62		

 © Agilent Technologies, Inc. 2016

1601	1900	1761	0.0893	0.0912	3.18		
1901	2200	2071	0.0747	0.0655	2.67		
2201	2500	2368	0.0635	0.0486	2.27		
2501	3000	2753	0.103	0.0686	3.68		
3001	4000	3512	0.132	0.0696	4.71		
4001	7000	5413	0.139	0.0489	4.96		
7001	15000	10793	0.106	0.0192	3.77		
15001	48500	25413	0.116	0.00919	4.14		

© Agilent Technologies, Inc. 2016

E2: Cat3: 4 2018 S. noctiflora

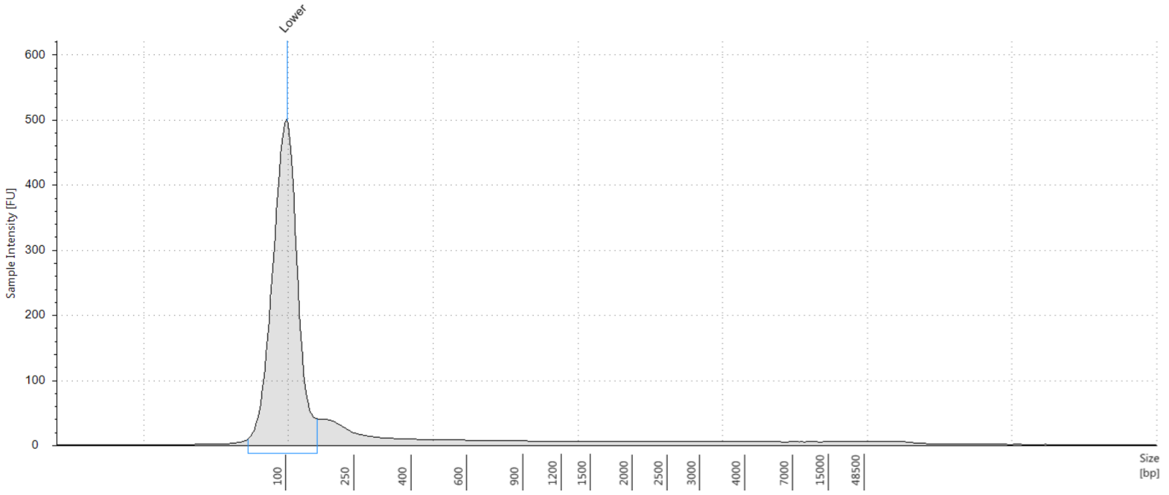

Sample Table

Well	DIN	Conc. [ng/µl]	Sample Description	Alert	Observations
E2	-	2.84	Cat3: 4	⚠	Sample concentration outside functional range for DIN

Peak Table

Size [bp]	Calibrated Conc. [ng/µl]	Assigned Conc. [ng/µl]	% Integrated Area	From [bp]	To [bp]	Peak Comment	Observations
100	8.50	8.50	-	59	151		Lower Marker
-	-	-	-	-	-		Sample Well

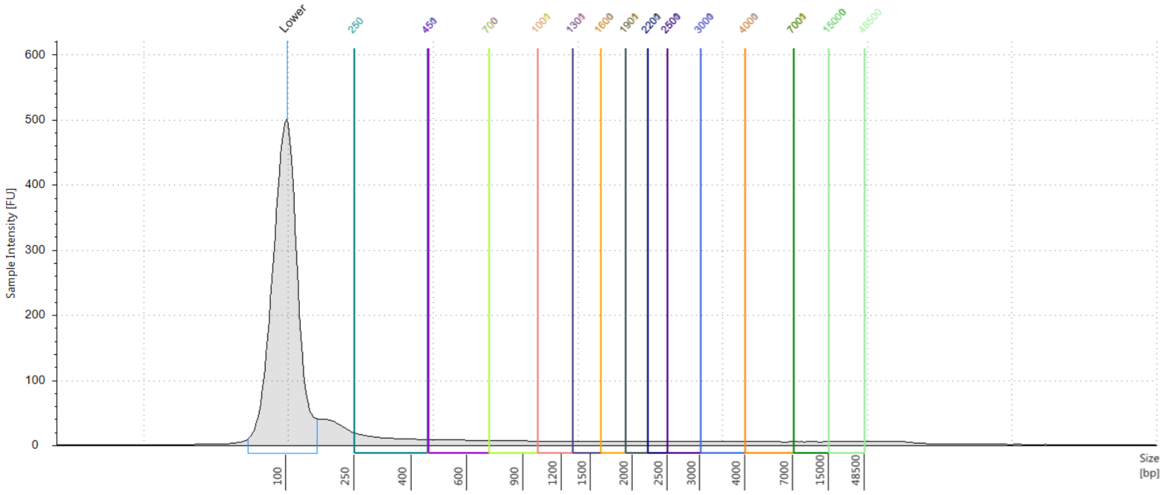

Region Table

From [bp]	To [bp]	Average Size [bp]	Conc. [ng/µl]	Region Molarity [nmol/l]	% of Total	Region Comment	Color
250	450	335	0.472	2.44	16.62		
451	700	565	0.263	0.820	9.27		
701	1000	843	0.177	0.376	6.23		
1001	1300	1154	0.112	0.175	3.94		
1301	1600	1463	0.0956	0.117	3.36		

 © Agilent Technologies, Inc. 2016

1601	1900	1761	0.0790	0.0804	2.78		
1901	2200	2063	0.0739	0.0643	2.60		
2201	2500	2372	0.0669	0.0505	2.35		
2501	3000	2768	0.104	0.0676	3.65		
3001	4000	3507	0.136	0.0707	4.80		
4001	7000	5416	0.133	0.0464	4.69		
7001	15000	10712	0.0979	0.0178	3.44		
15001	48500	25539	0.111	0.00882	3.91		

© Agilent Technologies, Inc. 2016

F2: Ladder

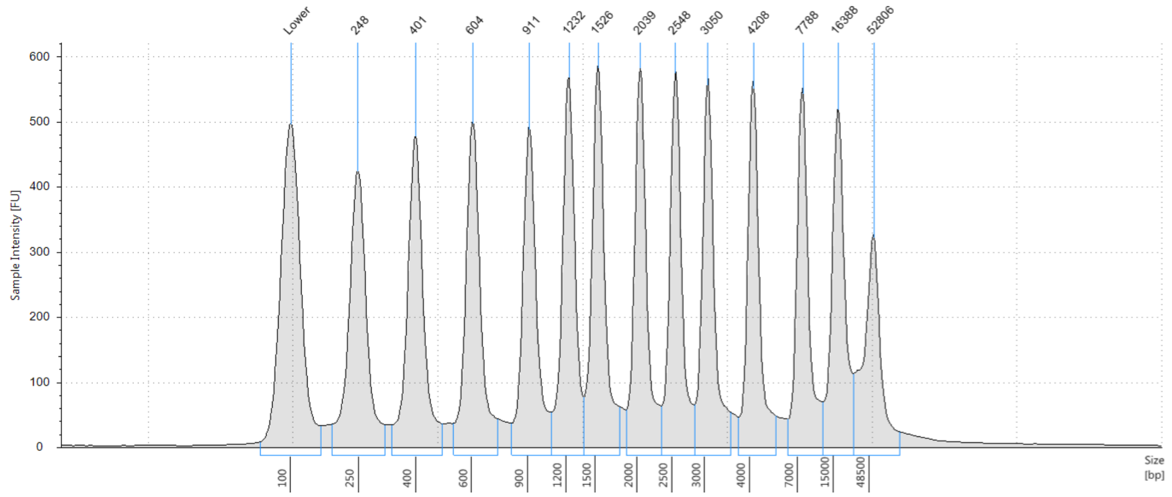

Sample Table

Well	DIN	Conc. [ng/µl]	Sample Description	Alert	Observations
F2	-	78.0	Ladder		Ladder run as sample

Peak Table

Size [bp]	Calibrated Conc. [ng/µl]	Assigned Conc. [ng/µl]	% Integrated Area	From [bp]	To [bp]	Peak Comment	Observations
100	8.50	8.50	-	66	150		Lower Marker
248	6.26	-	8.19	175	311		
401	6.36	-	8.33	328	483		
604	6.17	-	8.08	525	721		
911	5.70	-	7.47	799	1075		
1232	6.12	-	8.02	1075	1372		
1526	6.46	-	8.46	1372	1773		
2039	6.00	-	7.85	1853	2331		
2548	5.92	-	7.76	2331	2834		
3050	5.81	-	7.60	2834	3543		
4208	5.63	-	7.37	3724	5422		
7788	5.56	-	7.28	6201	11774		
16388	5.77	-	7.55	11774	22819		
52806	4.59	-	6.02	22819	>60000		
-	-	-	-	-	-		Sample Well

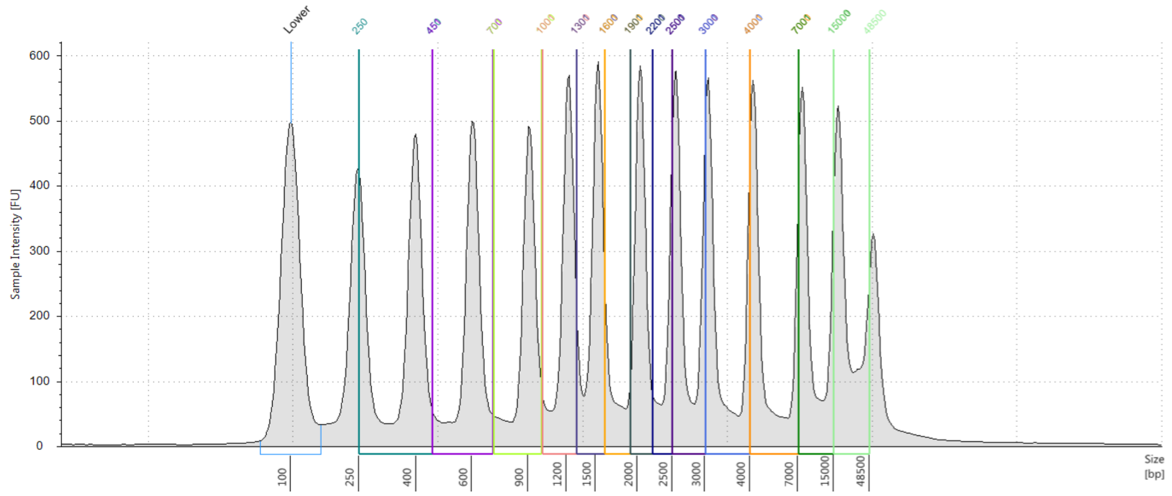

Region Table

From [bp]	To [bp]	Average Size [bp]	Conc. [ng/µl]	Region Molarity [nmol/l]	% of Total	Region Comment	Color
250	450	358	9.22	42.8	11.81		
451	700	598	6.59	17.7	8.45		
701	1000	897	5.84	10.4	7.48		
1001	1300	1207	6.03	7.89	7.73		
1301	1600	1505	6.17	6.46	7.91		
1601	1900	1739	1.56	1.48	2.00		
1901	2200	2049	5.48	4.18	7.02		
2201	2500	2433	2.16	1.41	2.76		
2501	3000	2732	6.68	3.87	8.57		
3001	4000	3409	5.92	2.80	7.58		
4001	7000	5102	6.31	2.07	8.08		
7001	15000	9887	5.86	1.01	7.50		
15001	48500	22762	6.53	0.512	8.37		

 © Agilent Technologies, Inc. 2016

Calibration

Molecular Weight Settings

Fitting type: Genomic DNA Sizing
Alignment type: From lower marker

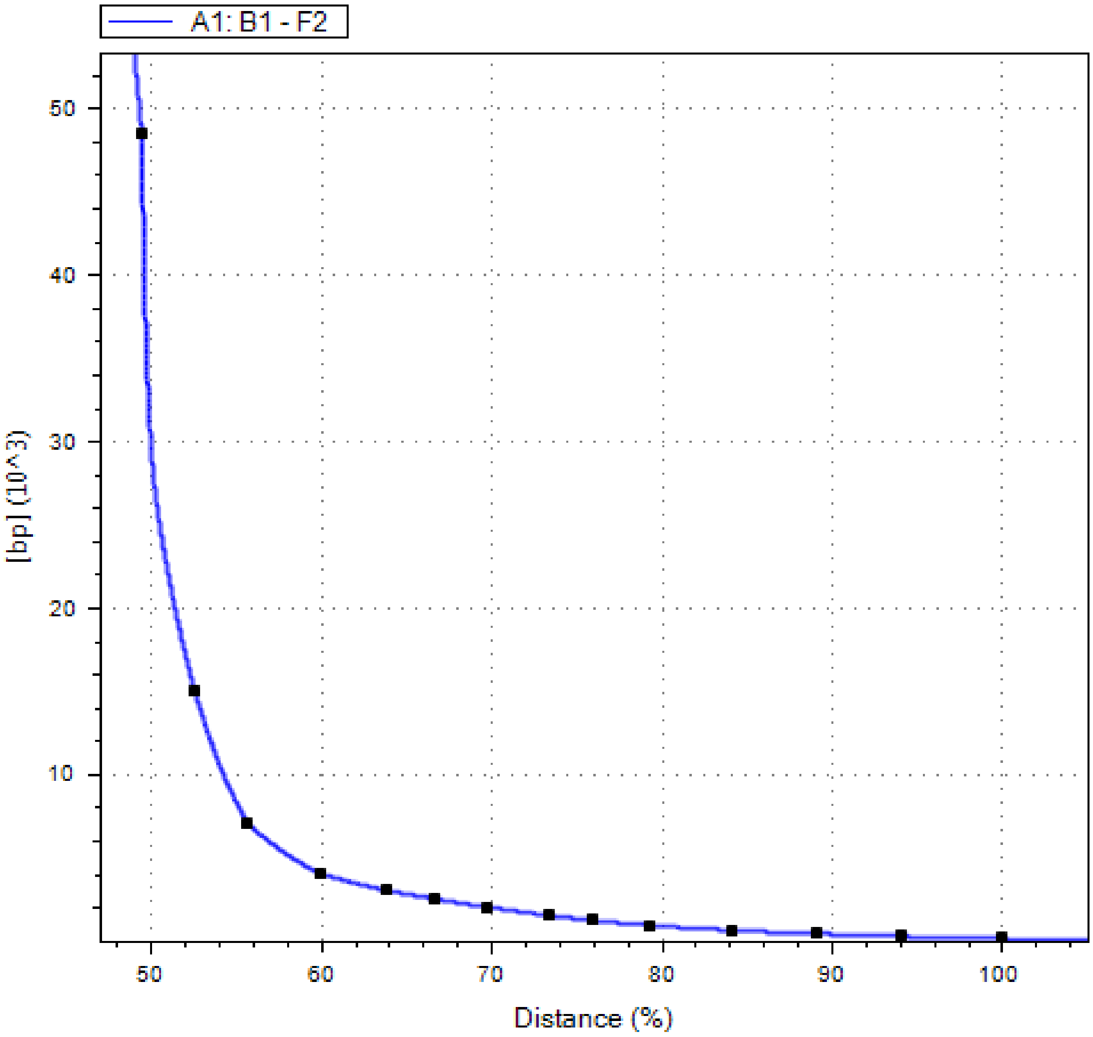

Concentration Settings

Calibration mode: Lower Marker
Normalise peaks from: Lower Marker
Fitting type: Linear Regression

 © Agilent Technologies, Inc. 2016

Experiment Properties

Run Properties

**Analysis Software Version**	2.1.38.8716
**Filename**	C:\Users\admin\Desktop\AnneSophie\2019-september-october-herbarium\library_prep\2019-10-07-01_after_0.4_size_selection.gDNA
**Assay**	Genomic DNA
**Run End Date**	07-Oct-2019 7:22 PM
**Last Saved Under Version**	2.1.38.8716
**DIN Version**	2.1.38.8716
**Study**	
**Comments**	

ScreenTape Device 1

**Username**	admin
**ScreenTape Device ID**	01-S025-190812-01-000053
**Expiry Date**	21-Oct-2019
**ScreenTape Device History**	First run 07-Oct-2019, 1 run performed
**Temperature [°C]**	23.5
**Electrophoresis Time [s]**	225
**Instrument Type**	6655
**Instrument Serial Number**	03-PM405
**Notes**	
**ScreenTape Device Run Date**	07-Oct-2019 7:00 PM

Controller Environment

**Computer**	LAB3210150
**Instrument Controller Software Version**	A.02.01 SR1
**First Run Analysis Version**	2.1.38.8716
**Operating System**	Microsoft Windows 7 Enterprise

 © Agilent Technologies, Inc. 2016
