# Supplementary material for: Retrieval of long DNA reads from herbarium specimens
Source: AoB Plants. 2023 Nov 8;15(6):plad074. doi: 10.1093/aobpla/plad074 (PMC10735254; doi:10.1093/aobpla/plad074)
Supplement: plad074_suppl_Supplementary_Appendix_S1_5 [file plad074_suppl_supplementary_appendix_s1_5.pdf]

Filename: 2019-10-27-01 after 25 cycles PCR and size selection.gDNA

Gel Image

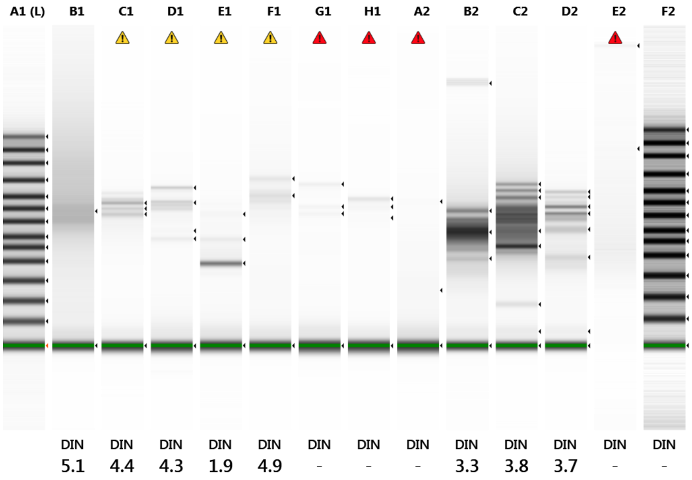

Default image (Contrast 100%)

Sample Info

Well	DIN	Conc. [ng/µl]	Sample Description	Alert	Observations
A1	-	79.7	Ladder		Ladder
B1	5.1	29.8	Cat1: 1		
C1	4.4	5.96	Cat1: 2		Sample concentration outside recommended range
D1	4.3	4.08	Cat1: 3		Sample concentration outside functional range for DIN
E1	1.9	5.77	Cat1: 4		Sample concentration outside recommended range
F1	4.9	5.34	Cat2: 5		Sample concentration outside recommended range
G1	-	1.96	Cat2: 6		Sample concentration outside functional range for DIN
H1	-	1.95	Cat2: 7		Sample concentration outside functional range for DIN
A2	-	2.49	Cat2: 8		Sample concentration outside functional range for DIN
B2	3.3	37.8	Cat3: 9		
C2	3.8	50.0	Cat3: 10		
D2	3.7	14.6	Cat3: 11		
E2	-		Cat3: 12		Marker(s) not detected
F2	-	198	Ladder		Ladder run as sample

 © Agilent Technologies, Inc. 2016

A1: Ladder

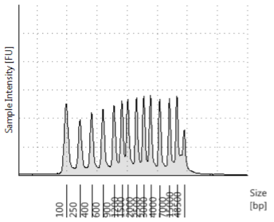

B1: Cat1: 1

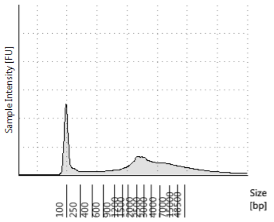

C1: Cat1: 2

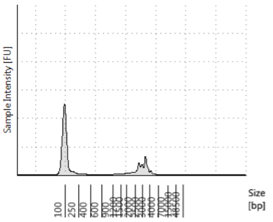

D1: Cat1: 3

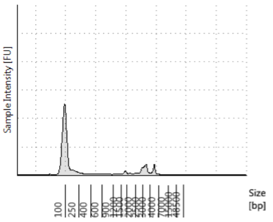

E1: Cat1: 4

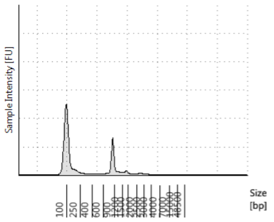

F1: Cat2: 5

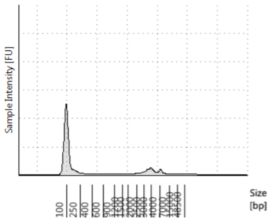

G1: Cat2: 6

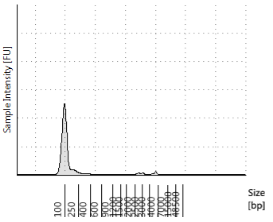

H1: Cat2: 7

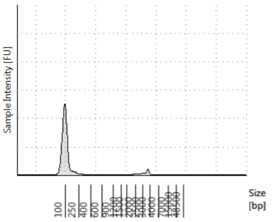

A2: Cat2: 8

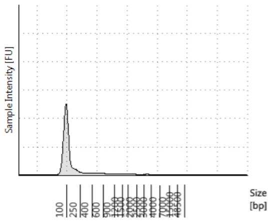

B2: Cat3: 9

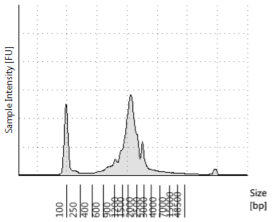

C2: Cat3: 10

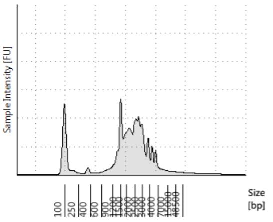

D2: Cat3: 11

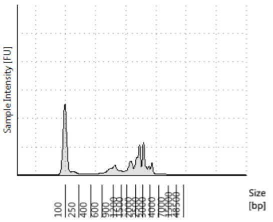

E2: Cat3: 12

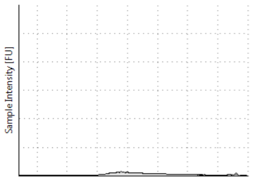

F2: Ladder

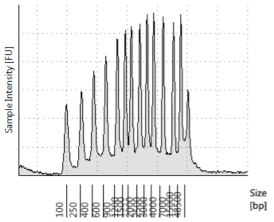

 © Agilent Technologies, Inc. 2016

A1: Ladder

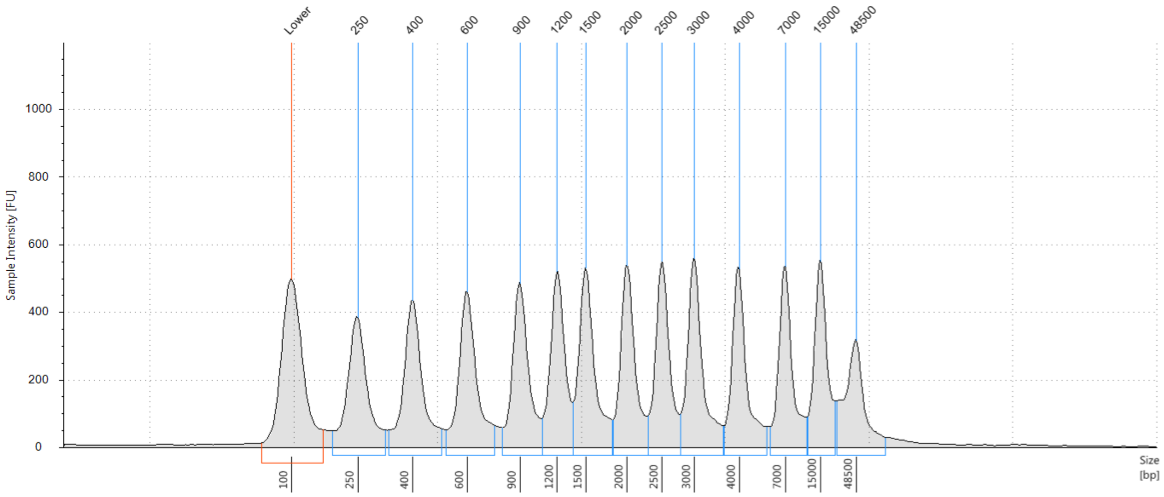

Sample Table

Well	DIN	Conc. [ng/µl]	Sample Description	Alert	Observations
A1	-	79.7	Ladder		Ladder

Peak Table

Size [bp]	Calibrated Conc. [ng/µl]	Assigned Conc. [ng/µl]	% Integrated Area	From [bp]	To [bp]	Peak Comment	Observations
100	8.50	8.50	-	66	154		Lower Marker
250	5.90	-	7.52	177	316		
400	6.27	-	8.00	327	494		
600	6.37	-	8.12	513	742		
900	6.14	-	7.83	786	1070		
1200	6.12	-	7.80	1070	1361		
1500	6.63	-	8.46	1361	1801		
2000	6.18	-	7.88	1817	2290		
2500	6.18	-	7.88	2290	2777		
3000	6.62	-	8.44	2777	3605		
4000	6.01	-	7.67	3634	5579		
7000	5.72	-	7.30	5838	11119		
15000	5.64	-	7.20	11426	20795		
48500	4.64	-	5.91	21369	>60000		

1994 acaulis

B1: Cat1: 1

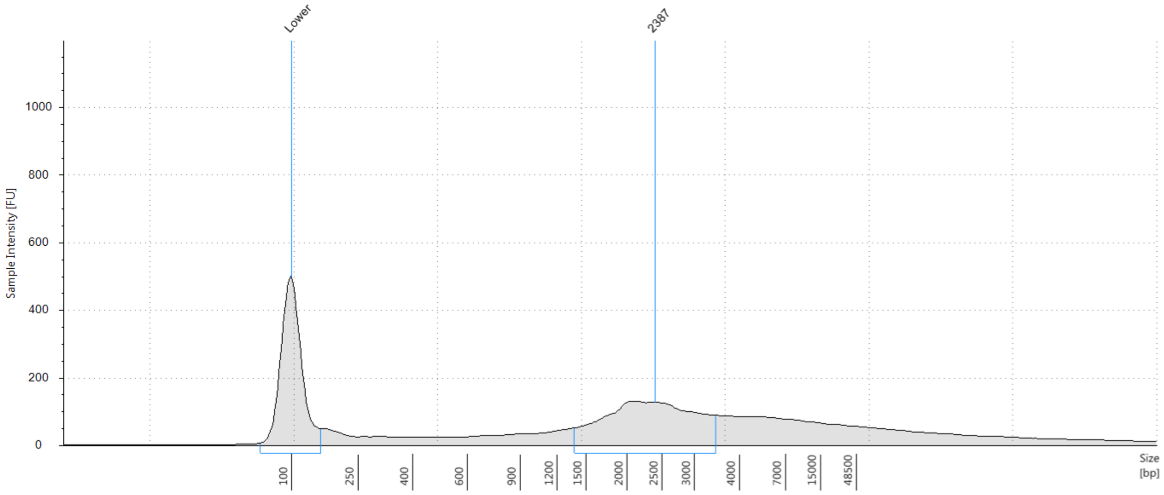

Sample Table

Well	DIN	Conc. [ng/µl]	Sample Description	Alert	Observations
B1	5.1	29.8	Cat1: 1		

Peak Table

Size [bp]	Calibrated Conc. [ng/µl]	Assigned Conc. [ng/µl]	% Integrated Area	From [bp]	To [bp]	Peak Comment	Observations
100	8.50	8.50	-	65	149		Lower Marker
2387	10.1	-	98.57	1368	3429		
-	-	-	-	-	-		Sample Well

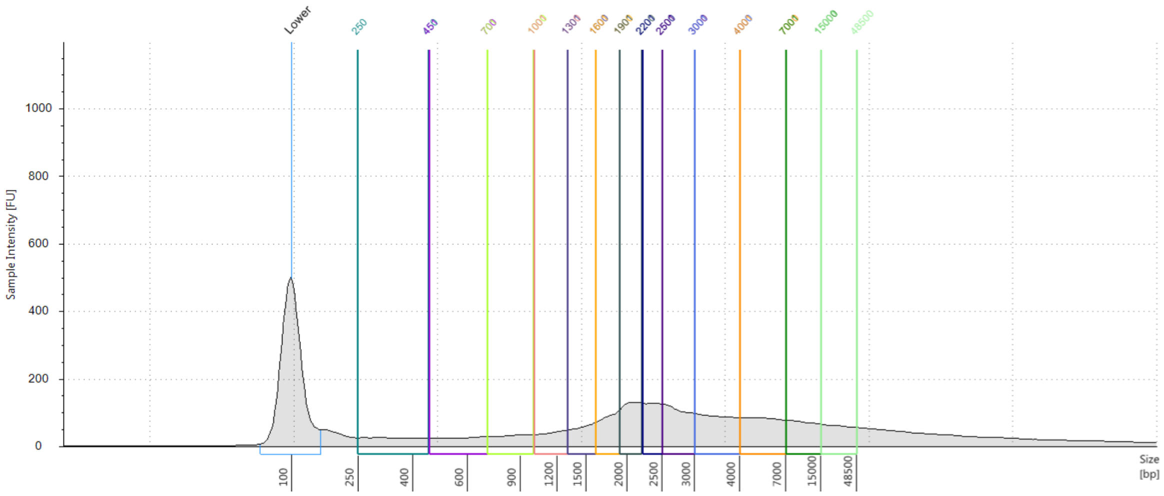

Region Table

From [bp]	To [bp]	Average Size [bp]	Conc. [ng/µl]	Region Molarity [nmol/l]	% of Total	Region Comment	Color
250	450	344	1.22	5.83	4.08		
451	700	575	1.06	3.00	3.56		
701	1000	855	1.03	1.93	3.45		
1001	1300	1160	0.999	1.37	3.35		
1301	1600	1468	1.17	1.26	3.94		

 © Agilent Technologies, Inc. 2016

1601	1900	1772	1.66	1.46	5.57		
1901	2200	2067	2.03	1.53	6.83		
2201	2500	2360	1.93	1.27	6.48		
2501	3000	2748	2.63	1.49	8.83		
3001	4000	3501	2.94	1.32	9.88		
4001	7000	5432	2.72	0.806	9.15		
7001	15000	10674	1.85	0.289	6.23		
15001	48500	25287	1.60	0.111	5.37		

 © Agilent Technologies, Inc. 2016

1987 burchellii

C1: Cat1: 2

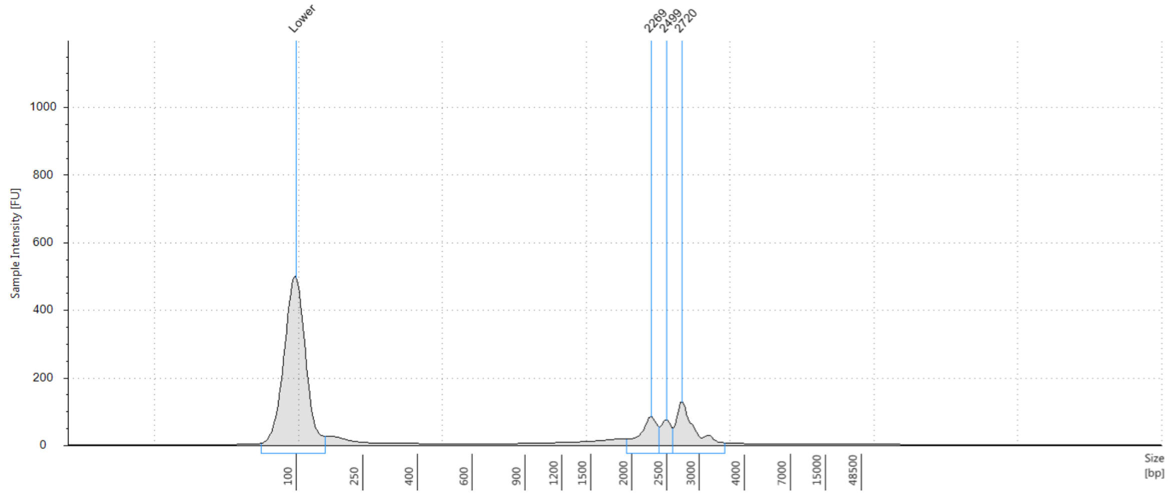

Sample Table

Well	DIN	Conc. [ng/µl]	Sample Description	Alert	Observations
C1	4.4	5.96	Cat1: 2	⚠	Sample concentration outside recommended range

Peak Table

Size [bp]	Calibrated Conc. [ng/µl]	Assigned Conc. [ng/µl]	% Integrated Area	From [bp]	To [bp]	Peak Comment	Observations
100	8.50	8.50	-	61	149		Lower Marker
2269	1.06	-	30.38	1925	2381		
2499	0.660	-	18.96	2381	2589		
2720	1.76	-	50.66	2589	3531		

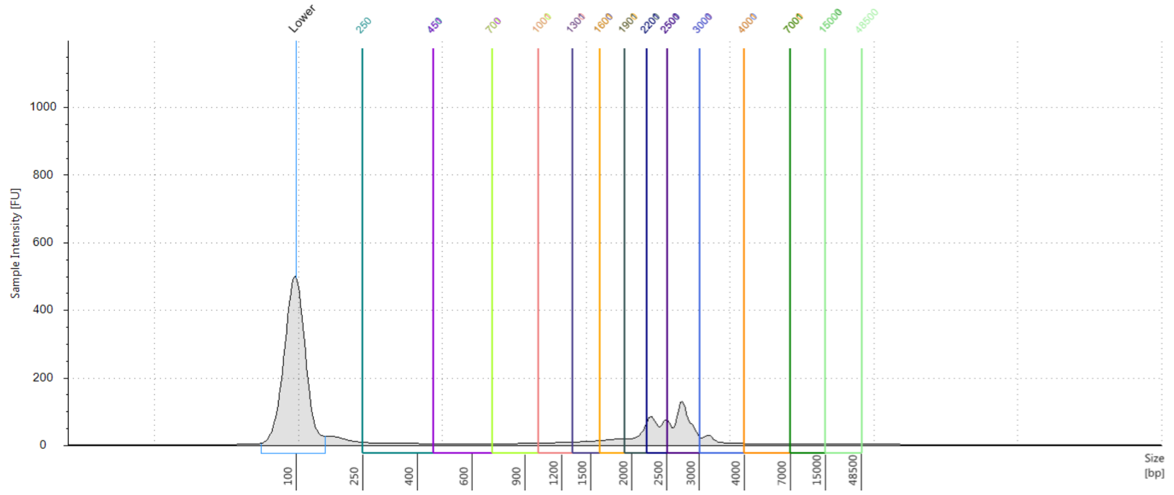

Region Table

From [bp]	To [bp]	Average Size [bp]	Conc. [ng/µl]	Region Molarity [nmol/l]	% of Total	Region Comment	Color
250	450	336	0.282	1.37	4.73		
451	700	570	0.174	0.495	2.91		
701	1000	861	0.159	0.296	2.66		
1001	1300	1168	0.176	0.239	2.96		

1301	1600	1478	0.221	0.234	3.71		
1601	1900	1773	0.289	0.254	4.85		
1901	2200	2108	0.507	0.374	8.51		
2201	2500	2368	1.03	0.676	17.33		
2501	3000	2746	1.68	0.948	28.20		
3001	4000	3328	0.407	0.192	6.83		
4001	7000	5385	0.127	0.0389	2.13		
7001	15000	10723	0.0772	0.0123	1.30		
15001	48500	24930	0.0794	0.00571	1.33		

1981 noctiflora

D1: Cat1: 3

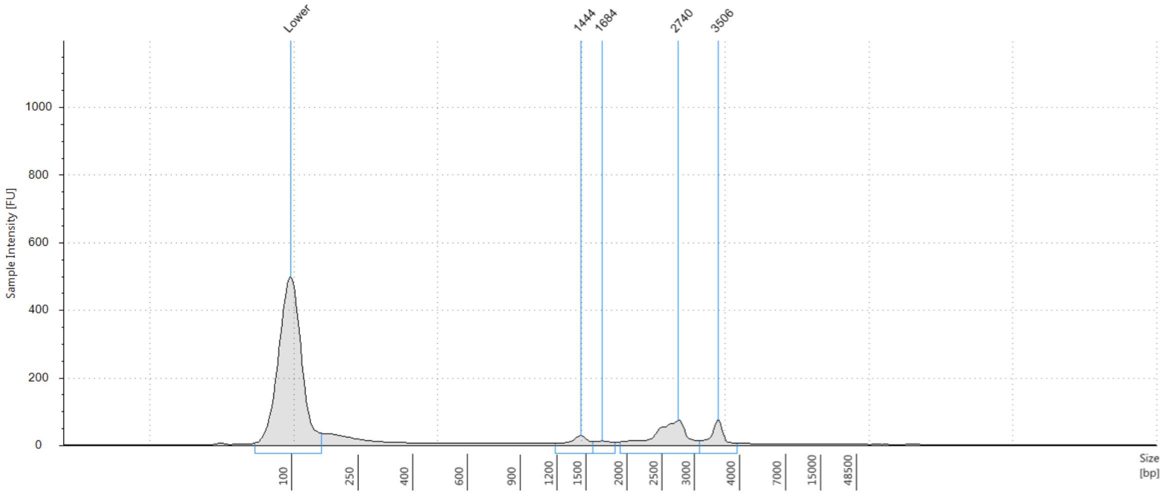

Sample Table

Well	DIN	Conc. [ng/µl]	Sample Description	Alert	Observations
D1	4.3	4.08	Cat1: 3	⚠	Sample concentration outside functional range for DIN

Peak Table

Size [bp]	Calibrated Conc. [ng/µl]	Assigned Conc. [ng/µl]	% Integrated Area	From [bp]	To [bp]	Peak Comment	Observations
100	8.50	8.50	-	60	152		Lower Marker
1444	0.317	-	12.05	1186	1583		
1684	0.134	-	5.10	1583	1840		
2740	1.55	-	58.91	1906	3105		
3506	0.630	-	23.94	3105	3959		

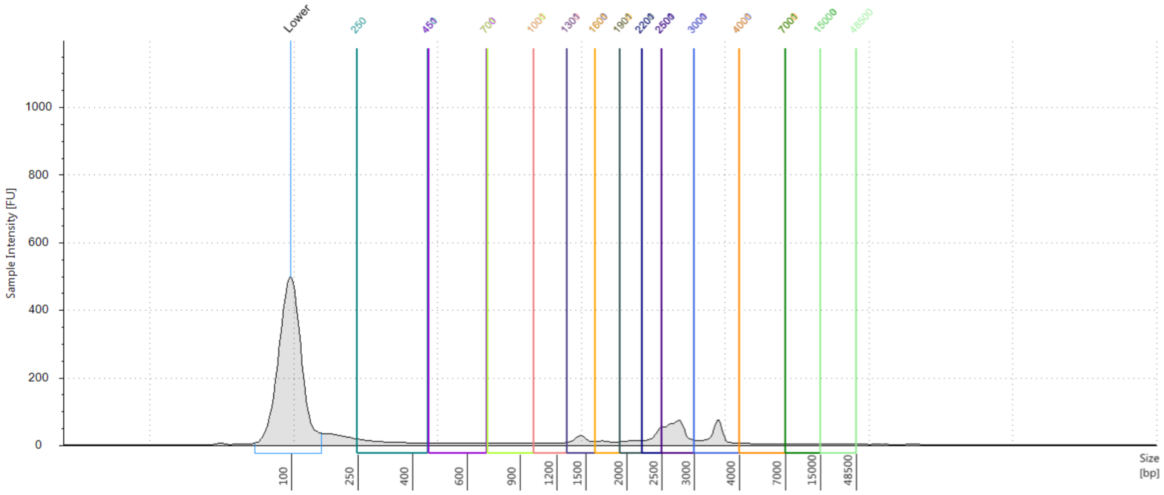

Region Table

From [bp]	To [bp]	Average Size [bp]	Conc. [ng/µl]	Region Molarity [nmol/l]	% of Total	Region Comment	Color
250	450	330	0.365	2.23	8.95		
451	700	567	0.148	0.629	3.64		
701	1000	850	0.113	0.321	2.78		

1001	1300	1166	0.0990	0.194	2.43		
1301	1600	1457	0.291	0.349	7.15		
1601	1900	1753	0.145	0.157	3.55		
1901	2200	2076	0.165	0.146	4.06		
2201	2500	2414	0.370	0.255	9.09		
2501	3000	2722	1.03	0.611	25.33		
3001	4000	3472	0.676	0.329	16.59		
4001	7000	5279	0.0674	0.0389	1.65		
7001	15000	10688	0.0291	0.0118	0.71		
15001	48500	25478	0.0210	0.00479	0.52		

1979 S. involucrata

E1: Cat1: 4

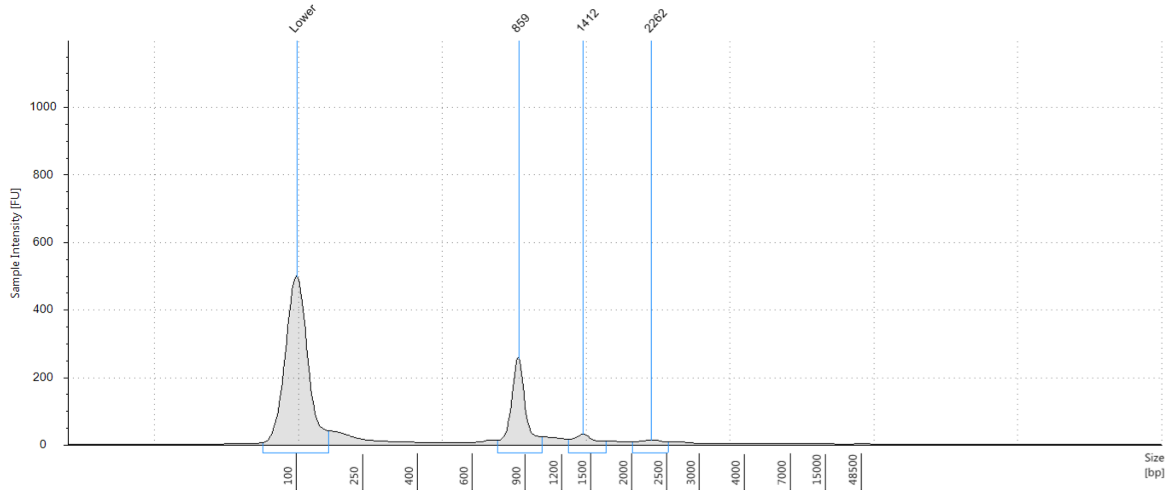

Sample Table

Well	DIN	Conc. [ng/µl]	Sample Description	Alert	Observations
E1	1.9	5.77	Cat1: 4	⚠	Sample concentration outside recommended range

Peak Table

Size [bp]	Calibrated Conc. [ng/µl]	Assigned Conc. [ng/µl]	% Integrated Area	From [bp]	To [bp]	Peak Comment	Observations
100	8.50	8.50	-	62	155		Lower Marker
859	2.68	-	79.02	727	1023		
1412	0.462	-	13.61	1255	1665		
2262	0.250	-	7.37	2004	2510		

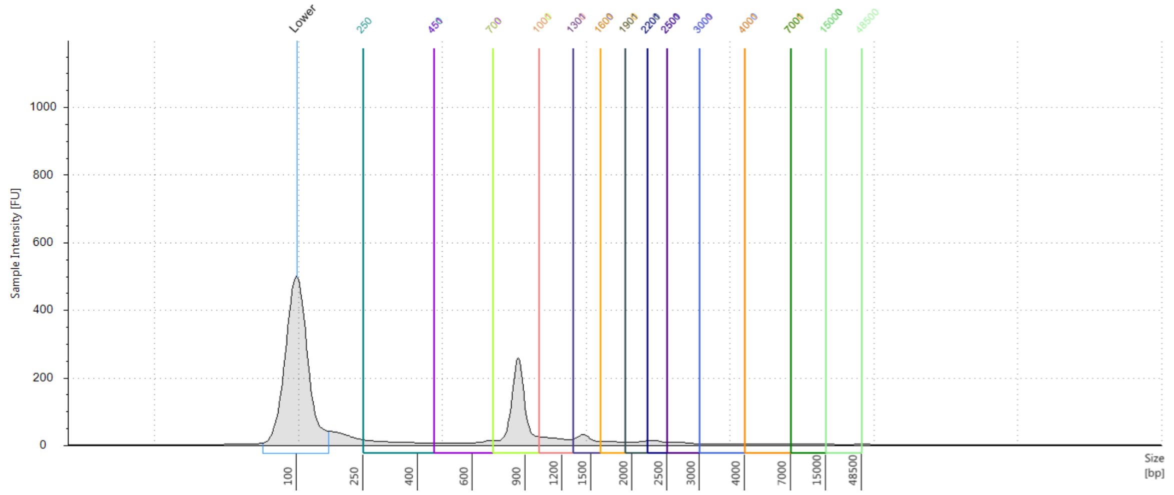

Region Table

From [bp]	To [bp]	Average Size [bp]	Conc. [ng/µl]	Region Molarity [nmol/l]	% of Total	Region Comment	Color
250	450	333	0.400	2.15	6.93		
451	700	592	0.281	0.857	4.88		
701	1000	858	2.67	4.87	46.36		
1001	1300	1142	0.450	0.646	7.81		

 © Agilent Technologies, Inc. 2016

1301	1600	1440	0.378	0.428	6.56		
1601	1900	1755	0.153	0.150	2.66		
1901	2200	2082	0.140	0.115	2.43		
2201	2500	2347	0.155	0.112	2.69		
2501	3000	2732	0.140	0.0921	2.42		
3001	4000	3483	0.0879	0.0530	1.52		
4001	7000	5407	0.0745	0.0315	1.29		
7001	15000	10688	0.0423	0.0101	0.73		
15001	48500	25172	0.0380	0.00435	0.66		

 © Agilent Technologies, Inc. 2016

1969 acaulis

F1: Cat2: 5

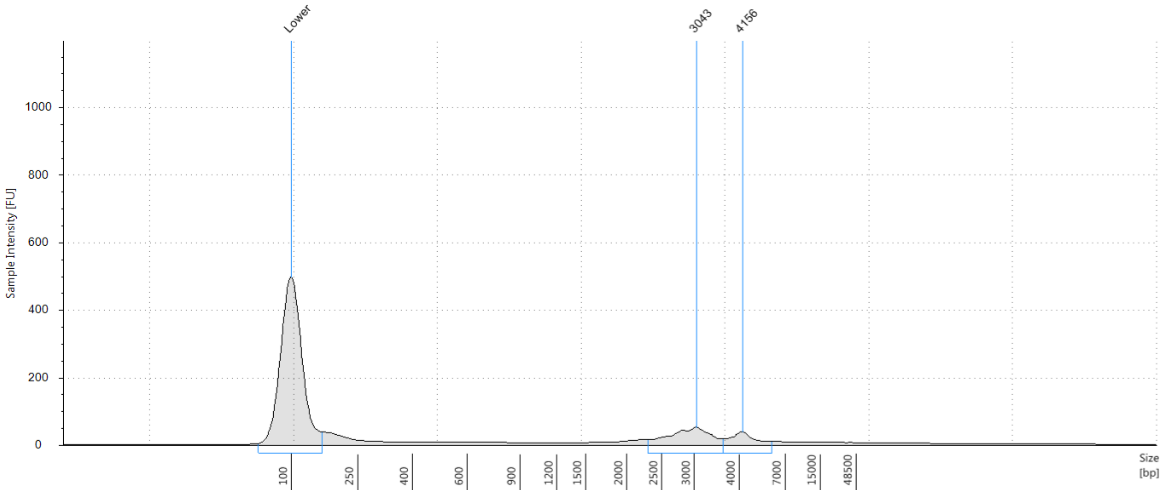

Sample Table

Well	DIN	Conc. [ng/µl]	Sample Description	Alert	Observations
F1	4.9	5.34	Cat2: 5	⚠	Sample concentration outside recommended range

Peak Table

Size [bp]	Calibrated Conc. [ng/µl]	Assigned Conc. [ng/µl]	% Integrated Area	From [bp]	To [bp]	Peak Comment	Observations
100	8.50	8.50	-	63	153		Lower Marker
3043	1.59	-	69.71	2289	3611		
4156	0.684	-	29.92	3611	5929		
-	-	-	-	-	-		Sample Well

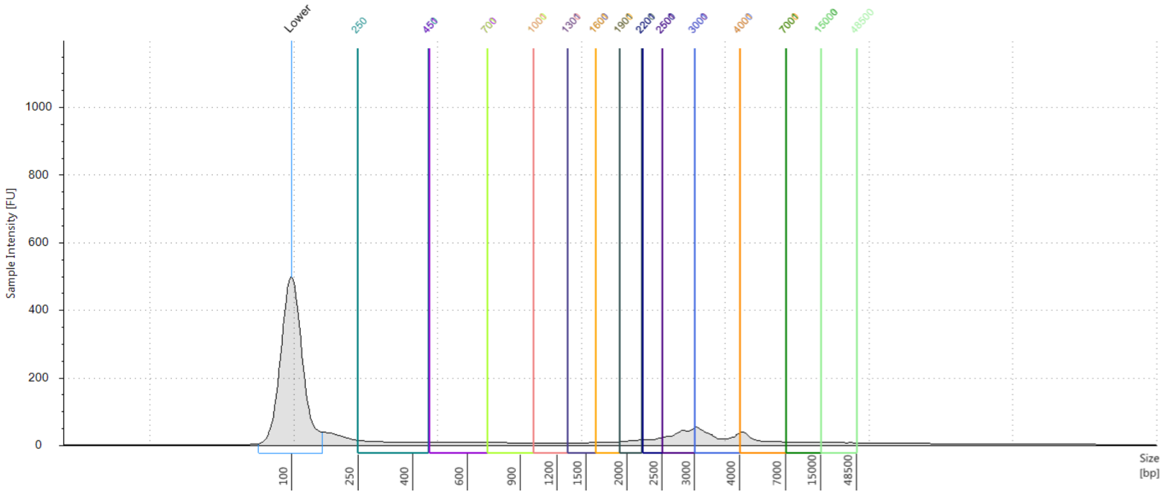

Region Table

From [bp]	To [bp]	Average Size [bp]	Conc. [ng/µl]	Region Molarity [nmol/l]	% of Total	Region Comment	Color
250	450	338	0.427	2.36	8.00		
451	700	570	0.305	1.00	5.71		
701	1000	840	0.201	0.459	3.76		
1001	1300	1157	0.130	0.219	2.44		

 © Agilent Technologies, Inc. 2016

1301	1600	1467	0.120	0.156	2.25		
1601	1900	1767	0.133	0.137	2.48		
1901	2200	2079	0.198	0.164	3.70		
2201	2500	2376	0.234	0.165	4.38		
2501	3000	2797	0.823	0.473	15.40		
3001	4000	3444	0.922	0.438	17.27		
4001	7000	5031	0.516	0.179	9.67		
7001	15000	10697	0.190	0.0347	3.56		
15001	48500	24938	0.160	0.0134	3.00		

© Agilent Technologies, Inc. 2016

1948 burchellii

G1: Cat2: 6

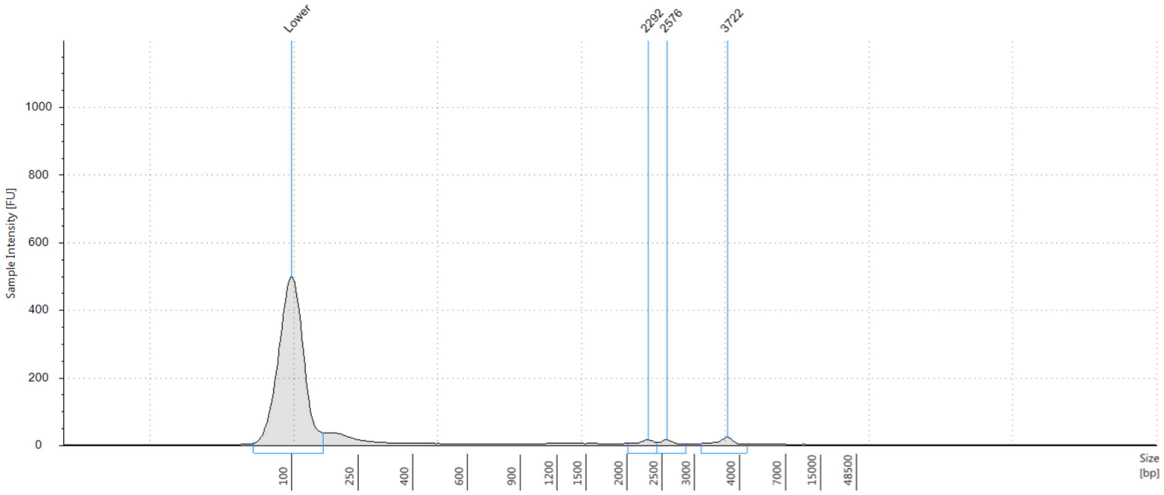

Sample Table

Well	DIN	Conc. [ng/µl]	Sample Description	Alert	Observations
G1	-	1.96	Cat2: 6	⚠	Sample concentration outside functional range for DIN

Peak Table

Size [bp]	Calibrated Conc. [ng/µl]	Assigned Conc. [ng/µl]	% Integrated Area	From [bp]	To [bp]	Peak Comment	Observations
100	8.50	8.50	-	59	155		Lower Marker
2292	0.149	-	28.49	2014	2425		
2576	0.136	-	26.08	2425	2867		
3722	0.238	-	45.43	3139	4394		

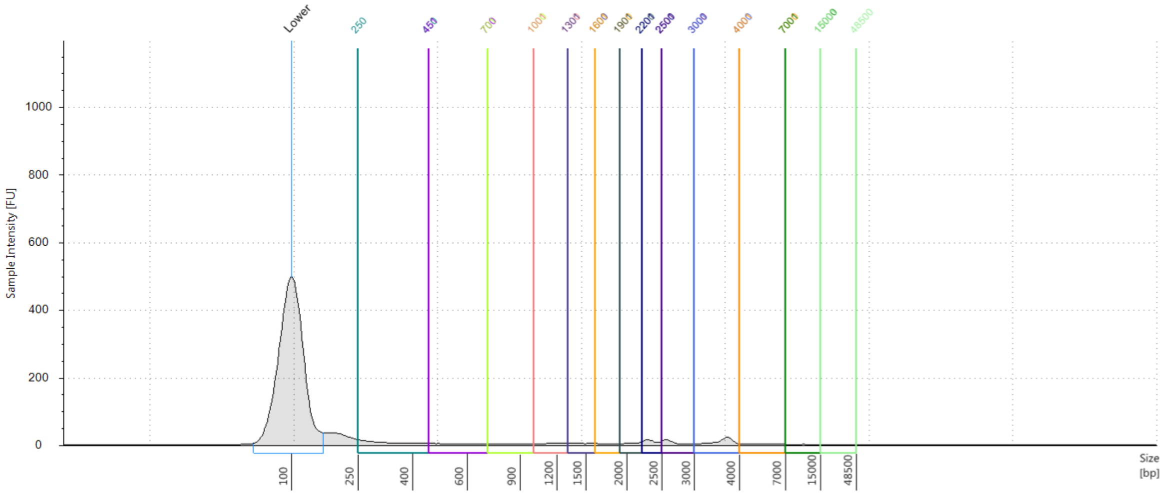

Region Table

From [bp]	To [bp]	Average Size [bp]	Conc. [ng/µl]	Region Molarity [nmol/l]	% of Total	Region Comment	Color
250	450	328	0.286	1.67	14.56		
451	700	566	0.118	0.456	5.99		
701	1000	851	0.0832	0.222	4.24		
1001	1300	1161	0.0783	0.142	3.99		

 © Agilent Technologies, Inc. 2016

1301	1600	1462	0.0649	0.0916	3.31		
1601	1900	1767	0.0508	0.0615	2.59		
1901	2200	2081	0.0633	0.0599	3.22		
2201	2500	2352	0.128	0.0940	6.51		
2501	3000	2689	0.131	0.0908	6.69		
3001	4000	3591	0.239	0.119	12.20		
4001	7000	5318	0.0540	0.0275	2.75		
7001	15000	10740	0.0235	0.00776	1.20		
15001	48500	24604	0.0190	0.00338	0.97		

© Agilent Technologies, Inc. 2016

1959 uralensis

H1: Cat2: 7

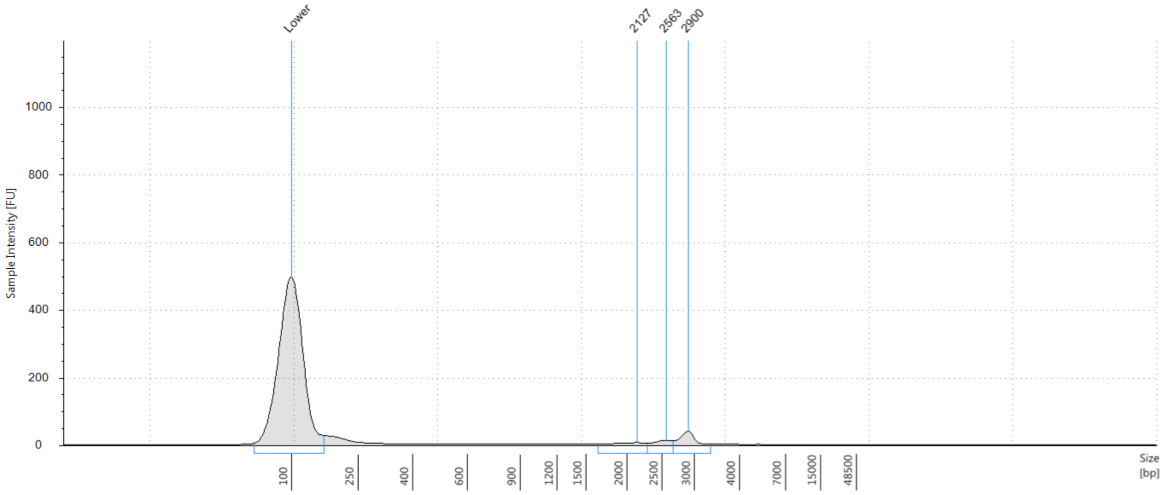

Sample Table

Well	DIN	Conc. [ng/µl]	Sample Description	Alert	Observations
H1	-	1.95	Cat2: 7	⚠	Sample concentration outside functional range for DIN

Peak Table

Size [bp]	Calibrated Conc. [ng/µl]	Assigned Conc. [ng/µl]	% Integrated Area	From [bp]	To [bp]	Peak Comment	Observations
100	8.50	8.50	-	59	157		Lower Marker
2127	0.157	-	21.12	1631	2272		
2563	0.170	-	22.81	2272	2658		
2900	0.417	-	56.07	2658	3323		

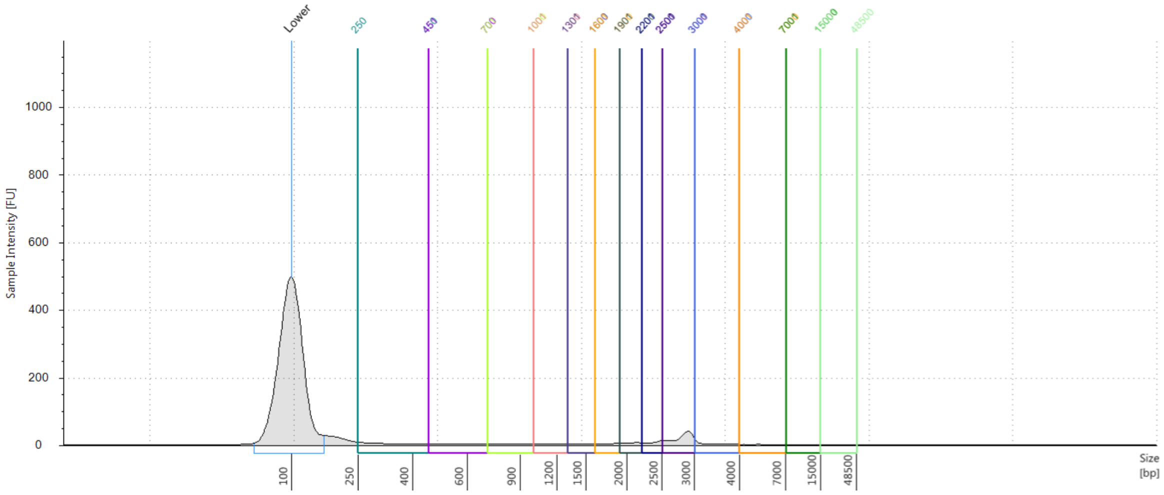

Region Table

From [bp]	To [bp]	Average Size [bp]	Conc. [ng/µl]	Region Molarity [nmol/l]	% of Total	Region Comment	Color
250	450	335	0.189	1.05	9.67		
451	700	572	0.115	0.386	5.90		
701	1000	851	0.0942	0.210	4.82		
1001	1300	1155	0.0709	0.115	3.63		

 © Agilent Technologies, Inc. 2016

1301	1600	1459	0.0573	0.0736	2.93		
1601	1900	1765	0.0626	0.0643	3.20		
1901	2200	2074	0.0911	0.0756	4.66		
2201	2500	2394	0.101	0.0708	5.18		
2501	3000	2810	0.451	0.256	23.08		
3001	4000	3389	0.103	0.0571	5.28		
4001	7000	5436	0.0489	0.0203	2.50		
7001	15000	10842	0.0291	0.00672	1.49		
15001	48500	25276	0.0263	0.00287	1.34		

© Agilent Technologies, Inc. 2016

1932 S. rigens

A2: Cat2: 8

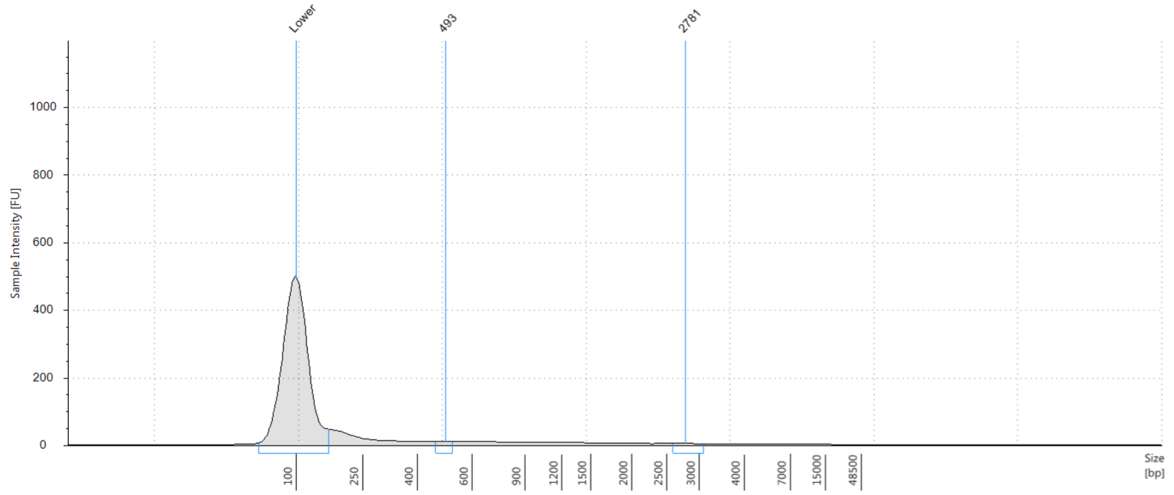

Sample Table

Well	DIN	Conc. [ng/µl]	Sample Description	Alert	Observations
A2	-	2.49	Cat2: 8	⚠	Sample concentration outside functional range for DIN

Peak Table

Size [bp]	Calibrated Conc. [ng/µl]	Assigned Conc. [ng/µl]	% Integrated Area	From [bp]	To [bp]	Peak Comment	Observations
100	8.50	8.50	-	59	156		Lower Marker
493	0.108	-	57.09	457	517		
2781	0.0811	-	42.91	2588	3086		

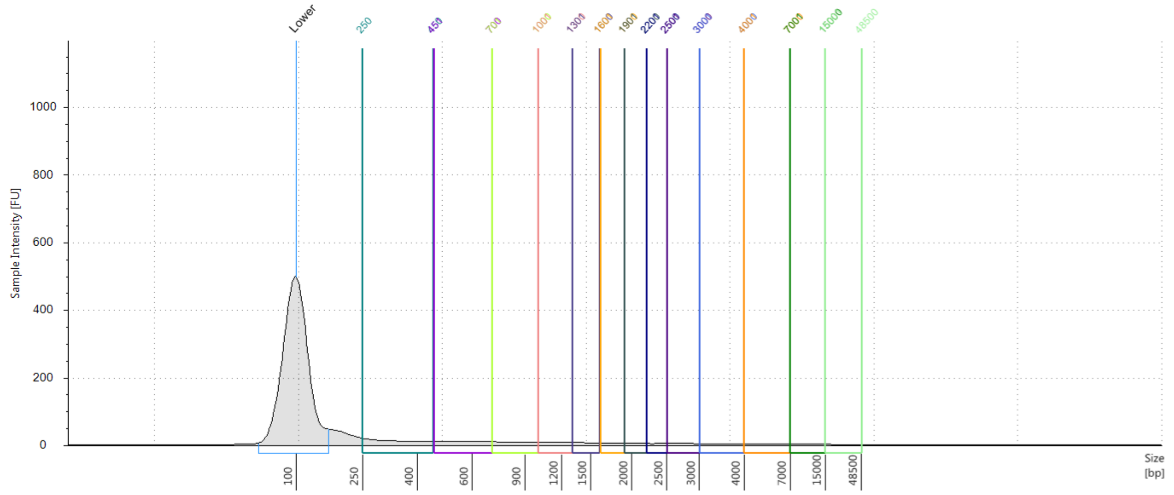

Region Table

From [bp]	To [bp]	Average Size [bp]	Conc. [ng/µl]	Region Molarity [nmol/l]	% of Total	Region Comment	Color
250	450	338	0.513	2.69	20.60		
451	700	569	0.350	1.09	14.04		
701	1000	845	0.205	0.446	8.23		
1001	1300	1156	0.144	0.230	5.79		
1301	1600	1457	0.0992	0.128	3.98		

1601	1900	1761	0.0761	0.0847	3.06		
1901	2200	2069	0.0549	0.0539	2.21		
2201	2500	2372	0.0493	0.0426	1.98		
2501	3000	2757	0.0871	0.0630	3.50		
3001	4000	3475	0.0648	0.0448	2.60		
4001	7000	5385	0.0467	0.0246	1.88		
7001	15000	10640	0.0323	0.00934	1.30		
15001	48500	24791	0.0227	0.00351	0.91		

 © Agilent Technologies, Inc. 2016

2017 S. sachalinensis

B2: Cat3: 9

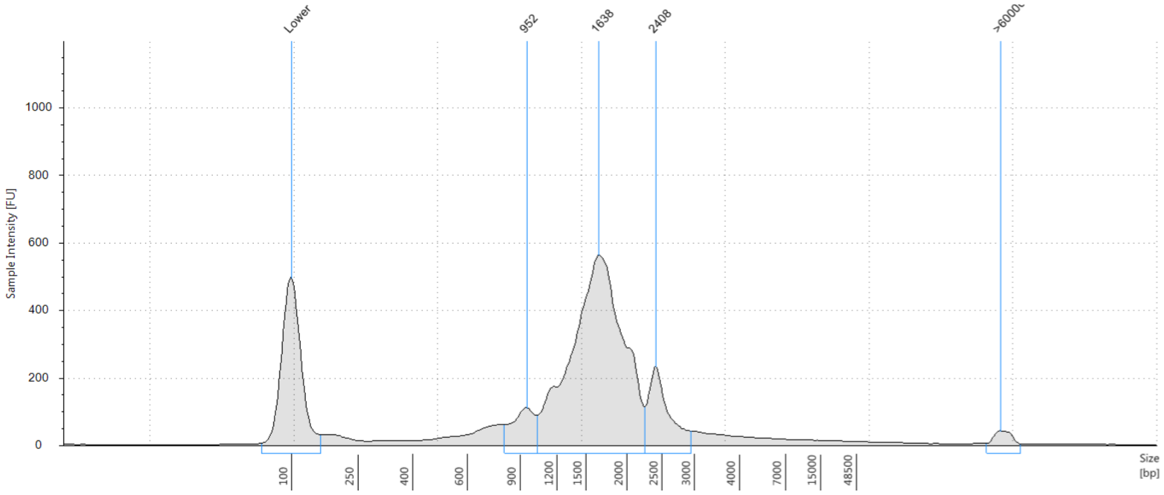

Sample Table

Well	DIN	Conc. [ng/µl]	Sample Description	Alert	Observations
B2	3.3	37.8	Cat3: 9		

Peak Table

Size [bp]	Calibrated Conc. [ng/µl]	Assigned Conc. [ng/µl]	% Integrated Area	From [bp]	To [bp]	Peak Comment	Observations
100	8.50	8.50	-	66	149		Lower Marker
952	2.14	-	6.60	800	1028		
1638	25.7	-	79.05	1028	2240		
2408	4.06	-	12.51	2240	2946		
>60000	0.599	-	1.84	>60000	>60000		

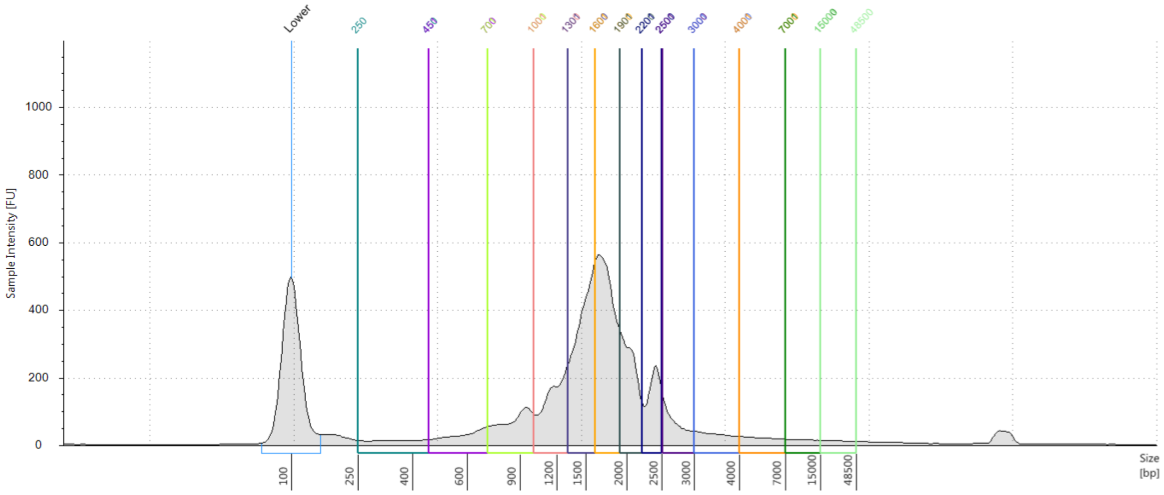

Region Table

From [bp]	To [bp]	Average Size [bp]	Conc. [ng/µl]	Region Molarity [nmol/l]	% of Total	Region Comment	Color
250	450	350	0.535	3.47	1.41		
451	700	595	1.25	3.77	3.29		
701	1000	872	2.67	5.05	7.06		
1001	1300	1181	4.06	5.47	10.73		

1301	1600	1485	8.53	8.99	22.55		
1601	1900	1746	9.36	8.37	24.73		
1901	2200	2050	4.42	3.39	11.69		
2201	2500	2377	2.79	1.86	7.38		
2501	3000	2703	1.77	1.08	4.68		
3001	4000	3466	1.02	0.524	2.71		
4001	7000	5367	0.658	0.239	1.74		
7001	15000	10775	0.342	0.0690	0.90		
15001	48500	25155	0.266	0.0261	0.70		

2019 S. acaulis

C2: Cat3: 10

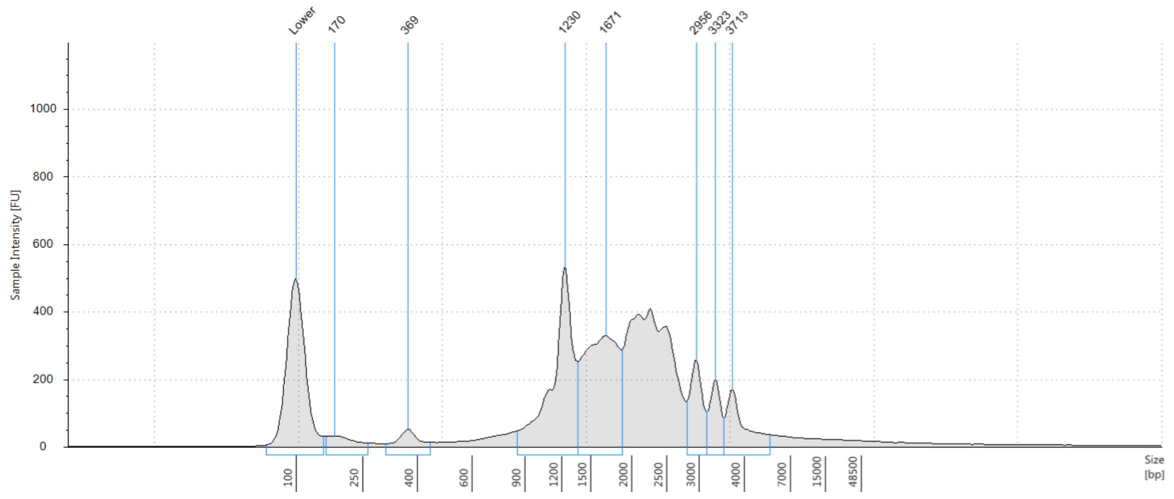

Sample Table

Well	DIN	Conc. [ng/µl]	Sample Description	Alert	Observations
C2	3.8	50.0	Cat3: 10		

Peak Table

Size [bp]	Calibrated Conc. [ng/µl]	Assigned Conc. [ng/µl]	% Integrated Area	From [bp]	To [bp]	Peak Comment	Observations
100	8.50	8.50	-	66	146		Lower Marker
170	0.682	-	2.27	151	263		
369	0.776	-	2.59	305	440		
1230	9.77	-	32.55	848	1355		
1671	11.0	-	36.52	1355	1870		
2956	3.04	-	10.11	2796	3143		
3323	2.08	-	6.93	3143	3513		
3713	2.68	-	8.92	3513	5454		
-	-	-	-	-	-		Sample Well

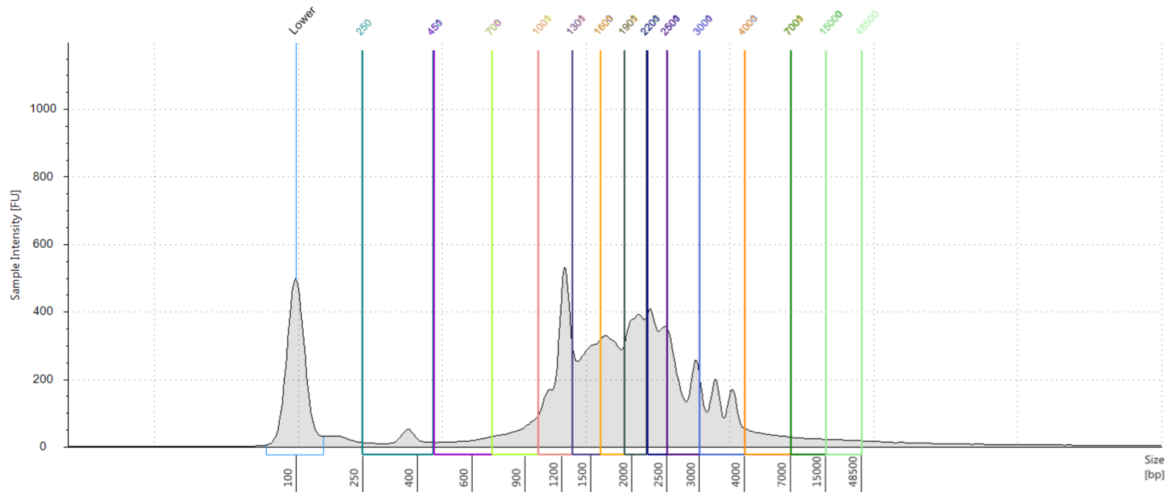

Region Table

From [bp]	To [bp]	Average Size [bp]	Conc. [ng/µl]	Region Molarity [nmol/l]	% of Total	Region Comment	Color
250	450	360	0.932	4.97	1.86		
451	700	588	0.699	2.32	1.40		

 © Agilent Technologies, Inc. 2016

701	1000	881	1.86	3.52	3.72		
1001	1300	1191	7.63	10.0	15.25		
1301	1600	1470	6.78	7.25	13.56		
1601	1900	1758	6.16	5.48	12.31		
1901	2200	2064	6.97	5.27	13.94		
2201	2500	2354	6.22	4.12	12.43		
2501	3000	2757	5.83	3.33	11.65		
3001	4000	3467	4.63	2.14	9.26		
4001	7000	5305	1.31	0.433	2.61		
7001	15000	10571	0.620	0.112	1.24		
15001	48500	24249	0.496	0.0421	0.99		

 © Agilent Technologies, Inc. 2016

2019 S. burchellii

D2: Cat3: 11

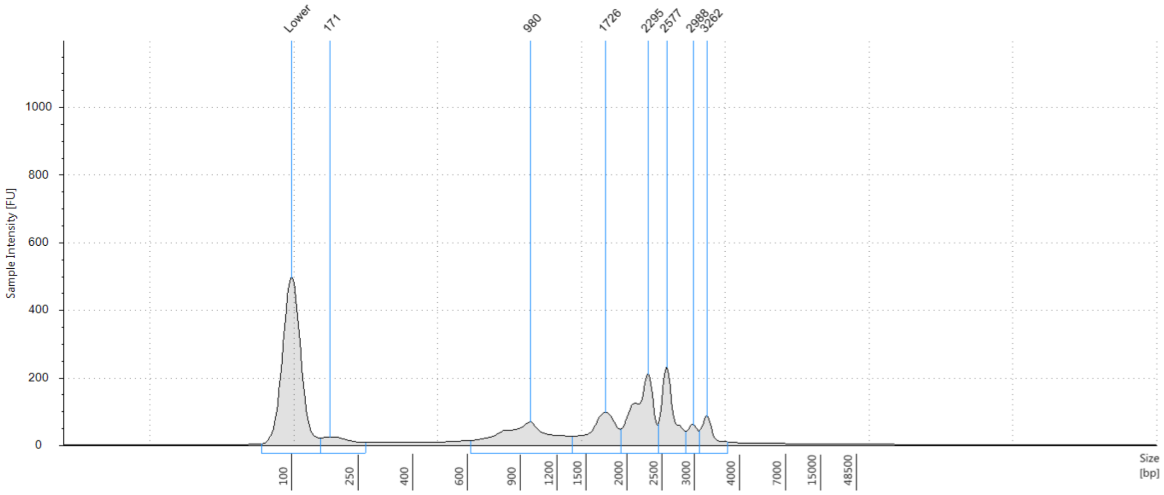

Sample Table

Well	DIN	Conc. [ng/µl]	Sample Description	Alert	Observations
D2	3.7	14.6	Cat3: 11		

Peak Table

Size [bp]	Calibrated Conc. [ng/µl]	Assigned Conc. [ng/µl]	% Integrated Area	From [bp]	To [bp]	Peak Comment	Observations
100	8.50	8.50	-	66	149		Lower Marker
171	0.628	-	4.66	149	267		
980	2.92	-	21.70	615	1348		
1726	2.21	-	16.39	1348	1917		
2295	3.67	-	27.28	1917	2447		
2577	2.55	-	18.97	2447	2864		
2988	0.601	-	4.47	2864	3108		
3262	0.879	-	6.53	3108	3709		

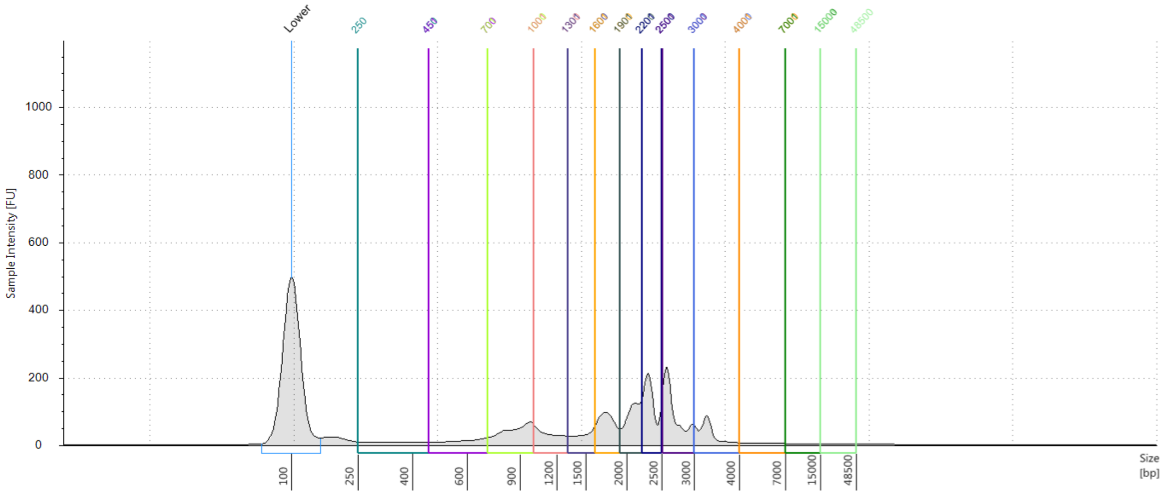

Region Table

From [bp]	To [bp]	Average Size [bp]	Conc. [ng/µl]	Region Molarity [nmol/l]	% of Total	Region Comment	Color
250	450	344	0.431	2.26	2.96		
451	700	592	0.570	1.63	3.91		
701	1000	876	1.72	3.13	11.83		

1001	1300	1140	0.957	1.34	6.57		
1301	1600	1485	0.731	0.786	5.02		
1601	1900	1749	1.56	1.40	10.73		
1901	2200	2090	1.77	1.32	12.14		
2201	2500	2336	2.33	1.55	16.02		
2501	3000	2679	2.70	1.58	18.55		
3001	4000	3307	1.16	0.559	7.93		
4001	7000	5385	0.198	0.0692	1.36		
7001	15000	10897	0.107	0.0203	0.74		
15001	48500	25261	0.0924	0.00821	0.63		

 © Agilent Technologies, Inc. 2016

2018 S. noctiflora

E2: Cat3: 12

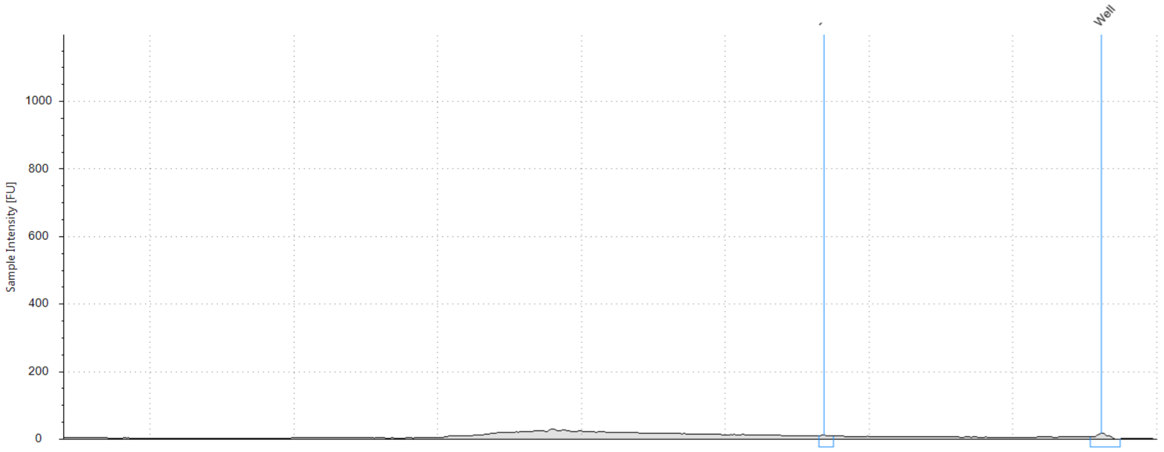

Sample Table

Well	DIN	Conc. [ng/µl]	Sample Description	Alert	Observations
E2	-		Cat3: 12	!	Marker(s) not detected

Peak Table

Size [bp]	Calibrated Conc. [ng/µl]	Assigned Conc. [ng/µl]	% Integrated Area	From [bp]	To [bp]	Peak Comment	Observations
-	-	-	39.58	-	-		
-	-	-	-	-	-		Sample Well

F2: Ladder

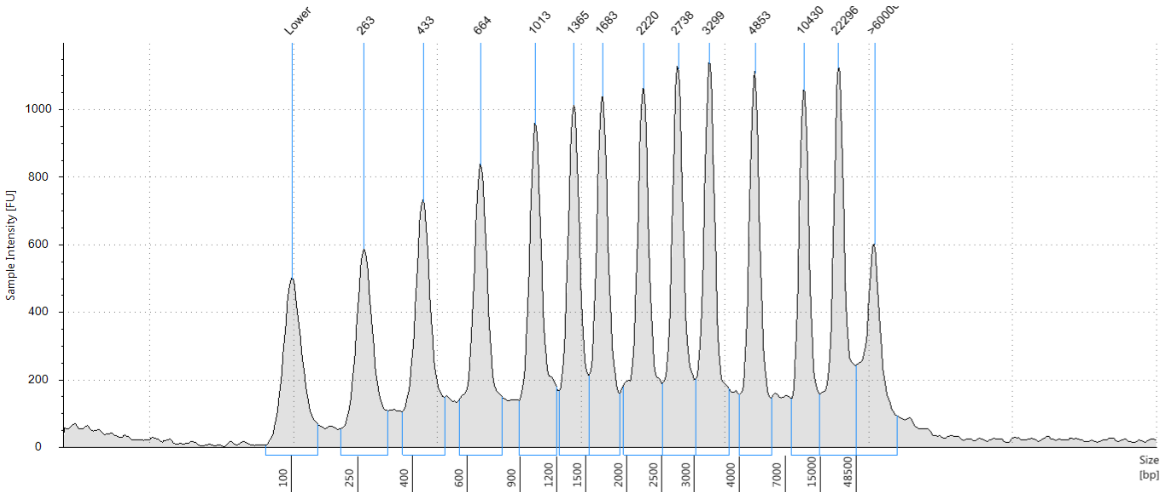

Sample Table

Well	DIN	Conc. [ng/µl]	Sample Description	Alert	Observations
F2	-	198	Ladder		Ladder run as sample

Peak Table

Size [bp]	Calibrated Conc. [ng/µl]	Assigned Conc. [ng/µl]	% Integrated Area	From [bp]	To [bp]	Peak Comment	Observations
100	8.50	8.50	-	70	144		Lower Marker
263	10.1	-	5.10	196	322		
433	13.3	-	6.71	366	506		
664	15.0	-	7.56	565	782		
1013	16.1	-	8.15	895	1192		
1365	16.2	-	8.17	1215	1529		
1683	16.6	-	8.37	1529	1900		
2220	17.7	-	8.91	1950	2500		
2738	17.7	-	8.94	2500	3022		
3299	17.6	-	8.90	3022	3748		
4853	15.9	-	8.00	3994	5903		
10430	14.4	-	7.29	7951	14840		
22296	17.3	-	8.73	14840	45106		
>60000	10.2	-	5.17	45106	>60000		

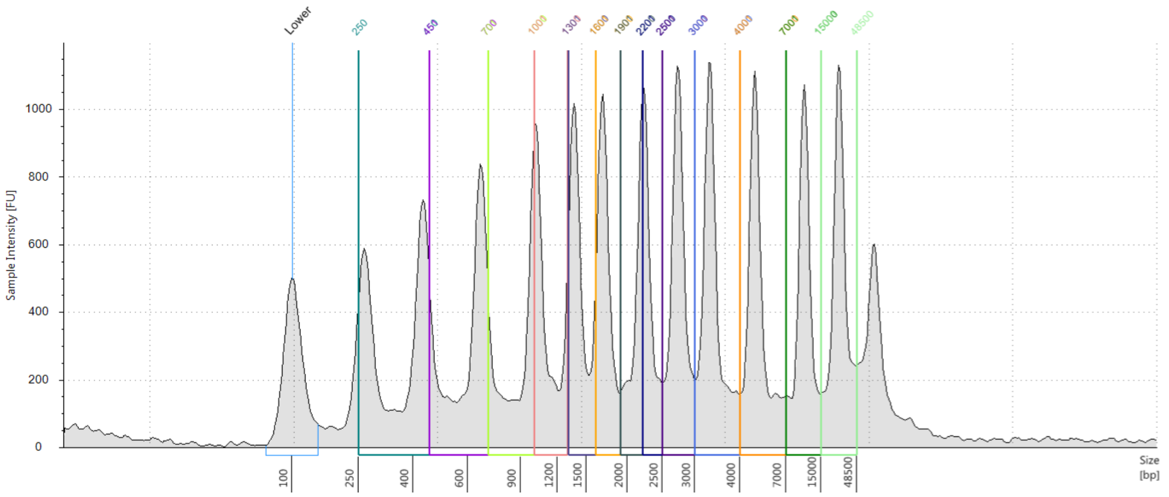

Region Table

From [bp]	To [bp]	Average Size [bp]	Conc. [ng/µl]	Region Molarity [nmol/l]	% of Total	Region Comment	Color
250	450	357	19.7	136	9.95		
451	700	609	16.4	64.5	8.27		
701	1000	889	9.54	28.8	4.81		
1001	1300	1114	13.3	25.5	6.72		
1301	1600	1429	16.0	21.7	8.10		
1601	1900	1717	15.0	16.6	7.58		
1901	2200	2119	8.17	8.18	4.13		
2201	2500	2304	11.3	9.55	5.72		
2501	3000	2752	17.6	12.5	8.88		
3001	4000	3391	18.3	11.3	9.26		
4001	7000	5086	16.5	7.14	8.35		
7001	15000	10741	14.8	2.92	7.45		
15001	48500	24213	17.4	1.52	8.80		

© Agilent Technologies, Inc. 2016

Calibration

Molecular Weight Settings

Fitting type: Genomic DNA Sizing
Alignment type: From lower marker

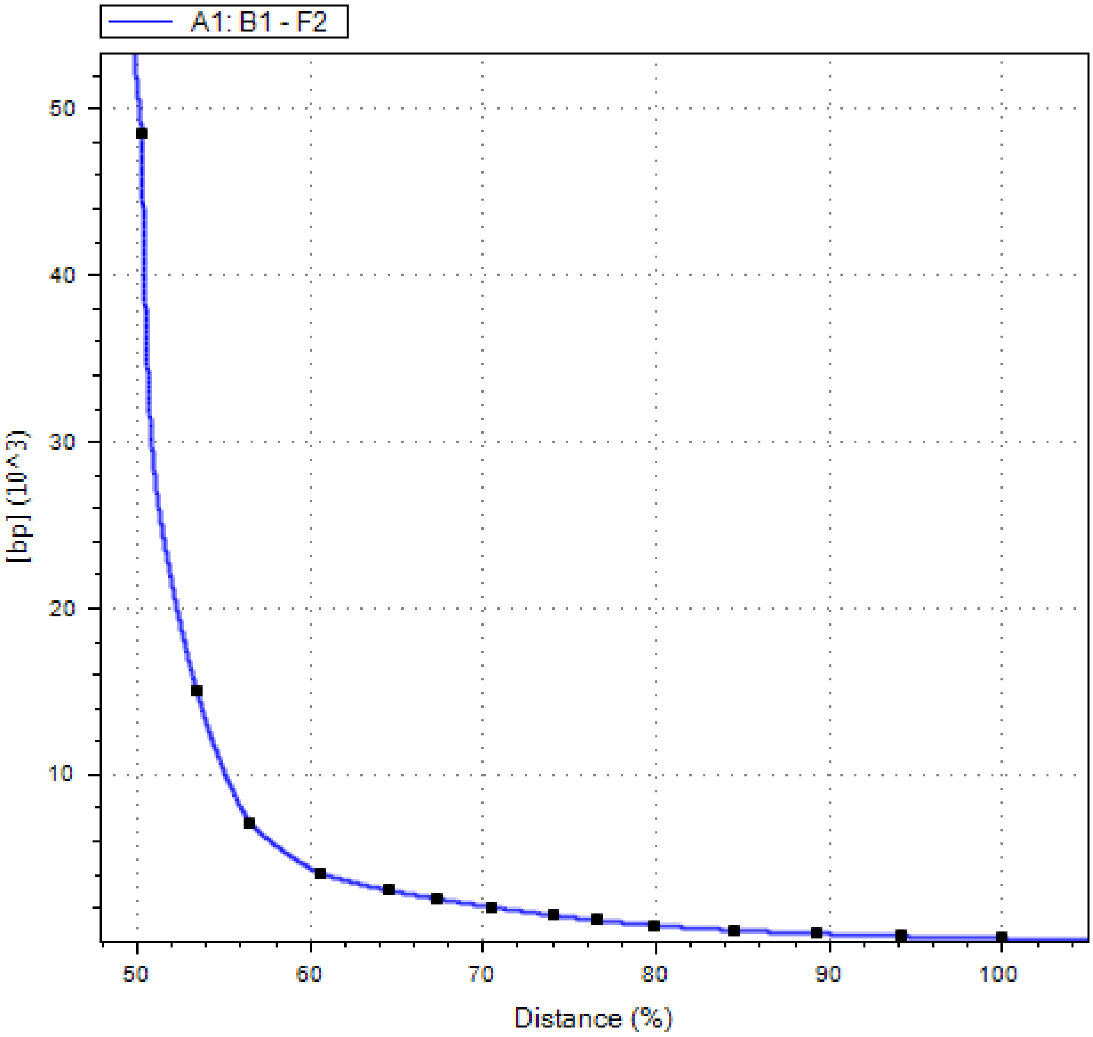

Concentration Settings

Calibration mode: Lower Marker
Normalise peaks from: Lower Marker
Fitting type: Linear Regression

 © Agilent Technologies, Inc. 2016

Experiment Properties

Run Properties

**Analysis Software Version**	2.1.38.8716
**Filename**	C:\Users\admin\Desktop\AnneSophie\2019-september-october-herbarium\2019-10-27-01 after 25 cycles PCR and size selection.gDNA
**Assay**	Genomic DNA
**Run End Date**	27-Oct-2019 8:18 PM
**Last Saved Under Version**	2.1.38.8716
**DIN Version**	2.1.38.8716
**Study**	
**Comments**	

ScreenTape Device 1

**Username**	admin
**ScreenTape Device ID**	01-S025-191007-01-000543
**Expiry Date**	10-Nov-2019
**ScreenTape Device History**	First run 27-Oct-2019, 1 run performed
**Temperature [°C]**	22.5
**Electrophoresis Time [s]**	229
**Instrument Type**	6655
**Instrument Serial Number**	03-PM405
**Notes**	
**ScreenTape Device Run Date**	27-Oct-2019 7:56 PM

Controller Environment

**Computer**	LAB3210150
**Instrument Controller Software Version**	A.02.01 SR1
**First Run Analysis Version**	2.1.38.8716
**Operating System**	Microsoft Windows 7 Enterprise

 © Agilent Technologies, Inc. 2016
