# Supplementary material for: Retrieval of long DNA reads from herbarium specimens
Source: AoB Plants. 2023 Nov 8;15(6):plad074. doi: 10.1093/aobpla/plad074 (PMC10735254; doi:10.1093/aobpla/plad074)

Appendix S3: (A) shows the frequency between 0 and 1 % of deamination for the first 25 base pairs at the 3'-ends extremities. (B) shows the frequency between 0 and 1 % of deamination for the first 25 base pairs at the 5'-ends extremities.

(A)

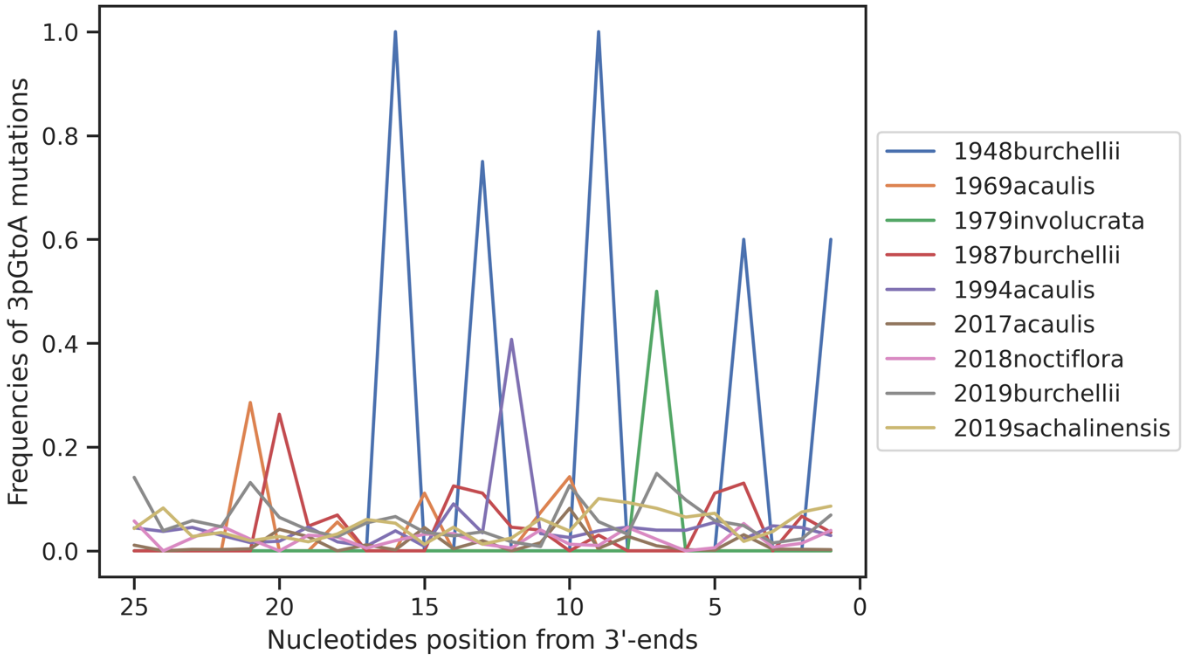

(B)

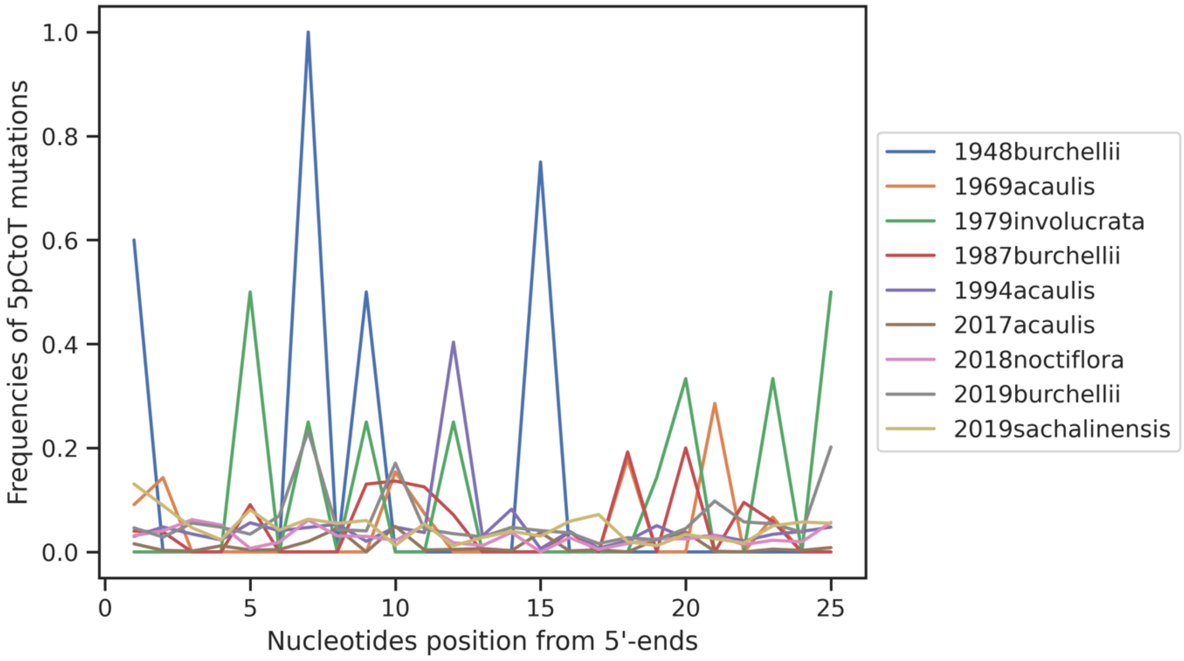

Appendix S5: Proportion of fragment > 1 kb after DNA extraction and each AMPure XP beads size selection. The proportions are calculated from the molarity of fragments > 1 kb (nmol/l) assessed for each sample by Agilent Tapestation (appendix S1), divided by the total molarity per sample (appendix 6). **Post-extraction**: proportion of fragments > 1kb after CTAB extraction. **After 1st size selection**: proportion of fragments > 1kb post-extraction size selection. **After 2nd size selection**: proportion of fragments > 1kb after PCR barcoding. **After 3rd size selection**: proportion of fragments > 1kb post-target-capture amplification. **Long barcoded reads**: proportion of barcoded reads > 1kb. **Long mapped reads**: proportion of mapped reads > 1kb.

Samples	Post-extraction	After 1st size selection	After 2nd size selection	After 3rd size selection	Long barcoded reads	Long mapped reads
2019 S. acaulis	0,28	0,35	0,19	0,78	0,91	0,89
2019 S. burchellii	0,26	0,41	0,17	0,55	0,57	0,28
2018 S. noctiflora	0,2	0,28	0,16	NA	0,65	0,8
2017 S. sachalinensis	0,22	0,26	0,18	0,71	0,77	0,78
1994 S. acaulis	0,28	0,35	0,21	0,5	0,85	0,89
1987 S. burchellii	0,11	0,13	0,25	0,58	0,73	0,67
1981 S. noctiflora	0,08	0,08	0,22	0,39	0,66	0
1979 S. involucrata	0,08	0,15	0,33	0,17	0,41	0,2
1969 S. acaulis	0,11	0,11	0,38	0,34	0,94	0,93
1959 S. uralensis	0,19	0,13	0,17	0,31	0,8	0
1948 S. burchellii	0,06	0,06	0,13	0,23	0,75	0,08
1932 S. rigens	0,08	0,08	0,18	0,14	0,24	0

Appendix S6: The number of BLAST hits per sample show the number of reads > 600 bp belonging either to the sample of interest or inferred as "cross-contaminants" with 95 % pairwise identity. The number of BLAST hits and the read length of each hit are plotted per sample and per taxonomic group (*e.i,* sections in the genus *Silene* referred in table 1 in main text). Samples "1969 *S. acaulis*", "1994 *S. acaulis*" and "2019 *S. acaulis*" belong to the section Siphonomorpha Otth (Table 1). Therefore, we expected most of the reads > 600 bp blasting against this taxonomic group, which is what we observe in appendix S6. Samples "1948 *S. burchellii*", "1987 *S. burchellii*" and "2019 *S. burchellii"* belong to the section Silene (L.) L. (Table 1). As expected, most of the barcoded reads > 600 bp map against this group (appendix S6), except for "1948 S. burchellii", the oldest sample of the group. This sample has as many > 600 bp reads blasting against Silene (L.) L. than blasting against other sections (appendix S6). Based on its higher cross-contamination level, it might also be considered as failed. Samples "1959 *S. uralensis*", "1979 *S. involucrata*" and "2017 *S. sachalinensis*" belong to the taxonomic section Physolychnis (Bentham) Bocquet (Table 1). Most of the barcoded reads > 600 bp mapped against this group (appendix S6). "2018 *S. noctiflora*" is included in the section Elisanthe (Fenzl) Fenzl and most of its barcoded reads > 600 bp blast against the group it belongs to.

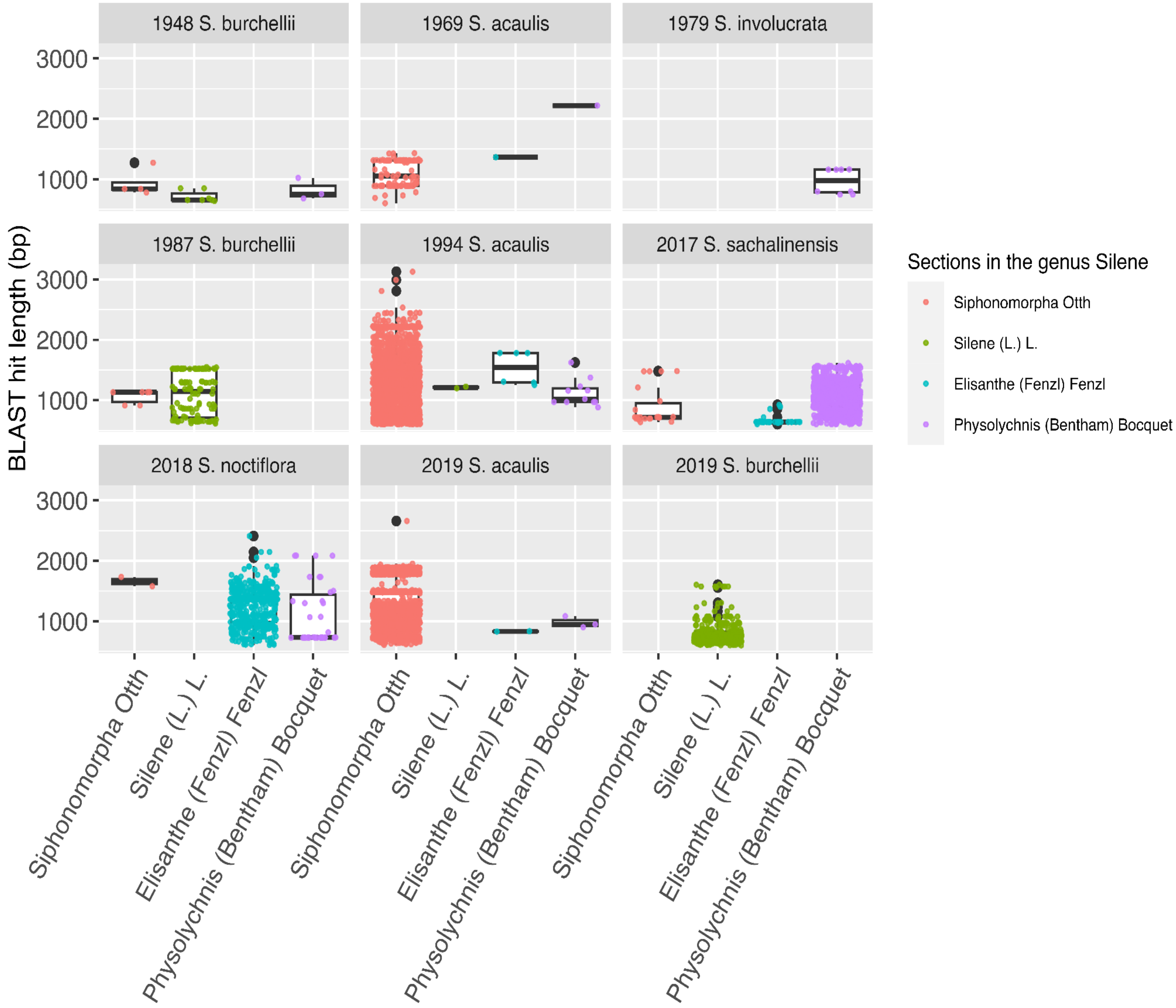

Appendix S8: libraries with higher molarity of long fragments yield more long on-target reads.

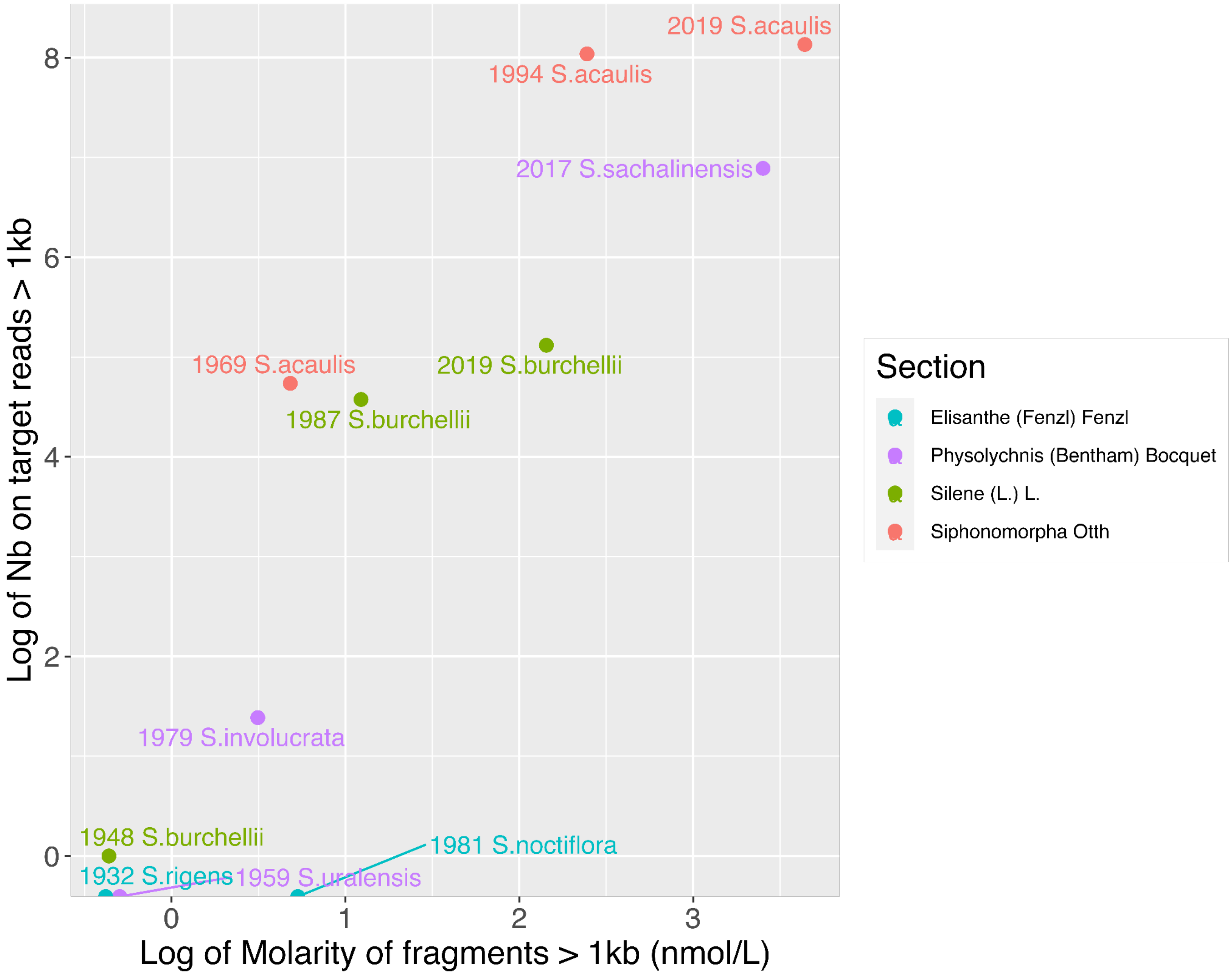

Supplement: plad074_suppl_Supplementary_Appendix_S3_S5_S6_S8 [file plad074_suppl_supplementary_appendix_s3_s5_s6_s8.pdf]
